# Transcriptome sequencing and marker development in winged bean (*Psophocarpus tetragonolobus*; Leguminosae)

**DOI:** 10.1038/srep29070

**Published:** 2016-06-30

**Authors:** Mohammad Vatanparast, Prateek Shetty, Ratan Chopra, Jeff J. Doyle, N. Sathyanarayana, Ashley N. Egan

**Affiliations:** 1 US National Herbarium (US), Department of Botany, Smithsonian Institution-NMNH, 10th and Constitution Ave, Washington DC, 20013, USA; 2 Department of Plant Biology, Michigan State University, 612 Wilson Road, Room 166, East Lansing, MI, 48824, USA; 3 United States Department of Agriculture, Agriculture Research Service, 3810 4th St., Lubbock, TX, 79415, USA; 4 Section of Plant Breeding & Genetics, School of Integrative Plant Science, Cornell University, 412 Mann Library, Ithaca, NY, 14853, USA; 5 Department of Botany, Sikkim University, 5th Mile, Tadong, Gangtok, Sikkim, 737102, India

## Abstract

Winged bean, *Psophocarpus tetragonolobus* (L.) DC., is similar to soybean in yield and nutritional value but more viable in tropical conditions. Here, we strengthen genetic resources for this orphan crop by producing a *de novo* transcriptome assembly and annotation of two Sri Lankan accessions (denoted herein as CPP34 [PI 491423] and CPP37 [PI 639033]), developing simple sequence repeat (SSR) markers, and identifying single nucleotide polymorphisms (SNPs) between geographically separated genotypes. A combined assembly based on 804,757 reads from two accessions produced 16,115 contigs with an N50 of 889 bp, over 90% of which has significant sequence similarity to other legumes. Combining contigs with singletons produced 97,241 transcripts. We identified 12,956 SSRs, including 2,594 repeats for which primers were designed and 5,190 high-confidence SNPs between Sri Lankan and Nigerian genotypes. The transcriptomic data sets generated here provide new resources for gene discovery and marker development in this orphan crop, and will be vital for future plant breeding efforts. We also analyzed the soybean trypsin inhibitor (STI) gene family, important plant defense genes, in the context of related legumes and found evidence for radiation of the Kunitz trypsin inhibitor (KTI) gene family within winged bean.

Winged bean (*Psophocarpus tetragonolobus* (L.) DC.) is a promising legume crop of the world’s tropical regions. It is predominantly self-pollinated and possesses a twining habit, tuberous roots, longitudinally winged pods, and both annual and perennial growth forms^1^. The genus *Psophocarpus* Neck. ex DC. comprises 10 species. Excluding cultivated winged bean, all other species are wild and native to Africa, Madagascar and the Mascarene Islands in the Indian Ocean^2^. Winged bean is speculated to have originated from the progenitor species *P. grandiflorus* R. Wilczek and is now cultivated extensively in Papua New Guinea and Southeast Asia, and to a lesser extent in Africa^1^,^2^. Winged bean has a diploid genome (2n = 2× = 18)^3^ and an estimated genome size of 1.22 Gbp/C (A.N. Egan, unpublished data).

Every part of the winged bean is edible, earning it the distinction of “*Supermarket on a stalk*”^4^. The exceptional nutritional quality of this plant, and the fact that it provides suitable human food sources at all stages of its life cycle, makes it a promising candidate for increased, widespread use in protein deficient tropical areas of the world. The young pods contain 2.4 grams (g) protein per 100 g of edible portion; the dried tubers and seeds contain 8–20% and 34% protein, respectively, as well as a high oil contents (18%) - traits which have earned it the name “*soybean of the tropics*”^5^. If both seed and tuber yields are combined, winged bean can outperform many other legume crops that are conventionally grown in the tropics and thus offers a cheap nutritional food source. Consequently, it is projected as a promising alternative to soybean in areas where soybean cultivation success is marginal.

Since the 1975 publication by the US National Academy of Sciences of *The Winged Bean: A High Protein Crop for the Tropics*^6^, considerable effort has been focused on studying the nutritional quality and climatic and ecological tolerances of the plant^7^,^8^. Winged bean reportedly possesses anti-nutritional factors such as phytoglutenins, cyanogenic glycosides, tannins, lectins, flatulence factors, and saponins^9^. However, processing using moist heat or soaking has been shown to safely eliminate these substances. Research efforts concerning such anti-nutritional components have yielded significant knowledge concerning trypsin, a serine protease that acts to hydrolyze proteins as part of vertebrate digestion, and trypsin inhibitors, proteins that stop the action of trypsin, thereby interfering with digestion. It has been suggested that trypsin inhibitors play a role in protecting plant tissues against the action of bacterial proteases at the colonization site of pathogenic bacteria^10^. In addition, studies show involvement in defense against insects that suck the phloem sap and against bacteria that invade upon wounding^11^. In biomedical research, these modes of action have made trypsin and trypsin inhibitors vital components of molecular cell research where they are widely used in cell culture to detach cells from tissue culture plates. Since their first discovery in soybeans in 1945^12^, other Kunitz-type trypsin inhibitors have been discovered and characterized from winged bean^13^,^14^, predominantly from seeds.

It is hard to find another high rainfall-adapted tropical legume with as many desirable characteristics as winged bean^1^. However, much needs to be done in terms of breeding efforts, especially to develop self-supporting, determinate cultivars bearing large numbers of relatively small pods having nutritious seeds and tubers, and cultivars resistant to biotic and abiotic stresses. Considerable variability for growth vigor and quantitative characters such as protein and oil content as well as photoperiodic responses has been recorded^15^. Several beneficial mutants were recovered during the 1970s and 80’s through mutation breeding experiments^16^. However, a recent study using inter-simple sequence repeat (ISSR) markers reported low genetic diversity among the winged bean germplasm collected from different parts of the world^17^. With the advent of genomic tools such as molecular markers, genetic maps, etc., the genetic improvement of underutilized crops has been greatly facilitated, enabling the development of improved genotypes or varieties with enhanced trait values^18^. In the case of winged bean, studies on genomic resource development for enabling basic and applied research on genetics, evolution, ecology and molecular breeding programs are lacking, yet the advent of genomic technologies provides significant prospects for improvement^19^. Transcriptome sequencing is cost-effective and a valuable method for efficient and rapid identification of molecular markers in resource poor plant species^20^.

The present study was undertaken with the following objectives: (a) to generate a set of expressed sequence tag (EST) resources through whole transcriptome analysis based on Roche 454-based transcriptomes for two winged bean accessions from Sri Lanka; (b) to develop a *de novo* assembly for these transcriptomes; (c) to annotate the transcriptome information; and (d) to discover microsatellite markers for future genetic studies. We also compared Sri Lankan accessions to a Nigerian winged bean transcriptome previously sequenced on the Illumina platform (e) to identify Single Nucleotide Polymorphisms (SNPs) evident between the geographically separated genotypes and (f) to present an analysis of the Kunitz trypsin inhibitor gene family in the context of related legumes.

## Results

### Sequencing and *De novo* assembly of winged bean transcriptomes

Pyrosequencing of two Sri Lankan accessions produced comparable sequence output, where genotype CPP34 produced a total of 369,820 single-end reads comprising 136,943,216 bp with an average read length of 574 bp and genotype CPP37 produced a total of 334,639 single-end reads comprising 92,126,948 bp with an average read length of 565 bp (Table 1). Using read count as a proxy, the depth of sequencing across our contigs was similar for the independent *de novo* assemblies, ranging from one to 4,953 reads, with an average read depth of 25 reads per contig for CPP34 and ranging from one to 3,972 reads with an average read depth of 30 reads per contig for CPP37. Comparison of transcripts from the CPP34 and CPP37 independent assemblies (Supplementary file 1, inclusive of Tables S1–S3 and Fig. S1) found fewer than 200 high-confidence SNPs between them (data not shown), equating to approximately one SNP every 150,000 bp. Therefore, reads from the independently sequenced accessions were combined and co-assembled. For the combined assembly (CPP34-7), this translated to 704,459 reads comprising 229,070,164 bases from both accessions (Table 1). Because 454 pyrosequencing produces comparatively long reads (300–800 bp long), unassembled reads, here notated as singletons post-assembly, may potentially represent full-length mRNA transcripts. In order to not lose potential information, singletons of the CPP34-7 were extracted and appended to the final assembly of CPP34-7 and used in the Gene Ontology (GO) and SNP analyses.

### Functional annotation & legume sequence similarity

**Figure 1:**
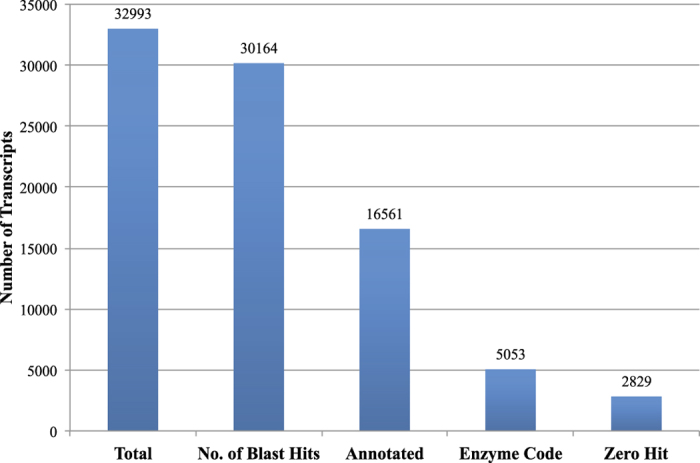
Summary of gene annotation analysis. Zero Hit refers to those in BLAST step without hits.

**Figure 2:**
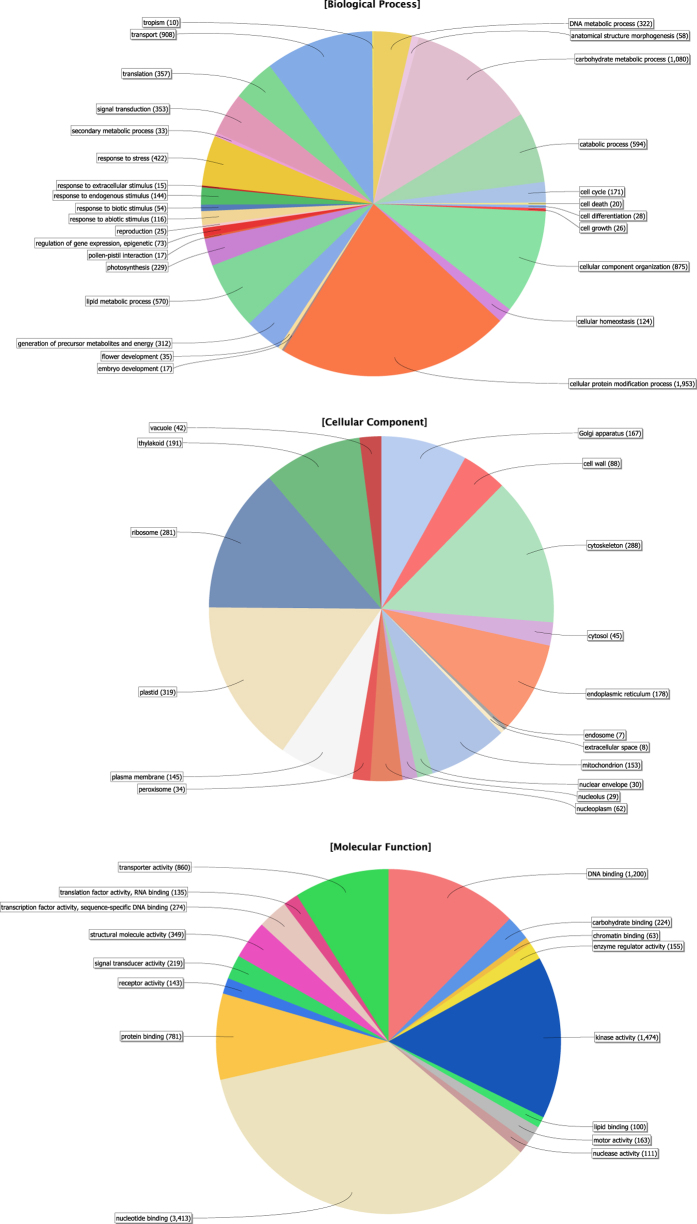
Gene ontology classifications of winged bean annotated transcripts. Numbers indicate the number of sequences associated with the particular GO term in each category.

For the GO analysis, the combined assembly of CPP34-7 was used with inclusion of singletons (16,115 contigs plus 81,126 singletons, Table 1). Using a total of 97,241 transcripts, TransDecoder could track 33,038 transcripts against BLAST and Pfam databases. Of these 33,038 transcripts, BLAST searches against NCBI’s nr database retrieved 32,993 transcripts with hits (see Supplementary file 2), discarding 45 transcripts that had zero hits in NCBI. Therefore, 64,248 (66%) of our original 97,241 transcripts did not hit any known gene or DNA region in NCBI and Pfam databases, of which 62,783 were singletons. Thus, 79% of singletons were discarded in the BLAST searching steps due to a lack of annotation. Of the 32,993 transcripts with BLAST hits, the GO analysis determined GO ID and enzyme code (EC) assignments for 16,561 (50.1%) with full or partial annotations (Fig. 1 in text, and see Supplementary file 2). Of the 16,561 annotated transcripts, 5,053 have predicted functions (EC codes). Overall, 2,829 transcripts were not functionally annotated by Blast2GO (zero hits) of which 1,932 (68%) corresponded to singletons. Participation of genes in a particular biological process and molecular function are shown in Fig. 2. Several transcripts were assigned to more than one GO term; therefore, the total number of GO terms obtained for our dataset was higher than the total number of transcripts. In total, 47,178 GO terms were retrieved, with 46.2%, 37% and 16.8%, corresponding to the molecular functions (MF), biological processes (BP), and cellular components (CC) categories, respectively. In the MF category, nucleotide binding (number of sequences = 3,413), kinase activity (1,474) and DNA binding (1,200) had the highest number of assigned sequences. In the BP category, cellular protein modification (1,953), carbohydrate metabolic processes (1,080) and transport (908) were the majority and in the case of CC, genes involved in the plastid (319), cytoskeleton (288) and ribosome (281) activities were highly represented (Fig. 2).

**Figure 3:**
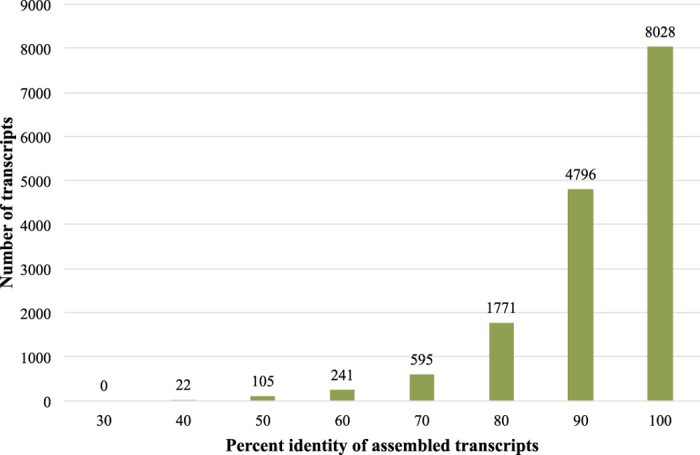
% Identity of CPP34-7 contigs against legume protein databases.

**Figure 4:**
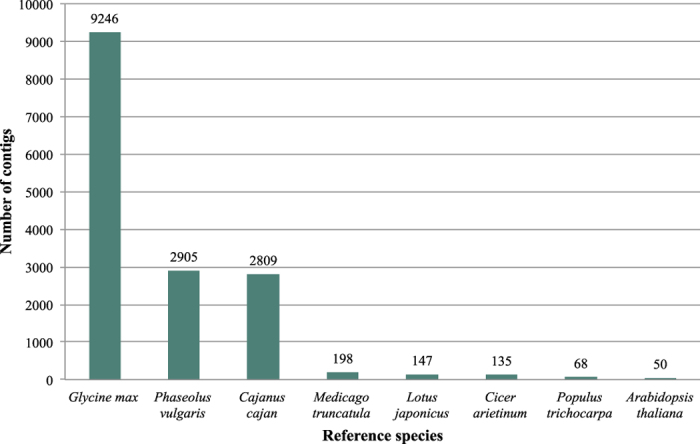
Legume sequence similarity analysis. Relative numbers of contigs that had significant sequence similarity by species for CPP34-7 contigs.

A comparison of our assembled contigs against other legume NCBI protein sequence databases from chickpea (*Cicer arietinum* L.), pigeon pea (*Cajanus cajan* (L.) Huth), soybean (*Glycine max* (L.) Merr.), common bean (*Phaseolus vulgaris* L.), *Medicago truncatula* Gaertn., and *Lotus japonicus* (Regel) K.Larsen using the BLASTX program from NCBI showed that 15,558 of 16,115 (96.5%) contigs from the CPP34-7 assembly had significant sequence similarity to sequences in one or more legume protein databases. About 90.5% of the 16,115 contigs had ≥80% sequence identity (Fig. 3). The majority of the contigs (57.3%) were most similar to *G. max* (Fig. 4), a finding that, at first glance, seems to contradict that expected based on evolutionary relationships of legume lineages, but is likely due to the relative over-representation of genes within the soybean genome due to i) recent whole genome duplication and ii) a much higher level and standard of annotation and gene discovery relative to other legume genomes. Differences in evolutionary rates across lineages may also impact this outcome. In relation to *Phaseolus vulgaris*, it is known that *Phaseolus* has a higher mutation rate than *Glycine* and related lineages^21^,^22^, which could increase the divergence, and thereby decrease the best-BLAST hits, of *Psophocarpus* against *Phaseolus* relative to *Glycine*. However, this explanation is invoked with caution given that it assumes similar relative rates between *Glycine* and *Psophocarpus*, information that is beyond the scope of this project.

### Identification of transcription factors

**Figure 5:**
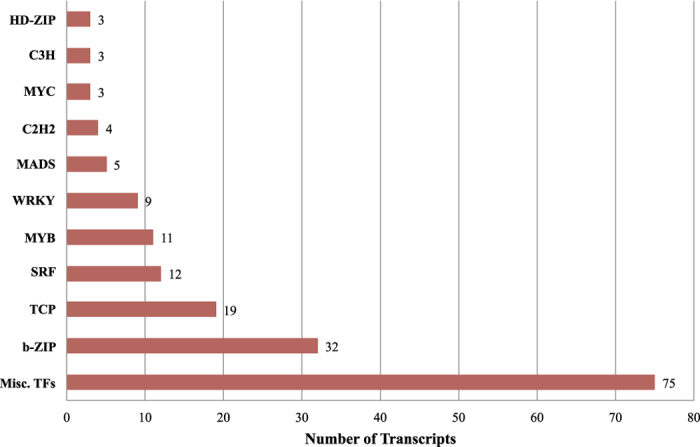
Transcription factor family analysis. Number of transcription factors determined within the CPP34-7 assembly by transcription factor family.

In the overall GO analysis, 274 transcripts were annotated as transcription factors (TFs) (Fig. 2). Of the 16,115 contigs, 176 putative winged bean transcription factor genes, distributed in at least ten families, were identified representing 1.1% of winged bean transcripts, which were assigned to different categories. Among these, basic leucine zipper (bZIP; 32), Teosinte-Branched1/Cycloidea/PCF (TCP; 19), MADS (17), MYB (11) and WRKY (9) were among the top five categories (Fig. 5.). The overall distribution of transcription factor encoding transcripts among the various known protein families is very similar to that of soybean and other legumes. However, almost all families showed minor species specific differences (for example, bZIP, MYB, WRKY etc.) with regard to TF gene families reported for *Lotus*, *Medicago* and *Glycine max.*

### Identification of simple sequence repeats

**Figure 6:**
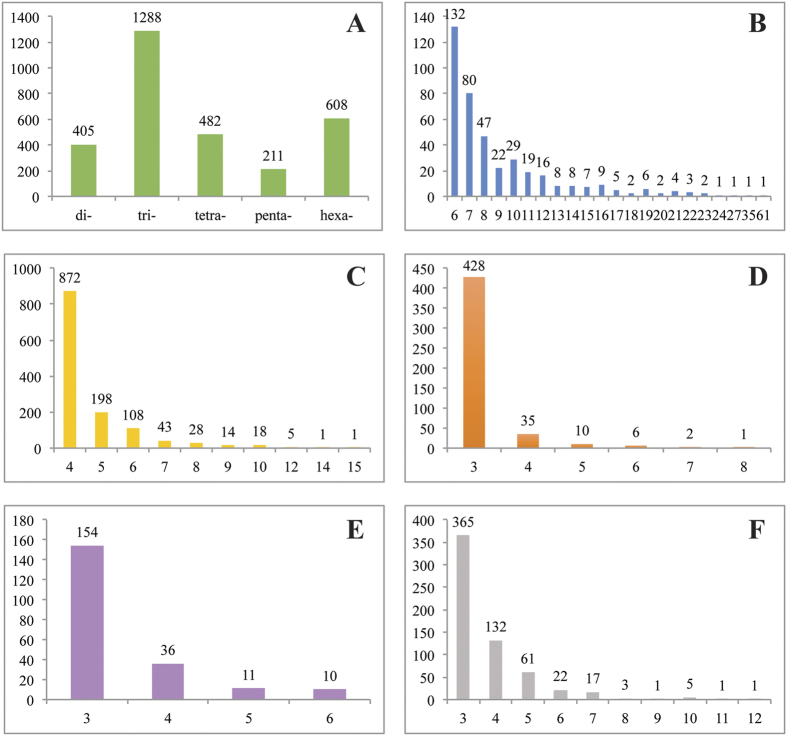
Results of microsatellite SSR analyses. (**A**) Distribution of the 2,994 perfect SSRs across different repeat size classes. Distribution of the number of repeats for (**B**) dimers (**C**) trimers (**D**) tetramers (**E**) pentamers and (**F**) hexamers.

The SSR analysis detected 10,984 perfect SSRs, 13 imperfect SSRs, and 1,959 compound SSRs, for a total of 12,956 SSRs (see Supplementary file 3). Of the 10,984 primary SSRs, 57 were adenine (A: 30) or thymine (T: 27) monomers with at least 13 repeats. These were assumed to represent remnants of mRNA poly-A tails and were thus removed prior to primer prediction. No runs of 12 of more cytosine or guanine monomeric repeats were found. Nearly three-quarters of the remaining 10,927 perfect SSRs (7,933) were hexamers with only two repeats. Although these 12-mers may be useful as linkage markers, the low number of repeat units would likely take these out of the microsatellite category. The remaining 2,994 perfect SSRs were distributed across di-, tri-, tetra-, penta-, and hexamer SSRs (Fig. 6) and were used for primer creation. The majority (63%) of SSRs were detected in the tri- and hexamer categories (Fig. 6A). In general, the number of SSRs detected in each size category decreased with increasing repeat number (Fig. 6B–F). Primers were successfully created for 2,594 SSRs with product sizes ranging from 100 to 280 bp (see Supplementary file 4). Analysis of the primed SSRs showed bias towards certain di- and tri- repeat type motifs (Table 2).

### Single nucleotide polymorphism discovery

GS Reference Mapper mapped 87.7% of reads from Chapman^23^ onto the CPP34-7 reference ‘genome’ which consisted of the 97,241 transcripts. Of the 14,571,393 bp of mapped reads, we identified 113,757 SNPs with >95% confidence from the 454HCDiffs file (available upon request), suggesting a SNP frequency of one in 128 bp of coding regions. Interestingly, the majority of high-confidence SNPs were found within singletons (91,686; 80.6%) vs. contigs (22,071; 19.4%), a higher percentage than expected given that singletons make up 67.9% of total transcript length. As a conservative measure, we filtered SNPs based on allele frequency from >95–100% confidence levels and those having >20× coverage (Table 3), producing a total of 13,091 SNPs distributed across 10,176 transcripts of which 5,196 (39.7%) were from contigs, representing 1 SNP every 1,113 bp. The subsequent increase in the proportion of SNPs within contigs is expected in this case given that more highly expressed genes will be more likely to be represented by >20× coverage and are most likely to assemble into contigs. Lastly, we removed all single nucleotide indels (7,665 of the 13,091) and those length variants that involved insertions or deletions of one or more nucleotides alone (i.e. those without point mutations involved in the length variants), resulting in a high-confidence set of 5,190 SNPs, 96% of which are one-to-one point mutations (see Supplemental file 5). Within the 5,190 SNPs, 151 unique SNP patterns were found and 211 (4%) SNPs were length variants involving one or more point mutation within the length variant. Of the 4,979 one-to-one polymorphisms, 3,433 (68.9%) were transitions and 1,546 (31.1%) were transversions, producing a transition:transversion ratio of 2.22.

### Kunitz-type trypsin inhibitor gene family analysis

**Figure 7:**
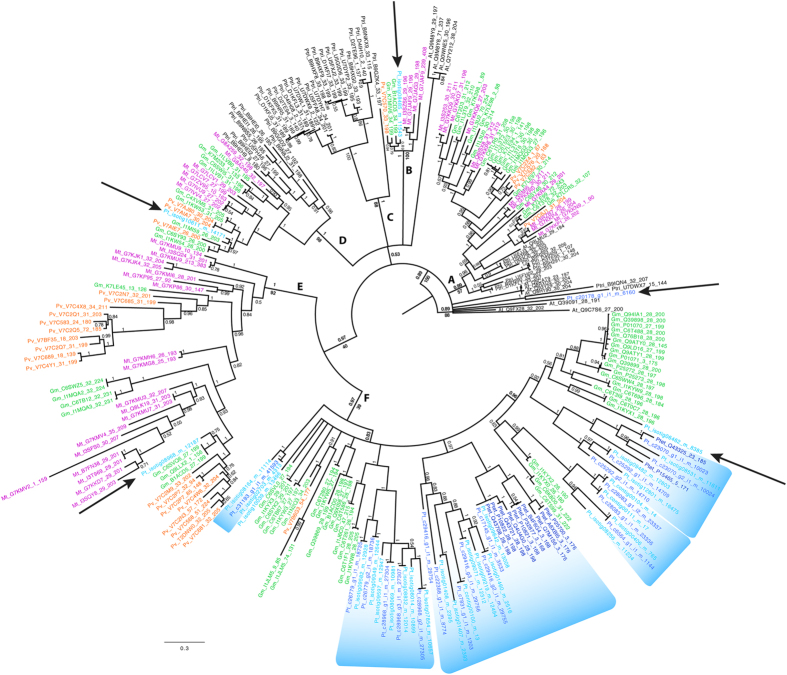
Gene tree of Kunitz trypsin inhibitor gene family. Non-legume sequences: *Arabidopsis thaliana* (At; black), *Populus trichocarpa* (Ptri; black). Legume sequences: *Medicago truncatula* (Mt; pink), *Phaseolus vulgaris* (Pv; orange), *Glycine max* (Gm; green), and *Psophocarpus tetragonolobus* from Pfam database (Ptet; navy blue), Chapman (2015) transcriptome (Pt_c; royal blue), and CPP34-7 (Pt_isotig/contig; aqua blue). Sequence notation is species abbreviation followed by Pfam accession number, contig/isotig number, or read, followed by the range of amino acids used in the alignment. Numbers at the nodes are posterior probability values and bootstrap supports. Subclades A–F are labeled. *Psophocarpus* clades are indicated by arrows or blue banding. Tree rooted arbitrarily at an *Arabidopsis* clade.

We identified 28 contigs from CPP34-7 and 20 contigs from the Chapman^23^ transcriptome assembly corresponding to the Kunitz trypsin inhibitor (KTI) gene family within the *Psophocarpus* transcriptome (see Supplementary file 6). Due to the large number of paralogues in each species, there is no obvious criterion available for rooting this tree, so it was rooted with the largest clade of non-legume sequences, a clade of *Arabidopsis* sequences. Given this rooting, the Bayesian soybean trypsin inhibitor (STI) gene tree has a polytomous backbone and suggests six distinct subclades based on relatively high posterior probability and bootstrap support, here labeled as A-F (Fig. 7 in text; and see Supplementary file 7). The dominant feature of the tree (regardless of rooting) is a lack of clear orthologous relationships across taxa, with evidence of lineage-specific amplification of STI and KTI genes in each species. For example, subclade A comprises two clades made up of only *Populus trichocarpa* Torr. & A. Gray sequences and an *Arabidopsis thaliana* (L.) Heynh. STI member as well as a number of clades containing *Glycine, Phaseolus,* or *Medicago* gene family members, but with no *Psophocarpus* sequences included, whereas subclade C comprises *Populus* sequences only, illustrating a major intra-specific STI radiation (Fig. 7). The vast majority of *Psophocarpus* sequences cluster in clade F, along with many *Glycine* and a single *Phaseolus* sequence. Of the *Psophocarpus* sequences in subclade F, 15 contigs are paired between CPP34-7 and Chapman, forming sister groups that likely represent the same gene in each transcriptome, whereas 13 are unique (Fig. 7). Subclade F illustrates lineage-specific KTI expansion in both *Psophocarpus* and *Glycine*. All *Psophocarpus* sequences obtained from the Pfam or NCBI databases were nested within subclade F, where the majority of the Pfam sequences appeared as monophyletic clades with a contig each from CPP34-7 and Chapman nested therein (Fig. 7).

## Discussion

The legumes represent the third largest family of the flowering plants, many of which are important sources of food, fodder, oil, fiber and medicines. However, with the exception of common pulse crops such as soybean, common bean, etc., a large number of legumes have remained underutilized due to poorly developed infrastructure, especially for genetic and genomic resources^24^. The advent of genomic technologies has brightened the prospects for such orphan crops^20^,^25^,^26^, with recent research focusing on lentil (*Lens culinaris* Medik.^27^), chickpea^28^, grass pea (*Lathyrus sativus* L.^29^), and a number of *Vigna* species^30^,^31^, among others. Winged bean represents a promising alternative to protein-rich soybean for tropical regions of the world that house nearly 40% of the world population, of which nearly one third is protein deficient, and many of whom are women and children^32^. Genomics assisted breeding and enabling biotechnologies that stem from it offer significant promise for targeted genetic improvement of nutritional and other quality traits in winged bean, thus aiding in the development of a low input, high quality legume-based protein diet for these parts of the world. Our combined assembly presents a genetic resource that can be mined for future genetic improvement and plant breeding initiatives. This paper reports development of genetic resources, including molecular markers, in winged bean, in addition to insights into the divergence of the Kunitz-type trypsin inhibitors, which are important anti-nutritive agents in winged bean and other legumes.

In this study, we were able to annotate 32,993 (34%) transcripts from the winged bean combined assembly (CPP34-7; Fig. 2). Schmutz *et al.*^21^ annotated 27,197 protein-coding genes and 31,638 protein-coding transcripts from the *Phaseolus vulgaris* genome, suggesting that our annotated gene complement is reasonable, although it is likely that our transcriptomes do not provide a full gene complement due to low sequencing depth. Our level of unannotated transcripts is similar to results reported from other non-model organisms, including chickpea^33^ and field pea^34^. These unidentified transcripts are likely due to: 1) correspondence to non-coding regions or pseudogenes, 2) short length of transcripts, or 3) novel coding genes that have yet to be described. Cellular, metabolic and transport processes were among the most highly represented groups in terms of GO analysis, as expected given that flower buds, young leaves and shoots are undergoing rapid growth and extensive metabolic activities.

Singletons (unassembled reads) in *de novo* transcriptome assemblies stem from such phenomena as differences in assembly algorithms, sequencing errors, artifacts in cDNA library construction, gene expression at low levels, or contamination from other organisms such as bacteria or fungi^35^. Assessing the GC content of a transcriptome assembly can aid in checking for possible contamination as different organisms have different genomic GC content. We compared the GC content in our data against related legumes to check for contamination and found no evidence of it (see Supplementary file 1). In the GO analysis, ~80% of the singletons were discarded in the BLAST step, while the remaining 20% persisted, but only 10% proceeded through the GO annotation. Others have found similar low levels of singleton annotation^36^, yet, this low level of singleton annotation has lead many to throw out unassembled reads. However, given the comparative length of 454 reads, these could easily represent full-length transcripts. Thus, we included singletons in the GO and SNP analyses to evaluate their potential.

Because transcription factors play important roles in regulating plant functions, we paid particular attention to their number and distribution within winged bean and in relation to other legumes. Several TF gene families are preserved across different plant genera, indicating conserved gene regulatory machinery in plants, as has been shown in legumes previously^37^. In this study, we found that 2.4% of the total transcripts are putative transcription factors according to GO analyses, a percentage much lower than the estimated 12% found in soybean (based on ~46,430 protein-coding genes)^38^. However, Libault *et al.*^37^ estimated the number of TF-encoding genes across a number of species and found soybean to have 3–4× the number of TFs relative to *Medicago truncatula* or *Lotus japonicus*, likely due to recent whole genome duplication in soybean. If we compare the estimated number of TFs in *M. truncatula* (1,473)^37^ against the number of putative protein-coding genes in *M. truncatula* (~66,000; phytozome v11; https://phytozome.jgi.doe.gov/pz/portal.html), we come out with a much more similar estimate (2.2%). However, this is likely an underestimate and a shifting target as the annotation of *M. truncatula* is ongoing.

Our overall distribution of transcription factors in winged bean within the known TF families is similar to that in soybean and other legume species, with bZIP, MYB, TCP, and WRKY highly represented^28^,^39^. The TF family most highly-represented in our data was the bZIP family, which includes regulators of many central developmental and physiological processes and abiotic and biotic stress responses^40^,^41^. In addition, elevated levels of expression were also found for TCPs and MYB: TCPs have been characterized in other plant species for their role in growth, development, and sex determination^42^,^43^, whereas the MYB family has been implicated in regulation of disease resistance and water loss regulation via stomatal movement^44^. However, a significant portion of our transcripts comprised several smaller TF families, here classified under the miscellaneous category for want of detailed characterization. Also, we observed minor species-specific differences in the numbers and proportion of our TFs relative to predicted TFs in *Lotus, Medicago* and *Glycine max*^37^. Further investigation is thus needed to elucidate the evolutionary and functional significance of these events in winged bean.

Simple sequence repeats, or microsatellite markers, have long been used for genetic diversity analyses and plant breeding efforts, largely due to their highly polymorphic, co-dominant nature, prevalence throughout the genome, ease of use, and cost-effectiveness^45^. Because they originate in coding regions, SSRs derived from genes have increased amplification success in related species, are useful for assessing functional diversity and for marker-assisted selection, and can act as anchor markers for evolutionary and comparative mapping studies^46^,^47^. While some research has suggested that SSRs derived from coding regions are less polymorphic than their anonymous counterparts^47^, numerous population genetic, evolutionary, and plant breeding studies have found them to have adequate, if not higher, levels of polymorphism within legumes^48^,^49^.

Within our data, we discovered nearly 5,000 perfect or compound genic-SSRs with three or more repeats. After filtering for perfect, simple SSRs, we discovered an unequal distribution across size-classes, with trinucleotide repeats making up the bulk (43%) of filtered SSRs. Given the coding nature of the transcriptome, this finding makes sense as proliferation of tri-nucleotide, in-frame repeats would be more tolerated^46^. The same trend has been noted in other plants, including for legumes *Medicago truncatula*^50^ and peanut (*Arachis hypogaea* L.^51^). Of the 2,594 SSRs for which primers were created, 1,928 (74.3%) were annotated in our Blast2GO analyses, 871 (45.2%) of which are putatively homologous to known proteins while 1,057 (54.8%) were similar to hypothetical, uncharacterized, unknown, or predicted proteins (mostly from *Phaseolus vulgaris* and *Glycine max* genome annotations).

Certain repeat motif types were more prevalent than others in our set of primed SSRs (Table 2), a not-uncommon finding that has been documented in legumes previously^52^. Zhang *et al.*^53^ first documented the bias of microsatellites to AG and AAG motifs in *Arabidopsis*, also noting differences of SSR distributions between 5′ and 3′ untranslated and coding regions, and correlation between trinucleotide repeat motifs and codon usage. In winged bean, the SSR repeat motif type (AG/GA/TC/CT)_n_ represented the majority (77.7%) of all dinucleotide repeats, while motif types (AT/TA)_n_ and (AC/CA/GT/TG)_n_ comprised 13.7% and 8.6%, respectively. Our distribution and ranking of dinucleotide repeat motifs mirrors that in *Arabidopsis* (Table 2). The bias towards the repeat type containing AG and against that of GC has also been found in other plants, including *Phaseolus*^54^, *Myrciaria dubia* (Kunth) McVaugh^55^ and across eukaryotes^56^. Past research has suggested that AG motifs are most prevalent in 5′ untranslated regions^52^,^57^ and possibly are involved in transcription and regulation^53^. As mentioned earlier, 25.7% of primed genic-SSR transcripts are unannotated, some of which may correspond to 5′ untranslated regions where AG motif types are more prevalent. The frequency of trinucleotide repeat motif types was biased towards AAG in our set of primed SSRs, with this motif type comprising 29.1% of the 10 trinucleotide types, followed by the ATC motif type, comprising 13.9%. The ranking of these and other motifs closely resembles that of *Arabidopsis* (Table 2), with the first two most prevalent motifs the same^53^.

SNPs provide another means of assessing genetic variation and, although less polymorphic than SSRs, are abundant and easily obtained via high-throughput sequencing. For example, Rajesh and Muehlbauer^58^ estimated SNP frequency to be one in 66 bp in coding regions and one in 71 bp in genomic regions of chickpea^58^. In another study, Hyten *et al.*^59^ reported 7,000–25,000 predicted SNPs through deep resequencing of soybean by a whole genome sequence approach. In this study, we discovered more than 5,190 high-confidence SNPs between our Sri Lankan samples and the geographically separated Nigerian genotype^23^. SNP markers identified in this study can be used in quantitative trait loci (QTL) mapping, generating linkage maps, genotyping and breeding studies. Validation of SNPs determined herein is beyond the scope of this paper, nevertheless, this list presents a significant resource for future work in plant breeding and genetic diversity assessment^60^ and marks the first SNP markers discovered to date in *Psophocarpus*.

Our high-confidence SNP set included 4,979 one-to-one SNPs (those without length variants and involving changes between a single nucleotide position), equating to a transition:transversion (ts:tv) ratio of 2.22. This bias is commonly observed across SNPs throughout a genome, resulting from rampant methyl cytosine to uracil mutations^61^. Similar ratios were found across SNPs in other legumes^62^,^63^. In total, 44% of SNPs identified were found in singletons, a proportion not unexpected given that 68% of transcript read length is in singletons. But the very fact that alignable and putatively homologous singletons were found across the geographically separated and independently sequenced genotypes provides vindication for their inclusion in transcriptome characterizations, at least for 454 data. However, caution is warranted due to the ‘singleness’ of the unassembled read acting as a reference sequence.

Trypsin inhibitors play important roles in plant development and defense systems and have been studied from various aspects like biotic stress and wounding. These compounds inhibit activity of proteases and are induced by mechanical wounding in leaves, suggesting a strong role as anti-herbivory agents^64^. Trypsin inhibitors present in legumes include KTIs, the Bowman-Birk trypsin inhibitor, and Cowpea trypsin inhibitor. The KTIs were first discovered from soybean in 1945^12^. Since then, a number of trypsin inhibitors have been discovered and characterized from winged bean^14^,^65^, predominantly from seeds, where they are shown to act as insecticidal agents, preventing seed loss during development^66^. The soybean trypsin inhibitor (STI) gene superfamily has been well studied among *Populus* species, where it has been identified as a rapidly evolving gene family and shown to play multiple roles in anti-herbivory and other stress responses^67^. Philippe *et al.*^64^ suggest that the STI gene family has expanded due to repeated gene duplications within poplar relative to other plant species because poplars need strong anti-herbivory actions to maintain their long-lived life cycle. Our study also discovered several *Populus*-specific radiations (subclades A, C, & D; Fig. 7).

In this study, we characterized 28 STI sequences from our CPP34-7 transcripts as well as 20 from the Nigerian winged bean transcriptome^23^. The majority of our STI sequences clustered with *Glycine* in subclade F (Fig. 7), which includes 28 of 32 overall, distinct *Psophocarpus* lineages, 15 of which are corroborated between the CPP34-7 and Chapman transcriptomes. Subclade F includes those proteins originally characterized as KTIs. Expansion of gene family members in *Psophocarpus* can be characterized as lineage (species)-specific or gene-specific (e.g., via tandem gene duplications). The lineage-specific radiations within *Psophocarpus* and *Glycine* may be inflated due to the presence of multiple alleles or alternatively spliced transcripts. Gene-specific amplification of STI family members in poplar is in part due to tandem duplication^64^. KTI genes (with highest sequence similarity to subclade F) are tandemly duplicated within soybean, with at least eight KTI loci linked within 68 kbp on chromosome 8 (between positions 44850000..44918000). Lineage-specific amplification of *Psophocarpus* KTI sequences is evident, and, given the expectation of conserved synteny between soybean and *Psophocarpus*, gene-specific amplification of *Psophocarpus* KTI sequences may be due to recent tandem duplications.

Besides the classically described KTI genes, several other prominent STI genes are present in our gene tree. Subclade B includes a single contig from our *Psophocarpus* transcriptome, with high sequence similarity to miraculin, a glycoprotein that strongly binds to human taste receptors in the presence of acidic compounds, modifying sour tastes into sweet ones^68^. Miraculin is classified into the STI family and encodes the Kunitz motif but differs from other STI or KTI family members in that it forms a homodimer instead of monomers^69^. Subclade D includes a single *Psophocarpus* contig that has high similarity to alpha-amylase/subtilisin inhibitor proteins known to inhibit the activity of insect a-amylase in *Vigna* species, thus protecting against insect attack^70^. Subclade E comprises only legume STI gene sequences, including a single paralog from *Psophocarpus* with high sequence similarity to Kunitz-*type* trypsin inhibitor-*like* 2 proteins.

The unequal distribution of *Psophocarpus* STI sequences across the six subclades may be due to tissue specificity, depth of transcriptome reads, amplification of certain gene subfamilies or gene loss over time. As mentioned earlier, most of the winged bean KTIs currently known were characterized from seeds, yet all of these are present in subclade F, in spite of the fact that the CPP34-7 transcriptome did not include seed transcripts, but was sequenced from young leaves, shoots, and buds. This subclade also includes a *Psophocarpus* nodulin (Ptet_Q43325) expressed in nodules of winged bean, likely as a delayed response of the host plant to *Rhizobium* infection^71^. Expression levels of STI genes in winged bean likely differ across plant tissues, as demonstrated in poplar^64^, and this may be one explanation for unequal distribution of *Psophocarpus* sequences across the gene tree, although inclusion of such tissue-specific genes as nodulins argues against that. Unfortunately, we cannot determine tissue-specific expression of our winged bean STIs due to the fact that our transcriptomes were sequenced from pooled tissue samples. But, given the roles of KTIs in wound and herbivory defense, radiation of KTI genes would be evolutionarily beneficial to large-leaved, highly nutritious plants such as winged bean. Deeper sequencing of the transcriptomes across more tissue types would likely yield other STI gene family members in *Psophocarpus* and provide a more holistic view of STI gene family evolution in the winged bean.

## Materials and Methods

### Plant material

Seeds of two winged bean (*Psophocarpus tetragonolobus*) genotypes were selected from the United States Department of Agriculture (USDA) Germplasm Resources Information Network (GRIN) seed bank. PI 639033 (CPP37) was field collected in 1999 while PI 491423 (CPP34) was donated in 1984, both from Sri Lanka. Seeds were grown to maturity in the greenhouse at Cornell University (Ithaca, NY, USA) for 3 years. Flowering and fruiting were induced by imposing a day length of less than 8 hours. For comparative purposes and to aid in the development of genetic resources for the winged bean, we compared our transcriptomes to an Illumina-based *P. tetragonolobus* transcriptome (SRR1772344) recently published and originally sourced from Nigeria^23^.

### RNA isolation and library preparation

Young leaves, young buds, and young shoots were collected from 3-year old plants into liquid nitrogen to preserve RNA. Total RNA was extracted from each tissue (leaves, shoots, and buds) separately using the Qiagen RNAeasy mini kit according to manufacturer instructions. The quality and quantity of each RNA tissue extract was assessed using a 2100 Bioanalyzer (Agilent Technologies, Santa Clara, CA, USA). All RNA samples had RIN (RNA integrity number) greater than 9.0 and were used for the analysis. RNA concentration was also quantified using the nanodrop 2000c spectrophotometer (NanoDrop Technologies, Inc., Montchanin, DE, USA). Before cDNA library construction, RNA from tissues for each accession was combined in equal molar amounts so as to allow each tissue equal representation in the final library construct. One microgram (ug) of the pooled tissue total RNA extracts were used for subsequent cDNA library construction of each accession using the Clontech SMARTer cDNA synthesis kit (Clontech Laboratories, Inc., Mountain View, CA, USA) according to manufacturer’s instructions but using a 3′ SMART CDS Primer IIA modified to 5′–AAGCAGTGGTATCAACGCAGAGTACTTTTTTGTTTTTTTCTTTTTTTVN–3′ which was purchased from IDT (Integrated DNA Technologies, Inc., CA, USA). cDNA libraries were then purified using the PureLink PCR purification kit (Life Technologies, (Invitrogen), Carlsbad, CA, USA) with Buffer HC which removed all fragments less than 300 bp.

### Transcriptome sequencing

Samples were sequenced using single-end 454 pyrosequencing on the Roche 454 Genome Sequencer FLX (Titanium chemistry) at the Brigham Young University Sequencing Center (Provo, UT, USA). Libraries were tagged with multiplex identifier (MID) barcodes to allow multiplexing of four species together over one titer plate. After sequencing, MID adaptors and primers were removed from reads during pre-processing. Preliminary visualization of data was done in FASTQC v. 0.11.3^72^.

### *De novo* assembly

For the CPP34 transcriptome we used the standard flowgram file (SFF) originally generated by the 454 GS FLX sequencer. However, for CPP37, we started with fasta (FNA) and quality value (QUAL) files. We converted the FNA and QUAL files for CPP37 into a single FASTQ file using a python script. We used the FASTX-Toolkit (http://hannonlab.cshl.edu/fastx_toolkit/index.html) to trim and clean the CPP37 reads: we discarded sequences shorter than 50 bp (-l 50) using FASTX CLIPPER and setting of first base of 15 (-f 15) and last base of 800 (-l 800) using FASTA TRIMMER. Finally, the FASTQ Quality Filter was used with minimum quality score of 20 (-q 20) and minimum percent of included bases of 80 (-p 80). In all steps we used the quality score ASCII offset command (-Q 33) to denote 454 file format. The quality of output reads after cleaning steps was inspected using FASTQC software v. 0.11.3^72^.

To determine the extent of divergence between our two independently sequenced 454-based transcriptomes, CPP34 and CPP37, we initially assembled each transcriptome independently and explored several contemporary assembly strategies, including Trinity^73^, Velvet^74^, MIRA^75^, and GS *De Novo* Assembler (aka Newbler, Roche, USA) (see methods and results in Supplementary file 1). Our initial findings found fewer than 200 high confidence SNPs between assemblies of CPP34 and CPP37 (SNPs were detected between CPP34 and CPP37 the same way they were assessed between Sri Lankan and Nigerian accessions; see SNP methods below), suggesting a high degree of similarity between these two Sri Lankan accessions. Therefore, for subsequent assemblies and analyses we combined the reads from our two Sri Lankan accessions and produced a single assembly, notated as CPP34-7. Ultimately, we chose to use GS *De Novo* Assembler over the other programs because of the reliable output, comparable contig length, the fact that it considers alternative splicing^76^, and that it is a program specifically designed for 454 data. Comparisons for several programs in the past showed that it performed best among other *de novo* assemblers for 454 transcriptome data^77^. Raw reads from CPP34 and CPP37 were combined by co-assembly within GS *De Novo* Assembler v. 2.9 with default settings using a minimum read length of 20, minimum overlap length of 40, minimum overlap identity of 90%, and Isotig threshold of 100.

### Functional annotation

Prior to functional annotation, we identified candidate coding regions and filtered sequences based on a minimum amino acid length of 100 using the TransDecoder program (https://transdecoder.github.io) v. 2.0.1 applied to CPP34-7 contigs plus singletons, using the TransDecoder.LongOrfs command. To identify open reading frames (ORFs) with homology to known proteins and to maximize sensitivity for capturing ORFs that may have functional significance, Blastp and Pfam searches were conducted. The Blastp search was done using the Swissprot database with the E-value of 1E-5 and Pfam search was done using HMMER (http://hmmer.janelia.org), a biosequence analysis program using profile hidden Markov models and the Pfam database (http://pfam.xfam.org). Output files that were generated from the Blastp and Pfam database searches were leveraged by TransDecoder to ensure that peptides with BLAST or domain hits were retained in the set of reported likely coding regions by running the TransDecoder.Predict command. Finally, output of the TransDecoder analysis was used as input for functional annotation using the Blast2GO program^78^. First, we conducted a BLAST search on the output from Transdecoder against the NCBI’s nonredundant (nr) database with the E-value of 1E-5 on the Smithsonian Hydra clusters. These BLAST results were then used as input to Blast2GO to assign Gene Ontology (GO) terms to our DNA regions.

### Sequence similarity with other legumes

To compare our complement of genes characterized from our winged bean transcriptome assembly against typical gene assemblies in other legumes, legume species’ protein sequences ((*Medicago truncatula*, *Glycine max*, *Lotus japonicus*, *Phaseolus vulgaris*, *Cicer arietinum*, and *Cajanus cajan*) along with *Populus trichocarpa* and *Arabidopsis thaliana* protein sequences were downloaded from NCBI. BLASTX searches were performed against the CPP34-7 contigs with E-value of 1E-4, and the top hit for each contig was used for further analysis.

### Transcription factor identification

CPP34-7 transcripts were translated to protein sequences for prediction of transcription factors in the assembly. Translated protein sequences were subjected to prediction using PlantTFDB (http://planttfdb.cbi.pku.edu.cn/), with further linking the prediction to best hits in Arabidopsis. Since not all transcription factors (TFs) could be predicted in the CPP34-7 assembly, we utilized the annotation results of BLASTX searches against legume databases. All identified and predicted transcription factors were further classified into categories.

### Simple sequence repeat identification

To retrieve simple sequence repeat (SSRs; microsatellite) markers and also to design primers, SSR Locator v.1^79^ program was used to detect SSRs across contigs from CPP34-7. A SSR site was defined as a monomer occurring at least 12× with a dimer at least 6×, trimers at least 4×, tetra- and pentamers at least 3×, and hexa- to decamers occurring at least 2×. The space between compound SSRs was set to 100 bp and the space between imperfect SSRs to 5 bp. Primers were produced and reported for primary SSRs only.

### Single nucleotide polymorphism identification

To identify SNPs between *Psophocarpus* transcriptomes, we used the transcripts (contigs + singletons) from our combined assembly CPP34-7 as a reference ‘genome’. We extracted singletons from the original reads and concatenated them with the contigs produced by our CPP34-7 assembly. We queried Chapman’s Nigerian, Illumina-based transcriptome^23^ against our CPP34-7 reference ‘genome’ using the GUI interface of GS Reference Mapper v. 2.9 (454 Life Sciences, Roche, USA) under default settings. We used only high-confidence variants to the reference sequence (454HCDiffs) and further filtered these to those having 20× or greater coverage. Lastly, to ensure the highest SNP call quality for use in future research, we followed the method of Schmutz *et al.*^21^ and discarded any SNPs where i) the reference or variant involved one or more N’s; and or ii) the reference or variant allele was a single nucleotide insertion or deletion or did not involve a point mutation in the length variant.

### Kunitz-type trypsin inhibitor gene family analysis

To reconstruct a gene tree of the STI superfamily, particularly the KTI gene families, and to understand the evolutionary diversification of this gene superfamily in *Psophocarpus* related to other legumes, we obtained available STI sequences for selected legumes and other angiosperms from the Pfam database (http://pfam.xfam.org). In total, 214 accessions were retrieved across *Arabidopsis*, *Populus*, *Medicago*, *Phaseolus*, *Glycine,* and *Psophocarpus*. We downloaded a reference alignment from the Pfam database and used this alignment as a scaffold upon which to align contigs garnered from our transcriptome (see Supplementary file 6). We extracted putative *Psophocarpus* STI regions from our transcriptome (CPP34-7) and Chapman’s^23^ after blasting against a local BLAST database^80^ based on available STI gene sequences obtained from the Pfam database.

We combined our extracted contigs with the Pfam STI sequences, converted the open reading frames to amino acid sequences, and aligned in MAFFT v. 7. 245^81^. Phylogenetic analysis was conducted in RAxML v. 8.1.24^82^ with 1000 rapid bootstrap inferences and using the best substitution model (LG + G) as determined by Prottest v. 3.4^83^. Additionally, Bayesian analysis was conducted using MrBayes v. 3.2.6^84^ under the JTT amino acid model “aamodelpr = fixed(jones)” and gamma rates. Two independent Markov Chain Monte Carlo (MCMC) analyses with 12 simultaneous chains and 25 million generations were run for each analysis. Trees were sampled every 10,000 generations and the first 25% of trees were discarded as burn-in. The convergence of MCMC chains was confirmed with Tracer version 1.6^85^. All runs and parameters were checked to ensure proper mixing as evidenced by effective sample size (ESS) scores being above 200 and the standard deviation of the split frequencies having dropped below 0.01^84^.

## Additional Information

**Accession codes:** Transcriptome datasets supporting the conclusions of this article are available in the NCBI SRA repository under the accession number SRP067662 (raw 454 reads). In addition, several large datasets stemming from analyses of these data are available in Supplementary files.

**How to cite this article**: Vatanparast, M. *et al.* Transcriptome sequencing and marker development in winged bean (*Psophocarpus tetragonolobus*; Leguminosae). *Sci. Rep.* **6**, 29070; doi: 10.1038/srep29070 (2016).

## Supplementary Material

Supplementary File 1

Supplementary File 2

Supplementary File 3

Supplementary File 4

Supplementary File 5

Supplementary File 6

Supplementary File 7

## Figures and Tables

**Table 1:** Sequencing and assembly metrics for independent and combined assemblies using GS *De Novo* Assembler.

Accessions	Genotype CPP34	Genotype CPP37	Combined Assembly (CPP34-7)
Number of raw reads	371,271	433,486	804,757
Number of bases (bp)	191,598,691	213,386,165	404,984,856
Number of reads post-filtering	369,820 (99.6%)	334,639 (77.2%)	704,459 (87.53%)
Number of bases post-filtering	136,943,216 (71.47%)	92,126,948 (43.17%)	178,911,104 (44.17%)
Number of reads aligned	277,351 (50.42%)	259,324 (63.04%)	435,897 (61.88%)
Number of contigs/bp	10,675/6,142,297	8,465/5,070,585	16,115/13,552,130
Avg. contig size (bp)	837	823	875
N50 (bp)	836	842	889
Longest contig (bp)	4,902	3,014	4,667
Number of singletons/bp	62,602/22,081,798	63,795/23,540,672	81,126/28,663,213
Number of transcripts/bp (contigs + singletons)	73,277/28,224,095	72,260/28,611,257	97,241/42,215,343

**Table 2:** Distribution of di- and trinucleotide repeat motif types in winged bean and comparison with *Arabidopsis*.

Dinucleotide Repeat Composition	Number of transcripts	Percentage of Winged bean di- Repeats	Winged bean Rank	Percentage of Arabidopsis di- Repeats
AC/CA/GT/TG	22	8.6	3	8
AG/GA/CT/TC	199	77.7	1	83
AT/TA	35	13.7	2	8.8
CG/GC	0	0	4	0.14
Total	256	100		100
**Trinucleotide Repeat Composition**	**Number of transcripts**	**Percentage of Winged bean tri- Repeats**	**Winged bean Rank**	**Arabidopsis Rank**
AAC/ACA/CAA/GTT/TGT/TTG	134	11.4	4	3
AAG/AGA/GAA/CTT/TCT/TTC	343	29.1	1	1
AAT/ATA/TAA/TTA/TAT/ATT	58	4.9	8	5
ACC/CAC/CCA/GGT/GTG/TGG	118	10.0	5	4
ACG/CGA/GAC/CGT/GTC/TCG	35	3.0	9	9
ACT/CTA/TAC/AGT/TAG/GTA	13	1.1	10	8
AGC/CAG/GCA/TGC/CTG/GCT	136	11.5	3	7
AGG/GGA/GAG/TCC/CTC/CCT	118	10.0	6	6
ATC/CAT/TCA/GAT/ATG/TGA	164	13.9	2	2
CCG/CGC/GCC/GGC/GCG/CGG	59	5.0	7	10
Total	1,178	100		

**Table 3:** Results of single nucleotide polymorphism (SNP) detection between Sri Lankan and Nigerian genotypes by degree of confidence.

Reads	95%	96%	97%	98%	99%	100%	Total
Contigs	43	68	76	94	50	2,552	2,883
Singletons	74	88	93	52	28	1,972	2,307
Total	117	156	169	146	78	4,524	5,190
