# Supplementary material for: Transcriptome sequencing and marker development in winged bean (Psophocarpus tetragonolobus; Leguminosae)
Source: Sci Rep. 2016 Jun 30;6:29070. doi: 10.1038/srep29070 (PMC4928180; doi:10.1038/srep29070)
Supplement: Supplementary File 1 [file srep29070-s1.pdf]

Transcriptome sequencing and marker development in winged bean (*Psophocarpus tetragonolobus*; Leguminosae)

Mohammad Vatanparast[1], Prateek Shetty[2], Ratan Chopra[3], Jeff J. Doyle[4], N. Sathyanarayana[5*] and Ashley N. Egan[1*]

[1]US National Herbarium (US), Department of Botany, Smithsonian Institution-NMNH, 10th and Constitution Ave, Washington DC, 20013, USA.
[2]Department of Plant Biology, Michigan State University, 612 Wilson Road, Room 166, East Lansing, MI, 48824, USA.
[3]United States Department of Agriculture, Agriculture Research Service, 3810 4th St., Lubbock, TX, 79415, USA.
[4]Section of Plant Breeding & Genetics, School of Integrative Plant Science, Cornell University, 412 Mann Library, Ithaca, NY, 14853, USA.
[5]Department of Botany, Sikkim University, 5th Mile, Tadong, Gangtok, Sikkim, 737102, India.

Email Addresses:
Mohammad Vatanparast: Vatanparastm@si.edu
Prateek Shetty: prateekshettys@gmail.com
Ratan Chopra: Ratan.Chopra@ARS.USDA.GOV
Jeff J. Doyle: jjd5@cornell.edu
N. Sathyanarayana: nsathyanarayana@cus.ac.in
Ashley N. Egan: egana@si.edu

**Corresponding authors:*
Ashley N. Egan (primary) N. Sathyanarayana (secondary)

Supplementary File 1. Preliminary independent assemblies and GC content analysis.

This file details the methods and results of *de novo* assemblies using five different assemblers across CPP34 and compares read statistics between the two transcriptomes sequenced. Inclusive of Table S1: read statistics for CPP34 & CPP37; Table S2: assembly metrics for 5 methods across CPP34; Table S3: Mapping statistics for 5 methods across CPP34; Figure S1: Comparison of GC content of CPP34 & CPP37 against various legumes.

Methods

Read Filtering

In order to make the most of our data, only those bases which would have otherwise interfered with the assembly process were clipped to an average quality of 15 over five bases. Adapter regions were identified and removed and homopolymer repeats of more than six bases were clipped. All the data preprocessing was carried out using the Trimmomatic software [1].

Independent Transcriptome Assemblies

Most assemblers currently used are created for short-read (i.e. Illumina) data. To explore the impact of assembler on our transcriptome assembly, preliminary analyses were conducted to test assembly strategies using four different open source *de novo* assemblers: MIRA v.3.4.0.1 [2]; Velvet v. 1.2.03 [3]; Trinity [4]; and GS *de novo* Assembler v 2.6 (formerly Newbler [5]) (Roche, Branford CT, USA). MIRA is a whole genome assembler/mapper and can be used across several different sequencing platforms. Velvet is a set of algorithms designed to make use of short read data to build contigs. Trinity is now the gold standard for Illumina-based assemblies, was initially developed specifically for transcriptome assembly, and is optimized for short-read technologies. The GS *de novo* assembler is specifically designed to assemble sequence data generated on the 454 pyrosequencing platform. Both MIRA and GS *de novo* assembler are based on overlap-layout-consensus (OLC) algorithms and are commonly suggested for use with longer-read NGS technologies, while Velvet and Trinity are based on de Bruijn graph algorithms and commonly used for shorter-read technologies. CAP3 is a basic sequence assembly program and is used here to refine contigs from MIRA.

MIRA was called using --job=denovo, est, accurate, 454 -GE:not=4 -AS:nop=4,sep=1 -SK:nrr=20 454_SETTINGS -AL:mrs=95. GS *de novo* Assembler was run using the cdna switch and 4 multiple threads under default conditions. Trinity was called using parameters --seqType fq --kmer_method inchworm --single seq_selected.pl --CPU 4. Velvet was performed at k-mer lengths from 49 to 69, with the optimal k-mer value of k=68 chosen by VelvetOptimiser [6]. We compared each method via assembly metrics, including the number, average length, maximum length, N50 and N90 value of the assembly. Finally, the reads were mapped back to the final assemblies using Bowtie2 [7] in order to assess the quality of all assemblies generated. All preliminary assemblies for comparative purposes were first run on CPP34 alone.

GC content analysis

Assessing the GC content of a transcriptome assembly can aid in checking for possible contamination as different species have different genomic GC content. Multiple peaks in a GC content histogram may suggest contamination by other species. GC content analysis was carried out on assembled, annotated contigs and as reference, on four other unigene datasets downloaded from NCBI; *Glycine max*, *Medicago truncatula*, *Vigna unguiculata* and *Lotus japonicus*. GC content was calculated using in house perl scripts.

Results & Discussion

Read Filtering

All reads with lengths shorter than 30 bases were discarded (Table S1). The final dataset after processing for genotype CPP34 had a total of 371,271 single-end reads comprising 140,095,060 bases with an average read length of 377 bases and genotype CPP37 had a total of 433,486 reads comprising 158,348,141 bases with an average read length of 365.

	CPP34		CPP37	
	Raw data	Trimmed data	Raw data	Trimmed data
**Number of reads**	371271	371271	433486	433486
**Total bases**	191,598,691	140,095,060	213,386,165	158,348,141
**Max read length**	1201	812	1201	706
**Average read length**	516	377	492	365
**Number of Ns**	0.46%	0.07%	0.93%	0.03%

Table S1: Summary of data generated for winged bean transcriptomes. Ns are the number of ambiguous nucleotide sites in the sequence.

Preliminary independent assemblies

The goal of a *de novo* transcriptome assembly is to assemble as many true transcripts as possible, avoiding chimaeras or split contigs. As estimated from *Arabidopsis*, it is believed that plants have ~27,000 non-redundant genes, although this number will vary depending on ploidy [8]. However, transcriptome assemblies will often produce much higher numbers, due to the presence of alternatively transcribed genes, chimaeras, or split contigs. Assembly results from the four assemblers varied greatly (Table S2), as did the mapping statistics (Table S3). Taken together, these statistics helped to determine the best assembler for our data.

Velvet generated the highest number of contigs, but more than a quarter of them were less than 100 bp in length. The number of contigs may be an overestimate of the true number of gene assemblies due to split contigs or chimaeras. Mapping reads back onto the assembled contigs can help determine the validity of gene assemblies and detect chimaeric transcripts. Velvet had less than 10% of reads map to the contigs, which may suggest a high degree of chimaeras. Velvet utilized the least amount of data and also had the shortest maximum contig length, average length, N50, and N90 of all five assembly strategies. Taken together, these stats suggest that Velvet generated the poorest assembly.

**Assembler**	**Velvet**	**GS *de novo* Assembler**	**Trinity**	**MIRA**	**MIRA +CAP3**
Total number of contigs	76146	13065	21740	48728	32157
Contigs (100bp or greater) (% of total contigs)	56263 (73.9%)	13053 (99.9%)	21740 (100%)	48728 (100%)	32157 (100%)
Maximum length (bp)	1295	4915	4363	2845	4496
Average length (bp)	237	523	656	604	699
N50	244	667	780	645	740
N90	151	282	358	429	470

Table S2: Assembly metrics. All assemblies were generated for CPP34.

Assembler	Velvet	GS *de novo* Assembler	Trinity	MIRA	MIRA +CAP3
Total number of mapped reads (overall alignment rate)	32046 (8.6%)	207347 (55.9%)	231309 (62.3%)	**285968 (77%)**	284465 (76.6%)
No. reads mapped once (% of mapped reads)	25419 (79.3%)	**197838 (95.4%)**	182239 (78.8%)	87696 (30.7%)	112308 (39.5%)
No. reads mapped more than once (% of mapped reads)	6627 (20.7%)	**9509 (4.6%)**	49070 (21.2%)	198272 (69.3%)	172157 (60.5%)
Total number of unmapped reads	339225 (91.4%)	163924 (44.1%)	139962 (37.7%)	**85303 (23%)**	86806 (23.4%)

Table S3: Mapping statistics. The number in bold represents the assembler that performed best in that category.

MIRA produced the second highest number of contigs. Even though MIRA+CAP3 produced fewer contigs than MIRA alone, the number is reasonable and MIRA+CAP3 produced a better overall assembly than MIRA as evidenced by the increase in maximum contig length, average length, N50, and N90. MIRA+CAP3 produced the assembly with the best N50 and N90, as well as the longest average contig length, and also had the highest overall alignment rate, mapping 76-77% of reads back onto contigs. However, the majority of reads mapped more than once, suggesting the presence of chimaeric or fractured contig assemblies.

Trinity produced a median number of contigs, coupled with relatively high maximum contig length, average contig length, and median N90. Of all five assemblers, Trinity had the highest N50. Although it had a lower overall alignment rate than the MIRA assemblies, Trinity had less than half the number of reads that mapped more than once, with almost 80% of reads mapping uniquely to the contig assemblies. This may indicate relatively few chimaeras.

Although GS *de novo* assembler produced the fewest contigs, it boasted the longest maximum contig length, a relatively high N50, and a median average contig length. However, GS *de novo* assembler had a median overall alignment rate and a relatively low N90. That said, GS *de novo* assembler had the highest number of uniquely mapped reads, suggesting that few chimaeras were created.

Comparing assembly algorithms, de Bruijn graphs vs OLC algorithms, the latter wins out for use with our 454 data: MIRA+CAP3 produced a reasonable number of contigs, had the highest maximum and average contig lengths, the best N90, and the second best N50, whereas GS *de novo* assembler had the highest number of read map uniquely, suggesting the purest contig assembly, even though fewer contigs were created. The suggestion that OLC algorithms are more appropriate for longer reads such as 454 data is supported here.

Ultimately, we chose to use GS *de novo* Assembler over the other programs because of the reliable output (fewer chimaeras), comparable contig length, the fact that it considers alternative splicing [9], and that it is a program specifically designed for 454 data. It should be

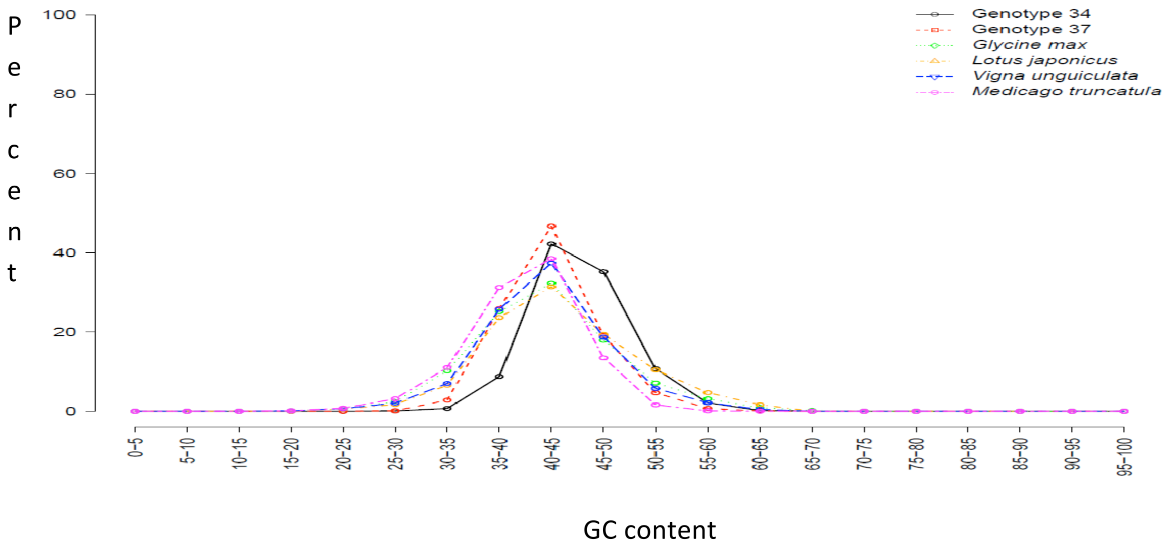

Figure S1: GC content analysis.

noted that assembly metrics for GS *de novo* Assembler differ here as compared to the analyses in the main text, which were run on a newer version of Newbler, which is packaged into the GS *de novo* Assembler.

GC content analysis

GC content analysis showed that all legume references had comparable GC content profiles (Figure S1). Genotypes CPP34 and CPP37 had a mean GC% of 45.16% and 42.44%, respectively. The GC content profiles showed a narrower range of distribution when compared to the references, but followed the overall pattern. CPP34 had a higher abundance of contigs within 45-50% GC content, while CPP37 showed higher abundance of contigs in the range of 35-40% GC content. However, such variation could be due to an incomplete transcriptome. Deeper sequencing might reveal the true GC content profile for winged bean.

References

1. Bolger AM, Lohse M, Usadel B: **Trimmomatic: a flexible trimmer for Illumina sequence data**. *Bioinformatics* 2014:btu170.
2. Chevreux B, Wetter T, Suhai S: **Genome sequence assembly using trace signals and additional sequence information**. In: *German conference on bioinformatics: 1999*; 1999: 45-56.
3. Zerbino DR, Birney E: **Velvet: algorithms for de novo short read assembly using de Bruijn graphs**. *Genome research* 2008, **18**(5):821-829.
4. Grabherr MG, Haas BJ, Yassour M, Levin JZ, Thompson DA, Amit I, Adiconis X, Fan L, Raychowdhury R, Zeng Q: **Trinity: reconstructing a full-length transcriptome without a genome from RNA-Seq data**. *Nature biotechnology* 2011, **29**(7):644.
5. Margulies M, Egholm M, Altman WE, Attiya S, Bader JS, Bemben LA, Berka J, Braverman MS, Chen Y-J, Chen Z: **Genome sequencing in microfabricated high-density picolitre reactors**. *Nature* 2005, **437**(7057):376-380.
6. Zerbino DR: **Using the Velvet de novo Assembler for Short-Read Sequencing Technologies**. In: *Current protocols in bioinformatics.* Edited by Baxevanis AD, al e; 2010: UNIT 11.15.

7. Langmead B, Salzberg SL: **Fast gapped-read alignment with Bowtie 2**. *Nature methods* 2012, **9**(4):357-359.
8. Lamesch P, Berardini TZ, Li D, Swarbreck D, Wilks C, Sasidharan R, Muller R, Dreher K, Alexander DL, Garcia-Hernandez M: **The Arabidopsis Information Resource (TAIR): improved gene annotation and new tools**. *Nucleic acids research* 2012, **40**(D1):D1202-D1210.
9. Mundry M, Bornberg-Bauer E, Sammeth M, Feulner PG: **Evaluating characteristics of de novo assembly software on 454 transcriptome data: a simulation approach**. *PloS one* 2012, **7**(2):e31410.
