# Supplementary material for: Transcriptome sequencing and marker development in winged bean (Psophocarpus tetragonolobus; Leguminosae)
Source: Sci Rep. 2016 Jun 30;6:29070. doi: 10.1038/srep29070 (PMC4928180; doi:10.1038/srep29070)
Supplement: Supplementary File 6 [file srep29070-s6.doc]

**Transcriptome sequencing and marker development in winged bean (*Psophocarpus tetragonolobus*; Leguminosae)**

Mohammad Vatanparast^1^, Prateek Shetty^2^, Ratan Chopra^3^, Jeff J. Doyle^4^, N. Sathyanarayana^5*^ and Ashley N. Egan^1*^

^1^US National Herbarium (US), Department of Botany, Smithsonian Institution-NMNH, 10^th^ and Constitution Ave, Washington DC, 20013, USA.

^2^Department of Plant Biology, Michigan State University, 612 Wilson Road, Room 166, East Lansing, MI, 48824, USA.

^3^United States Department of Agriculture, Agriculture Research Service, 3810 4^th^ St., Lubbock, TX, 79415, USA.

^4^Section of Plant Breeding & Genetics, School of Integrative Plant Science, Cornell University, 412 Mann Library, Ithaca, NY, 14853, USA.

^5^Department of Botany, Sikkim University, 5th Mile, Tadong, Gangtok, Sikkim, 737102, India.

*Email Addresses:*

Mohammad Vatanparast: [Vatanparastm@si.edu](mailto:Vatanparastm@si.edu)

Prateek Shetty: [prateekshettys@gmail.com](mailto:prateekshettys@gmail.com)

Ratan Chopra: [Ratan.Chopra@ARS.USDA.GOV](mailto:Ratan.Chopra@ARS.USDA.GOV)

Jeff J. Doyle: [jjd5@cornell.edu](mailto:jjd5@cornell.edu)

N. Sathyanarayana: [nsathyanarayana@cus.ac.in](mailto:nsathyanarayana@cus.ac.in)

Ashley N. Egan: [egana@si.edu](mailto:egana@si.edu)

**Corresponding authors:*

Ashley N. Egan (primary) N. Sathyanarayana (secondary)

**Supplementary file 6: Kunitz trypsin inhibitor alignment.** Alignment of Pfam Kunitz trypsin inhibitors amino acid sequences in addition to those garnered from the CPP34-7 and Chapman transcriptome assemblies. Abbreviations are as listed in Figure 7.

>pv_V7C568_51_224

--------VVDISGNPISP------------------G--LTYYIIPSI-----------RGPRAGG---LKLGQ-S-----G-NS-P---C---ALTVLQ-H-----PFA--NFR-GI--PVKF-TI-PG-----DSSGII-FTG-TEL-EIE---F-V-E---KP--ECS-E---------------------SSKWLVF-MDEA-----IQK--------ACV-GVGGAEGHPG-QQTY-AG---KFHIEKYH---------------PF-A-YKFVFCIT--------G-TPT--C---LD-IGRFY-AH--NGEEG-----KRLSLTE----H-E--AFDLRFME---------

>pv_V7C681_32_205

--------VLDINGKPISP------------------G--LTYYIIPTM-----------RGPGGGG---LKLGQ-T-----G-NS-Q---C---ALTVLQ-D-----PFA--NFR-GI--PVKF-TI-PG-----DSSGII-FTG-TEL-EIE---F-V-E---KP--ECS-E---------------------SSKWLVF-LDEA-----IQK--------ACV-GVGGAEGHPG-QQTY-AG---KFHIQKYY---------------QF-S-YKLVFCIT--------G-TPT--C---LD-IGRFN-AH--NGEEG-----KRLSLTE----H-E--GFNLGFVE---------

>pv_T2DNW0_32_205

--------VLDTNRNPIRP------------------G--LTYSILPAI-----------RGPDGGG---LKLGQ-T-----G-NS-P---C---ALTVLQ-D-----PFT--FFG-SI--PVKF-TI-PG-----DSSGII-FTG-TEL-EIE---F-V-E---KP--ECS-E---------------------SSKWLVF-LDEA-----IQK--------ACV-GVGGAEGHPG-QQTY-AG---KFHIQKYY---------------QF-S-YKLVFCIT--------G-QPT--C---LD-IGRFN-AQ--NGEEG-----KRLSLTE----H-E--AFRLVFAR---------

>pv_V7C4W6_32_204

--------VVDVNGNPIFP------------------G--LTYYILPAI-----------RGPPGGG---LKLGQ-T-----G-NS-Q---C---ALTVLQ-D-----YIE--VFR-GI--PVKF-TI-PG-----VSPGII-FTG-TKL-EIE---F-V-E---KP--ECS-E---------------------SSKWLVF-VDET-----IQK--------ACV-GVGGAEGHPG-QQTF-GG---KFHIEKYQ----------------F-G-YKLVFCIT--------G-SPT--C---LD-IGRFD-AE--NGEDG-----RRLNLTE----H-E--AFDLVFVQ---------

>Gm_B1ACD5_27_199

--------VVDISGNPIFP------------------G--GTYYIMPST-----------WGAAGGG---LKLGR-T-----G-NS-N---C---PVTVLQ-D-----YSE--IFR-GT--PVKF-SI-PG-----ISPGII-FTG-TPL-EIE---F-A-E---KP--YCA-E---------------------SSKWVAF-VDNE-----IQK--------ACV-GIGGPEGHPG-QQTF-SG---TFSIQKYK----------------F-G-YKLVFCIT--------G-SGT--C---LD-IGRFD-AK--NGEGG-----RRLNLTE----H-E--AFDIVFIE---------

>Gm_Q9LLX2_1_156

---------------------------------------------MPST-----------WGAAGGG---LKLGR-T-----G-NS-N---C---PVTVLQ-D-----YSE--IFR-GT--PVKF-SI-PG-----ISPGII-FTG-TPL-EIE---F-A-E---KP--YCA-E---------------------SSKWVAF-VDNE-----IQK--------ACV-GIGGPEGHPG-QQTF-SG---TFSIQKYK----------------F-G-YKLVFCIT--------G-SGT--C---LD-IGRFD-AK--NGEGG-----RRLNLTE----H-E--AFDIVFIE---------

>Gm_C6SVQ6_27_199

--------VVDISGNPIFP------------------G--GTYYIMPST-----------WGAAGGG---LKLGR-T-----G-NS-N---C---PVTVLQ-D-----YSE--IFR-GT--PVKF-SI-PG-----ISPGII-FTG-TPL-EIE---F-A-E---KP--YCA-E---------------------SSKWVAF-VDNE-----IQK--------ACV-GIGGPEGHPG-QQTF-SG---TFSIQKYK----------------F-G-YKLVFCIT--------G-SGT--C---LD-IGRFD-AK--NGEGG-----RRLNLTE----H-E--AFDIVFIE---------

>isotig08968_m_12187

--------VKDINGNPIFP------------------G--GEYFIMPAI-----------WGPPGGG---LTLGQ-T-----G-NS-D---C---PTTVLQ-D-----YRE--IFQ-GI--PVKF-KI-PG-----ISPGII-FTG-TPL-EIE---F-I-R---KP--DCA-E---------------------SSKWLVF-VDNA-----IQK--------ACV-GIGGAEGHPG-QQTF-SG---TFHIEKYK----------------V-G-YKLVFCIA--------G-SPT--C---LD-IGRFD-AK--NDEGG-----RRLNLTE----H-E--AFDIVFIDASEVGI---

>pv_V7C2P7_85_148

-----------------------------------------------------------------------------------------------------------------------------------------------------------------------------------------------------------------------------------------IHHT-WNPI-ST---RFHIENYH----------------F-G-YKLVFCIT--------G-SPT--C---LD-IGRFD-AE--NGEDG-----RRLNLTE----H-E--AFDLVFVK---------

>pv_V7C2N3_57_172

----------------------------------------------------------------------------------------------------------------------------F-TF-LT-----IIMESL-NCS-TEL-EIE---F-V-R---KP--ECS-E---------------------SSKWLVF-VDEA-----IQK--------TCV-GVGGAEGHPG-QQTF-GG---KFRIENYQ----------------F-G-YKLVFCMT--------E-TPT--C---SN-IGTFN-AH--NDEDG-----RRLNLTE----H-E--AFDLVFVK---------

>pv_V7C2M9_33_202

--------VVDADGNPIVS------------------G--LTYYILPAV-----------YGPFGGG---FKLAQ-T-----G-NS-D---C---PLTVLQ-D-----YSE--AFR-GL--PVKF-S----------SSGTI-FTG-NEL-EIE---F-V-D---KP--ECE-E---------------------SSKWLVF-LDEA-----INK--------LCV-GIGGAQGHPT-ELTF-DG---KFHIEEYQ----------------Y-G-YKLAFCIT--------A-PPT--C---SP-IGRFD-AE--NHEDG-----RRLILTE---DD-E--EFYIVFVK---------

>Mt_G7KMU7_31_203

--------VLDINGNPIFP------------------G--GQYYILPAL-----------RGPGGGG---VRLGR-T-----G-DL-K---C---PVTVLQ-D-----RRE--VKN-GL--PVKF-TI-PG-----ISPGII-FTG-TPL-EIE---Y-T-K---KP--SCA-A---------------------STKWLIF-VDNV-----IGK--------ACI-GIGGPENYPG-VQTL-KG---KFNIQKHA--------------SGF-G-YNLGFCVT--------G-SPT--C---LD-IGRFD-----NDEAG-----RRLNLTE----H-E--VYQVVFVD---------

>Mt_Q8LK19_31_203

--------VLDINGNPIFP------------------G--GQYYILPAL-----------RGPGGGG---VRLGR-T-----G-DL-K---C---PVTVLQ-D-----RRE--VKN-GL--PVKF-TI-PG-----ISPGII-FTG-TPL-EIE---Y-T-K---KP--SCA-A---------------------STKWLIF-VDNV-----IGK--------ACI-GIGGPENYPG-VQTL-KG---KFNIQKHA--------------SGF-G-YNLGFCVT--------G-SPT--C---LD-IGRFD-----NDEAG-----RRLNLTE----H-E--VYQVVFVD---------

>Mt_G7KMU3_32_207

--------VLDINGNPIFP------------------G--GKYYILPAI-----------RGPLGGG---LRLGK-S-----S-NS-D---C---EVTVVQ-D-----YNE--VIN-GV--PVKF-SI-PE-----ISPGII-FTG-TPI-DIE---F-T-K---KP--NCV-E---------------------SSKWLIF-VDSV-----IQK--------ACV-GIGGPENYPG-FRTL-SG---TFNIEKHE--------------SGF-G-YRLGYCVK--------D-SPT--C---LD-IGRAH-EEV-EDEGG-----SRLHLTH----Q-V--AFAVVFVD---------

>pv_V7C2P7_32_94

--------IVDTNGNPIFR------------------G--FTYYIFPAI-----------FGPSGGG---LKLAQ-T-----G-NS-K---C---ALTVLQ-D-----YSN--LFR-GI-------------------------------------------------------------------------------------------------------------------HHT-WNPI-ST------------------------------------------------------------------------------------------------------------------

>Mt_G7KMI6_28_201

--------VRDSNGNPIFF------------------S--SRFYVKPSI-----------FGAAGGG---VKLGE-T-----G-NS-S---C---PLTVLQ-D-----YSE--VVN-GL--PVKF-ST-DAE----IFIDLI-STDTSRV-DIV---F-P-E---KP--ECA-E---------------------SSKWLLI-EDD------FPR--------PWV-GIGGIEDYIG-KHII-DG---KFKIVKHG----------------F-G-YKLVFCPT-FTAP-----PGL--C---HD-IGRYD-D-----KNG-----RRLILTE----D-D--PYEVVFEH---------

>Mt_G7KP86_30_147

--------VFDTNGNPIFP------------------G--GTFYIMPSI-----------FGAAGGG---LRLGK-T-----K-NS-K---C---PLTVLQ-D-----YSE--VVN-GL--PVKF-TR-LE-----AGHDII-STN-TAL-DIA---F-T-T---KP--DCA-E---------------------SSKWVLV-DDFN----KLTG--------PWV-GIGGTEDNED------------------------------------------------ITAP----------------------------------------------------------------------

>Mt_G7KP95_27_92

--------VIDTHGKPMFA------------------G--GTFYILPAI-----------FGAAGGG---LRLAK-T-----G-NS-K---C---PFTVLQ-DYFGSIYFK--IVS-LI--FITF-RR-T-------------------------------------------------------------------------------------------------------------------------------------------------------------------------------------------------------------------------------------

>Mt_G7KMG8_25_193

--------VEDINGNPVFP------------------G--GKYYIAPLI-----------SKGGGGG---LKLGK-T-----G-DS-E---C---PVTVIQ-D-----FSE--VVR-GF--PVRF-II-------RVRRGVI-FTT-DEL-DIE---F-V-K---KP--KCA-E---------------------SAKWVLA-HDD------FPT--------SWV-GIGDN------IDAF-QG---KFKIETLG---------S----GSG-A-YKLVYCPL-FSAP-----PGA--C---SD-IGRYR-D-----ENG-----WRLVPTE----N-D--PFRVVFVD---------

>Mt_G7KMH6_26_193

--------VEDINGNPVFP------------------G--GKYYIAPLI-----------SKGGGGG---LKLGK-T-----G-DS-E---C---PVTVLQ-D-----FSE--VVR-GL--PVRF-TI-------IVKRGVI-FTT-DEV-DIE---F-V-K---KP--KCA-E---------------------SAKWVLA-HDD------FPT--------SWV-GIGDN------IDAF-QG---KFKIETLG---------S----GSG-A-YKLVYCPL-FSAP-----PGA--C---SD-IGRYR-D-----ENG-----WRLVPTE----N-D--PFRVVFI----------

>Mt_I3SQY8_29_203

--------VLDINGTPLTP------------------G--GQYYILPES-----------DNPSIGG---LILNK-I-----D-DL-E---C---PVTVVQ-DI---------TVI-GL--PVKF-SM-LE-----NSTSNI-LPG-TDL-EIE---F-T-T---KP--DCA-K---------------------SSKWSMF-VDHD-----TQL--------SFV-GIGGSANNPG-VETT-SG---KFLVVKHQ---------H---GSGH-A-YRIGFCLD--------T-TGD--C---GF-IALEFFN---SEDGG-----PRLIFTV----N-D--AYSVVFVD---------

>Mt_G7KKD7_29_201

--------VLDIHGTPLIP------------------G--SQYYIFPAS-----------ENPNSGG---LTLNK-V-----G-DL-E---C---PVTVLQ-N-------N--AMI-GL--PVKF-TV-PE-----NNTGNI-LTG-TDL-EIE---F-T-K---KP--DCA-E---------------------SSKWLMF-LDHN-----TQL--------SCV-GIGGATNYHG-IETI-SG---KFLIVKHG--------------SGH-V-YRLGFCLD--------V-TGD--C---GY-IGLQMFN---SEEGG-----SRLFLTA----V-D--AYSVVFVD---------

>Mt_I3T969_29_201

--------VFDKHGNPLTP------------------G--NQYYILPAS-----------DNPSSGG---LTLDK-V-----G-DS-V---C---PLTVLQ-N-------N--AVT-GL--PVKF-TI-LE-----NSTSNI-VTG-TDL-EIE---F-I-N---KP--DCA-E---------------------SSKWLMV-VDHV-----TQL--------SFV-GIGGPANYPG-VELI-SG---KFLILKHG--------------SGN-A-YRVGFCLD--------T-TGD--C---AY-LGLHEFN---SGEGG-----SRLILTA----I-N--AYSVVFVD---------

>Mt_B7FN38_29_201

--------VFDKHGNPLTP------------------G--NQYYILPAS-----------DNPSSGG---LTLDK-V-----G-DS-V---C---PLTVLQ-N-------N--AVT-GL--PVKF-TI-LE-----NSTSNI-VTG-TDL-EIE---F-I-N---KP--DCA-E---------------------SSKWLMV-VDHV-----TQL--------SFV-GIGGPANYPG-VELI-SG---KFLILKHG--------------SGN-A-YRVGFCLD--------T-TGD--C---AY-LGLQEFN---SGEGG-----SRLILTA----I-N--AYSVVFVD---------

>Mt_I3SFS0_30_207

--------VLDTNGNPIVP------------------G--GEYYIFPAT-----------QDPYKGG---LRLAK-T-----G-DS-K---C---PVTILQ-N-------E--SIT-GL--PVKF-TI-QG-----ISNDII-MTG-TEL-KIE---F-T-K---KP--NCV-K---------------------SSQWLMF-FDYD-----ASS--------FNV-IIGVDEYIPE-IIII-DG---TFYIQKYG----------------N-A-YKLGFCND------DMG-EQN--C---LD-IMRWN-N---SKDGGSR---LRLILKAEQEVQ-D--PYPVVFVD---------

>pv_V7C4Y1_31_199

--------VRDSNGLPISP------------------F--VQYRLSDLN----------RHGPQGGG---VTSDF-G-----R-NS-V---C---NVAVTS-K-----FD-----L-GV--NLRF-NI-EG-----RSSGVI-LTE-TPL-EIR---F-G-F---IP--YCA-E---------------------SSEWVVV--DD------FPK--------KWI-AIGKNGDHLG-KQIL-SG---TFMFKKYG----------------E-G-YNFAFCSN------NTN-HNT--C---FS-IGRTN-D-----HEG-----RHLVLMD----D-S--HTNVPFNFQ--------

>pv_V7C689_18_139

--------VRDSNGLPISP------------------S--VQYRLSVLN----------RHGPQGGG---VTLDF-G-----R-NS-I---C---NVAVTS-K-----FD-----L-GV--NLRF-NI-EG-----RSSGVI-LTE-TPL-EIR---F-G-F---IP--YCA-E---------------------STEWVVV--DD------FPK--------KWI-AIGKNGDHLG-KQIL-SG---TFMFKKY--------------------------------------------------------------------------------------------------------

>pv_V7C2Q1_31_203

--------VVDSNGLPISP------------------F--VQYDLSQLN----------FQGPQGGG---VELDF-D-----G-NS-R---C---QVVVIQ-N-----YDQ--FFR-GE--GLRF-ST-EG-----RSSGAI-LTE-TPL-EIR---F-D-Y---HP--YCA-S---------------------SSKWVVV-GDD------FPA--------KWV-GIGDGADHAG-KEIL-SG---TFMIKKYG----------------E-G-YKFAFCSN------NTN-HNT--C---FS-IGRID-D-----HKG-----RRLVLMD----D-S--HLNTPFNFV--------

>pv_V7C2Q5_72_185

------------------------------------------------------------------------------------------------------------------------------NT-EG-----RSSGAI-LTE-TPL-EIR---F-D-Y---HP--YCA-S---------------------SSKWVVV-GDD------FPT--------KWV-GIGDGTDHAG-KEIL-SG---TFMIKKYG----------------E-G-YKFVFCSN------NTN-HNT--C---FS-IGRID-D-----HKR-----RRLVLMD----D-S--HLNTPFNFA--------

>pv_V7C583_24_180

-FSRVLEQVLDS---------------------------------------------------NGGG---VEVDF-D-----G-NP-R---C---QVSVIQ-N-----YDK--FFR-GE--GLRF-NT-EG-----RSSGAI-LTE-TSL-EIK---F-D-Y---HP--YCA-S---------------------SSKWVVG-GDD------FPA--------KCV-GIGDGANHAG-KEIL-SG---TFTIKKYG----------------E-G-YKFALCSN------NTN-HNT--C---FS-VGRID-D-----HKR-----RRLILMD----D-S--HPNAPFN----------

>pv_V7C2Q7_31_199

--------VLDSNGLPISP------------------S--NQYILSQLI----------RNGPQGGG---VEVDS-G-----G-NA-R---S---KVVVTK-N-----CD-----R-GE--GLIL-NI-EG-----RSSGVI-LPE-APL-EIK---F-D-F---HL--YCA-E---------------------SSKWVVV-GDD------FPT--------KWV-GIGEGGDHPR-KEIL-SG---TFMIKKYG----------------E-G-YKFAFCNN------NTN-HNT--C---FS-IERID-N-----HEG-----RRLVLMD----D-S--HTNVPFNF---------

>pv_V7BF35_18_203

----------DSHGTNFSHGAYLILILCISIRNAQQCS--NQYTLLHLN----------RNGPQGGG---VEVGS-G-----P-IE-R---C---KVVATKIN-----Y------R-GK--GLIF-NT-KG-----RSSGVI-LTE-TPL-EIR---F-N-F---IP--YCS-E---------------------SSKWVVF-GDD------FPT--------KWV-GIGEGGDHLG-MEYL-SG---SFMIKKYG----------------E-G-YKFAFCSN------NTN-HNT--C---FS-IGRID-D-----HEG-----RDLVLMD----N-S--HTNVPFNFA--------

>pv_V7C2N7_32_201

--------VTDTQGRPISG------------------A--GKYYISQVN----------GGATGGGG---LFLAQ-T-----G-NS-K---C---QVTILQ-D-----YYD--GHR-GL--AVKL-GA-QG-----AGSGGV-FTG-TPL-DVA---L-D-L---KP--SCA-S---------------------SSKWVVV-ADG------FSQ--------KWV-GIGGGSDHPG-AAIV-TG---TLKIEKYN----------------E-G-YKFVFCVS----------ATT--C---SD-IGTLD-D----GETG-----KRLVLTN----N-A--PFKIAFVS---------

>pv_V7C685_31_199

--------VVDSQGRPVSS------------------A--AKYYILPFF-----------SGPTGGG---VAVGK-A-----G-NS-T---C---DATVLQ-T-----TDE--SDR-GQ--AVKF-ST-RG-----TSSDAI-FTG-QPL-DIA---F-A-D---EP--LCA-S---------------------SSKWVVV-SDD------FPE--------AWV-GIGGAEDHPG-KKIM-SG---TFKIEKFD----------------E-G-YKLVFCAT-------TT-TTT--C---YN-IGRHD-D-----VKG-----RRLVLTN------K--PFQVSFNH---------

>pv_V7C4X8_34_211

--------ILDQNGVPVSA------------------S--AEYYVAQLN-----------GGPTGGG---LTTGL-G-----T-NI-T---C---PLIVLQ-V-----YYE--AVR-GQ--KVKF-TT-RGT---TKSDDKI-RTG-TPL-DIE---F-V-D---KP--SCA-S---------------------SSKWVVV-YDSK-----YPG--------QWL-GIGDAADLPG-KKIV-DG---VFKIEKYS--------------FLE-G-YKFMFCPT--------A-RNV--C---LN-FARLE-D-----ANG-----RRLILVN--ETS-SPYILEAGFDK---------

>Mt_G7KJK4_32_205

--------VKDIFGNPVVP------------------S--GSYYIWPD------------YLVSGGE---LRLGE-T-----E-NS-T---C---PFTVLQ-D-----YSN--LGP-GL--PVKF-TP-QNQ---TSGDDPI-TLS-LHI-DIA---F-E-N---KP--DCA-E---------------------SSKWLVV-EAEN----EYPT--------PWL-AIDGTG-----KKVYDDG---WFEIIGYK---------------KT-G-YLIYFCHK-LSPT-----LGE--C---IY-LSRKN-D-----KNG-----MRLVYEM---DG-D--ALAAVFVN---------

>Mt_G7KJK1_32_204

--------VKDIFGNPVVP------------------S--GSYYIWPD------------YLINGGE---LRLGE-T-----E-NS-T---C---PFTVLQ-D-----YSN--LGP-GL--PVKF-TP-QNQ---TSSDDPI-TLM-LPI-EIT---F-E-N---KP--DCA-E---------------------SSKWLVV-EAEN----EYPT--------PWV-TIDGTN-----KNVY-DG---YFMIVGFK---------------KT-G-YLIFFCHKLLSPT-----PGV--C---IY-LSRRN-D-----ENG-----MRLVYEM---DG-D--ALGAVFV----------

>Gm_K7LE45_13_126

----------------------------------------------------------------------LLLGF-T-----T-N---------------------------------L--PLAF-SQ-EKVHD-FQGPGAI-FTE-TPL-DIA---F-V-D---KP--KCA-T---------------------SSKWVAV-VDD------FPS--------KWL-GIGGAQDHPGLKQII-TG---NFNIKKYD----------------L-G-YKLVFCPP-------------------------------------------VLVLVL---------TLRGMMIRTGGV-----

>Mt_G7KMV4_35_209

--------VVDKNGIPLIP------------------G--TSYYISPAN--------------TGGR---ITLGK-T-----V-DS-D---C---SFLVLQ-D-----DEK--MIY-GR--QVKF-SL-SVG---IIPASLI-FTN-TAL-DIE---F-V-Y---KD--SCV-E---------------------SSKWLIF-VDNV----NNNK--------SFV-GIGGPENYPQGTQIL-NG---KFNIKKSG--------------SEN-A-YKFGFCVK----E-----TPS--C---WD-IGRYM-S---IGEEGG----RRLSFNA----T-E--DFEAVFA----------

>Mt_G7KMU9_213_383

--------LRDKNGNPILV------------------S--KKYFIWPA-------------DGSGGG---LRLNE---------TE-Q---C---PLVVQQ-A-----FSE--DVK-SL--PLKF-IP-TE-----NINDFI-FTGYTSL-DIV---F-E-K---KT--KCA-E---------------------SSKWVVV-KGG------FME--------PWI-GIGGGVN--G-KSVI-DG---LFKIETIR--------------SFR-G-YKLVFCPT-ISDP-----TGQ--C---NN-IGRFF-D----NENG-----LRLIMSE----NFK--PFEVVFVD---------

>Mt_I3SQ24_31_201

--------VKDKNGSPILV------------------S--KKYFIWPA-------------DGSGGG---LRLNE---------TE-Q---C---PLVVQQ-A-----FSE--DVK-SL--PLKF-IP-TE-----NINDFI-FTGYTSL-DIV---F-E-K---KT--KCA-E---------------------SSKWVVV-KGG------FME--------PWI-GIGGGVN--G-KSVI-DG---LFKIETIR--------------SFR-G-YKLVFCPT-ISDP-----TGQ--C---NN-IGRFF-D----NENG-----LRLIMSE----NFK--PFEVVFVD---------

>Mt_G7KMU9_10_134

------------------------------------------------------------------------------------------------------------------YS-KL--FLRM-LK-TE-----NINDFI-FTGYTSL-DIV---F-E-K---KT--KCA-E---------------------SSKWVVV-KGG------FME--------PWI-GIGGGVN--G-KSVI-DG---LFKIERIR--------------GFL-R-YKLVFCPT-ISDP-----PGL--C---NN-IGRFF-D----NENG-----LRLIMSE----NFK--PFEVVFVD---------

>Gm_I1MQA3_32_231

--------IWDTGGQQLFA------------------S--YAYYITARH-----------PDLGSGG---LKLIK-T-----G-NS-T---C---PNTILQ-Y-----FPK--YTH-GL--PMYL-LI-SV-----ISNFIV-YEG-TPL-AIF---M-A-K---KP--NCV-ESLVPDPMKGSPDPPSPLNPPNSSRLLVF-VDNS-----IHK--------TCV-GTAGPEAHPG-LLTY-SG---TFHIEILE---------N-VRFGYS-S-YKLVFCFD------GSD-YQN--C---SY-IGTYD-N-----GEGG----RRLILTE----T-N--PFLFSFVH---------

>Gm_C6TB12_32_231

--------IWDTGGQQLFA------------------S--YAYYITARH-----------PDLGSGG---LKLIK-T-----G-NS-T---C---PNTILQ-Y-----FPK--YTH-GL--PMYL-LI-SV-----ISNFIV-YEG-TPL-AIF---M-A-K---KP--NCV-ESLVPDPMKGSPDPPSPLNPPNSSRLLVF-VDNS-----IQK--------TCV-GTGGPEAHPG-LLTY-SG---TFHIEILE---------N-VRFGYS-S-YKLVFCFD------GSD-YQN--C---SY-IGTYD-N-----GEGG----RRLILTE----T-N--PFLFSFVH---------

>Gm_I1MQA2_32_224

--------IMDASAGQMFP------------------S--TPYYLMPRH-----------HELGGGG---IKLIK-T-----G-NS-T---Y---PNTILQ-Y-----FPN--DNF-GL--PIHL-YI-SV-----ISLLLI-FEG-IPL-AIC---M-E-Q---NP--ACV-ISSVSVPIEGSGN-------PNSSRWLVF-VDNS-----IQK--------TCV-GTGGPEAHPG-LLTY-SG---TFHIEILE---------K-DPLRYN-S-YKLVFCFA------GSD-YKN--C---SY-IGTYD-N-----GEGG----RRLILTE----T-N--PFLFSFFH---------

>Gm_C6SWZ5_32_224

--------IMDASAGQMFP------------------S--TPYYLMPRH-----------HELGGGG---IKLIK-T-----G-NS-T---Y---PNTILQ-Y-----FPN--DNF-GL--PIHL-YI-SV-----ISLLLI-FEG-IPL-AIC---M-E-Q---NP--ACV-ISSVSVPIEGSGN-------PNSSRWLVF-VDNS-----IQK--------TCV-GTGGPEAHPG-LLTY-SG---TFHIEILE---------K-DPLRYN-S-YKLVFCFA------GSD-YKN--C---SY-IGTYD-N-----GEGG----RRLILTE----T-N--PFLFSFFH---------

>pv_V7C6J1_31_204

--------VVDTSGQKLRT------------------G--VKYYILPV------------FRGRGGG---LTVSS-S-----G-NN-T---C---PLFVVQ-E-----KPE--VLN-GT--PVTF-TP-YN-----AKSGVI-LTS-TDL-NIK--SY-G-T---TT--SCD-K---------------------PPVWKLL-KV------LTGV--------WFL-STGGVEGNPG-IDTI-VN---WFKIEKAE----------------K-D-YVISFCPS-VC----KC-QTL--C---RE-LGLYV-G-----DDGN----KHLSLSD------KVPSFRVMFKR---------

>Gm_K7LE99_72_243

--------VLDTSGQKLRT------------------G--VKYYILPV------------FRGRGGG---LTVSS-S-----G-NN-T---C---PLFVVQ-E-----KLE--VSK-GT--PVTF-TP-YN-----AESGVI-LTS-TDL-NIK--SY-V-K---ST--TCD-K---------------------PPVWKLL-KV------LTGV--------WFL-STGGVEGNPG-VNTV-VN---WFKIEKAE----------------K-D-YVLSFCPS-F------A-QTL--C---RE-LGLYV-G-----DDGN----KHLSLSD------KVPSFRVIFKR---------

>Gm_C6T3V8_32_203

--------VLDTSGQKLRT------------------G--VKYYILPV------------FRGKGGG---LTVSS-S-----G-NN-T---C---PLFVVQ-E-----KLE--VSK-GT--PVTF-TP-YN-----AKSGVI-LTS-TDL-NIK--SY-G-K---TT--TCD-K---------------------PPVWKLL-KV------LTGV--------WFL-STGGVEGNPG-VNTV-VN---WFKIEKAE----------------K-D-YVLSFCPS-F------A-QTL--C---RE-LGLYV-G-----DDGN----KHLSLSD------KVPSFKVMFKR---------

>Mt_I3RZB7_26_199

--------VLDISGKKVTT------------------G--VKYYILPV------------IRGKGGG---LTVVN-ENNLNGN-NN-T---C---PLYVLQ-E-----KLE--VKN-GQ--AVTF-TP-YN-----AKKGVI-LTS-TDL-NIK--SY-V-T---KT--TCA-Q---------------------SQVWKLN-KV------LSGV--------WFL-ATGSVEGNPG-FDTI-FN---WFKIEKAD----------------K-D-YVFSFCPS-VC----KC-QTL--C---RE-LGLYV-Y-----DHGK----KHLALSD------QVPSFR--------------

>Mt_G7KKP4_26_204

--------VLDISGKKVTT------------------G--VKYYILPV------------IRGKGGG---LTVVN-ENNLNGN-NN-T---C---PLYVLQ-E-----KLE--VKN-GQ--AVTF-TP-YN-----AKKGVI-LTS-TDL-NIK--SY-V-T---KT--TCA-Q---------------------SQVWKLN-KV------LSGV--------WFL-ATGGVEGNPG-FDTI-FN---WFKIEKAD----------------K-D-YVFSFCPS-VC----KC-QTL--C---RE-LGLYV-Y-----DHGK----KHLALSD------QVPSFRVVFKR---------

>Mt_G7KKP1_26_202

--------VLDISGKQLTT------------------G--VKYYILPV------------IRGKGGG---LTVAN-HGE---N-NQ-T---C---PLYVVQ-E-----KLE--VKN-GE--AVTF-TP-YN-----AKQGVI-LTS-TDL-NIK--SF-V-T---KT--KCP-Q---------------------TQVWKLL-KE------LTGV--------WFL-ATGGVEGNPS-MATV-GN---WFKIEKAD----------------K-D-YVLSFCPAEAC----KC-QTL--C---RE-LGLFV-D-----DKGN----KHLALSD------QIPSFRVVFKR---------

>Gm_K7LCR5_32_107

--------VLDTSSHKLRI------------------G--VKYYILSV------------FKGKGGG---LTISS-S-----D-NN-T---C---SFFVRS-L----------KSQ-RH--PVTF-TP-YN-----AKSGVI-LTS-IDL-NIK--SY-------------P-------------------------------------------------------------------------------------------------------------------------------------------------------------------------------------------

>Mt_G7KKN9_1_90

--------------------------------------------------------------------------------------------------------------------------------------------MI-LTS-TDL-NIK--SY-V-T---KT--TCA-Q---------------------SQVWQLS-KV------LSGV--------WFF-STEGVEGNPC-FNTN-KE--YWFKIEKAD----------------N-D-YVLSFCPL-VC----QC-QTL--VIKFHH-LGLCL------------------------------------------------

>Ptri_B9H290_33_207

--------VLDTDGEKLRA------------------G--TEYYILPV------------FRGRGGG---LTMAS-T-----R-DE-T---C---PLDVVQ-D-----PLE--ISK-GL--PLTF-TP-VN-----PKKGVI-RVS-TDL-NIK---F-S-A---SS--ICV-Q---------------------STVWKIQ-KSVN----SEIQ--------WFV-TTGGVEGNPG-IETI-TN---WFKIEKAG----------------D-D-YKLVFCPT-VC----DC-GAL--C---RD-VGIYI-H-----DNGV----RTLSLSD------ALQPFLVNFKK---------

>Ptri_D1KFL1_33_207

--------VLDTDGEKLRA------------------G--TEYYILPV------------FRGRGGG---LTMAS-T-----R-DE-T---C---PLDVVQ-D-----PLE--ISK-GL--PLTF-TP-VN-----PKKGVI-RVS-TDL-NIK---F-S-A---SS--ICV-Q---------------------STVWKIQ-KSVN----SEIQ--------WFV-TTGGVEGNPG-IETI-TN---WFKIEKAG----------------D-D-YKLVFCPT-VC----DC-GAL--C---RD-VGIYI-H-----DNGV----RTLSLSD------ALQPFLVNFKK---------

>Ptri_U5FH02_28_203

--------VLDIDGEKLVA------------------G--TEYYILPV------------FRGRGGG---ITMAS-N-------KT-S---C---PLAVVQ-D-----RLE--VSK-GL--PLTF-TPAAD-----DKKGVI-LVS-TDL-NIK---F-L-A---KT--TCP-Q---------------------STVWKIT-KSSN----SKVQ--------WFV-STGGVEGNPG-FNTV-TN---WFQIEKAD----------------D-D-YKIVFCPTKVC----NC-GVL--C---RD-IGIYI-E-----DNGT----RTLSLSD------ALQPFKVQFKK---------

>Ptri_U5FJJ3_32_205

--------VLDVTGKILRI------------------G--TSYYILPV------------IRGRGGG---LKMAS-T-----V-RR-T---C---PLDVVQ-D-----RYE--ASN-GL--PLKF-TP-VN-----TKKGVV-RVH-TDL-NIR---F-S-A---AS--ICH-Q---------------------STAWKLD-NYDE----WTKQ--------WFV-TTDGVEGNPG-PETT-NN---WFKIEKFE----------------D-K-YKLVFCPT-VC-Q--HC-KVM--C---KD-IGIYV-D-----AKGV----RRLALTN------V--PLKVMFKK---------

>Ptri_U5FHI8_32_205

--------VLDVTGKILRT------------------G--TSYYILPV------------IRGRGGG---LKMAS-T-----V-RR-T---C---PLDVVQ-D-----RYE--ASN-GL--PLKF-TP-VN-----TKKGVV-RVH-TDL-NIR---F-S-A---AS--ICH-Q---------------------STAWKLD-NYDE----WTKQ--------WFV-TTDGVEGNPG-PETT-NN---WFKIEKFE----------------D-K-YKLVFCPT-VC-Q--HC-KVM--C---KD-IGIYV-D-----AKGV----RRLALTN------V--PLKVMFKK---------

>Ptri_U5FKJ0_32_205

--------VLDVTGKILRT------------------G--TSYYILPV------------IRGRGGG---LKMAS-T-----V-RR-T---C---PLDVVQ-D-----RYE--ASN-GL--PLKF-TP-VN-----TKKGVV-RVH-TDL-NIR---F-S-A---GS--ICH-Q---------------------STAWKLD-NYDE----WTKQ--------WFV-TTDGVEGNPG-PETT-NN---WFKIEKFE----------------D-K-YKLVFCPT-VC-Q--HC-KVM--C---KD-IGIYV-D-----AKGV----RRLALTN------V--PLKVMFKK---------

>Ptri_B9H293_32_206

--------VLDIAGKVLRT------------------G--TYYDILPV------------ERGRGGG---ITFAC-T-----G-HK-S---C---PVDVML-E-----DYE--DSD-GL--PLQF-IP-AN-----RKKGVI-RLS-TDL-NIK---F-P-G---PA--SCA-A---------------------TAVWKVE-KYDE----LTSQ--------MFI-STSGVEGNPG-PETV-DN---WFKIEKYG----------------N-D-YKLVFCPT-VC-N-DHC-KVL--C---KD-IGIYV-D-----KEGF----KRLALSD------V--PLKVKFKK---------

>At_Q9LMU2_29_194

--------VKDINGKSLLT------------------G--VNYYILPV------------IRGRGGG---LTMSN-L-----K-TE-T---C---PTSVIQ-D-----QFE--VSQ-GL--PVKF-SP-YD------KSRTI-PVS-TDV-NIK---F-S-P---------------------------------TSIWELA-NFDE----TTKQ--------WFI-STCGVEGNPG-QKTV-DN---WFKIDKFE----------------K-D-YKIRFCPT-VC-N--FC-KVI--C---RD-VGVFV-------QDGK----RRLALSD------V--PLKVMFKR---------

>Ptri_B9H291_30_204

--------VLDIQGEELKA------------------G--TEYIITSA------------IWGAGGG---DVSAT---------NK-T---C---PDDVIQ-Y-----SLD--QLQ-GL--PVTF-SP-AS-----SEDDVI-RVS-TDL-NIK---F-S-I---KK--ACD-H---------------------SSVWKIQ-KSSN----SEVQ--------WFV-TTGGEEGNPG-VHTL-TN---WFKIEKAG----------------TLG-YKLVFCPEDIC----HC-GVL--C---RD-IGIYF-E-----NNRG----RILSLSD------KLSPFVVLFKK---------

>Ptri_D4IH09_1_107

----------------------------------------------SA------------IWGAGGG---DVSAT---------NK-T---C---PDDVIQ-Y-----SSD--QLQ-GL--PVTF-SP-AS-----SEDDVI-RVS-TDL-NIK---F-S-I---KK--ACD-H---------------------SSVWKIQ-KSSN----SEVQ--------WFV-TTGGEEGNPG-VDTL-TN---WFKIEKAG-------------------------------------------------------------------------------------------------------

>Ptri_D2TE95_1_148

----------------------------------------------------------------GGG---DVSAT---------NK-T---C---PDDVIQ-Y-----SSD--QLQ-GL--PVTF-SP-AS-----SEDDVI-RVS-TDL-NIK---F-S-I---KK--ACD-H---------------------SSVWKIQ-KSSN----SEVQ--------WFV-TTGGEEGNPG-VDTL-TN---WFKIEKAG----------------TLG-YKLVFCPEDIC----HC-GVL--C---RD-IGIYF-E-----NNRG----RILSLSD------KLSPFVVP------------

>pv_V7C2X4_1_67

-----------------------------------------------------------------------------------------------PLR---------------------------------------------------------------------------------------------------------------------------------------NNPG-SQTI-SN---WFKIEKYE----------------D-A-YKMVYCPS-VC-N--YC-NYP--C---SD-IGIYQ-D-----QYG-----NPLALTS------E--PYKVQFQR---------

>pv_V7C576_20_168

----------------EAA------------------G--DNYYIVPA--SSD----------V-GG---LSLL--V-----T-GE-N---C---PLDVVA--------VD--GYQ-GK--QLSF-VP-AN-----DKKGVI-CVS-TDL-NIY---F-S-T---CT--SCP-Q---------------------ST--------------------------WFVTTTGGVLGNPG-SQTI-SN---WFKIEKYE----------------D-A-YKMVYCPS-VC-N--YC-NYP--C---SD-IGIYQ-D-----QYG-----NRLALTS------E--PDKVQFQR---------

>pv_V7C582_1_63

--------------------------------------------------SSD----------V-GG---LSLS--I-----T-GE-D---C---PLDVVV--------VD--GYQ-GQ--QLIF-VP-VN-----DKKGVI-RVS-TDF-NIY---F-S-T---CI--SCP-Q---------------------ST------------------------------------------------------------------------------------------------------------------------------------------------------------------

>Gm_I1L3T2_30_198

--------VLDTSGKIVRA------------------R--SSYYIVPA--SPD----------L-GG---LDMA--S-----T-GA-D---C---PLDVVA--------VD--GYQ-GQ--PLIF-TP-VN-----FNKGVI-RVS-TDL-NIY---F-P-V---GT--SCP-Q---------------------TTAWKLK-DYDY----STSQ--------WFV-TTGGDFGNPG-SQTV-AN---WFKIEKYE----------------D-A-YKLVYCPS-VC-N--DC-SYP--C---SD-IGIYQ-D-----EYG-----KRLALSS------E--PYKVKFQR---------

>Gm_C6T5E3_30_198

--------VLDTAGKIVRA------------------R--SSYYIVPA--SPD----------L-GG---LDMA--S-----T-GA-D---C---PLDVVA--------VD--GYQ-GQ--PLIF-TP-VN-----FNKGVI-RVS-TDL-NIY---F-P-V---GT--SCP-Q---------------------TTVWKLK-DYDY----STSQ--------WFV-TTGGDFGNPG-SQTV-AN---WFKIEKYE----------------D-A-YKLVYCPS-VC-N--DC-SYP--C---SD-IGIYQ-D-----EYG-----KRLALSS------E--PYKVKFQR---------

>Gm_K7LE94_1_141

------------------------------------------------------------------------MA--S-----T-GA-D---C---PLDVVA--------VD--GYQ-GQ--PLIF-TP-VN-----FNKGVI-RVS-TDL-NIY---F-P-V---GT--SCP-Q---------------------TTVWKLK-DYDY----SASQ--------WFV-TTGGDFGNPG-SQTM-AN---WFKIEKYE----------------D-A-YKLVYGPS-VC-N--DC-SYP--C---SD-IGIYQ-D-----EYG-----KRLALSS------E--PYKVKFQR---------

>Gm_I1L3T6_30_198

--------VLDTSGKIVRA------------------R--SSYYIVPA--SPD----------L-GG---LDMA--S-----T-GA-D---C---PLDVVA--------ID--GYQ-GQ--PLIF-TP-VN-----FNKGVI-RVS-TDL-NIY---F-P-V---AT--SCP-Q---------------------TTVWKLK-DYDY----STSQ--------WFV-TTGGDFGNPG-SQTM-AN---WFKIEKYE----------------D-A-YKLVYCPS-VC-N--DC-SYP--C---SD-IGIYQ-D-----QYG-----KRLALSS------E--PYKVKFLR---------

>Gm_I1L3T4_30_198

--------VLDTSGKIVRA------------------R--SSYYIVPA--SPD----------L-GG---LDMA--T-----T-GA-D---C---PLDVVV--------VD--GYQ-GQ--PLIF-TP-VN-----FNKGVI-RVS-TDL-NIY---F-P-V---AT--SCP-Q---------------------TTVWKLK-DYDY----STSQ--------RFV-TTGGDFGNPG-SQTV-AN---WFKIEKYE----------------D-A-YKLVYCPS-VC-N--DC-SYP--C---GD-IGIYQ-D-----EYG-----KRLALSS------E--PYKVKFQR---------

>Gm_I1MQG2_30_198

--------VLDTSGKMVRA------------------R--TSYYIVPA--SPD----------V-GG---LAMA--S-----T-GE-D---C---PLDVVA--------VD--GYQ-GQ--PLIF-TP-VN-----VNKGVI-RVS-TDL-NIY---F-P-I---DT--SCP-L---------------------TKAWKLK-DYDY----STSQ--------WFV-TTGGDFGNPG-SQTL-AN---WFKIEKYE----------------D-A-YKLVYCPS-VC-K--DC-SYP--C---SD-IGIYQ-D-----QYG-----KRLALSS------E--PYRVKFQR---------

>Gm_I1MQG3_27_196

--------VLDALGKKVRA------------------D--SIYYIVPA--SSD----------I-GG---LASA--R-----T-DV-D---C---PLDVVA--------VD--GDL-GL--PLSF-TP-VN-----DKKGII-RVS-SDL-NIY---F-T-S--YTI--FCP-Q---------------------TTVWKLK-YYDD----STSQ--------WFV-TTGGELGHPS-SQTV-AN---WFKIEKYE----------------D-A-YKLVYCPS-VC-S--DC-NHQ--C---SD-IGIYQ-D-----QYG-----KRLALSS------E--PYKVQFER---------

>Gm_C6SY46_30_212

--------VIDTEGKKVRA------------------G--VDYYIRPV--PTTPCDGRG-PCVVGSGY--VLIAR-S-----S-NH-T---C---PLSVAV--------VE--GFR-GL--AVTF-KL-VN-----PKKGVI-RVS-TDL-NIK---T-S-L--TNT--SCS-E---------------------STVWKLD-AFDD----STGQ--------WFV-TTGGVLGNPG-KDTI-DN---WFKIEEYD----------------D-D-YKLVFCPT-VC-N--FC-KPL--C---RN-VGVFR-D-----SNGN----QRVALTD------E--PYKVRFQP---------

>Mt_I3SMI6_30_211

--------VVDTEGKKVRA------------------G--VDYYIRPV--PTTPCDGRG-PCVVGSGF--VLIAR-S-----P-NE-T---C---PLNVVV--------VE--GFR-GQ--GVTF-TP-VN-----PKKGVI-RVS-TDL-NIK---T-S-L---NT--SCE-E---------------------STIWTLD-DFDS----STGQ--------WFV-TTGGVLGNPG-KDTV-DN---WFKIEKYE----------------D-D-YKFVFCPT-VC-N--FC-KVM--C---RN-VGIFR-D-----SNGN----QRVALTD------V--PYKVRFQP---------

>Mt_G7KKR0_30_211

--------VVDTEGKKVRA------------------G--VDYYIRPV--PTTPCDGRG-PCVVGSGF--VLIAR-S-----P-NE-T---C---PLNVVV--------VE--GFR-GQ--GVTF-TP-VN-----PKKGVI-RVS-TDL-NIK---T-S-L---NT--SCE-E---------------------STIWTLD-DFDS----STGQ--------WFV-TTGGVLGNPG-KDTV-DN---WFKIEKYE----------------D-D-YKFVFCPT-VC-N--FC-KVM--C---RN-VGIFR-D-----SNGN----QRVALTD------V--PYKVRFQP---------

>Gm_C6T2D3_31_212

--------VIDTSGKKLRA------------------D--ANYHIIPAV-PFTICGFV--SCFTGGG---LSLDS-------I-DE-S---C---PLDVII--------EK--ANE-GL--PLRF-SP-VN-----TKKGVI-RVS-TDL-NIF---F-S-D--SDE--RCP-HH--------------------STVWMLD-QFDA----SIGQ--------TYV-TTGGVVGNPG-EHTI-LN---WFKIQKYE----------------D-A-YKLVYCPR-VC-P--SC-HHL--C---KD-IGMFV-D-----ANRR----MHLALSD------D--PFKIKFKE---------

>Mt_I3S253_30_211

--------VIDTSGKKLRA------------------D--TNYYIIPAK-PFTTCGFV--SCFNSGG---IALET-------V-GE-S---C---PLDVVV--------VK--HNQ-GL--PLRF-TP-VN-----NKKGAV-RVS-TDL-NIK---F-S-NDAYDS--RCP-NH--------------------SLVWKID-PF------SKEE--------TFV-TTNGVLGNPG-FNTI-HN---WFQIEKYE----------------D-A-YKLVYCPN-VC-P--SC-KHV--C---KD-IGIYV-Y-----KYRE----MRLALTN------V--PFKVKFQK---------

>Mt_G7KKQ9_30_211

--------VIDTSGKKLRA------------------D--TNYYIIPAK-PFTTCGFV--SCFNSGG---IALET-------V-GE-S---C---PLDVVV--------VK--HNQ-GL--PLRF-TP-VN-----NKKGVV-RVS-TDL-NIK---F-S-NDAYDS--RCP-NH--------------------SLVWKID-PF------SKEE--------TFV-TTNGVLGNPG-SNTI-HN---WFQIEKYE----------------D-A-YKLVYCPN-VC-P--SC-KHV--C---KD-IGIYV-Y-----KYRE----MRLALTN------V--PFKVKFQK---------

>Mt_G7KKQ7_31_198

--------VLDISGKKLRT------------------D--SDYYIIPA---------------NGGD---ISLES-S-----I-GE-S---C---PLHVVV--------VK--HRQ-GLGFPLRL-AP-V--------KGDI-RVS-TDL-NIM---L-G-N--YDD--RCP-NY--------------------SVVWKID-PY------SKEA--------TFV-TTNGILGHPG-SNSI-HS---WFKIEKYE----------------D-A-YKLVYCPN-VC-P--SC-NHV--C---KD-IGIYK-Y-----KNRE----MRLALTN------V--PLKIKFQQ---------

>Gm_C6TLR5_31_208

--------VVDTLGKKLRV------------------G--TNYYIVPSL-PYT-------KIRTTRG---LGLAS-------V-GK-P--YC---PLDVVV--------VN--GYH-GL--PVTF-SP-VN-----PKKGVI-RVS-TDL-NIK---F-S-A---RT--SCPRQY--------------------STVWKLD-DFDF----SKRQ--------WFV-TTGGVVGNPS-LETI-HN---WFKIEKYD----------------G-A-YKLVYCPS-VV-K--CP-KHL--C---KN-VGLFV-D-----EKGN----KRLALTD------V--PLKVQFQQ---------

>Mt_Q6ISX8_31_212

--------VVDTLGKKLRA------------------D--ANYYIIPV--PIYKCGPYGKCRSSGSS---LALAS-------I-GK-T---C---PLDVVV--------VD--RYQ-AL--PLTF-IP-VN-----PKKGVI-RVS-TDL-NIK---F-S-S---RA--TCL-HH--------------------SMVWKLD-RFNV----SKRQ--------WFI-TIGGVAGNPG-WETI-NN---WFKIEKYG----------------D-A-YKLVFCPS-VV-Q--SF-KHM--C---KD-VGVFV-D-----ENGN----KRLALSD------V--PLKVKFQQ---------

>Mt_G7KKP6_31_212

--------VVDTLGKKLRA------------------D--ANYYIIPV--PIYKCGPYGKCRSSGSS---LALAS-------N-GK-T---C---PLDVVV--------VD--RYQ-AL--PLTF-IP-VN-----PKKGVI-RVS-TDL-NIK---F-S-S---RA--TCL-HH--------------------SMVWKLD-RFNV----SKRQ--------WFI-TIGGVAGNPG-WETI-NN---WFKIEKYG----------------D-A-YKLVFCPS-VV-Q--SF-KHM--C---KD-VGVFV-D-----ENGN----KRLALSD------V--PLKVKFQQ---------

>Gm_I1L3U2_70_254

--------VIDTSGTELQP------------------G--LSYYVVPAMRSFTRCGKF--ECLNAEG---LSLAS-------I-GE-S---C---PLDVVV--------EQ--RSF-GL--PLSF-SP-LD-----TNESVV-RVS-TDL-NIM---F-C-T--DRTSYSCA-EY--------------------SPVWKLD-HFDV----SKGK--------WFV-TTGGSMGNPS-WETI-RN---WFKIEKCD----------------S-A-YRIVYCPS-VC-P--SS-KHM--C---KD-VGVFV-D-----ENGY----RRLALSD------V--PFKVKFQL---------

>Gm_I1L3U0_30_214

--------VVDTSGKKLRA------------------G--LSYYIVPAV-PLTRCGRYE-RCMGGGG---LSLAS-------I-GE-S---C---PLDVVV--------VP--RSH-GL--PLQF-SP-VD-----PKKGVV-RVS-TDL-NIM---F-S-T--DHT--SCA-EY--------------------SPVWKLD-HFDV----SKGK--------WFV-STGGSMGNPS-WETI-RN---WFKIEKCD----------------G-A-YKIVYCPS-VF-PSSSS-KHM--C---KD-IGVFV-D-----ENGF----RRLALSN------V--PFKVKFQR---------

>Gm_K7LE98_2_98

-------------------------------------------------QTITKCGKYE--CLNAEG---LPLAS-------I-GE-S---C---PLDVVV--------VQ--RSF-GL--PLSF-SP-VN-----PDEGVVIPMS-TDL-NFI---F-S-I--GRT--ICA-EY--------------------SPVWKLD-HFH-------------------------AIGYPG-WKTI-HN---W--------------------------------------------------------------------------------------------------------------

>Gm_I1L3T9_32_210

--------VLDTSGKVLRE------------------G--VNYNILISM-PYT-------SCRSPQG---LGLSK-------I-GN-S---C---PLDVVV--------VD--INH-RL--PLRF-IP-VN-----PKKGVI-RVA-TDL-NIM---F-P-D--RNV--TCP-HH--------------------STVWKVD-NFHV----SKGH--------RLV-TTGGVVGYPG-RETI-GN---WFKIEKYD--------------GAY-N-YKLVYCPS-VC-P--SC-KHE--C---KN-VGMVV-D-----QNGN----QRLALSD------V--PYQFRFFK---------

>Gm_K7K398_1_89

-----------------------------------------------------------------------------------------------------------------------------------------------------------------------------------------------------MWKND-NFHV----SKGH--------RLV-TTGGVVGNLG-KETV-GN---WFMIEKTD--------------GAY-N-YKIVYCLS-EC-L--SC-KRK--F---KN-VGMVV-D-----QNGN----QHLALSD------V--PFQFRFLK---------

>Mt_G7KKQ6_27_203

--------VVDITGKNLRA------------------N--AYYNVLLSM-PYT-------NSRSPEG---LGLSN-N-----I-GQ-P---C---PLDVIV--------VS--RYQ-SL--PIRF-TP-LN-----LKKGVI-RVS-SDL-NIM---F-R-S---NS--SCP-YH--------------------TTVWKLD-RFDA----SKGK--------SFV-TTDGFIGNPG-PQSI-SN---WFKIEKYV----------------E-G-YKLVYCPI-VC-P--SC-KHE--C---KN-VGLFE-D-----ENGN----KRLALSD------V--PYQVKFVK---------

>Gm_K7LE97_30_214

--------VVDTSGNILRV------------------G--ANYYIIPN--PTTKCSIFS-KYKGNNG---LVLAKVA-----A-NK-T---F---PLDVLV--------VE--GQQLGQ--PLTF-TP-IHD---QKKGAPV-RVS-TDL-NIE---F-S-M---QT--SCS-Q---------------------SNVWKID-HFDR----ATRK--------WFV-TTGGVVGHPS-WRTI-SN---WFKIEKYD----------------G-D-YKLVSCPT-FC-A--YC-KVQ--C---RD-IGVYE-D-----QNGN----KRLALTD------A--PYKVRFQK---------

>Mt_G7KRA4_29_201

--------VRDTSGNLVRN------------------S--INYFILPS------------SIQCGTR---CEMALLN-----T-NK-T---C---PLDVVE------------EEE-AM--QFSF-VP-FN-----FKKGVI-RVS-TDL-NVI---H-S-F---PT--NCS-TSS-------------------VTVWKVD-KVDV----ATSQ--------RFV-TTGGVQGNPG-RETV-DN---WFKIERFE----------------S-G-YKLVFCPT-VC-R--EC-EVV--C---KD-IGIFL-D-----ENRN----TRFVLSD------F--PFGVKFQR---------

>At_Q93Y29_30_204

--------VVDIDGNAMFH---------------------ESYYVLPV------------IRGRGGG---LTLAG-R-----G-GQ-P---C---PYDIVQ-E-----SSE--VDE-GI--PVKF-SN-WR-----LKVAFV-PES-QNL-NIE---T-D-V--GAT--ICI-Q---------------------STYWRVG-EFDH----ERRQ--------YFV-VAGPKPEGFG-QDSL-KS---FFKIEKSG---------------ED-A-YKFVFCPR-TC-D--SG-NPK--C---SD-VGIFI-D-----ELGV----RRLALSD------K--PFLGYVQK---------

>At_Q9CAT9_23_197

--------VVDIDGNAMFH---------------------ESYYVLPV------------IRGRGGG---LTLAG-R-----G-GQ-P---C---PYDIVQ-E-----SSE--VDE-GI--PVKF-SN-WR-----LKVAFV-PES-QNL-NIE---T-D-V--GAT--ICI-Q---------------------STYWRVG-EFDH----ERKQ--------YFV-VAGPKPEGFG-QDSL-KS---FFKIEKSG---------------ED-A-YKFVFCPR-TC-D--SG-NPK--C---SD-VGIFI-D-----ELGV----RRLALSD------K--PFLVMFKK---------

>At_Q8RXD5_30_204

--------VVDIDGNAMFH---------------------ESYYVLPV------------IRGRGGG---LTLAG-R-----G-GQ-P---C---PYDIVQ-E-----SSE--VDE-GI--PVKF-SN-WR-----LKVAFV-PES-QNL-NIE---T-D-V--GAT--ICI-Q---------------------STYWRVG-EFDH----ERKQ--------YFV-VAGPKPEGFG-QDSL-KS---FFKIEKSG---------------ED-A-YKFVFCPR-TC-D--SG-NPK--C---SD-VGIFI-D-----ELGV----RRLALSD------K--PFLVMFKK---------

>At_Q8H190_30_204

--------VVDIDGNAMFH---------------------ESYYVLPV------------IRGRGGG---LTLAG-R-----G-GQ-P---C---PYDIVQ-E-----SSE--VDE-GI--PVKF-SN-WR-----LKVAFV-PES-QNL-NIE---T-D-V--GAT--ICI-Q---------------------STYWRVG-EFDH----ERRQ--------YFV-VAGPKPEGFG-QDSL-KS---FFKIEKSG---------------ED-A-YKFVFCPR-TC-D--SG-NPK--C---SD-VGIFI-D-----ELGV----RRLALSD------K--PFLVMFKK---------

>At_Q39091_28_191

--------VEDSVGRLLRP------------------G--QTYHIVPA------------NPETGGG-----IFS-N-----S-EE-I---C---PLDIFQ-S-----NNP--LDL-GL--PIKF-KS---------ELWFV-KEM-NSI-TIE---F-E-A---PNWFLCP-KE--------------------SKGWRVV-YSEE----FKKS--------LII-STGGSSN---------PS---GFQIHRVD---------------GG-A-YKIVYCTN-------IS-TTT--C---MN-VGIFT-D-----ISGA----RRLALTS----D-E--ALLVKFQK---------

>At_Q9FX28_32_202

--------VLDIAGHPVQS------------------N--VQYYIIPA------------KIGTGGG---LIPSN-------R-NLSTQDLCL--NLDIVQ-S-----SSP--FVS-GL--PVTF-SP-LN-----TKVKHV-QLS-ASL-NLE---F-D-S---TVW-LCP-D---------------------SKVWRID-HSVQ-----LRK--------SFV-SIGGQKGKG-------NS---WFQIQEDG----------------D-A-YKLMYCPI-------SS-IVA--C---IN-VSLEI-D-----DHGV----RRLVLST----D-Q--SFVVKFQK---------

>Gm_I1JLM5_74_131

----------------------------------------------------------------------------------T-DE-------------------------------------------------------------------------------------------------------------------------------------------------------------MN---IMRLQKYI--------------F---N-RKLVFCAD-------AN-TTS--C---ED-VGVYV-D-----GEQN----RRLVLS----EV-G--GVAVKFMN---------

>pv_V7B6G3_54_177

-------------------------------------------------------------------------------------------C----------SD----TSQ--IFR-GL--PVRI-SS-PY------GIAYI-NEG-LIL-NLA---F-A-S---SP--SCA-PT--------------------PSKWSVV-KDLP------EG--------EAV-KLPEY------PSTV-SG---WFKIVPSS--------------LKY-L-YKVVFCASS---------GGT--C---GE-VGISV-D-----DEGM----RRLVVS----ED-E--GIMIRFTK---------

>Gm_I1N2Q3_34_203

--------VTDRDGAAIRN------------------G--GTYHILPL------------FGVKNGG---LELAA-T-----R-NE-T---C---PLTVVQ-SR----TAQ--IFR-GL--PVRI-SS-PY------RIAYI-SEG-LIL-NLA---F-A-S---SP--SCA-PT--------------------PPNWTVV-KGLP------EG--------QGV-KLLGYG-----RSTV-SG---WFKIEKSS--------------LEY-L-YKLVFCARA---------SKA--C---GE-IGISV-D-----DEGI----NRLVLTE--EEG-D--GIIVEFMK---------

>Gm_C6T586_28_198

--------VFDTEGNPIRN------------------G--GTYYVLPV------------IRGKGGG---IEFAK-T-----E-TE-T---C---PLTVVQ-S-----PFE--VSK-GL--PLII-SS-PF------KILDI-TEG-LIL-SLS---F-T-Y---VP--PCA-ST--------------------PSRWTVILKGLP------EE--------LHV-KLTGY------KNTI-DG---WFRIQRAS---------S----ESN-Y-YKLVFCTS-------ND-DSS--C---GD-IVAPI-D-----REGN----RPLIVTH--DQN-H--PLLVQFQK---------

>Gm_Q9XIS8_27_197

--------VFDTEGNPIRN------------------G--GTYYVLPV------------IRGKGGG---IEFAK-T-----E-TE-T---C---PLTVVQ-S-----PFE--VSK-GL--PLII-SS-PF------KILDI-TEG-LIL-SLS---F-T-Y---VP--PCA-ST--------------------PSRWTVILKGLP------EE--------LHV-KLTGY------KNTI-DG---WFRIQRAS---------S----ESN-Y-YKLVFCTS-------ND-DSS--C---GD-IVAPI-D-----REGN----RPLIVTH--DQN-H--PLLVQFQK---------

>Gm_B1ACD0_28_198

--------VFDTEGXSIRN------------------G--GTYYVLPV------------IRGKGGG---IEFAK-T-----E-TE-T---C---PLTVVQ-S-----PFE--VSK-GL--PLII-SS-PF------KILDI-TEG-LIL-SLS---F-T-Y---VP--PCA-ST--------------------PSRWTVILKGLP------EE--------LHV-KLTGY------KNTI-DG---WFRIQRAS---------S----ESN-Y-YKLVFCTS-------ND-DSS--C---GD-IVAPI-D-----REGN----RPLIVTH--DQN-H--PLLVQFQK---------

>Gm_Q39869_28_199

--------VFDTEGNPIRN------------------G--GTYYVLPV------------IRGKGGG---IEFAK-T-----E-TE-T---C---PLTVVQ-S-----PFEG-LQR-GL--PLII-SS-PF------KILDI-TEG-LIL-SLK---F-H-L---CT--PLS-LN--------------------SFSVDRYSQGSA------RR--------TPC-QTHWL------QKHN-RC---WFRIQRAS---------S----ESN-Y-YKLVFCTS-------ND-DSS--C---GD-IVAPI-D-----REGN----RPLIVTH--DQN-H--PLLVQFQK---------

>Gm_I1KYW5_27_194

--------IFDINGDFVRN------------------G--GTYYILPV------------IRGDGGG---IEFAA-T-----G-NE-T---C---PLTVVQ-S-----PLE--VSK-GL--PLII-SS-PF------EILSI-QEG-LIL-NIG---F-T-F---VP--PCA-LI--------------------PSEWTTV-KGLP------EG--------LAV-KLTGY------ENKV-PG---WFKIERVS---------L----EFN-D-YKLVFCA--------TE-DST--C---VD-IGVYI-D-----GEGN----RRLVVTE---NN-D--PLLVHFKK---------

>Gm_C6T280_27_194

--------IFDINGDFVRN------------------G--GTYYILPV------------IRGDGGG---IEFAA-T-----G-NE-T---C---PLTVVQ-S-----PLE--VSK-GL--PLII-SS-PF------EILSI-QEG-LIL-NIG---F-T-F---VP--PCA-LI--------------------PSEWTTV-KGLP------EG--------LAV-KLTGY------ENKV-PG---WFKIERVS---------L----EFN-D-YKLVFCA--------TE-DST--C---VD-IGVYI-D-----GEGN----RRLVVTE---NN-D--PLLVHFRK---------

>Gm_I1KYX2_26_190

--------VVDTDGDILQN------------------P--GTYFILSV------------FR-PGGG---VEFAA-T-----G-NE-T---C---PLTVVQ-T-----LF----GR-GF--PAIL-SS-RL------RIPFI-GEG-QLF-SIL---F-R-I---VP--WCA-TT--------------------PSKWTIV-EGLP------ES--------PAV-KLTGY------DNTV-PG---EFKIEKAN---------P----FHN-D-YTLLFCPA-------GE-ESK--C---GH-IGIHF-D-----DDGN----RRLVVS----EE-N--ILRVQFQK---------

>Gm_I1KYX0_26_190

--------VVDTNGDILQN------------------P--GTYFILSV------------FR-PGGG---VEFAA-T-----G-NE-T---C---PLTVVQ-T-----LF----GR-GF--PVIL-SS-QL------RIPII-GEG-QLF-SIL---F-R-I---VP--WCA-TT--------------------PSKWTIV-EGLP------ES--------PAV-KLTGY------DNTV-PG---EFKIEKAN---------P----FHN-D-YTLLFCPA-------GE-ESK--C---GH-IGIHF-D-----DDGN----RRLVVS----EE-N--ILRVQFQK---------

>Gm_Q94IA1_28_200

--------VLDNEGNPLEN------------------G--GTYYILSD------------IT-AFGG---IRAAP-T-----G-NE-R---C---PLTVVQ-S-----RNE--LDK-GI--GTII-SS-PY------RIRFI-AEG-HPL-SLKFDSF-A-V---IM--LCV-GI--------------------PTEWSVV-EDLP------EG--------PAV-KIGEN------KDAM-DG---WFKLERVS---------D---DEFN-N-YKLVFCPQQ------AE-DDK--C---GD-IGISI-D----HDDGT----RRLVVS----KN-K--PLVVQFQK---------

>Gm_Q39898_28_200

--------VLDNEGNPLEN------------------G--GTYYILSD------------IT-AFGG---IRAAP-T-----G-NE-R---C---PLTVVQ-S-----RNE--LDK-GI--GTII-SS-PY------RIRFI-AEG-HPL-SLKFDSF-A-V---IM--LCV-GI--------------------PTEWSVV-EDLP------EG--------PAV-KIGEN------KDAM-DG---WFRLERVS---------D---DEFN-N-YKLVFCPQQ------AE-DDK--C---GD-IGISI-D----HDDGT----RRLVVS----KN-K--PLVVQFQK---------

>Gm_P01070_27_199

--------VLDNEGNPLEN------------------G--GTYYILSD------------IT-AFGG---IRAAP-T-----G-NE-R---C---PLTVVQ-S-----RNE--LDK-GI--GTII-SS-PY------RIRFI-AEG-HPL-SLKFDSF-A-V---IM--LCV-GI--------------------PTEWSVV-EDLP------EG--------PAV-KIGEN------KDAM-DG---WFRLERVS---------D---DEFN-N-YKLVFCPQQ------AE-DDK--C---GD-IGISI-D----HDDGT----RRLVVS----KN-K--PLVVQFQK---------

>Gm_C6T488_28_200

--------VLDNEGNPLEN------------------G--GTYYILSD------------IT-AFGG---IRAAP-T-----G-NE-R---C---PLTVVQ-S-----RNE--LDK-GI--GTII-SS-PY------RIRFI-AEG-HPL-SLKFDSF-A-V---IM--LCV-GI--------------------PTEWSVV-KDLP------EG--------PAV-KIGEN------KDAM-DG---WFRLERVS---------D---DEFN-N-YKLVFCPQQ------AE-DDK--C---GD-IGISI-D----HDDGT----RRLVVS----KN-K--PLVVQFQK---------

>Gm_Q76B18_28_200

--------VLDNEGNPLEN------------------G--GTYYILSD------------IT-AFGG---IRAAP-T-----G-NE-R---C---PLTVVQ-S-----RNE--LDK-GI--ETII-SS-PY------RIRFI-AEG-HPL-SLKFDSF-A-V---IM--LCV-GI--------------------PTEWSVV-EDLP------EG--------PAV-KIGEN------KDAM-DG---WFRLERVS---------D---DEFN-N-YKLVFCPQQ------AE-DDK--C---GD-IGISI-D----HDDGT----RRLVVS----KN-K--PLVVQFQK---------

>Gm_Q9ATY0_28_145

--------VLDNEGNPLEN------------------G--GTYYILSD------------IT-AFGG---IRATP-T-----E-NE-R---C---PLTAVQ-S-----RNE--LDK-GI--GTII-SS-PY------RIRFI-AEG-HPL-SLKFDSF-A-V---IM--LCV-GI--------------------PTEWSVV-EDLP------EG--------PAV-KIGEN------KDAM-DG---WFRL-----------------------------------------------------------------------------------------------------------

>Gm_Q9LD16_27_199

--------VLDNEGNPLEN------------------G--GTYYILSD------------IT-AFGG---IRAAP-T-----G-NE-R---Y---PLTVVQ-S-----RNE--LDK-GI--GTII-SS-PY------RIRFI-AEG-HPL-SLKFDSF-A-V---IM--LCV-GI--------------------PTEWSVV-EDLP------EG--------PAV-KIGEN------KDAM-DG---WFRLERVS---------D---DEFN-N-YKLVFCPQQ------AE-DDK--C---GD-IGISI-D----HDDGT----WRLVVS----KN-K--PLAVQFQK---------

>Gm_P01071_3_175

--------VLDNEGNPLSN------------------G--GTYYILSD------------IT-AFGG---IRAAP-T-----G-NE-R---C---PLTVVQ-S-----RNE--LDK-GI--GTII-SS-PF------RIRFI-AEG-NPL-RLKFDSF-A-V---IM--LCV-GI--------------------PTEWSVV-EDLP------EG--------PAV-KIGEN------KDAV-DG---WFRIERVS---------D---DEFN-N-YKLVFCTQQ------AE-DDK--C---GD-IGISI-D----HDDGT----RRLVVS----KN-K--PLVVQFQK---------

>Gm_Q39899_28_200

--------VLDNEGNPLDS------------------G--GTYYILSD------------IT-AFGG---IRAAP-T-----G-NE-R---C---PLTVVQ-S-----RNE--LDK-GI--GTII-SS-PF------RIRFI-AEG-NPL-RLKFDSF-A-V---IM--LCV-GI--------------------PTEWSVV-EDLP------EG--------PAV-KIGEN------KDAV-DG---WFRIERVS---------D---DEFN-N-YKLVFCTQQ------AE-DDK--C---GD-IGISI-D----HDDGT----RRLVVS----KN-K--PLVVQFQK---------

>Gm_Q9ATY1_28_199

--------VLDNEGNPLEN------------------G--GTYYILSD------------IT-AFGG---IRAAP-T-----G-NE-R---C---PLTVVQ-S-----RNE--LDK-GI--GTII-SS-PY------RIRFI-AEG-HPL-SLKFDSF-A-V---IM--LC--GI--------------------PTEWSVV-EDLP------EE--------HAV-KIGEN------KDAM-DG---WFRLERVS---------D---DEFN-N-YKLVFCPQQ------AE-DDK--C---GD-IGISI-D----HDDGT----RRLVVF----KN-K--PLVVQFQK---------

>Gm_P25272_28_197

--------VLDTDDDPLQN------------------G--GTYYMLPV------------MRGKGGG---IEVDS-T-----G-KE-I---C---PLTVVQ-S-----PNE--LDK-GI--GLVF-TS-PL------HALFI-AER-YPL-SIKFGSF-A-V---IT--LCA-GM--------------------PTEWAIV-ER--------EGL-------QAV-KLAA-------RDTV-DG---WFNIERVS---------R----EYN-D-YKLVFCPQQ------AE-DNK--C---ED-IGIQI-D-----DDGI----RRLVLS----KN-K--PLVVQFQK---------

>Gm_C6SWW4_28_197

--------VLDTDDDPLQN------------------G--GTYYMLPV------------MRGKGGG---IEVDS-T-----G-KE-I---C---PLTVVQ-S-----PNE--LDK-GI--GLVF-TS-PL------HALFI-AEG-YPL-SIKFGSF-A-V---IT--LCA-GM--------------------PTEWAIV-ER--------EGL-------QAV-KLAA-------RDTV-DG---WFNIERVS---------R----EYN-D-YKLVFCPQQ------AE-DNK--C---ED-IGIQI-D-----DDGI----RRLVLS----KN-K--PLVVQFQK---------

>Gm_P25273_28_198

--------VLDTDDDPLQN------------------G--GTYYMLPV------------MRGKSGG---IEGNS-T-----G-KE-I---C---PLTVVQ-S-----PNK--HNK-GI--GLVF-KS-PL------HALFI-AER-YPL-SIKFDSF-A-V---IP--LCG-VM--------------------PTKWAIV-ER--------EGL-------QAV-TLAA-------RDTV-DG---WFNIERVS---------R----EYN-DYYKLVFCPQE------AE-DNK--C---ED-IGIQI-D-----NDGI----RRLVLS----KN-K--PLVVEFQK---------

>Gm_C6T0C7_28_196

--------VVDMEGNDLQN------------------G--GKYYVLPV------------IESSYGG---IRVAA-T-----G-KE-R---C---PLTVVQ-S-----ADP--YDK-GI--ATII-ST-PY------RVPVI-REG-FPL-NITFGDF-A-V---IL--PCV-PL--------------------RSEWTVV-NGQP------EG--------PAV-KIGSP------PNAE-NG---WFEIEKLL---------T------S-G-YKLVFCTR-------PE-RSY--C---QD-IGIHV-D-----DENH----ARLVLT----ND-D--PLVVEFLK---------

>Gm_I1KYX1_28_196

--------VVDMEGNDLQN------------------G--GKYYVLPV------------IESSYGG---IRVAA-T-----G-KE-R---C---PLTVVQ-S-----ADP--YDK-GI--ATII-ST-PY------RVPVI-REG-FPL-NITFGNF-A-V---IL--PCV-PL--------------------RSEWTVV-NGQP------EG--------PAV-KIGSP------PNAE-NG---WFEIEKLL---------T------S-G-YKLVFCTR-------PE-RSY--C---QD-IGIHV-D-----DENH----ARLVLT----ND-D--PLVVEFLK---------

>Gm_I1KYW9_28_198

--------VLDMDGNPLGN------------------GWQNEYFMLPV------------TRGSGGG---IALAA-T-----G-NE-R---C---PVTVVQ-S-----HIE--RDK-GY--AATF-RA-PF------HLPFI-AEG-LPL-TISFDNF-E-V---LP--RCV-PT--------------------PLWWAIV-DGLA------EG--------PAV-KIE-Y------RDIV-EG---WFKIQKAY---------P------L-G-YKLLFCPVP------LE-DST--C---GD-IGIYT-D-----DKGF----RRLVVT----KN-K--PLVVQFQK---------

>Gm_C6T696_28_184

--------VLDMDGNPLGN------------------GWQNEYFMLPV------------TRGSGGG---IALAA-T-----G-NE-R---C---PVTVVQ-S-----HIE--RDK-GY--AATF-RA-PF------HLPFI-AEG-LPL-TISFDNF-E-V---LP--RCV-PT--------------------PLWWAIV-DGLA------EG--------PAV-KIE-Y------RDIV-EG---WFKIQKAY---------P------L-G-YKLLFCPVP------LE-DST--C---GD-IGIYT-D-----DKGF----SVW------------------------------

>Gm_C6T3I9_28_196

--------VLDTDGGVLQN------------------G--GQYSVLPV------------MRGSGGG---LVVRA-T-----G-NE-R---C---PLTVAQ-T-----RNE--LDK-GI--GTII-SS-PL------RVAVI-AEG-HPL-SISFGFF-P-V---MP--SCI-PL--------------------TGDWGIV-DGLP------EG--------PAV-KLAEY------KNIV-DG---WFKIEKAH---------P------L-G-YKLLFCPL-------LE-GST--C---GD-IGIQT-D-----DDGI----RRLVVT----KN-N--PLLVQFQK---------

>Gm_I1KYW8_28_205

--------VLDSDGNPLEN------------------G--GTYYV-PA-------------PNNCGG---IEYTT-S-----G-NE-T---C---PITVVQ-H-----HDP--TCK-GF--PITI-SS-PA------RIRYI-SEG-LNV-NIG---F-T-F--RPP--PCA-P---------------------SSLWTVL-KDQS------EHPDPYEGVILPV-KLNNEDNS---NNTV-PG---WFKIQKLP---------L---DFIT-T-YYIVFCP--------LD-QSP--C---WS-VGSNF-D-----QYGN----RRLVVAQ--FAR----RQDIEFQK---------

>Gm_C6T1F1_28_205

--------VLDSDGNPLEN------------------G--GTYYV-PA-------------PNNCGG---IEYTT-S-----G-NE-T---C---PITVVQ-H-----HDP--TCK-GF--PITI-SS-PA------RIRYI-SEG-LNV-NIG---F-T-F--RPP--PCA-P---------------------SSLWTVL-KDQS------EHPDPYEGVILPV-KLNNEDNN---NNTV-PG---WFKIQKLP---------L---DFIT-T-YYIVFCP--------LD-QSP--C---WS-VGSNF-D-----QYGN----RHLVVAQ--FAR----RPDIEFQK---------

>Gm_C6T261_26_194

--------VVDTDGNPVEN------------------G--GTYFVLPT------------IILNGGG---IEYAT-F-----G-NE-T---C---PVTVAQ-S-----RDQ--FCK-GF--PITI-SS-PA------RIRHI-SEG-LSL-NIG---F-T-F---AS--PCS-P---------------------ASEWTIV-KDQP------EG--------LAV-KLTGF------KNTV-PG---VFTLKRVP---------A---DEII-G-YNILFCP--------LD-NNP--C---GY-VAVHF-D-----QFRN----RRLVVSE--VQE-D--GLWVMFQK---------

>Gm_I1J6C0_47_215

--------VVDTDGNPVEN------------------G--GTYFVLPT------------IILNGGG---IEYAT-F-----G-NE-T---C---PVTVAQ-S-----RDQ--FCK-GF--PITI-SS-PA------RIRHI-SEG-LSL-NIG---F-T-F---AS--PCS-P---------------------ASEWTIV-KDQP------EG--------LAV-KLTGF------KNTV-PG---VFTLKRVP---------A---DEII-G-YNILFCP--------LD-NNP--C---GY-VAVHF-D-----QFRN----RRLVVSE--VQE-D--GLWVMFQK---------

>isotig09349_m_12644

--------VLDTDGNVLLN------------------S--GTYQILQA------------IILNGGG---LEYAA-T-----G-NE-T---C---PLTVVS-S-----PFH--TNH-GF--PVVI-SS-PL------LFTHI-SEG-FPL-YLR---F-T-S---SP--SCA-PS--------------------PSWWTIA-KGLP------EG--------LAV-KLSGY------DTIL-PG---VFSIMKVT---------V----ADF-G-YNILFCPYEE-----GS-LNR--C---QY-LQISS-D-----DKGN----RALVLGD--SDE-N--AMWVVFKK---------

>c26779_g2_i1_m_18736

--------VLDTDGNVLLN------------------S--GTYQILQA------------IILNGGG---LEYAA-T-----G-NE-T---C---PLTVVS-S-----PFH--TNH-GF--PVVI-SS-PL------LFTHI-SEG-FPL-YLR---F-T-S---SP--SCA-PS--------------------PSWWTIA-KGLP------EG--------LAV-KLSGY------DTIL-PG---VFSIMKVT---------V----ADF-G-YNILFCPYEE-----GS-LNR--C---QY-LQISS-D-----DKGN----RALVLGD--SDE-N--AMWVVFKK---------

>isotig09582_m_12928

--------VLDTDGNVLVN------------------R--GRYYVLQA------------VTLNGGG---LEYAA-T-----G-NE-T---C---PLTVVG-S-----PFH--TDH-GY--PVII-SS-PL------LFTHI-SEG-ILV-YFG---Y-A-S---AP--SCA-PH--------------------SFWWTVV-KGLQ------EG--------LAV-KLEYY------SNLV-LG---GFNIVKAS---------V----ADF-G-YNILFCPFEE-----GT-VSE--C---KY-VQISS-D-----DKGN----RALVLSD--SEE-N--AIWVVFKK---------

>c26779_g1_i1_m_18735

--------VLDTDGNVLVN------------------R--GRYYVLQA------------VTLNGGG---LEYAA-T-----G-NE-T---C---PLTVVG-S-----PFH--TDH-GY--PVII-SS-PL------LFTHI-SEG-ILV-YFG---Y-A-S---AP--SCA-PH--------------------SFWWTVV-KGLQ------EG--------LAV-KLEYY------SNLV-LG---GFNIVKAS---------V----ADF-G-YNILFCPFEE-----GT-VSE--C---KY-VQFSS-D-----DKGN----RALVLSD--SEE-N--AIWVVFK----------

>isotig01407_m_2393

--------LFDTAGNWVEN------------------G--GSYYIKPY------------IWKLGGG---LKLAK-T-----G-SE-T---C---PLSVVQ-S-----PKS--GQY-GI--PVKI-SA-PY------KSRYI-RPG--KV-YLS---F-VDE---VP--TCV-TT--------------------PATWTLV-KGLS------QK--------PLV-KNT--------VNTT-KCLLWRLNFNKVN--------------RF----KKPGFGSQ----------------------LGSIE------YSPGSR---NDLTSSS--V---D--SISVQDNITY-------

>isotig01408_m_2395

--------LFDTAGNWVEN------------------G--GSYYIKPY------------IWKLGGG---LKLAK-T-----G-SE-T---C---PLSVVQ-S-----PKS--GQY-GI--PVKI-SA-PY------KSRYI-RPG--KV-YLS---F-VDE---VP--TCV-TT--------------------PATWTLV-KGLS------QK--------PLV-KVTGY------ENTV-EG---SFKVERAL---------Y---QQDY-S-HKLFFCA--------SD-SDH--C---EN-IGIVN-------GNND----RALGVVS--KND-P--AFEIIFDK---------

>isotig01460_m_2516

--------IIDGTGKPVVT------------------G--STYYIIPA------------IWAHGGG---LRLGQ-T-----G-NE-T---C---PLSVVQ-I-----PFE--LNN-GL--PLKI-SS-PY------RSYFL-FPG-SKL-DLS---F-T-T---VP--TCA-ST--------------------PSAWTVV-KGLS------GQ--------PLV-KVTGY------ENTL-NG---YFAIQESS---------T-GFSLQN-S-YKFQFCT--------FD-SDE--C---AN-IGIVK-G-----DNND----RQLGLIS--EDD-P--AFMFVLVN---------

>c29416_g2_i1_m_29755

--------IIDGTGKPVVT------------------G--STYYIIPA------------IWAHGGG---LRLGQ-T-----G-NE-T---C---PLSVVQ-I-----PFE--LNN-GL--PLKI-SS-PY------RSYFL-FPG-SKL-DLS---F-T-T---VP--TCA-ST--------------------PSAWTVV-KGLS------GQ--------PLV-KVTGY------ENTL-NG---YFAIQESS---------T-GFSLQN-S-YKFQFCT--------FD-SDE--C---AN-IGIVK-G-----DNND----RQLGLIS--EDD-P--AFMFVLVN---------

>isotig09071_m_12312

--------IVDTDGEPVRN------------------G--GSYYVLPL------------IWSLGGG---LGLGQ-T-----G-NE-T---C---PLSVVQ-T-----LFR--VSN-GL--PLEI-SS-PY------RLPFL-MPG-HRL-DLS---F-T-I---VP--TCA-TT--------------------PSKWTVV-KGLS------EQ--------PLV-KVTGY------ENTI-EG---AFVIQHAS---------S-KVDIPN-T-YKFQFCQ--------FN-SVE--C---EN-IGIVK-G-----DKND----RLLAVIS--KED-L--EFPFVLVK---------

>c29416_g3_i1_m_29756

--------IVDTDGEPVRN------------------G--GSYYVLPL------------IWSLGGG---LGLGQ-T-----G-NE-T---C---PLSVVQ-T-----LFR--VSN-GL--PLKI-SS-PY------RLPFL-MPG-HRL-DLS---F-T-I---VP--TCA-TT--------------------PSKWTVV-KGLS------EQ--------PLV-KVTGY------ENTI-EG---AFVIQHAS---------S-KVDIPN-T-YKFQFCQ--------FN-SVE--C---EN-IGIVK-G-----DKND----RLLAVIS--KED-L--EFPFVLVK---------

>isotig09219_m_12494

--------IVDSDGRPVVN------------------G--AQYYVIPS------------VLSLGGD---LGLAQ-T-----G-NE-T---C---PISVVQ-V-----PFQ--ILN-GL--PLKI-SS-PI------KTLFI-EEG-YRL-DLS---F-T-K---LP--TCA-TT--------------------PSAWTVV-KGLS------EK--------PLV-KVTGY------DNTL-NG---AFVIQKAS---------S-KIPFQN-S-YKFQFCQ--------FD-SDE--C---SN-IGIVK-D-----DSTN--GIRLLGVTS--KDD-P--ALEFVLV----------

>c29416_g1_i1_m_29754

--------VYDIEGKPIQN------------------G--GSYYLLPT------------IWSSGGG---IGLAK-T-----G-NE-T---C---PLSVVQ-S-----PSK--LSN-GL--PVKI-SS-PF------TSLFV-PQG--RV-SLS---F-A-A---VP--TCA-TT--------------------PSKWTVV-KGLP------EG--------PVV-KLSGY------ENTI-DG---VFYIHKAS--------------NIL-T-YKLLFCI--------ID-NTV--C---GF-VGIVK-D-----DDDN----LLLAVT----RD-N--GFQFGLVK---------

>contig00104_m_14

--------LVDTDGDFVKN------------------G--GSYFLAPI-------------WRPGGG---AVVLQ-T-----G-NE-I---C---PITVAQ-E-----PV---GGS-GL--PVQF-ST-PK------KILYL-SEG-SYL-TVR---F-S-L---VP--WCA-PT--------------------PSQWTVL-EGSH------Q-----------V-KLTGY------NNTV-PG---SFKVVESA--------------DNG-V-YSLK-------------------------------------------------------------------------------

>c28083_g1_i2_m_23337

--------LVDTDGDFVRN------------------G--GSYFLAPI-------------WRPGGG---AVVLQ-T-----G-NE-I---C---PITVAQ-E-----PV---GGS-GL--PVQF-ST-PK------KILYL-SEG-SYL-TVR---F-S-L---VP--WCA-PT--------------------PSQWTVL-EGSH------Q-----------V-KLTGY------NNTV-PG---SFKVVESA--------------DNG-V-YSLKFCPLP------ID-DGT--C---GS-VGGAR---------------GPLVVY----DS-N--TFLFRFQK---------

>contig00111_m_17

--------LVDTDGDFVRN------------------G--GSYFLAPV-------------WRPGGG---ATVSQ-T-----G-NE-I---C---PITVAQ-E-----SV---VGN-GL--PVQF-ST-PK------LILHI-SEA-DYL-QAR---F-S-L---VP--WCA-PT--------------------PSEWTVL-EGSH------E-----------V-KLTGY------NNTV-PG---LFKVVESP--------------SDG-I-YSLR-------------------------------------------------------------------------------

>c28083_g1_i1_m_23336

--------LVDTDGDFVRN------------------G--GSYFLAPV-------------WRPGGG---ATVSQ-T-----G-NE-I---C---PITVAQ-E-----SV---VGN-GL--PVQF-ST-PK------LILHI-SEA-DYL-QAR---F-S-L---VP--WCA-PT--------------------PSEWTVL-EGSH------E-----------V-KLTGY------NNTV-PG---LFKVVESP--------------SDG-I-YSLRFCPLP------ID-DGT--C---GS-VGRAR---------------GPLVVY----DS-N--TFLFRFQK-----PKL-

>isotig08256_m_11234

--------LLDTDGEAVRN------------------A--GVYFMAPV-------------LRPGGG---ITVTQ-S-----E-NE-I---C---PITVMK-E-----QV---LGR-GL--PVQF-IS-RL------KIATL-SEG-DLL-TVR---F-P-L---VP--WCA-PT--------------------PSQWTVL-RGSN------E-----------V-KLSGY------NNTV-SG---RFKVVKGT--------------FQN-S-YTLSFCPL----------VGI--C---RR-VGGPYFG-----GSGN------LVVGA---KA-N--VMQFRFQK---------

>isotig00406_m_765

--------LLDINGEEVVN------------------G--GWYFMVSV-------------MRPGGG---ITALQ-S-----E-NE-I---C---PITVSK-L-----SL---WGS-GL--PVEF-ST-PL------AILNL-REV-DYL-TVR---F-P-L---VP--WCA-PT--------------------PSQWTVL-EGSK------E-----------V-KLTGY------NETV-SG---KFKVKHVS---------G------T-D-YSLYFCSS----------AST--C---EP-VGGPSFG-----GIGN------LVVGP---EA-N--DMRFMFKKFTGQLGEV-

>c6564_g1_i1_m_1144

--------LLDINGEEVVN------------------G--GWYFMVSV-------------MRPGGG---ITALQ-S-----E-NE-I---C---PITVSK-L-----SL---WGS-GL--PVEF-ST-PL------AILNL-REV-DYL-TVR---F-P-L---VP--WCA-PT--------------------PSQWTVL-EGSK------E-----------V-KLTGY------NETV-SG---KFKVKHVS---------G------T-D-YSLYFCSS----------AST--C---EP-VGGPSFG-----GIGN------LVVGP---EA-N--DMRFMFKKFTGQLGEV-

>Gm_I1KYW3_27_197

--------VLDVDGDPIRN------------------G--FIYYVLPA------------IRGNGGG---IERAA-L-----G-KD-T---C---PITVVQ-S-----PNP--NSK-GL--EIKF-ES-AY------PAYYI-NET-LIL-QIK---F-S-Y---PQ--QCE-RK--------------------NPWWAIS-KDIS------EGP-------PAI-KLSGF------HGTE-LG---WFKIQKAS---------K--SCDSN-D-YKLVFCQ--------YD-ETW--C---LD-VGIYV-D-----RQGN----RRLVLAV---TG-E--PFLVHFHK---------

>Gm_C6TB67_27_197

--------VLDVDGDPIRN------------------G--FIYYVLPA------------IRGNGGG---IERAA-L-----G-KD-T---C---PITVVQ-S-----PNP--NSK-GL--EIKF-ES-AY------PAYYI-NET-LIL-QIK---F-S-Y---PQ--QCE-RK--------------------NPWWAIF-KDIF------EGP-------PAI-KLFGF------HGTE-LG---WFKIQKAS---------K--SRDFN-D-YKLVFCQ--------YD-ETW--C---LD-VGIYV-D-----RQGN----RRLVLAV---TG-E--PFLVHFHK---------

>contig00100_m_13

--------IVDKNGELVEN------------------G--GSYYIIPH------------GSFYGDG---LGAAE-M-----G-NE-T---C---PLSVVL-K-----PLD--ASK-GL--AVTL-SS-RF------RSAVL-ISG--IV-DIS---F-T-A---VP--TCA-ET--------------------PSKWTVV-SDLT------PV--------PLV-KLTGY------QNT--NG---VFIIEKAY---------T---SSDN-T-YSLSFCL--------IN-SNQ--C---QP-VGLER-DF---ISFSN----NMLFVVS--RSV-T--PFTFVLDK---------

>c7831_g1_i1_m_1303

--------IVDKNGELVEN------------------G--GSYYIIPH------------GSFYGDG---LGAAE-M-----G-NE-T---C---PLSVVL-K-----PLD--ASK-GL--AVTL-SS-RF------RSAVL-ISG--IV-DIS---F-T-A---VP--TCA-ET--------------------PSKWTVV-SDLT------PV--------PLV-KLTGY------QNT--NG---VFIIEKAY---------T---SSDN-T-YSLSFCL--------IN-SNQ--C---QP-VGLER-DF---ISFSN----NMLFVVS--PSV-T--PFTFVLDK---------

>c22388_g1_i1_m_8774

-------------GNIARN------------------G--GQFYIKPR------------IFSLGGG---IRLVK-T-----G-NE-T---R---PLSVVQ-S-----PLE--TDV-GL--PVTI-SS-PY------RVEFI-PEG--PV-NLD---F-D-H-------PLG-SK--------------------SYEWIAV-GDHS------EG--------TFV-KV-GY------QN-----------------------------------------------------------------------------------------------------------------------

>Ptet_P25700_3_176

--------LVDVEGKTVRN------------------G--GTYYLVPQ------------LRPGGGG---MEAAK-V-----G-NE-D---C---PLTVVK-S-----LDE--NSN-GE--PIRI-AS-RL------RSTFI-PEY-SLV-NLG---F-A-D---PP--KCA-P---------------------SPFWTVV-KDQS------ERL-------PSI-KLGEY------KDSELDY---PFKFERVY---------A--ASKMY-A-YKLLYCGSEDE----EE-EMM--C---KD-IGVYR-D-----QEGY----QRLVVS----KH-N--PLVVGFKK---------

>Ptet_A6XBI5_3_176

--------LVDVEGKTVRN------------------G--GTYYLVPQ------------LRPGGGG---MEAAK-V-----G-NE-D---C---PLTVVK-S-----LDE--NSN-GE--LIRI-AS-RL------RSTFI-PEY-SLV-NLG---F-A-D---PP--KCA-P---------------------SPFWTVV-KDQS------ERL-------PSI-KLGEY------KDSELDY---PFKFERVY---------A--ASKMY-A-YKLLYCGSEDE----EE-EMM--C---KD-IGVYR-D-----QEGY----QRLVVS----KH-N--PLVVGFKK---------

>Ptet_Q7M1S6_3_176

--------LLDVEGKAVRN------------------G--GTYYLVPQ------------LRPHGGG---IEVAK-I-----G-KE-D---C---PLTVVK-S-----LDE--NSN-GE--PIMI-AS-PL------RSAFI-PEY-SLL-KIG---F-S-D---PP--KCA-P---------------------SPWWTVV-KDQS------ERR-------PTI-KLSEY------KRSELDY---PFQFERVY---------T--ASKMY-A-YKLLYCGSEDE----EE-EII--C---KD-IGIFR-D-----QEGY----QRLIVS----RK-N--PLVVGFKK---------

>Ptet_B4F6G4_3_168

--------LLDSEGELVRN------------------G--GTYYLLPD------------RWALGGG---IEAAA-T-----G-TE-T---C---PLTVVR-S-----PNE--VSV-GE--PLRI-SS-QL------RSGFI-PDY-SLV-RIG---F-A-N---PP--KCA-P---------------------SPWWTVV-EDQP------QQ--------PSV-KLSEL------KSTKFDY---LFKFEKVT---------S----KFS-S-YKLKYCAK----------RDT--C---KD-IGIYR-D-----QKGY----ARLVVT----DE-N--PLVVIFKK---------

>Ptet_P32877_3_168

--------LLDSEGELVRN------------------G--GTYYLLPD------------RWALGGG---IEAAA-T-----G-TE-T---C---PLTVVR-S-----PNE--VSV-GE--PLRI-SS-QL------RSGFI-PDY-SLV-RIG---F-A-N---PP--KCA-P---------------------SPWWTVV-EDQP------QQ--------PSV-KLSEL------KSTKFDY---LFKFEKVT---------S----KFS-S-YKLKYCAK----------RDT--C---KD-IGIYR-D-----QKGY----ARLVVT----DE-N--PLVVIFKK---------

>Ptet_P10821_3_168

--------LLDSEGELVRN------------------G--GTYYLLPD------------RWALGGG---IEAAA-T-----G-TE-T---C---PLTVVR-S-----PNE--VSV-GE--PLRI-SS-QL------RSGFI-PDY-SVV-RIG---F-A-N---PP--KCA-P---------------------SPWWTVV-EDQP------QQ--------PSV-KLSEL------KSTKFDY---LFKFEKVT---------S----KFS-S-YKLKYCAK----------RDT--C---KD-IGIYR-D-----QKGY----ERLVVT----DE-N--PLVVIFKK---------

>Ptet_Q4U4G0_28_198

--------LVDVEGNLVEN------------------G--GTYYLLPQ------------ITAHGGG---IETAK-T-----G-NE-P---C---PLTVVQ-S-----PYE--VSN-GE--PIRI-SS-LF------LSLFI-PRG-SLV-ALG---F-V-N---PP--SCA-A---------------------SPWWTVV--DSP------QG--------PAV-KLSQQ------KTSD-QDT-VVFQFQKVS---------S---SNLN-A-YKLLYCQREE------E-DVK--C---DQYIGVHR-D-----RNGN----RRLVVT----KK-N--PLELVLVK---------

>Ptet_Q43721_28_198

--------LVDVEGNLVEN------------------G--GTYYLLPQ------------ITAHGGG---IETAK-T-----G-NE-P---C---PLTVVQ-S-----PYE--VSN-GE--PIRI-SS-LF------LSLFI-PRG-SLV-ALG---F-V-N---PP--SCA-A---------------------SPWWTVV--DSP------QG--------PAV-KLSQQ------KLPE-KDI-LVFKFEKVS---------H---SNIH-V-YKLLYCQRDE------E-DVK--C---DQYIGIHR-D-----RNGN----RRLVVT----KK-N--PLELVLVK---------

>Ptet_P10822_28_198

--------LVDAEGNLVEN------------------G--GTYYLLPH------------IWAHGGG---IETAK-T-----G-NE-P---C---PLTVVR-S-----PNE--VSK-GE--PIRI-SS-QF------LSLFI-PRG-SLV-ALG---F-A-N---PP--SCA-A---------------------SPWWTVV--DSP------QG--------PAV-KLSQQ------KLPE-KDI-LVFKFEKVS---------H---SNIH-V-YKLLYCQHDE------E-DVK--C---DQYIGIHR-D-----RNGN----RRLVVT----EE-N--PLELVLLK---------

>Ptet_Q43708_28_198

--------LVDAEGNLVEN------------------G--GTYYLLPH------------IWAHGGG---IETAK-T-----G-NE-P---C---PLTVVR-S-----PNE--VSK-GE--PIRI-SS-QF------LSLFI-PRG-SLV-ALG---F-A-N---PP--SCA-A---------------------SPWWTVV--DSP------QG--------PAV-KLSQQ------KLPE-KDI-LVFKFEKVS---------H---SNIH-V-YKLLYCQRDE------E-DVK--C---DQYIGIHR-D-----RNGN----RRLVVT----EE-N--PLELVLLK---------

>isotig09642_m_13008

--------LVDAEGNLVEN------------------G--GTYYLLPH------------IWAHGGG---IETAK-T-----G-NE-P---C---PLTVVR-S-----PNE--VSK-GE--PIRI-SS-QF------LSLFI-PRG-SLV-ALG---F-A-N---PP--SCA-A---------------------SPWWTVV--DSP------QG--------PAV-KLSQQ------KLPE-KDI-LVFKFEKVS---------H---SNIH-V-YKLLYCQRDE------E-DVK--C---DQYIGIHR-D-----RNGN----RRLVVT----EE-N--PLELVLLKAKSETASSH

>c17145_g1_i1_m_3533

--------LVDAEGNLVEN------------------G--GTYYLLPH------------IWAHGGG---IETAK-T-----G-NE-P---C---PLTVVR-S-----PNE--VSK-GE--PIRI-SS-QF------LSLFI-PRG-SLV-ALG---F-A-N---PP--SCA-A---------------------SPWWTVV--DSP------QG--------PAV-KLSQQ------KLPE-KDI-LVFKFEKVS---------H---SNIH-V-YKLLYCQRDE------E-DVK--C---DQYIGIHR-D-----RNGN----RRLVVT----EE-N--PLELVLLKAKSETASSH

>Gm_C6K8D0_31_222

--------VYDTDGDKLQY------------------G--VNYFVLPV------------IRGNGGG---IQVAK-A-----G-NE-T---C---PLTVVQ-S-----GNE--LSE-GL--PIKI-ASRSA------GVAFI-TQG-QLFKSIQFGVF-PST--LRP--GCP-PSPIPS----KWD--------PPSKWTIV-EGLP------ERG-------LAV-KLVGY------QNRV-SG---WFSIVKVA---------DDASSSSV-G-YKLVFCLWPEE-E--VM-IHL--C---KN-VGIRT-D-----GKGI----RRLVLS----EN-T--PLVVQFQK---------

>Gm_C6T599_31_222

--------VYDTDGDKLQY------------------G--VNYFVLPV------------IRGNGGG---IQVAK-A-----G-NE-T---C---PLTVVQ-S-----GNE--LSE-GL--PIKI-ASRSA------GVAFI-TQG-QLFKSIQFGVF-PST--LRP--GCP-PSPIPS----KWD--------PPSKWTIV-EGLP------ERG-------LAV-KLVGY------QNRV-SG---WFSIVKVA---------DDASSSSV-G-YKLVFCLWPEE-E--VM-IHL--C---KN-VGIRT-D-----GKGI----SAWCCL----RT-L--PIAVQFQK---------

>Gm_C6SVL0_27_194

--------VYDTDGNILQK------------------G--RTYFVLAA------------TRGNGGG---IEFAA-T-----G-ND-T---C---PLTVVQ-S-----PSG--DSI-GF--PIRF-SY-PFI----ETLDDI-LEG-FGL-EIR---F-A-Y---RP--PCS-PSSIA-----------------TTKWTIV-N-------------------DAV-KLTKTS-----SSII-PG---FFYIWGAS---------P----NSK-D-YKLEFCD--------LD-NIK--C---GD-VGIHT-D-----DDGS----RRLVIT----QN-D--PLLVHFQN---------

>isotig07854_m_10657

--------VYDSDGNPLQN------------------G--VAYYIVDA------------SSYGGGG---LQYVK-T-----G-NE-T---C---PLTVVY-S-----DSW--HSN-GY--PLNI-TS-SES----TYLSNI-ALN-STV-NLA---F-I-N---PP--SCG-LT--------------------PSEWTVV-KGLP------EG--------DAV-KLTGY------PKIE-LG---NFRIKELD---------E----DSD-G-YNIEFCAFDIVGH--EY-TEV--C---KY-VDFYW-D-----RSGN----KRLVLTD--DED-G--AAYFEFEK---------

>c28968_g2_i1_m_27305

--------VYDSDGNPLQN------------------G--VAYYIVDA------------SSYGGGG---LQYVK-T-----G-NE-T---C---PLTVVY-S-----DSW--HSN-GY--PLNI-TS-SES----TYLSNI-ALN-STV-NLA---F-I-N---PP--SCG-LT--------------------PSEWTVV-KGLP------EG--------DAV-KLTGY------PKIE-LG---NFRIKELD---------E----DSD-G-YNIEFCAFDIVGH--EY-TEV--C---KY-VDFYW-D-----RSGN----KRLVLTD--DED-G--AAYFEFEK---------

>isotig08014_m_10899

--------VYDSDGNPLQN------------------G--VAYYIVDA------------SFYGSGG---LQYVK-T-----G-NE-T---C---PLTVVN-L-----ASW--SNN-GY--QLNI-TS-SDT----PFLSNI-TLE-TSV-NFA---F-I-N---PP--ACG-LT--------------------PSEWTIV-KGLP------EG--------AAV-KLTGY------PKIA-FG---SFKIKKSH---------------SE-G-YNIEFCSIEIVDH--YY-TEV--C---EY-VNFYR-D-----RRGN----RRLVLTN--KDY-N--AVCFAFKK---------

>isotig08832_m_12014

--------VFDSDGNPLKN------------------G--VAYYMVHA------------VFLSGGG---LQYIK-T-----G-NE-T---C---PLTVVN-M-----VSW--NNK-GY--PLNI-TS-SDT----PYLSDI-SEE-TSV-NIA---F-T-N---PP--ACG-LT--------------------PSEWTIV-KGLP------EG--------AAV-KLTGY------PNIA-FG---SFKIKKSY--------------FGI-G-YNIEFCSIEIVDH--YY-TEV--C---KL-LDIYW-D-----KMRN----KRLVLTD--SKT-S--ALWFKFKK---------

>c28968_g3_i1_m_27307

--------VFDSDGNPLKN------------------G--VAYYMVHA------------VFLSGGG---LQYIK-T-----G-NE-T---C---PLTVVN-M-----VSW--NNK-GY--PLNI-TS-SDT----PYLSDI-SEE-TSV-NIA---F-T-N---PP--ACG-LT--------------------PSEWTIV-KGLP------EG--------AAV-KLTGY------PNIA-FG---SFKIKKSY--------------FGI-G-YNIEFCSIEIVDH--YY-TEV--C---KL-LDIYW-D-----KMRN----KRLVLTD--SKT-S--ALWFKFKK---------

>isotig09597_m_12947

--------VYDSDGNPLIN------------------G--GAYYIVQA------------ISLSGGG---VQYEK-T-----E-NE-T---C---PLTVVN-L-----ASS--SNN-GY--PLNI-TS-SDT----PFLSNI-TEE-TSV-NIE---F-I-N---PP--PCG-LT--------------------PSEWTAV-KGLP------EG--------TAV-KLTGY------PKIE-LG---SFKIKKSY--------------VSI-G-YNIEFCSIEIVDH--YY-TEV--C---EY-VDIST-D-------------KRLVLTD--NNL-G--AAWFQFKK---------

>isotig08069_m_10981

--------VYDSSGKPLQN------------------G--VAYYIVQS------------IGFSGGG---VEYRK-T-----G-NE-T---C---PLTVVN-S-----GPW--FAN-GL--PVNI-TS-SDT----RYLSNI-SLE-TSV-NFA---F-T-N---PP--PCG-LT--------------------PSEWTVV-KGLP------EG--------SAV-KLTGY------PNIA-FG---SFKIKKSW---------------GL-G-YNIEFCSIEIVDH--YY-TEV--C---EY-VTVST-D-----KKGN----KLLVLKA--SDT-N--AVWFVFKK---------

>c28968_g1_i1_m_27304

--------VYDSSGKPLQN------------------G--VAYYMVQS------------IGFSGGG---VEYRK-T-----G-NE-T---C---PLTVVN-S-----GPW--FAN-GL--PVNI-TS-SDT----RYLSNI-SLE-TSV-NFA---F-T-N---PP--PCG-LT--------------------PSEWTVV-KGLP------EG--------SAV-KLTGY------PNIA-FG---SFKIKKSW---------------GL-G-YNIEFCSIEIVDH--YY-TEV--C---EY-VTVST-D-----KKGN----KLLVLKA--SDT-N--AVWFVFKK---------

>isotig10039_m_13496

--------VLDTDGNPVEN------------------K--GKYYILPAP-----------SEQRGGG---IEAAA-T-----G-NE-T---C---PLTVVL-S-----PIK--SSK-GK--PILI-SS-PT------FVRFI-SEE-RML-KFE---F-N-G---VP--TCA-S---------------------SPLWSVV-KGASSPSEEEEK--------AAV-KLSGYY-----ENAV-KG---WFEIRKVS---------D------G-V-YKLGFCARD---------ADT--C---ES-IGFNV-D-----EFGY----RHLVLT----ED-H--PVQVVFEK---------

>Gm_C6SYK2_29_201

--------VLDTDGHAVEN------------------H--GTYYLLPAK-----------SGSGGGG---IEVAA-T-----G-KE-S---C---ALTVVQ-S-----LNE--DSM-GL--PLKL-SS-PSI-----TTSHF-TEY-TSL-SIE---F-T-S---AP--APC-SS--------------------ASEWTVV-KGLP------EG--------RAV-KLNDY------GNTV-EG---DFAFVCAK---------REFYRCNK-S-YQLIFCP--------YG-LMR--C---ED-VGISI-D-----DDGN----RRLVIS----DG-N--PFLFKLQK---------

>Gm_I1KYX3_29_201

--------VLDTDGHAVEN------------------H--GTYYLLPAK-----------SGSGGGG---IEVAA-T-----G-KE-S---C---ALTVVQ-S-----LNE--DSM-GL--PLKL-SS-PSI-----TTSHF-TEY-TSL-SIE---F-T-S---AP--APC-SS--------------------ASEWTVV-KGLP------EG--------RAV-KLNDY------GNTV-EG---DFAFVCAK---------REFYRCNK-S-YQLIFCP--------YG-LMR--C---ED-VGISI-D-----DDGN----RRLVIS----DG-N--PFLFKLQK---------

>isotig06462_m_8385

--------VYDTDGDRLTD------------------G--SRYYVLPV------------EEGSFGG---IEATA-T-----G-KD-T---C---PLTVVQ-S-----SDK--NSK-GI--PISI-IL-PY-----SRTGLV-RDD-FPL-IIR---F-VPS-AVTT--SCV-GI--------------------STAWSLV--------------------NEAV-KLDVDP-----NDAV-HG---YFEIDIVS---------S----ANH-H-YKFKFCG-----------RDS--C---RS-VGAFD-D-----AKGK----RRLVLSN------N--PLLVKFEK---------

>isotig02703_m_4313

--------VFDSDGEPITI------------------G--ASYYLAPV-------------GRYGGG---IERTA-T-----G-NE-T---C---PLTVVQ-S-----PYD--YSP-GS--PIRF-SS-PN-----KATYNI-HEG-ASL-DVG---F-E-C---VP--WCA-PT--------------------PSKFTIV-RGDD---QQGEQ--------FSV-KLSGSS-----ENTV-SG---SFSFQSANYYGLGLKLKS---WRRY-A-YTLVFCTD----------DGT--C---RG-VGIYA-D-----RYGN----LRLVLTQR-----D--PVVLQIRS---------

>c31193_g1_i1_m_41593

--------VFDSDGEPITI------------------G--ASYYLAPV-------------GRYGGG---IERTA-T-----G-NE-T---C---PLTVVQ-S-----PYD--YSP-GS--PIRF-SS-PN-----KATYNI-HEG-ASL-DVG---F-E-C---VP--WCA-PT--------------------PSKFTIV-RGDD---QQGEQ--------FSV-KLSGSS-----ENTV-SG---SFSFQSANYYGLGLKLKS---WRRY-A-YTLVFCTD----------DGT--C---RG-VGIYA-D-----RYGN----LRLVLTQR-----D--PVVLQI-----------

>isotig08164_m_11114

--------VLDSDGEPLTV------------------G--STYYVMPV-------------GRYSGG---IEPIV-T-----G-NE-T---C---PMTVVQ-S-----PYD--YSP-GS--PIRF-SP-PN-----QTTNKI-YEG-VSF-DVG---F-G-C---VP--WCA-QT--------------------PSKFTVV-RGDD---QRGEQ--------FSL-KLSGSS-----ENTV-SG---SFSFQTANYYGLG--FKS---WRRY-A-YTLVFCTD----------DGT--C---RG-VGIYV-D-----RYGN----RLLVLTQR-----D--PVVLQIRS---------

>Ptet_Q43325_23_185

--------VYDTNGNQLRN------------------N--GEYYIVPV--------------SGGAG---IDVVA-T-----G-TE-K---C---PLTIVQ-S-----SSST-----KY--STVF-KS-EATL--PRPINYI-TQN-MAF-SIK---F-G-C---NQ------YR--------------------GRPWTVV-RGLP------EG--------LAL-KLNGY------TNTA--R---VLRIKPYS---------P----GSR-N-YKLLFCPD----------GSQ--C---AN-VGFTL-M-----LSER----KRLVVSN---TL-P--TLQVQFQK---------

>c23070_g1_i1_m_10023

--------VYDTNGNQLRN------------------N--GEYYIVPV--------------SGGAG---IDVVA-T-----G-TE-R---N---PLTIVQ-S-----SSST-----KY--STVF-KS-EATL--PPPINYI-TQN-MAF-SIK---F-G-N---NQ------YR--------------------GRPWTVV-RGLP------EG--------LAL-KLNGY------TNTA-PG---NFRIKPYS---------P----GSR-N-YKLLFCPD----------GSQ--C---AN-VAVYT-D-----AQRK----KRLVVSN---TL-P--TLQVQFQK---------

>isotig08531_m_11611

--------VYDTNGNQLAN------------------N--GEYYIVPVT-----------GGATPNG---IDVLP-T-----G-TE-E---S---SHTIVQ-A-----SRGI-----RF--STVF-TS-NVEV--RPRPRFI-LQN-MAF-DIE---F-G-----AP------YK--------------------GQPWIVV-STQP------EG--------LAL-KLGGY------KTTV-KG---HFRIQKFS---------A----GST-N-YKLLFCPK----------VGQ--C---GN-IAVYT-D-----GDGL----RRLVVSD---SK-A--ALEVQFQK---------

>c23070_g2_i1_m_10024

--------VYDTNHNRVLN------------------N--GEYYILPI---------------KGSG---IEVLQ-T-----G-SD-P---Y---PLTIVQ-S-----SRL------KL--STVF-SS-KIEI----PTNYI-SQN-QAF-NIT---F-G-----DP------FR--------------------GKKWTVV-NNQP------EG--------LAL-KVEGYY-----TNTV-KG---NFRIKIFS---------A----TSA-N-YKLLFCPD----------VGQ--C---GN-IAVYT-D-----TEGF----NRLVVSN---TL-A--ALEVKFQK---------

>Ptet_P15465_5_171

--------VYDAEGNKLVN------------------R--GKYTIVSF--------------SDGAG---IDVVA-T-----G-NE-N---PE-DPLSIVK-S-----TRN--IMY-----ATSI-SS-EDKT--PPQPRNI-LEN-MRL-KIN---F-A-T---DP------HK--------------------GDVWSVV-DFQP------DG--------QQL-KLAGRY-----PNQV-KG---AFTIQKGS---------N----TPR-T-YKLLFCPV----------GSP--C---KN-IGIST-D-----PEGK----KRLVVSY---QS-D--PLVVKFHR---------

>isotig09482_m_12803

--------ILDTDGKELRN------------------P--GQYYILPV------------KSESGRG---LDLVS-T-----G-NE-D---C---PRTIAL-A-----TRDF-----KL--RTIF-SS-LV------PSPFI-LQE-DPI-SIKFRKL-A-I---TT--RCI-PI--------------------TLEWTVV-KGLP------EG--------LAL-KADGY------ENQV-RG---TFFIQAAS---------S----TNN-H-YKILFCSYG---------KNP-FC---AN-LAVYD-D-----GQGT----KRLVVSN---KT-D--PLVVQFEK---------

>c25292_g1_i1_m_14709

--------ILDTDGKELRN------------------P--GQYYILPV------------KSESGRG---LDLVS-T-----G-NE-D---C---PRTIAL-A-----TRDF-----KL--RTIF-SS-LV------PSPFI-LQE-DPI-SIKFRKL-A-I---TT--RCI-PI--------------------TLEWTVV-KGLP------EG--------LAV-KADGY------ENQV-RG---TFFIQAAS---------S----TNN-H-YKILFCSYG---------KNP-FC---AN-LAVYD-D-----GQGT----KRLVVSN---KT-D--PLVVQFE----------

>c25292_g2_i1_m_14710

VPSLNAQPILDTEGNLLEN------------------P--GEYYILPV------------TSESGRG---LDLVS-T-----G-NE-H---C---PQTIAL-A-----TRDH-----KL--PTIF-SS-ML------RIPVI-FQG-HPI-SIELGNF-A-I---IT--KCI-PA--------------------SPLWTVV-KGLP------EG--------LAV-KIDGF------ENQV-PG---RFTIREAS---------S----TNS-H-YKILFCPYE---------YSS--C---TN-LAVYD-D-----GEGT----KRLVVSN---ET-D--PLVVQF-----------

>isotig12801_m_16475

--------IFDREGKRLEN------------------P--GHYYILPV------------TSESGRG---LDLVS-T-----G-NE-T---C---PRTIAL-A-----TRDI-----KL--PTNF-LS-LM------HVPFI-REE-YPF-SIDIGNF-N-I---KA--RCI-PI--------------------APAWTVV-KGLQ------EG--------LAV-KAYGY------ENKV-PV---ALSFEKLP---LPI--------------TTTRFCS----------------------------------------------------------------------------

>Gm_I1JLM5_9_85

--------VHDTDDRMLEN------------------G--GDYFILPS------------FDEEGGG---VTLAS-I-------GV-T---H---PLAVVQ-S-----SSK--NHL-GL--PASI-KT---------KETMI-IFT-DEM-NIM------------------------------------------------------------------------------------------------RLQK----------------------Y----------------------------------------------------------------------------------

>pv_V7C570_33_198

--------VVDKQGEPLVP------------------G--VGYYVWPL------------WA-DEGG---LTLGQ-T-----R-NK-T---C---PLDVIR-D-----PSFI-----GS--PVAF-SA--------EGLDHI-PTL-TDL-TID---F-P-V---VT--ACN-Q---------------------PTVWKLS-KEG------SGF--------WFV-STSGN------PQDI-TS---KFKIERLE---------G--DHAYE-I-YSFKFCPS-------RP-GVL--C---AP-VGTFQ-D-----SDGT----KVMAVGD--NI--D--PYYVRFQK---------

>Gm_K7MIV6_34_199

--------VVDKQGNPLVP------------------G--VGYYVWPL------------WA-DNGG---LTLGQ-T-----R-NK-T---C---PLDVIR-D-----PSFI-----GS--PVRF-HA--------SGLNHI-PTL-TDL-TID---F-P-V---VT--VCN-Q---------------------PTVWRLS-KEG------SGF--------WFV-STRGN------PQDL-IT---KFKIERLE---------G--DHAYE-I-YSFKFCPS-------VP-GVL--C---AP-VGTFV-D-----ADGT----KVMAVGD--NI--D--PYYVRFQK---------

>Gm_B1ACD3_34_199

--------VVDKQGNPLEP------------------G--VGYYVWPL------------WA-DEGG---LTLGQ-T-----R-NK-T---C---PLYVIR-D-----PSFI-----GT--PVSF-LA--------PGLDHV-PTL-TDL-TID---F-P-V---VT--VCN-Q---------------------PTVWRLN-KVG------SGF--------WFV-STSGD------PNDI-TS---KFKIERLE---------G--DHAYE-I-YSFKFCPS-------VP-GAL--C---AP-VGTFE-D-----ADGT----KVMAVGD--DI--E--PYYVRFQK---------

>isotig08485_m_11543

--------VVDKQGNPLEP------------------G--VGYYVWPL------------WA-DNGG---LTLGQ-T-----R-NK-T---C---PLDVIR-D-----PSFI-----GT--PVSF-LA--------PGLDHI-PTL-TDL-TID---F-P-V---TT--ICN-Q---------------------PTVWRLL-KEG------SGF--------WFV-STSGN------PGDL-TS---KFKIERLA---------G--EHAYE-I-YSFKFCPS-------VP-GVL--C---AP-VGTFE-D-----ADGT----KVMAVGD--NI--E--PYYVRFQK---------

>Mt_I3SZS6_29_196

--------VVDKQGNPLKP------------------G--EGYYVFPL------------WA-DNGG---ITLGR-T-----R-NK-T---C---PLDVIR-N-----PDAI-----GT--PVYF-SA--------SGLDYI-PTL-TDL-TIE---I-P-I--LGS--PCN-E---------------------PKVWRLL-KVG------SGF--------WFV-STGGA------AGDL-VS---KFKIERLA---------G--EHAYE-I-YSFKFCPS-------VP-GVL--C---AP-VGTFV-D-----TDGT----KVMAVGD--GIE-E--PYYVRFQK---------

>Mt_G7JAF9_29_196

--------VVDKQGNPLKP------------------G--EGYYVFPL------------WA-DNGG---ITLGH-T-----R-NK-T---C---PLDVIR-N-----PDAI-----GT--PVYF-SA--------SGLDYI-PTL-TDL-TIE---I-P-I--LGS--PCN-E---------------------PKVWRLL-KVG------SGF--------WFV-STGGA------AGDL-VS---KFKIERLA---------G--EHAYE-I-YSFKFCPS-------VP-GVL--C---AP-VGTFV-D-----TDGT----KVMAVGD--GIE-E--PYYVRFQK---------

>Mt_G7JAG3_29_198

--------VVDKQGNPLKP------------------G--EGYYVFPL------------WA-DNGG---ITLGQ-T-----R-NK-T---C---PLDVIR-N-----PEAI-----GS--PVYF-YE--------YEHDYI-PTL-TDL-TVE---I-P-I--LGS--PCS-E---------------------RKVWKIS-KEGT----RARF--------WFV-STGGF------PGNL-FS---QFKIERLE---------G--EHAYE-I-YSFLYCPS-------VP-GTL--C---AP-VGTFV-D-----TDGT----KVMALGA--GIE-E--PYYVRFQK---------

>Mt_G7JAF9_239_408

--------VVDRHGKPLES------------------G--KGYYVWQF------------WAHDIGG---LTLSS-T-----R-NK-T---C---PLDVIR-N-----PKEL-----GS--PVYF-SA--------PGFKHI-PTQ-TDL-SIK---I-R-F--RSS--SCN-Q---------------------SKVLKLS-KEG------SGF--------WFL-STGGV------AGDV-VS---KFKIEKLE---------G--DTGIP-I-YIFKFCPS-------VP-GAL--C---AP-VRTFT-D-----TDGT----KVMAVGD--GNDLE--PYYVRFQR---------

>pv_V7AIA7_30_204

--------VMDTHGDPLES------------------D--EEYFIRPA------------ITDNGGR---FTLIN-------R-NQ-S---C---PLHVGL-E-----NTD--LPQ-GY--PVRF-TP-FAR---EEDDDEV-RVD-RDL-KVE---FVE-V---SS--TCV-Q---------------------STAWKLG-ENDT----SIGR--------RVI-VTGLDDG----THSA-GN---YFRIEETQ--------------NAS-I-YNIRWCPTEVC-P--TC-RFI--C---GT-GGIVR-------ENGT----ILFALDG------S--ALPVVFQK---------

>pv_V7AJ80_30_204

--------VMDTHGDPLES------------------D--EEYFIRPA------------ITDNGGR---FTLIN-------R-NQ-S---C---PLHVGL-E-----NTD--LPQ-GY--PVRF-TP-FAR---EEDDDEV-RVD-RDL-KVE---FVE-V---SS--TCV-Q---------------------STAWKLG-ENDT----SIGR--------RVI-ITGLDDG----THSA-GN---YFRIEETQ--------------NVS-I-YNIRWCPTEVC-P--TC-RFI--C---GT-GGIVR-------ENGT----ILFALDG------S--ALPVVFQK---------

>Gm_I1KW53_31_205

--------VFDTHGDPLET------------------D--DEYYIRPA------------ITDNGGR---FTLIN-------R-NR-S---C---PLYVGL-E-----NTD--TPQ-GY--PMKF-TP-FAN---KDDDDNL-RVN-TDL-KVT---LVQ-V---ST--TCV-Q---------------------STEWKLG-ENDT----RSGR--------RLI-VTGRDNG----IQSA-GN---YFRIVETE--------------SVG-I-YNIRWCPTEAC-P--TC-RFI--C---GT-GGILR-------ENGR----ILFALDG------T--TLPVVFQK---------

>Gm_C4XVM5_31_205

--------VFDTHGDPLET------------------G--DEYYIRTA------------ITDNGGR---FTLIN-------R-NR-S---C---PLYVGL-E-----NTD--TPQ-GY--PMKF-TP-FAN---KDDDDNL-RVN-TDL-KVT---LVQ-V---ST--TCV-Q---------------------STEWKLG-ENDT----RSGR--------RLI-VTGRDNG----IQSA-GN---YFRIVETE--------------SVG-I-YNIRWCPTEAC-P--TC-RFI--C---GT-GGILR-------ENGR----ILFALDG------T--TLPVVFQK---------

>Gm_I1MI59_26_203

--------VLDTNRERVDS------------------D--DEYYIRPA------------ITDNGGR---FTLIN-------R-NR-S---C---PLYVGL-E-----NTD--TPL-GY--PVKF-TP-FSRNNNDDDDDDI-RVN-RDL-RVA---FDE-V---ST--TCV-Q---------------------STEWRVG-ENDT----RSGR--------RLI-ITGRDET----TGSY-GN---YFRIVETE--------------NVG-I-YNIQWCPTEVC-P--TC-RFI--C---GT-GGILR-------ENGR----ILFALDG------T--PLPVMFQK---------

>isotig10611_m_14171

--------VLDTNGERVDS------------------D--DEYYIRPA------------ITDNGGR---FTLIN-------R-NG-S---C---PLYVGL-E-----NTD--TPQ-GY--PVKF-TP-FAHNN-NDDDDDI-RVN-RDL-RVT---FDE-V---ST--TCI-N---------------------SNEWRVG-ENDT----RSGR--------RLI-ITGRDNT----TGSY-GN---YFRIVETQ--------------NAS-I-YNIEWCPTEVC-P--TC-RFI--C---GT-GGILR-------ENGR----ILFALDG------T--PLPVVFQK------KED

>pv_V7AIE7_26_200

--------VIDTNGEPVDN------------------D--EEYYIKPA------------ITENGGR---FTLIN-------R-ND-S---C---PLYVGL-E-----DTD--SAQ-GF--PVKF-TH-FAN---NIQDEDI-GVN-RDL-KVE---FVE-V---SS--TCV-Q---------------------TTEWRVG-ENDT----GSGR--------RLI-ISGEDDS----AGLY-GN---YFRIVETE--------------SAG-I-YNFQWCPMELC-S--SC-GFV--C---GN-AGILR-------ENGK----ILFALDG------A--SLPVVFQK---------

>Gm_I1KW54_26_200

--------VIDTNGEPVDN------------------D--DEYYIRPA------------ITDNGGR---FTLIN-------R-NG-S---C---PLYVGL-E-----NTD--TPL-GY--PVKF-TH-FAL---NVQDEDI-RVN-SDL-RIE---FVE-V---ST--TCV-Q---------------------STEWRVG-ENDT----RSGR--------RLI-ITGLDDN----FGSI-GN---YFRIVETQ--------------SVG-I-YNIEWCPMEIC-S--DC-GFV--C---ST-GGILR-------EDGR----IFFALDG------T--PLPVVFQK---------

>Gm_C6SY93_26_200

--------VIDTNGEPVDN------------------D--DEYYIRPA------------ITDNGGR---FTLIN-------R-NG-S---C---PLYVGL-E-----NTD--TPL-GY--PVKF-TH-FAL---NVQDEDI-RVN-SDL-RIE---FVE-V---ST--TCV-Q---------------------STEWRVG-ENDT----RGGR--------RLI-ITGLDDN----FGSI-GN---YFRIVETQ--------------SVG-I-YNIEWCPMEIC-S--DC-GFV--C---ST-GGILR-------EDGR----IFFALDG------T--PLPVVFQK---------

>Mt_G7ZV95_10_163

--------VIDTSGEPVED------------------D--EEYFIRPA------------ITGNGGG---SILVT-------I-NG-P---C---PLHVGL-G-----NSE--GTL-GL--AVKF-TP-FAPRH-DDDDDDV-RLN-RDL-RVT---F-Q-G---FT--GCG-Q---------------------STDWRLG-EKDA----TSGR--------RLI-VTGRDNG----AGSH------------------------------------------EAC-P--SC-KVQ--C---GT-VGVIR-------ENGK----ILLALDG------G--ALPVVFQK---------

>Mt_G7IVV4_27_202

--------VIDTSGEPVED------------------D--EEYFIRPA------------ITGNGGG---SILVT-------R-NA-P---C---PLHVGL-G-----NSE--GTL-GV--AVKF-TP-FAPRH-DDDDDDV-RLN-RDL-RVT---F-Q-G---FT--GCG-Q---------------------STDWRLG-EKDA----TSGR--------RLI-VTGRDNG----AGSH-GN---FFRIVQTQ--------------TGG-I-YNIQWCPTEAC-P--SC-KVQ--C---GT-VGVIR-------ENGK----ILLALDG------G--ALPVVFQK---------

>Mt_G7IVU9_27_202

--------VIDTSGEPVED------------------D--EEYFIRPA------------ITGNGGG---SILVT-------R-NG-P---C---PLHVGL-G-----NSE--GTL-GM--AVKF-TP-FAPRH-DDDDDDV-RLN-RDL-RVT---F-Q-G---FT--GCG-Q---------------------STDWRLG-EKDA----TSGR--------RLI-VTGRDNG----AGSH-GN---FFRIVQTQ--------------TGG-I-YNIQWCPTEAC-P--SC-KVQ--C---GT-VGVIR-------ENGK----ILLALDG------G--ALPVVFQK---------

>Mt_G7LCV7_27_199

--------VIDTSGEPVEN------------------D--EDYFIRPA------------ITGNGGS---LTLVT-------R-N--S---C---PFNVGL-------DPD--APQ-GF--AVLL-SP-FVS---NREEDEV-RLG-RDL-RVI---F-Q-A---GT--SCG-Q---------------------STEWRLG-ERDA----TTGR--------RFI-ITGRDDST---VGSY-GN---FFRIVQTP--------------SRG-I-FNIQWCPTEVC-P--SC-KFE--C---GT-VGIVR-------ENGK----ILLALDG------S--ALPVAFQK---------

>Mt_G7LCV1_29_203

--------VLDTVGEPVEG------------------D--EEYFIRPV------------ITNKGGR---STMVS-------R-NE-S---C---PLHVGL-E-----LTG--LGR-GL--VVKF-TP-FAP---HHDFDDV-RVN-RDL-RIT---F-Q-A---SS--SCV-Q---------------------STEWRLG-EKDT----KSGR--------RLI-ITGTDSAT---NGSY-GN---FFRIVETP--------------LEG-M-YNIQWCPTEVC-P--SC-KFE--C---GT-VDMLN-------ENGK----ILLALDG------G--PLPLVFQK---------

>Gm_I1LVB1_31_196

--------VLDTQGNPLEP------------------G--KDYYIKPA------------ITDVGGR---VTLLS-------R-NN-P---C---PLYVGQ-E-----NSD--AAE-GL--PLFF-TP-FA-----EEDDVV-KVN-RDF-KVT---F-S-A---AS--ICV-Q---------------------GTNWNLA-EKDS----ESGR--------RLI-AASGR-----------DD---YFRITETP--------------IKG-S-YYIGWCPTDVC-P--FC-RFD--C---GI-VGGLR-------ENGK----ILLALDG------N--VLPVVFEK---------

>Gm_C6SWI5_31_196

--------VLDTQGNPLEP------------------G--KDYYIKPA------------ITDVGGR---VTLLS-------R-NN-P---C---PLYVGQ-E-----NSD--AAE-GL--PLFF-TP-FA-----EEDDVV-KVN-RDF-KLT---F-S-A---AS--ICV-Q---------------------GTNWNLA-EKDS----ESGR--------RLI-AASGR-----------DD---YFRITETP--------------IKG-S-YYIGWCPTDVC-P--FC-RFD--C---GI-VGGLR-------ENGK----ILLALDG------N--VLPVVFEK---------

>Gm_K7M233_1_133

-------------------------------------G--KDYCIKPA------------IADVGGR---ATLLS-------RNNN-P---C---PLYVGQ-E-----NSD--AEE-GL--PIFF-TP-FA-----EEDDVV-KVN-REF-KVT---F-S-A---AS--ICV-Q---------------------GTNWNLA-EKDS----QSGR--------RLI-AASGS-----------DD---YLRITKTP--------------ING-T-YYIGWCPTYEC-P--LC-RFD--S---GI-VG---------------------------------------------------

>Gm_I1LVB0_31_195

--------VLDTEGHPLEP------------------G--RDYYITPA------------VTDIGGR---ATIVD---------NG-T---C---PLFVGQ-E-----NTF--VEE-SF--AVFF-TP-FA-----KEDDVV-KVN-RDF-QVA---F-S-A---AT--LCL-Q---------------------GTGWTLG-ERDT----ESGR--------RLI-VVGGV-----------GS---YFRISETQ--------------VKG-V-YNIGWCPIDVC-P--FC-KFD--C---GI-VGGLR-------ENKK----IFLALDG------N--VLPVVFER---------

>Mt_G8A245_32_197

--------ILDTKGHPLER------------------G--KEYYIKPA------------ITDSGGR---FTLID-------R-NG-S---C---PLYVGQ-E-----NTD--LGK-GL--PVIF-TP-FA-----KEDKVI-KDS-RDF-KVK---F-S-A---SS--ICV-Q---------------------STEWKLG-DRDT----KSGR--------RVI-IAGSD-----------GS---YFRIVKAE--------------FEG-V-YNIRFCPTDTC-S--FC-RFD--C---GF-VGGLR-------ENGK----ILLALDG------G--VLPVVFEC---------

>Mt_G8A259_32_198

--------VLDLTGRPLES------------------G--RKYYIRSD------------VSDFGGR---ITLVN-------K-NG-S---C---PLYVGQ-E-----TTD--FGQ-GL--SVIL-TP-LE-----NDDTVV-KVN-RDF-KVK---F-S-S---SS--SCG-Q---------------------STEWKLG-DRDN----RSGR--------RLI-IAGSD-----------GN---SFRILKISF--------GIEG-VIG-N-YNIRFCPSDT---------VN--C---GT-VGNLR-------ENGK----ILLALDD--R---N--VLRVGFER---------

>Ptri_B9N8J2_31_198

--------VLDTSGQPLET------------------G--VEYYILPG------------ITDVAGG---LTLVN-------R-NGIR---C---PFYVGQ-E-----PLASAEPN-GT--SVIF-TP-YT-----SGETII-RES-RDL-SVQ---F-Q-A---ST--ICI-Q---------------------STAWRVG-EEDP----ETGR--------RFI-VTAGDKS--------------YFRIDNNG----------------G-V-YNFSWCPTESC-P--NCARPR--C---GS-AGILI-------EDDK----RLLALDG------P--AFPFVFTR---------

>Ptri_U5FHA6_27_199

--------VLDADGQPLRS------------------G--VEYYVLPG------------VTDVGGG---LTLVD-------R-NG-S---C---PLYVGQ-E-----PLAPMVSR-GI--PVFF-TP-RV------GDTII-RES-RDF-TVE---F-S-G---AS--TCG-Q---------------------STAWMVG-EENP----ETTT--------RYV-VTGMEPR----PSST-LW---YFNIENNG---------------QG-V-YALRWCPNCLT-T--NCPRPA--C---ES-AGVID-------ENGK----RLLVLDG------S--AFPFIFRR---------

>Ptri_B9HEH9_8_180

--------VLDANGQPLRS------------------G--VEYYVLPA------------VTDVAGG---LILVN-------L-NN-G-SIC---PLFVGQ-E-----PLAPVVSR-GT--SVIF-TP-RV------ADTVI-RET-RDF-TVA---F-T-G---VT--ICA-Q---------------------STAWRVG-ERNP----ETRR--------RYI-LAETDPI----PSSN-AW---HFNIVKND---------------QG-L-YNFQWCPNCLT-R--VCPKPL--C---GD-AGIVV-------ENER----RLLVLDELAEHKLD-------------------

>Ptri_B9HEI2_27_196

--------VLDADGQPLRS------------------G--VEYYVLPA------------VTDVAGG---LTLVN-------L-NN-G-SIC---PLFVGQ-E-----PLAPVVSR-GT--SVIF-TP-RV------ADTVV-RET-RDF-TVA---F-T-G---VT--ICA-Q---------------------STAWMVG-EQNP----ETRR--------RYI-LAETDPN----PSSN-AW---HFNIVKND---------------QG-L-YNFQWCPNCLT-E--VCPRPL--C---GD-AGIVV-------ENER----RLLVLDGLRQI----------------------

>Ptri_B9GGD2_27_192

--------VLDTAGRPVRS------------------G--VEYYILPA------------ATDIAGG---LTLVA-------R-NG-S---C---PSFVGQ-E-----PLTPVVSQ-GL--PVVF-SP-YV-----AGETIV-RES-RSF-IIE---F-S-A---AS--TCV-S---------------------STKWNLA-ARDP----ATSR--------RNI-GIGRS-----------GS---YFMITKEN----------------N-L-YYLAFCPADTC-N--TC-RFD--C---GT-AGITI-------ENGK----RFLTLDG------P--VFPFRFRR---------

>Ptri_B9N9S5_26_195

--------VLDTDGNPLTR------------------G--VEYYVDPA------------ITDVAGG---LTLVA-------R-NG-S---C---PSYVGQ-V-----PIGPGSVE-DL--PVIF-TP-SN-----PGDTII-TET-TEF-TVA---F-S-A---SS--TCV-Q---------------------NTTWGIG-EEDP----ETRK--------RFI-VIGGEPT--------------VFDIDSDQ--------------AVG-P-YTIGWCPACLNPP--LCGRPR--C---GL-AGILE-------QNGT----RFLTLDG------P--AFPFRFRR---------

>Ptri_B9HEI1_26_195

--------VLDTDGNPVTR------------------G--VEYYVDPA------------VTDVAGG---LTLVT-------R-NG-S---C---PSYVGQ-V-----PIGSGNVQ-GL--PVIF-TP-RD-----SGETVI-TEN-TQF-TVA---F-S-A---AS--ICV-S---------------------DTTWGIG-EEDP----ETTR--------RLI-VIGDEPA--------------IFSISRNQ--------------APG-P-YTFGYCPECNTPP--PCGRPR--C---GI-AGILE-------QNGT----RFLTIDG------P--AFPFSFRR---------

>Ptri_B9HEI0_26_195

--------VLDAGGEPLRS------------------G--VEYLADPA------------VADVAGS---LTLVA-------R-NG-S---C---PFYVGQ-E-----SAR--SGRLGI--PVIF-TP-RN-----PQETII-TES-TEV-TVT---F-S-G---VS--TCV-R---------------------NTAWTIG-GEDS----QTRR--------RFV-VTGAEPS--------------YFQINSRN---------Q----LGG-S-YTFQGCPQCVDEP--DCGRAT--C---GT-AGILI-------QNGT----RFLVLDG------P--EFTFMFVR---------

>c20178_g1_i1_m_6160

--------VVDIDGNPVKV------------------G--GKYYVLPS------------LRGSGGG---LNLSR-I-----V-DK-SLKVC---PQDIVQ-D-----PKE--FNY-GR--AVEF-FP-AY------PNETI-LVN-NPI-NVK---F-V-PENETS--SCE-D---------------------FTVWKMD----------KKY--------KYV-VARGTLGA---LNRI-RN---WFRIVPYG---------K--------A-YRFVYCPS-LC---VPC-KIK--C---AD-LYISY-E-----NANV----RRLAAST--------------------------

>Mt_G7KMV2_1_159

---------MDTLGAPLRS------------------G--ESYQISVV------------VADHPGA---LTIGK-T-----D-DL-D---C----VYLVS-S-----QDD--SSH-GL--SVKF-HS-------------------TDI-------L---G---SP--ACV-E---------------------SAKWLVF-VDSE-----LDPL-P----IHYV-GIGGPENYPSHTEIF-DG---TFSIQRSE-------------LFPL-A-YTLNYCRM----DH---------C---SY-VGINK---VLIGNESD----RRLMLRQ---------AIVVVFEH---------

>Ptri_U5FFA8_34_201

--------VIDVNGNEVTP------------------D--ARYFIGAA------------SDDNTTT---LAVSA-T-----S-QI-I---C---NSDVTL-SS---------MSN-GL--PVTFSSP-VGE----SNDGVI-RED-SYL-NVN---F-D-A---A---TCR-MAGV------------------STMWKME-LRPT-----MRG--------FVV-TTGGVDG---------LN---RFKITKYE---------G----GNN-S-YQLSYCPI-SD-P--MC-ECSCAC---VP-LGNVV---------------DRLAPSTI--------PFPVVFEP---------

>Ptri_U7DYH7_34_201

--------VIDVNGNEVTA------------------D--ARYFIGAA------------SDDNTTT---LAVSA-T-----S-QI-I---C---NSDVIL-SS---------MSN-GL--PVTFSSP-VGE----SNDGVI-RED-SYL-NVN---F-D-A---A---TCR-MAGV------------------STMWKME-LRPT-----MRG--------FVV-TTGGVDG---------LN---RFKITKYE---------G----GNN-S-YQLSYCPI-SD-P--MC-ECSCAC---VP-LGNVV---------------DRLAPSTI--------PFPVVFEP---------

>Ptri_U7DWX9_2_122

----------------------------------------------------------------------------------------------------F-SP---------MSD-GL--PVIF-SP-VVE----SNDSVI-HED-SYL-NVD---F-D-A---A---TCR-MAGV------------------STMWKIE-LRPT-----ARG--------FVV-TTGGVAG---------LN---RFKITKYE---------G----GNN-L-YQLSYCPI-SE-P--IC-ECS--C---VP-LGQVV---------------NRLAPRTI--------PFPVVFV----------

>Ptri_U7DWL1_31_189

--------VIDVFGDEVRT------------------G--DRYIIGAA------------SNDFAVT---------S-----S-RI-I---C---NSDVMF-SP---------MSD-GL--PVIF-SP-VVE----SNDSVI-HED-SYL-NVD---F-D-A---A---TCR-MAGV------------------STMWKIE-LRPT-----ARG--------FVV-TTGGVAG---------LN---RFKITKYE---------G----GNN-L-YQLSYCPI-SE-P--IC-ECS--C---VP-LGQVV---------------NRLAPSTV--------PFPVVFV----------

>Ptri_D1KFL3_31_189

--------VIDVFGDEVRT------------------G--DRYIIGAA------------SNDFAVT---------S-----S-RI-I---C---NSDVMF-SP---------MSD-GL--PVIF-SP-VVE----SNDSVI-HED-SNL-NVD---F-D-A---A---TCR-MAGV------------------STMWKIE-LRPT-----ARG--------FVV-TTGGVAG---------LN---RFKITKYE---------G----GNN-L-YQLSYCPI-SE-P--IC-KCS--C---VP-LGKVV---------------NRLAPSTV--------PFPVVFV----------

>Ptri_D1KFJ5_31_189

--------VIDAFGDEVRT------------------G--DRYIIGAA------------SNDFAVT---------S-----S-RI-I---C---NSDVVF-SP---------MSD-GL--PVIF-SP-VVE----SNDSVI-HED-SNL-NVD---F-D-A---A---TCR-MAGV------------------STMWKIE-MRPT-----ARG--------FVV-TTGGVAG---------LN---RFKITKYE---------G----GNN-L-YQLSYCPI-SE-P--IC-KCS--C---VP-LGKVV---------------NRLAPSTV--------PFPVVFV----------

>Ptri_B9INR5_31_189

--------VIDVFGDEVRT------------------G--DRYIIGAA------------SNDFAVT---------S-----S-RI-I---C---NSDVVF-SP---------MSD-GL--PVIF-SP-VVE----SNDSVI-HED-SNL-NVD---F-D-A---A---TCR-MAGV------------------STMWKIE-LRPT-----ARG--------FVV-TTGGVAG---------LN---RFKITKYE---------G----GNN-L-YQLSYCPI-SE-P--IC-KCS--C---VP-LGKVV---------------NRLAPSTV--------PFPVVFV----------

>Ptri_D2TE94_3_140

-------------------------------------------------------------NDFAVT---------S-----S-RI-I---C---NSDVVF-SP---------MSD-GL--PVIF-SP-VVE----SNDSVI-HED-SNL-NVD---F-D-A---A---TCR-MAGV------------------STMWKIE-LRPT-----ARG--------FVV-TTGGVAG---------LN---RFKITKYE---------G----GNN-L-YQLSYCPI-SE-P--IC-KCS--C---VP-LGKVV---------------NRLAPSTV--------PFPVVFV----------

>Ptri_D4IH08_2_137

---------------------------------------------------------------FAVT---------S-----S-RI-I---C---NSDVVF-SP---------MSD-GL--PVIF-SP-VVE----SNDSVI-HED-SNL-NVD---F-D-A---A---TCR-MAGV------------------STMWKIE-LRPT-----ARG--------FVV-TTGGVAG---------LN---RFKITKYE---------G----GNN-L-YQLSYCPI-SE-P--IC-KCS--C---VP-LGKVV---------------NRLAPSTV--------PFPVVFV----------

>Ptri_D1KFK5_31_189

--------VIDAFGDEVRT------------------G--DRYIIGAA------------SNDFAVT---------S-----S-SI-I---C---NSDVFL-SP---------MSH-GL--PVIF-SP-VVE----SNDSVI-HED-SNL-NVD---F-D-A---A---TCR-MAGV------------------STMWKIE-MRPT-----ARG--------FVV-TTGGVAG---------LN---RFKITKYE---------G----GNN-L-YQLSYCPI-SE-P--IC-KCS--C---VP-LGKVV---------------NRLAPSTV--------PFPVVFV----------

>Ptri_U7DYP2_33_185

--------VLDVFGHEVQA------------------G--ARYLIVAP------------STDNTTT---LAVTA-T-----S-KI-I---C---NSDVIL-ST---------LNE-SL--PITF-SP-AIK----SNDGVI-REG-SYL-NVN---F-D-A---P---SCR-MGGV------------------TTMWMIE----------SEG--------LIV-TTGGVDR---------LN---RFKITKYE---------G----DNS-F-YQLSFCPM-SE-P--FC-ECS--C---VP-VGVNKMN-------------KSLTSS---------------------------

>Ptri_B9HXG0_33_193

--------VLDVFGHEVQA------------------G--ARYLIVAP------------STDNTTT---LAVTA-T-----S-KI-I---C---NSDVIL-ST---------LNE-SL--PITF-SP-AIK----SNDGVI-REG-SYL-NVN---F-D-A---P---SCR-MGGV------------------TTMWMIE----------SEG--------LIV-TTGGVDR---------LN---RFKITKYE---------G----DNS-F-YQLSFCPM-SE-P--FC-ECS--C---VP-VGVNS-D-------------KHLAPNV------G--PLLVMFE----------

>Ptri_B9HXF8_33_198

--------VLDVFGHEVQA------------------G--ARYLIVAP------------STDNTTT---LAVTI-N-----G-QV-L---C---NSDVIL-ST---------LNE-SL--PITF-SP-VIQ----STDSVI-REG-THL-NVN---F-A-G---PIA-MCA-MAGV------------------TPMWKIR-FSTT-----LKG--------YIV-TT-VVDR---------LN---RFKITKYE---------G----DNS-F-YQLSFCPM-SE-P--FC-ECS--C---VP-VSVNG-D-------------KNLVPGA------G--PLLVMFE----------

>Ptri_D1KFI7_33_199

--------VLDVFGHEVQA------------------G--ARYLIVAP------------STDNTTT---LAVTI-N-----G-QV-L---C---NSDVIL-ST---------LNE-SL--PITF-SP-VIQ----STDSVI-REG-THL-NVN---F-A-G---PSA-MCL-MGGV------------------TPMWKIR-FSTT-----LKG--------YIV-TTGGVDR---------LN---RFKITKYE---------G----DNS-F-YQLSFCPM-SE-P--FC-ECS--C---VP-VGVNG-D-------------KNLVPGA------G--PLLVMFE----------

>Ptri_U5FXJ2_33_199

--------VLDVFGHEVQA------------------G--ARYLIVAP------------STDNTTT---LAVTI-N-----G-QV-L---C---NSDVIL-ST---------LNE-SL--PITF-SP-VIQ----STDSVI-REG-THL-NVN---F-A-G---PSA-MCL-MGGV------------------TPMWKIR-FSTT-----LKG--------YTV-TTGGVDR---------LN---RFKITKYE---------G----DNS-F-YQLSFCPM-SE-P--FC-ECS--C---VP-VGVNG-D-------------KNLVPGA------G--PLLVMFE----------

>Ptri_U5G0D6_33_199

--------VLDVFGHEVQA------------------G--ARYLIVAP------------STDNTTT---LAVTI-N-----G-QV-L---C---NSDVIL-ST---------LNE-SL--PITF-SP-VIQ----STDSVI-REG-THL-NVN---F-A-G---PSA-MCL-MGGV------------------TPMWKIG-FSTT-----LKG--------YIV-TTGGVDR---------LN---RFKITKYE---------G----DNS-F-YQLSFCPM-SE-P--FC-ECS--C---VP-VGVNG-D-------------KNLVPGA------G--PLLVMFE----------

>Ptri_B9HXF9_33_199

--------VLDVFGHEVQA------------------G--ARYLIVAP------------STDNTTT---LAVTI-N-----G-QV-L---C---NSDVIL-ST---------LNE-SL--PITF-SP-VMQ----STDSVI-REG-THL-NVN---F-A-G---PIA-MCA-MAGV------------------TPMWKIR-FSTT-----LKG--------YIV-TTGGVDR---------LN---RFKITKYE---------G----DNS-F-YQLSFCPM-SE-P--FC-ECS--C---VP-VGVNG-D-------------KNLVPGA------G--PLLVMFE----------

>Ptri_D2TE96_1_137

-----------------------------------------------------------------------VVNA-T-----I-NP-I---C---NSDVIL-ST---------GIE-GL--PVTF-SP-VIN----STDGVI-REG-TLI-TVS---F-D-A---S---TCG-MAGV------------------TPMWKIG-FNST-----AKG--------YIV-TTGGVDR---------LN---LFKITKFE---------S----DSS-F-YQLSYCPN-SE-P--FC-ECP--C---VP-VGANS-D-------------KYLAPNV------S--YADFRFK----------

>Ptri_D4IH10_2_140

-------------------F--------------------------------------------------RVVNA-T-----I-NP-I---C---NSDVIL-ST---------GIE-GL--PVTF-SP-VIN----STDGVI-REG-TLI-TVS---F-D-A---S---TCG-MAGV------------------TPMWKIG-FNST-----AKG--------YIV-TTGGVDR---------LN---LFKITKFE---------S----DSS-F-YQLSYCPN-SE-P--FC-ECP--C---VP-VGANS-D-------------KYLAPNV------S--YADFRFK----------

>Ptri_B9NKX9_33_115

--------VLDFNGHEVQA------------------G--ASYLI-----------------DEEDI---LVVNA-T-----I-NP-I---C---NSDVIL-ST---------GTE-GL--PVRF-SP-VIN----STDGVI-REG-TLI-TVS---F-H-A---N---TCNTTAGV------------------T-------------------------------------------------------------------------------------------------------------------------------------------------------------------

>Ptri_B9GZK4_33_197

--------VVDSNDNEVLL------------------G--LDYYIAVT------------SPFDG-----MSLAM---------EA-S---C---PPPVVH-RN---------NLS-LL--PIKF-SS-VVD----SNDNVV-RED-TSL-NLE---F-N-VELNNS--DCN----V------------------PTIWKVE-FNAS-----MQQ--------WLV-MIGGDRS---------HN---RFQIAKAC---------P---YRKY-F-YQLRYCPV-LG----SI-QFP--C---VT-VRSLF-------KNGL----NYLALNG------D--PIAIVL----G------

>Ptri_B9IQN4_32_207

--------VLDVKGLGLLP------------------G--VPYYMISSE-----------WPIVGGV---VSLGN-D-----I-NG-T---C---PLDVIL-L-----ENF--CVT-GT--PVTF-TI-ASG----DQELFI-TDS-TDL-YIS---F-D-S---TS--NCT-NE--------------------TMVWMHE-SSNS-----SST--------ELL-TIGGV------EGDV-NT---LFRIVNVG---------G---SFVS-N-YKLVAYKL-SSYDL----ALT--A---SD-VGAVF-DF----TTGI----RYLALTE------P--PLIVGFQV---------

>Ptri_U7DWX7_15_144

--------IRDIDGDELFG------------------G--QKYYAATL------------YTAPAPG---------------R--------------DVVL------LRNP--SDR-GT--PVTA-ST-KC-----ETGTAI-TDS-TSV-FIS---F-N-T---TS--TCA-E---------------------STEWRFE----------ENG--------GFM-TIG--------EGKT-KT---EFRIINKG-----------WR-FGN-G-YKL---------------------------LAMFFSDF----GFGT----GYIGVD---------------------------

>At_Q9C7S6_27_200

--------VKDTAGNPLNT------------------R--EQYFIQPVK----------TESKNGGG---LVPAAIT-----V-LP-F---C---PLGITQ-T-----LLP--YQP-GL--PVSF-VL-AL-----GVGSTV-MTS-SAV-NIE---F-K-S-NIWP--FCK-EF--------------------SKFWEVD-DSSS----APKE--------PSI-LIGGKMGDR-------NS---SFKIEKAG---------E--GARAN-V-YKLT-------------------T-FYGT-VGAIP---------GVWLSAPQLIITK--DTA-K--TLLVKFKK---------

>At_Q9M8Y9_29_197

--------VYDGEGDQVKP------------------N--VPYYISFM------------TSDYNMWICRKKWRS-N-----D-PN-S---CPQQPLMVTHPN----------MAA-PT--PVMF-VL-SN-----KSETVV-RES-AKL-KIK---F-V-D---PR--PCG-E---------------------SGFWRVV-QRTS----SEGE----------V-VLNGSES----TSDN-AS---TFAIEQTN----------------E-Y-YKFTFGDG----P-----DYL------TT-ISLSN-DY----------PIYRLLSKKF-----S-GEMEIYFYK---------

>At_Q9M8Y8_71_237

--------VYDGEGDPVKP------------------N--VPYYISFM------------TSDYNMWICRMQYGS-T-----D-PN-S---CPQQPLMVTHPN----------LAA-PT--PVMF-VL-AN------KSDVV-RES-AKL-KIK---F-V-G---PR--QCG-K---------------------SGFWKVV-QRNS----SEGE----------V-FLNGSKS----MSHN-DS---TFAIHKTN----------------E-Y-YKFTFGDG----------DYP------TT-ISMTN-DY----------PIYRLLSKRL-----S-GEMEIYFYK---------

>At_Q0WNE5_30_196

--------VYDGEGDPVKP------------------N--VPYYISFM------------TSDYNMWICRMQYGS-T-----D-PN-S---CPQQPLMVTHPN----------LAA-PT--PVMF-VL-AN------KSDVV-RES-AKL-KIK---F-V-G---PR--QCG-K---------------------SGFWKVV-QRNS----SEGE----------V-FLNGSKS----MSHN-DS---TFAIHKTN----------------E-Y-YKFTFGDG----------DYP------TT-ISMTN-DY----------PIYRLLSKRL-----S-GEMEIYFYK---------

>At_Q7Y212_38_204

--------VYDGEGDPVKP------------------N--VPYYISFM------------TSDYNMWICRMQYGS-T-----D-PN-S---CPQQPLMVTHPN----------LAA-PT--PVMF-VL-AN------KSDVV-RES-AKL-KIK---F-V-G---PR--QCG-K---------------------SGFWKVV-QRNS----SEGE----------V-FLNGSKS----MSHN-DS---TFAIHKTN----------------E-Y-YKFTFGDG----------DYP------TT-ISMTN-DY----------PIYRLLSKRL-----S-GEMEIYFYK---------
