# Supplementary material for: Transcriptome sequencing and marker development in winged bean (Psophocarpus tetragonolobus; Leguminosae)
Source: Sci Rep. 2016 Jun 30;6:29070. doi: 10.1038/srep29070 (PMC4928180; doi:10.1038/srep29070)
Supplement: Supplementary File 7 [file srep29070-s7.doc]

**Transcriptome sequencing and marker development in winged bean (*Psophocarpus tetragonolobus*; Leguminosae)**

Mohammad Vatanparast^1^, Prateek Shetty^2^, Ratan Chopra^3^, Jeff J. Doyle^4^, N. Sathyanarayana^5*^ and Ashley N. Egan^1*^

^1^US National Herbarium (US), Department of Botany, Smithsonian Institution-NMNH, 10^th^ and Constitution Ave, Washington DC, 20013, USA.

^2^Department of Plant Biology, Michigan State University, 612 Wilson Road, Room 166, East Lansing, MI, 48824, USA.

^3^United States Department of Agriculture, Agriculture Research Service, 3810 4^th^ St., Lubbock, TX, 79415, USA.

^4^Section of Plant Breeding & Genetics, School of Integrative Plant Science, Cornell University, 412 Mann Library, Ithaca, NY, 14853, USA.

^5^Department of Botany, Sikkim University, 5th Mile, Tadong, Gangtok, Sikkim, 737102, India.

*Email Addresses:*

Mohammad Vatanparast: [Vatanparastm@si.edu](mailto:Vatanparastm@si.edu)

Prateek Shetty: [prateekshettys@gmail.com](mailto:prateekshettys@gmail.com)

Ratan Chopra: [Ratan.Chopra@ARS.USDA.GOV](mailto:Ratan.Chopra@ARS.USDA.GOV)

Jeff J. Doyle: [jjd5@cornell.edu](mailto:jjd5@cornell.edu)

N. Sathyanarayana: [nsathyanarayana@cus.ac.in](mailto:nsathyanarayana@cus.ac.in)

Ashley N. Egan: [egana@si.edu](mailto:egana@si.edu)

**Corresponding authors:*

Ashley N. Egan (primary) N. Sathyanarayana (secondary)

**Supplementary File 7. Kunitz trypsin inhibitor tree file.** Nexus tree file for kunitz trypsin inhibitor amino acid gene tree illustrated in Figure 7.

#NEXUS

begin taxa;

dimensions ntax=262;

taxlabels

pv_V7C568_51_224

pv_V7C681_32_205

pv_T2DNW0_32_205

pv_V7C4W6_32_204

Gm_B1ACD5_27_199

Gm_Q9LLX2_1_156

Gm_C6SVQ6_27_199

isotig08968_m_12187

pv_V7C2P7_85_148

pv_V7C2N3_57_172

pv_V7C2M9_33_202

Mt_G7KMU7_31_203

Mt_Q8LK19_31_203

Mt_G7KMU3_32_207

pv_V7C2P7_32_94

Mt_G7KMI6_28_201

Mt_G7KP86_30_147

Mt_G7KP95_27_92

Mt_G7KMG8_25_193

Mt_G7KMH6_26_193

Mt_I3SQY8_29_203

Mt_G7KKD7_29_201

Mt_I3T969_29_201

Mt_B7FN38_29_201

Mt_I3SFS0_30_207

pv_V7C4Y1_31_199

pv_V7C689_18_139

pv_V7C2Q1_31_203

pv_V7C2Q5_72_185

pv_V7C583_24_180

pv_V7C2Q7_31_199

pv_V7BF35_18_203

pv_V7C2N7_32_201

pv_V7C685_31_199

pv_V7C4X8_34_211

Mt_G7KJK4_32_205

Mt_G7KJK1_32_204

Gm_K7LE45_13_126

Mt_G7KMV4_35_209

Mt_G7KMU9_213_383

Mt_I3SQ24_31_201

Mt_G7KMU9_10_134

Gm_I1MQA3_32_231

Gm_C6TB12_32_231

Gm_I1MQA2_32_224

Gm_C6SWZ5_32_224

pv_V7C6J1_31_204

Gm_K7LE99_72_243

Gm_C6T3V8_32_203

Mt_I3RZB7_26_199

Mt_G7KKP4_26_204

Mt_G7KKP1_26_202

Gm_K7LCR5_32_107

Mt_G7KKN9_1_90

Ptri_B9H290_33_207

Ptri_D1KFL1_33_207

Ptri_U5FH02_28_203

Ptri_U5FJJ3_32_205

Ptri_U5FHI8_32_205

Ptri_U5FKJ0_32_205

Ptri_B9H293_32_206

At_Q9LMU2_29_194

Ptri_B9H291_30_204

Ptri_D4IH09_1_107

Ptri_D2TE95_1_148

pv_V7C2X4_1_67

pv_V7C576_20_168

pv_V7C582_1_63

Gm_I1L3T2_30_198

Gm_C6T5E3_30_198

Gm_K7LE94_1_141

Gm_I1L3T6_30_198

Gm_I1L3T4_30_198

Gm_I1MQG2_30_198

Gm_I1MQG3_27_196

Gm_C6SY46_30_212

Mt_I3SMI6_30_211

Mt_G7KKR0_30_211

Gm_C6T2D3_31_212

Mt_I3S253_30_211

Mt_G7KKQ9_30_211

Mt_G7KKQ7_31_198

Gm_C6TLR5_31_208

Mt_Q6ISX8_31_212

Mt_G7KKP6_31_212

Gm_I1L3U2_70_254

Gm_I1L3U0_30_214

Gm_K7LE98_2_98

Gm_I1L3T9_32_210

Gm_K7K398_1_89

Mt_G7KKQ6_27_203

Gm_K7LE97_30_214

Mt_G7KRA4_29_201

At_Q93Y29_30_204

At_Q9CAT9_23_197

At_Q8RXD5_30_204

At_Q8H190_30_204

At_Q39091_28_191

At_Q9FX28_32_202

Gm_I1JLM5_74_131

pv_V7B6G3_54_177

Gm_I1N2Q3_34_203

Gm_C6T586_28_198

Gm_Q9XIS8_27_197

Gm_B1ACD0_28_198

Gm_Q39869_28_199

Gm_I1KYW5_27_194

Gm_C6T280_27_194

Gm_I1KYX2_26_190

Gm_I1KYX0_26_190

Gm_Q94IA1_28_200

Gm_Q39898_28_200

Gm_P01070_27_199

Gm_C6T488_28_200

Gm_Q76B18_28_200

Gm_Q9ATY0_28_145

Gm_Q9LD16_27_199

Gm_P01071_3_175

Gm_Q39899_28_200

Gm_Q9ATY1_28_199

Gm_P25272_28_197

Gm_C6SWW4_28_197

Gm_P25273_28_198

Gm_C6T0C7_28_196

Gm_I1KYX1_28_196

Gm_I1KYW9_28_198

Gm_C6T696_28_184

Gm_C6T3I9_28_196

Gm_I1KYW8_28_205

Gm_C6T1F1_28_205

Gm_C6T261_26_194

Gm_I1J6C0_47_215

isotig09349_m_12644

c26779_g2_i1_m_18736

isotig09582_m_12928

c26779_g1_i1_m_18735

isotig01407_m_2393

isotig01408_m_2395

isotig01460_m_2516

c29416_g2_i1_m_29755

isotig09071_m_12312

c29416_g3_i1_m_29756

isotig09219_m_12494

c29416_g1_i1_m_29754

contig00104_m_14

c28083_g1_i2_m_23337

contig00111_m_17

c28083_g1_i1_m_23336

isotig08256_m_11234

isotig00406_m_765

c6564_g1_i1_m_1144

Gm_I1KYW3_27_197

Gm_C6TB67_27_197

contig00100_m_13

c7831_g1_i1_m_1303

c22388_g1_i1_m_8774

Ptet_P25700_3_176

Ptet_A6XBI5_3_176

Ptet_Q7M1S6_3_176

Ptet_B4F6G4_3_168

Ptet_P32877_3_168

Ptet_P10821_3_168

Ptet_Q4U4G0_28_198

Ptet_Q43721_28_198

Ptet_P10822_28_198

Ptet_Q43708_28_198

isotig09642_m_13008

c17145_g1_i1_m_3533

Gm_C6K8D0_31_222

Gm_C6T599_31_222

Gm_C6SVL0_27_194

isotig07854_m_10657

c28968_g2_i1_m_27305

isotig08014_m_10899

isotig08832_m_12014

c28968_g3_i1_m_27307

isotig09597_m_12947

isotig08069_m_10981

c28968_g1_i1_m_27304

isotig10039_m_13496

Gm_C6SYK2_29_201

Gm_I1KYX3_29_201

isotig06462_m_8385

isotig02703_m_4313

c31193_g1_i1_m_41593

isotig08164_m_11114

Ptet_Q43325_23_185

c23070_g1_i1_m_10023

isotig08531_m_11611

c23070_g2_i1_m_10024

Ptet_P15465_5_171

isotig09482_m_12803

c25292_g1_i1_m_14709

c25292_g2_i1_m_14710

isotig12801_m_16475

Gm_I1JLM5_9_85

pv_V7C570_33_198

Gm_K7MIV6_34_199

Gm_B1ACD3_34_199

isotig08485_m_11543

Mt_I3SZS6_29_196

Mt_G7JAF9_29_196

Mt_G7JAG3_29_198

Mt_G7JAF9_239_408

pv_V7AIA7_30_204

pv_V7AJ80_30_204

Gm_I1KW53_31_205

Gm_C4XVM5_31_205

Gm_I1MI59_26_203

isotig10611_m_14171

pv_V7AIE7_26_200

Gm_I1KW54_26_200

Gm_C6SY93_26_200

Mt_G7ZV95_10_163

Mt_G7IVV4_27_202

Mt_G7IVU9_27_202

Mt_G7LCV7_27_199

Mt_G7LCV1_29_203

Gm_I1LVB1_31_196

Gm_C6SWI5_31_196

Gm_K7M233_1_133

Gm_I1LVB0_31_195

Mt_G8A245_32_197

Mt_G8A259_32_198

Ptri_B9N8J2_31_198

Ptri_U5FHA6_27_199

Ptri_B9HEH9_8_180

Ptri_B9HEI2_27_196

Ptri_B9GGD2_27_192

Ptri_B9N9S5_26_195

Ptri_B9HEI1_26_195

Ptri_B9HEI0_26_195

c20178_g1_i1_m_6160

Mt_G7KMV2_1_159

Ptri_U5FFA8_34_201

Ptri_U7DYH7_34_201

Ptri_U7DWX9_2_122

Ptri_U7DWL1_31_189

Ptri_D1KFL3_31_189

Ptri_D1KFJ5_31_189

Ptri_B9INR5_31_189

Ptri_D2TE94_3_140

Ptri_D4IH08_2_137

Ptri_D1KFK5_31_189

Ptri_U7DYP2_33_185

Ptri_B9HXG0_33_193

Ptri_B9HXF8_33_198

Ptri_D1KFI7_33_199

Ptri_U5FXJ2_33_199

Ptri_U5G0D6_33_199

Ptri_B9HXF9_33_199

Ptri_D2TE96_1_137

Ptri_D4IH10_2_140

Ptri_B9NKX9_33_115

Ptri_B9GZK4_33_197

Ptri_B9IQN4_32_207

Ptri_U7DWX7_15_144

At_Q9C7S6_27_200

At_Q9M8Y9_29_197

At_Q9M8Y8_71_237

At_Q0WNE5_30_196

At_Q7Y212_38_204

;

end;

begin trees;

translate

1 pv_V7C568_51_224,

2 pv_V7C681_32_205,

3 pv_T2DNW0_32_205,

4 pv_V7C4W6_32_204,

5 Gm_B1ACD5_27_199,

6 Gm_Q9LLX2_1_156,

7 Gm_C6SVQ6_27_199,

8 isotig08968_m_12187,

9 pv_V7C2P7_85_148,

10 pv_V7C2N3_57_172,

11 pv_V7C2M9_33_202,

12 Mt_G7KMU7_31_203,

13 Mt_Q8LK19_31_203,

14 Mt_G7KMU3_32_207,

15 pv_V7C2P7_32_94,

16 Mt_G7KMI6_28_201,

17 Mt_G7KP86_30_147,

18 Mt_G7KP95_27_92,

19 Mt_G7KMG8_25_193,

20 Mt_G7KMH6_26_193,

21 Mt_I3SQY8_29_203,

22 Mt_G7KKD7_29_201,

23 Mt_I3T969_29_201,

24 Mt_B7FN38_29_201,

25 Mt_I3SFS0_30_207,

26 pv_V7C4Y1_31_199,

27 pv_V7C689_18_139,

28 pv_V7C2Q1_31_203,

29 pv_V7C2Q5_72_185,

30 pv_V7C583_24_180,

31 pv_V7C2Q7_31_199,

32 pv_V7BF35_18_203,

33 pv_V7C2N7_32_201,

34 pv_V7C685_31_199,

35 pv_V7C4X8_34_211,

36 Mt_G7KJK4_32_205,

37 Mt_G7KJK1_32_204,

38 Gm_K7LE45_13_126,

39 Mt_G7KMV4_35_209,

40 Mt_G7KMU9_213_383,

41 Mt_I3SQ24_31_201,

42 Mt_G7KMU9_10_134,

43 Gm_I1MQA3_32_231,

44 Gm_C6TB12_32_231,

45 Gm_I1MQA2_32_224,

46 Gm_C6SWZ5_32_224,

47 pv_V7C6J1_31_204,

48 Gm_K7LE99_72_243,

49 Gm_C6T3V8_32_203,

50 Mt_I3RZB7_26_199,

51 Mt_G7KKP4_26_204,

52 Mt_G7KKP1_26_202,

53 Gm_K7LCR5_32_107,

54 Mt_G7KKN9_1_90,

55 Ptri_B9H290_33_207,

56 Ptri_D1KFL1_33_207,

57 Ptri_U5FH02_28_203,

58 Ptri_U5FJJ3_32_205,

59 Ptri_U5FHI8_32_205,

60 Ptri_U5FKJ0_32_205,

61 Ptri_B9H293_32_206,

62 At_Q9LMU2_29_194,

63 Ptri_B9H291_30_204,

64 Ptri_D4IH09_1_107,

65 Ptri_D2TE95_1_148,

66 pv_V7C2X4_1_67,

67 pv_V7C576_20_168,

68 pv_V7C582_1_63,

69 Gm_I1L3T2_30_198,

70 Gm_C6T5E3_30_198,

71 Gm_K7LE94_1_141,

72 Gm_I1L3T6_30_198,

73 Gm_I1L3T4_30_198,

74 Gm_I1MQG2_30_198,

75 Gm_I1MQG3_27_196,

76 Gm_C6SY46_30_212,

77 Mt_I3SMI6_30_211,

78 Mt_G7KKR0_30_211,

79 Gm_C6T2D3_31_212,

80 Mt_I3S253_30_211,

81 Mt_G7KKQ9_30_211,

82 Mt_G7KKQ7_31_198,

83 Gm_C6TLR5_31_208,

84 Mt_Q6ISX8_31_212,

85 Mt_G7KKP6_31_212,

86 Gm_I1L3U2_70_254,

87 Gm_I1L3U0_30_214,

88 Gm_K7LE98_2_98,

89 Gm_I1L3T9_32_210,

90 Gm_K7K398_1_89,

91 Mt_G7KKQ6_27_203,

92 Gm_K7LE97_30_214,

93 Mt_G7KRA4_29_201,

94 At_Q93Y29_30_204,

95 At_Q9CAT9_23_197,

96 At_Q8RXD5_30_204,

97 At_Q8H190_30_204,

98 At_Q39091_28_191,

99 At_Q9FX28_32_202,

100 Gm_I1JLM5_74_131,

101 pv_V7B6G3_54_177,

102 Gm_I1N2Q3_34_203,

103 Gm_C6T586_28_198,

104 Gm_Q9XIS8_27_197,

105 Gm_B1ACD0_28_198,

106 Gm_Q39869_28_199,

107 Gm_I1KYW5_27_194,

108 Gm_C6T280_27_194,

109 Gm_I1KYX2_26_190,

110 Gm_I1KYX0_26_190,

111 Gm_Q94IA1_28_200,

112 Gm_Q39898_28_200,

113 Gm_P01070_27_199,

114 Gm_C6T488_28_200,

115 Gm_Q76B18_28_200,

116 Gm_Q9ATY0_28_145,

117 Gm_Q9LD16_27_199,

118 Gm_P01071_3_175,

119 Gm_Q39899_28_200,

120 Gm_Q9ATY1_28_199,

121 Gm_P25272_28_197,

122 Gm_C6SWW4_28_197,

123 Gm_P25273_28_198,

124 Gm_C6T0C7_28_196,

125 Gm_I1KYX1_28_196,

126 Gm_I1KYW9_28_198,

127 Gm_C6T696_28_184,

128 Gm_C6T3I9_28_196,

129 Gm_I1KYW8_28_205,

130 Gm_C6T1F1_28_205,

131 Gm_C6T261_26_194,

132 Gm_I1J6C0_47_215,

133 isotig09349_m_12644,

134 c26779_g2_i1_m_18736,

135 isotig09582_m_12928,

136 c26779_g1_i1_m_18735,

137 isotig01407_m_2393,

138 isotig01408_m_2395,

139 isotig01460_m_2516,

140 c29416_g2_i1_m_29755,

141 isotig09071_m_12312,

142 c29416_g3_i1_m_29756,

143 isotig09219_m_12494,

144 c29416_g1_i1_m_29754,

145 contig00104_m_14,

146 c28083_g1_i2_m_23337,

147 contig00111_m_17,

148 c28083_g1_i1_m_23336,

149 isotig08256_m_11234,

150 isotig00406_m_765,

151 c6564_g1_i1_m_1144,

152 Gm_I1KYW3_27_197,

153 Gm_C6TB67_27_197,

154 contig00100_m_13,

155 c7831_g1_i1_m_1303,

156 c22388_g1_i1_m_8774,

157 Ptet_P25700_3_176,

158 Ptet_A6XBI5_3_176,

159 Ptet_Q7M1S6_3_176,

160 Ptet_B4F6G4_3_168,

161 Ptet_P32877_3_168,

162 Ptet_P10821_3_168,

163 Ptet_Q4U4G0_28_198,

164 Ptet_Q43721_28_198,

165 Ptet_P10822_28_198,

166 Ptet_Q43708_28_198,

167 isotig09642_m_13008,

168 c17145_g1_i1_m_3533,

169 Gm_C6K8D0_31_222,

170 Gm_C6T599_31_222,

171 Gm_C6SVL0_27_194,

172 isotig07854_m_10657,

173 c28968_g2_i1_m_27305,

174 isotig08014_m_10899,

175 isotig08832_m_12014,

176 c28968_g3_i1_m_27307,

177 isotig09597_m_12947,

178 isotig08069_m_10981,

179 c28968_g1_i1_m_27304,

180 isotig10039_m_13496,

181 Gm_C6SYK2_29_201,

182 Gm_I1KYX3_29_201,

183 isotig06462_m_8385,

184 isotig02703_m_4313,

185 c31193_g1_i1_m_41593,

186 isotig08164_m_11114,

187 Ptet_Q43325_23_185,

188 c23070_g1_i1_m_10023,

189 isotig08531_m_11611,

190 c23070_g2_i1_m_10024,

191 Ptet_P15465_5_171,

192 isotig09482_m_12803,

193 c25292_g1_i1_m_14709,

194 c25292_g2_i1_m_14710,

195 isotig12801_m_16475,

196 Gm_I1JLM5_9_85,

197 pv_V7C570_33_198,

198 Gm_K7MIV6_34_199,

199 Gm_B1ACD3_34_199,

200 isotig08485_m_11543,

201 Mt_I3SZS6_29_196,

202 Mt_G7JAF9_29_196,

203 Mt_G7JAG3_29_198,

204 Mt_G7JAF9_239_408,

205 pv_V7AIA7_30_204,

206 pv_V7AJ80_30_204,

207 Gm_I1KW53_31_205,

208 Gm_C4XVM5_31_205,

209 Gm_I1MI59_26_203,

210 isotig10611_m_14171,

211 pv_V7AIE7_26_200,

212 Gm_I1KW54_26_200,

213 Gm_C6SY93_26_200,

214 Mt_G7ZV95_10_163,

215 Mt_G7IVV4_27_202,

216 Mt_G7IVU9_27_202,

217 Mt_G7LCV7_27_199,

218 Mt_G7LCV1_29_203,

219 Gm_I1LVB1_31_196,

220 Gm_C6SWI5_31_196,

221 Gm_K7M233_1_133,

222 Gm_I1LVB0_31_195,

223 Mt_G8A245_32_197,

224 Mt_G8A259_32_198,

225 Ptri_B9N8J2_31_198,

226 Ptri_U5FHA6_27_199,

227 Ptri_B9HEH9_8_180,

228 Ptri_B9HEI2_27_196,

229 Ptri_B9GGD2_27_192,

230 Ptri_B9N9S5_26_195,

231 Ptri_B9HEI1_26_195,

232 Ptri_B9HEI0_26_195,

233 c20178_g1_i1_m_6160,

234 Mt_G7KMV2_1_159,

235 Ptri_U5FFA8_34_201,

236 Ptri_U7DYH7_34_201,

237 Ptri_U7DWX9_2_122,

238 Ptri_U7DWL1_31_189,

239 Ptri_D1KFL3_31_189,

240 Ptri_D1KFJ5_31_189,

241 Ptri_B9INR5_31_189,

242 Ptri_D2TE94_3_140,

243 Ptri_D4IH08_2_137,

244 Ptri_D1KFK5_31_189,

245 Ptri_U7DYP2_33_185,

246 Ptri_B9HXG0_33_193,

247 Ptri_B9HXF8_33_198,

248 Ptri_D1KFI7_33_199,

249 Ptri_U5FXJ2_33_199,

250 Ptri_U5G0D6_33_199,

251 Ptri_B9HXF9_33_199,

252 Ptri_D2TE96_1_137,

253 Ptri_D4IH10_2_140,

254 Ptri_B9NKX9_33_115,

255 Ptri_B9GZK4_33_197,

256 Ptri_B9IQN4_32_207,

257 Ptri_U7DWX7_15_144,

258 At_Q9C7S6_27_200,

259 At_Q9M8Y9_29_197,

260 At_Q9M8Y8_71_237,

261 At_Q0WNE5_30_196,

262 At_Q7Y212_38_204

;

tree con_50_majrule = [&U] (1[&prob=1.00000000e+00,prob_stddev=0.00000000e+00,prob_range={1.00000000e+00,1.00000000e+00},prob(percent)="100",prob+-sd="100+-0"]:7.492319e-02[&length_mean=7.72733763e-02,length_median=7.49231900e-02,length_95%HPD={3.63762600e-02,1.24479800e-01}],((4[&prob=1.00000000e+00,prob_stddev=0.00000000e+00,prob_range={1.00000000e+00,1.00000000e+00},prob(percent)="100",prob+-sd="100+-0"]:2.997606e-02[&length_mean=3.17726682e-02,length_median=2.99760600e-02,length_95%HPD={6.00517700e-03,5.99744600e-02}],(((((5[&prob=1.00000000e+00,prob_stddev=0.00000000e+00,prob_range={1.00000000e+00,1.00000000e+00},prob(percent)="100",prob+-sd="100+-0"]:3.330973e-03[&length_mean=4.95814630e-03,length_median=3.33097300e-03,length_95%HPD={9.94258800e-07,1.50743300e-02}],6[&prob=1.00000000e+00,prob_stddev=0.00000000e+00,prob_range={1.00000000e+00,1.00000000e+00},prob(percent)="100",prob+-sd="100+-0"]:3.765344e-03[&length_mean=5.44716372e-03,length_median=3.76534400e-03,length_95%HPD={7.31160900e-07,1.60075400e-02}],7[&prob=1.00000000e+00,prob_stddev=0.00000000e+00,prob_range={1.00000000e+00,1.00000000e+00},prob(percent)="100",prob+-sd="100+-0"]:3.365992e-03[&length_mean=4.93727300e-03,length_median=3.36599200e-03,length_95%HPD={2.05906400e-07,1.50495100e-02}])[&prob=9.99729730e-01,prob_stddev=3.82219882e-04,prob_range={9.99459459e-01,1.00000000e+00},prob(percent)="100",prob+-sd="100+-0"]:1.034371e-01[&length_mean=1.04809958e-01,length_median=1.03437100e-01,length_95%HPD={5.76482200e-02,1.57444300e-01}],8[&prob=1.00000000e+00,prob_stddev=0.00000000e+00,prob_range={1.00000000e+00,1.00000000e+00},prob(percent)="100",prob+-sd="100+-0"]:8.119736e-02[&length_mean=8.35425082e-02,length_median=8.11973600e-02,length_95%HPD={3.83351700e-02,1.31104300e-01}])[&prob=7.47837838e-01,prob_stddev=1.03199368e-02,prob_range={7.40540541e-01,7.55135135e-01},prob(percent)="75",prob+-sd="75+-1"]:3.105957e-02[&length_mean=3.30658827e-02,length_median=3.10595700e-02,length_95%HPD={3.83686200e-03,6.76066000e-02}],((((12[&prob=1.00000000e+00,prob_stddev=0.00000000e+00,prob_range={1.00000000e+00,1.00000000e+00},prob(percent)="100",prob+-sd="100+-0"]:3.412386e-03[&length_mean=4.97573468e-03,length_median=3.41238600e-03,length_95%HPD={2.35370700e-06,1.50855800e-02}],13[&prob=1.00000000e+00,prob_stddev=0.00000000e+00,prob_range={1.00000000e+00,1.00000000e+00},prob(percent)="100",prob+-sd="100+-0"]:3.430612e-03[&length_mean=4.92838358e-03,length_median=3.43061200e-03,length_95%HPD={5.39264500e-06,1.48820700e-02}])[&prob=1.00000000e+00,prob_stddev=0.00000000e+00,prob_range={1.00000000e+00,1.00000000e+00},prob(percent)="100",prob+-sd="100+-0"]:1.320869e-01[&length_mean=1.33857819e-01,length_median=1.32086900e-01,length_95%HPD={7.55395000e-02,1.97222800e-01}],(((((21[&prob=1.00000000e+00,prob_stddev=0.00000000e+00,prob_range={1.00000000e+00,1.00000000e+00},prob(percent)="100",prob+-sd="100+-0"]:1.828319e-01[&length_mean=1.85134812e-01,length_median=1.82831900e-01,length_95%HPD={1.17244600e-01,2.56910500e-01}],22[&prob=1.00000000e+00,prob_stddev=0.00000000e+00,prob_range={1.00000000e+00,1.00000000e+00},prob(percent)="100",prob+-sd="100+-0"]:9.163630e-02[&length_mean=9.38106375e-02,length_median=9.16363000e-02,length_95%HPD={4.21859200e-02,1.47923600e-01}])[&prob=7.08918919e-01,prob_stddev=1.94932140e-02,prob_range={6.95135135e-01,7.22702703e-01},prob(percent)="71",prob+-sd="71+-2"]:5.005172e-02[&length_mean=5.28569015e-02,length_median=5.00517200e-02,length_95%HPD={9.39640000e-03,1.01418800e-01}],(23[&prob=1.00000000e+00,prob_stddev=0.00000000e+00,prob_range={1.00000000e+00,1.00000000e+00},prob(percent)="100",prob+-sd="100+-0"]:6.956529e-03[&length_mean=8.52931826e-03,length_median=6.95652900e-03,length_95%HPD={5.13772200e-06,2.14545300e-02}],24[&prob=1.00000000e+00,prob_stddev=0.00000000e+00,prob_range={1.00000000e+00,1.00000000e+00},prob(percent)="100",prob+-sd="100+-0"]:3.979665e-03[&length_mean=5.55740844e-03,length_median=3.97966500e-03,length_95%HPD={1.32589300e-06,1.58978100e-02}])[&prob=1.00000000e+00,prob_stddev=0.00000000e+00,prob_range={1.00000000e+00,1.00000000e+00},prob(percent)="100",prob+-sd="100+-0"]:1.054367e-01[&length_mean=1.08800359e-01,length_median=1.05436700e-01,length_95%HPD={5.20876800e-02,1.74498500e-01}])[&prob=9.98918919e-01,prob_stddev=1.52887953e-03,prob_range={9.97837838e-01,1.00000000e+00},prob(percent)="100",prob+-sd="100+-0"]:2.096656e-01[&length_mean=2.10773032e-01,length_median=2.09665600e-01,length_95%HPD={7.82894400e-02,3.48140300e-01}],234[&prob=1.00000000e+00,prob_stddev=0.00000000e+00,prob_range={1.00000000e+00,1.00000000e+00},prob(percent)="100",prob+-sd="100+-0"]:8.868003e-01[&length_mean=8.94706693e-01,length_median=8.86800300e-01,length_95%HPD={6.57082900e-01,1.17093200e+00}])[&prob=5.16756757e-01,prob_stddev=5.65685425e-02,prob_range={4.76756757e-01,5.56756757e-01},prob(percent)="52",prob+-sd="52+-6"]:1.045945e-01[&length_mean=1.09590205e-01,length_median=1.04594500e-01,length_95%HPD={1.72552500e-02,2.07810700e-01}],25[&prob=1.00000000e+00,prob_stddev=0.00000000e+00,prob_range={1.00000000e+00,1.00000000e+00},prob(percent)="100",prob+-sd="100+-0"]:3.736441e-01[&length_mean=3.76673156e-01,length_median=3.73644100e-01,length_95%HPD={2.51707900e-01,4.96316000e-01}])[&prob=5.45135135e-01,prob_stddev=5.46574431e-02,prob_range={5.06486486e-01,5.83783784e-01},prob(percent)="55",prob+-sd="55+-5"]:1.208427e-01[&length_mean=1.25196192e-01,length_median=1.20842700e-01,length_95%HPD={3.97514500e-02,2.22168400e-01}],39[&prob=1.00000000e+00,prob_stddev=0.00000000e+00,prob_range={1.00000000e+00,1.00000000e+00},prob(percent)="100",prob+-sd="100+-0"]:4.113091e-01[&length_mean=4.01190712e-01,length_median=4.11309100e-01,length_95%HPD={1.82275700e-01,5.99301900e-01}])[&prob=9.77027027e-01,prob_stddev=4.20441870e-03,prob_range={9.74054054e-01,9.80000000e-01},prob(percent)="98",prob+-sd="98+-0"]:1.111244e-01[&length_mean=1.14247036e-01,length_median=1.11124400e-01,length_95%HPD={2.59981200e-02,2.04019400e-01}])[&prob=8.31081081e-01,prob_stddev=6.49773799e-03,prob_range={8.26486486e-01,8.35675676e-01},prob(percent)="83",prob+-sd="83+-1"]:3.067124e-02[&length_mean=3.39267325e-02,length_median=3.06712400e-02,length_95%HPD={4.11203000e-05,7.25803200e-02}],14[&prob=1.00000000e+00,prob_stddev=0.00000000e+00,prob_range={1.00000000e+00,1.00000000e+00},prob(percent)="100",prob+-sd="100+-0"]:1.763229e-01[&length_mean=1.78248476e-01,length_median=1.76322900e-01,length_95%HPD={1.13299200e-01,2.51538500e-01}])[&prob=9.99729730e-01,prob_stddev=3.82219882e-04,prob_range={9.99459459e-01,1.00000000e+00},prob(percent)="100",prob+-sd="100+-0"]:8.603593e-02[&length_mean=8.93662014e-02,length_median=8.60359300e-02,length_95%HPD={3.39317400e-02,1.50731000e-01}],(((((16[&prob=1.00000000e+00,prob_stddev=0.00000000e+00,prob_range={1.00000000e+00,1.00000000e+00},prob(percent)="100",prob+-sd="100+-0"]:2.639115e-01[&length_mean=2.65080812e-01,length_median=2.63911500e-01,length_95%HPD={1.58449500e-01,3.58106800e-01}],(17[&prob=1.00000000e+00,prob_stddev=0.00000000e+00,prob_range={1.00000000e+00,1.00000000e+00},prob(percent)="100",prob+-sd="100+-0"]:8.085073e-02[&length_mean=8.62874549e-02,length_median=8.08507300e-02,length_95%HPD={3.44611500e-03,1.71473300e-01}],18[&prob=1.00000000e+00,prob_stddev=0.00000000e+00,prob_range={1.00000000e+00,1.00000000e+00},prob(percent)="100",prob+-sd="100+-0"]:3.338685e-01[&length_mean=3.41245752e-01,length_median=3.33868500e-01,length_95%HPD={1.79474600e-01,5.00784400e-01}])[&prob=9.90000000e-01,prob_stddev=3.82219882e-04,prob_range={9.89729730e-01,9.90270270e-01},prob(percent)="99",prob+-sd="99+-0"]:1.157738e-01[&length_mean=1.20195787e-01,length_median=1.15773800e-01,length_95%HPD={1.68025000e-02,2.20169300e-01}])[&prob=9.18918919e-01,prob_stddev=0.00000000e+00,prob_range={9.18918919e-01,9.18918919e-01},prob(percent)="92",prob+-sd="92+-0"]:7.609990e-02[&length_mean=7.99558670e-02,length_median=7.60999000e-02,length_95%HPD={8.68462000e-03,1.49505500e-01}],((36[&prob=1.00000000e+00,prob_stddev=0.00000000e+00,prob_range={1.00000000e+00,1.00000000e+00},prob(percent)="100",prob+-sd="100+-0"]:6.461320e-02[&length_mean=6.63256896e-02,length_median=6.46132000e-02,length_95%HPD={2.03722100e-02,1.17921800e-01}],37[&prob=1.00000000e+00,prob_stddev=0.00000000e+00,prob_range={1.00000000e+00,1.00000000e+00},prob(percent)="100",prob+-sd="100+-0"]:5.384489e-02[&length_mean=5.59366185e-02,length_median=5.38448900e-02,length_95%HPD={1.19146100e-02,1.05082500e-01}])[&prob=1.00000000e+00,prob_stddev=0.00000000e+00,prob_range={1.00000000e+00,1.00000000e+00},prob(percent)="100",prob+-sd="100+-0"]:6.235682e-01[&length_mean=6.29992290e-01,length_median=6.23568200e-01,length_95%HPD={4.56192700e-01,8.15534700e-01}],(((40[&prob=1.00000000e+00,prob_stddev=0.00000000e+00,prob_range={1.00000000e+00,1.00000000e+00},prob(percent)="100",prob+-sd="100+-0"]:1.058520e-02[&length_mean=1.25234727e-02,length_median=1.05852000e-02,length_95%HPD={2.91170100e-04,2.94817900e-02}],41[&prob=1.00000000e+00,prob_stddev=0.00000000e+00,prob_range={1.00000000e+00,1.00000000e+00},prob(percent)="100",prob+-sd="100+-0"]:9.303378e-03[&length_mean=1.10684579e-02,length_median=9.30337800e-03,length_95%HPD={1.48998900e-06,2.69125100e-02}])[&prob=7.83783784e-01,prob_stddev=1.37599157e-02,prob_range={7.74054054e-01,7.93513514e-01},prob(percent)="78",prob+-sd="78+-1"]:2.657052e-02[&length_mean=2.99777199e-02,length_median=2.65705200e-02,length_95%HPD={2.52956500e-05,6.76317100e-02}],42[&prob=1.00000000e+00,prob_stddev=0.00000000e+00,prob_range={1.00000000e+00,1.00000000e+00},prob(percent)="100",prob+-sd="100+-0"]:8.982817e-02[&length_mean=9.23895405e-02,length_median=8.98281700e-02,length_95%HPD={3.82962200e-02,1.52980200e-01}])[&prob=1.00000000e+00,prob_stddev=0.00000000e+00,prob_range={1.00000000e+00,1.00000000e+00},prob(percent)="100",prob+-sd="100+-0"]:4.046858e-01[&length_mean=4.10126164e-01,length_median=4.04685800e-01,length_95%HPD={2.64834100e-01,5.60152400e-01}],((((((((47[&prob=1.00000000e+00,prob_stddev=0.00000000e+00,prob_range={1.00000000e+00,1.00000000e+00},prob(percent)="100",prob+-sd="100+-0"]:3.367006e-02[&length_mean=3.56492591e-02,length_median=3.36700600e-02,length_95%HPD={8.65212700e-03,6.52510700e-02}],((48[&prob=1.00000000e+00,prob_stddev=0.00000000e+00,prob_range={1.00000000e+00,1.00000000e+00},prob(percent)="100",prob+-sd="100+-0"]:2.452705e-02[&length_mean=2.64781622e-02,length_median=2.45270500e-02,length_95%HPD={6.32110600e-03,5.18270800e-02}],49[&prob=1.00000000e+00,prob_stddev=0.00000000e+00,prob_range={1.00000000e+00,1.00000000e+00},prob(percent)="100",prob+-sd="100+-0"]:9.260617e-03[&length_mean=1.11682421e-02,length_median=9.26061700e-03,length_95%HPD={2.30065300e-07,2.75245900e-02}])[&prob=6.82162162e-01,prob_stddev=2.29331929e-02,prob_range={6.65945946e-01,6.98378378e-01},prob(percent)="68",prob+-sd="68+-2"]:2.910379e-02[&length_mean=3.01800650e-02,length_median=2.91037900e-02,length_95%HPD={6.93651100e-04,5.92142400e-02}],53[&prob=1.00000000e+00,prob_stddev=0.00000000e+00,prob_range={1.00000000e+00,1.00000000e+00},prob(percent)="100",prob+-sd="100+-0"]:2.390109e-01[&length_mean=2.45331968e-01,length_median=2.39010900e-01,length_95%HPD={1.34618400e-01,3.67497100e-01}])[&prob=5.12162162e-01,prob_stddev=3.24886899e-02,prob_range={4.89189189e-01,5.35135135e-01},prob(percent)="51",prob+-sd="51+-3"]:3.206489e-02[&length_mean=3.33650287e-02,length_median=3.20648900e-02,length_95%HPD={1.31361500e-03,6.38283600e-02}])[&prob=9.99459459e-01,prob_stddev=7.64439763e-04,prob_range={9.98918919e-01,1.00000000e+00},prob(percent)="100",prob+-sd="100+-0"]:8.511153e-02[&length_mean=8.72905909e-02,length_median=8.51115300e-02,length_95%HPD={2.89866100e-02,1.50746100e-01}],(((50[&prob=1.00000000e+00,prob_stddev=0.00000000e+00,prob_range={1.00000000e+00,1.00000000e+00},prob(percent)="100",prob+-sd="100+-0"]:8.018664e-03[&length_mean=9.62931505e-03,length_median=8.01866400e-03,length_95%HPD={1.81409100e-04,2.32071100e-02}],51[&prob=1.00000000e+00,prob_stddev=0.00000000e+00,prob_range={1.00000000e+00,1.00000000e+00},prob(percent)="100",prob+-sd="100+-0"]:3.240678e-03[&length_mean=4.67736924e-03,length_median=3.24067800e-03,length_95%HPD={2.50822800e-06,1.42970500e-02}])[&prob=9.73783784e-01,prob_stddev=6.49773799e-03,prob_range={9.69189189e-01,9.78378378e-01},prob(percent)="97",prob+-sd="97+-1"]:3.401962e-02[&length_mean=3.69417237e-02,length_median=3.40196200e-02,length_95%HPD={1.81604900e-03,7.54910300e-02}],54[&prob=1.00000000e+00,prob_stddev=0.00000000e+00,prob_range={1.00000000e+00,1.00000000e+00},prob(percent)="100",prob+-sd="100+-0"]:2.180857e-01[&length_mean=2.22676189e-01,length_median=2.18085700e-01,length_95%HPD={1.25062500e-01,3.22674300e-01}])[&prob=9.54864865e-01,prob_stddev=9.55549704e-03,prob_range={9.48108108e-01,9.61621622e-01},prob(percent)="95",prob+-sd="95+-1"]:4.649040e-02[&length_mean=4.89052794e-02,length_median=4.64904000e-02,length_95%HPD={7.13734600e-03,9.34332600e-02}],52[&prob=1.00000000e+00,prob_stddev=0.00000000e+00,prob_range={1.00000000e+00,1.00000000e+00},prob(percent)="100",prob+-sd="100+-0"]:1.059487e-01[&length_mean=1.07552504e-01,length_median=1.05948700e-01,length_95%HPD={5.99127900e-02,1.60425800e-01}])[&prob=9.96486486e-01,prob_stddev=3.82219882e-04,prob_range={9.96216216e-01,9.96756757e-01},prob(percent)="100",prob+-sd="100+-0"]:6.739601e-02[&length_mean=7.00836923e-02,length_median=6.73960100e-02,length_95%HPD={2.02767100e-02,1.24262100e-01}])[&prob=1.00000000e+00,prob_stddev=0.00000000e+00,prob_range={1.00000000e+00,1.00000000e+00},prob(percent)="100",prob+-sd="100+-0"]:2.879781e-01[&length_mean=2.92732050e-01,length_median=2.87978100e-01,length_95%HPD={1.98850600e-01,4.03160200e-01}],62[&prob=1.00000000e+00,prob_stddev=0.00000000e+00,prob_range={1.00000000e+00,1.00000000e+00},prob(percent)="100",prob+-sd="100+-0"]:3.106594e-01[&length_mean=3.12637769e-01,length_median=3.10659400e-01,length_95%HPD={2.07325000e-01,4.26238600e-01}])[&prob=6.80810811e-01,prob_stddev=1.03199368e-02,prob_range={6.73513514e-01,6.88108108e-01},prob(percent)="68",prob+-sd="68+-1"]:6.266465e-02[&length_mean=6.62456301e-02,length_median=6.26646500e-02,length_95%HPD={8.42700100e-03,1.26423100e-01}],(((((((66[&prob=1.00000000e+00,prob_stddev=0.00000000e+00,prob_range={1.00000000e+00,1.00000000e+00},prob(percent)="100",prob+-sd="100+-0"]:4.307975e-02[&length_mean=4.71075278e-02,length_median=4.30797500e-02,length_95%HPD={5.91712200e-03,9.59676400e-02}],67[&prob=1.00000000e+00,prob_stddev=0.00000000e+00,prob_range={1.00000000e+00,1.00000000e+00},prob(percent)="100",prob+-sd="100+-0"]:3.638363e-02[&length_mean=3.94431498e-02,length_median=3.63836300e-02,length_95%HPD={2.90942400e-03,8.22058300e-02}])[&prob=7.25405405e-01,prob_stddev=2.98131508e-02,prob_range={7.04324324e-01,7.46486486e-01},prob(percent)="73",prob+-sd="73+-3"]:4.120532e-02[&length_mean=4.40061126e-02,length_median=4.12053200e-02,length_95%HPD={5.73539200e-05,9.17356100e-02}],68[&prob=1.00000000e+00,prob_stddev=0.00000000e+00,prob_range={1.00000000e+00,1.00000000e+00},prob(percent)="100",prob+-sd="100+-0"]:5.880793e-02[&length_mean=6.39454841e-02,length_median=5.88079300e-02,length_95%HPD={6.59883300e-03,1.28577500e-01}])[&prob=9.99729730e-01,prob_stddev=3.82219882e-04,prob_range={9.99459459e-01,1.00000000e+00},prob(percent)="100",prob+-sd="100+-0"]:6.489660e-02[&length_mean=6.81586525e-02,length_median=6.48966000e-02,length_95%HPD={2.04184600e-02,1.25358500e-01}],((((69[&prob=1.00000000e+00,prob_stddev=0.00000000e+00,prob_range={1.00000000e+00,1.00000000e+00},prob(percent)="100",prob+-sd="100+-0"]:8.208494e-03[&length_mean=9.83268389e-03,length_median=8.20849400e-03,length_95%HPD={2.44331600e-05,2.34933200e-02}],70[&prob=1.00000000e+00,prob_stddev=0.00000000e+00,prob_range={1.00000000e+00,1.00000000e+00},prob(percent)="100",prob+-sd="100+-0"]:7.919590e-03[&length_mean=9.50611973e-03,length_median=7.91959000e-03,length_95%HPD={9.37122500e-07,2.30662900e-02}],71[&prob=1.00000000e+00,prob_stddev=0.00000000e+00,prob_range={1.00000000e+00,1.00000000e+00},prob(percent)="100",prob+-sd="100+-0"]:1.933746e-02[&length_mean=2.15033293e-02,length_median=1.93374600e-02,length_95%HPD={2.72466300e-03,4.51761000e-02}],73[&prob=1.00000000e+00,prob_stddev=0.00000000e+00,prob_range={1.00000000e+00,1.00000000e+00},prob(percent)="100",prob+-sd="100+-0"]:2.691579e-02[&length_mean=2.83559317e-02,length_median=2.69157900e-02,length_95%HPD={6.67973300e-03,5.29671500e-02}])[&prob=8.12162162e-01,prob_stddev=4.96885846e-03,prob_range={8.08648649e-01,8.15675676e-01},prob(percent)="81",prob+-sd="81+-0"]:1.005671e-02[&length_mean=1.19337735e-02,length_median=1.00567100e-02,length_95%HPD={1.39206200e-04,2.86557900e-02}],72[&prob=1.00000000e+00,prob_stddev=0.00000000e+00,prob_range={1.00000000e+00,1.00000000e+00},prob(percent)="100",prob+-sd="100+-0"]:1.771656e-02[&length_mean=1.93380762e-02,length_median=1.77165600e-02,length_95%HPD={3.07412700e-03,4.16324200e-02}])[&prob=9.98378378e-01,prob_stddev=0.00000000e+00,prob_range={9.98378378e-01,9.98378378e-01},prob(percent)="100",prob+-sd="100+-0"]:3.294430e-02[&length_mean=3.49481108e-02,length_median=3.29443000e-02,length_95%HPD={6.80844600e-03,6.51525300e-02}],74[&prob=1.00000000e+00,prob_stddev=0.00000000e+00,prob_range={1.00000000e+00,1.00000000e+00},prob(percent)="100",prob+-sd="100+-0"]:4.916210e-02[&length_mean=5.10928503e-02,length_median=4.91621000e-02,length_95%HPD={1.71853700e-02,8.64509100e-02}])[&prob=1.00000000e+00,prob_stddev=0.00000000e+00,prob_range={1.00000000e+00,1.00000000e+00},prob(percent)="100",prob+-sd="100+-0"]:8.403154e-02[&length_mean=8.59999930e-02,length_median=8.40315400e-02,length_95%HPD={4.02926300e-02,1.36986500e-01}],75[&prob=1.00000000e+00,prob_stddev=0.00000000e+00,prob_range={1.00000000e+00,1.00000000e+00},prob(percent)="100",prob+-sd="100+-0"]:1.566033e-01[&length_mean=1.58610866e-01,length_median=1.56603300e-01,length_95%HPD={9.47607700e-02,2.21303200e-01}])[&prob=9.62702703e-01,prob_stddev=1.52887953e-03,prob_range={9.61621622e-01,9.63783784e-01},prob(percent)="96",prob+-sd="96+-0"]:4.165988e-02[&length_mean=4.44712261e-02,length_median=4.16598800e-02,length_95%HPD={6.68183000e-03,8.88302600e-02}])[&prob=1.00000000e+00,prob_stddev=0.00000000e+00,prob_range={1.00000000e+00,1.00000000e+00},prob(percent)="100",prob+-sd="100+-0"]:2.037489e-01[&length_mean=2.06790554e-01,length_median=2.03748900e-01,length_95%HPD={1.19349200e-01,2.93748100e-01}],((((79[&prob=1.00000000e+00,prob_stddev=0.00000000e+00,prob_range={1.00000000e+00,1.00000000e+00},prob(percent)="100",prob+-sd="100+-0"]:1.853163e-01[&length_mean=1.87708406e-01,length_median=1.85316300e-01,length_95%HPD={1.16917700e-01,2.61667000e-01}],((80[&prob=1.00000000e+00,prob_stddev=0.00000000e+00,prob_range={1.00000000e+00,1.00000000e+00},prob(percent)="100",prob+-sd="100+-0"]:1.158993e-02[&length_mean=1.31397978e-02,length_median=1.15899300e-02,length_95%HPD={1.25041500e-03,2.86462900e-02}],81[&prob=1.00000000e+00,prob_stddev=0.00000000e+00,prob_range={1.00000000e+00,1.00000000e+00},prob(percent)="100",prob+-sd="100+-0"]:3.177685e-03[&length_mean=4.69745706e-03,length_median=3.17768500e-03,length_95%HPD={5.46645000e-07,1.40489400e-02}])[&prob=1.00000000e+00,prob_stddev=0.00000000e+00,prob_range={1.00000000e+00,1.00000000e+00},prob(percent)="100",prob+-sd="100+-0"]:5.397217e-02[&length_mean=5.55461096e-02,length_median=5.39721700e-02,length_95%HPD={2.17567600e-02,9.47565300e-02}],82[&prob=1.00000000e+00,prob_stddev=0.00000000e+00,prob_range={1.00000000e+00,1.00000000e+00},prob(percent)="100",prob+-sd="100+-0"]:1.534228e-01[&length_mean=1.55468444e-01,length_median=1.53422800e-01,length_95%HPD={9.21556100e-02,2.18056300e-01}])[&prob=1.00000000e+00,prob_stddev=0.00000000e+00,prob_range={1.00000000e+00,1.00000000e+00},prob(percent)="100",prob+-sd="100+-0"]:1.163955e-01[&length_mean=1.18576025e-01,length_median=1.16395500e-01,length_95%HPD={5.84078900e-02,1.78483400e-01}])[&prob=1.00000000e+00,prob_stddev=0.00000000e+00,prob_range={1.00000000e+00,1.00000000e+00},prob(percent)="100",prob+-sd="100+-0"]:1.256377e-01[&length_mean=1.28233884e-01,length_median=1.25637700e-01,length_95%HPD={6.46546800e-02,2.00669800e-01}],((89[&prob=1.00000000e+00,prob_stddev=0.00000000e+00,prob_range={1.00000000e+00,1.00000000e+00},prob(percent)="100",prob+-sd="100+-0"]:2.403752e-02[&length_mean=2.78991033e-02,length_median=2.40375200e-02,length_95%HPD={1.18217500e-03,6.50035900e-02}],90[&prob=1.00000000e+00,prob_stddev=0.00000000e+00,prob_range={1.00000000e+00,1.00000000e+00},prob(percent)="100",prob+-sd="100+-0"]:1.705359e-01[&length_mean=1.72762430e-01,length_median=1.70535900e-01,length_95%HPD={8.75581100e-02,2.52041400e-01}])[&prob=1.00000000e+00,prob_stddev=0.00000000e+00,prob_range={1.00000000e+00,1.00000000e+00},prob(percent)="100",prob+-sd="100+-0"]:1.714771e-01[&length_mean=1.73849602e-01,length_median=1.71477100e-01,length_95%HPD={9.30724300e-02,2.51424200e-01}],91[&prob=1.00000000e+00,prob_stddev=0.00000000e+00,prob_range={1.00000000e+00,1.00000000e+00},prob(percent)="100",prob+-sd="100+-0"]:2.230939e-01[&length_mean=2.25216104e-01,length_median=2.23093900e-01,length_95%HPD={1.46846600e-01,3.07558700e-01}])[&prob=1.00000000e+00,prob_stddev=0.00000000e+00,prob_range={1.00000000e+00,1.00000000e+00},prob(percent)="100",prob+-sd="100+-0"]:1.179692e-01[&length_mean=1.20601445e-01,length_median=1.17969200e-01,length_95%HPD={5.91006200e-02,1.88668100e-01}])[&prob=6.73243243e-01,prob_stddev=1.71998947e-02,prob_range={6.61081081e-01,6.85405405e-01},prob(percent)="67",prob+-sd="67+-2"]:3.717265e-02[&length_mean=4.00837034e-02,length_median=3.71726500e-02,length_95%HPD={1.09684800e-03,8.14405100e-02}],((86[&prob=1.00000000e+00,prob_stddev=0.00000000e+00,prob_range={1.00000000e+00,1.00000000e+00},prob(percent)="100",prob+-sd="100+-0"]:5.975232e-02[&length_mean=6.20626513e-02,length_median=5.97523200e-02,length_95%HPD={2.22330600e-02,1.08780500e-01}],88[&prob=1.00000000e+00,prob_stddev=0.00000000e+00,prob_range={1.00000000e+00,1.00000000e+00},prob(percent)="100",prob+-sd="100+-0"]:2.089133e-01[&length_mean=2.11675865e-01,length_median=2.08913300e-01,length_95%HPD={1.18634900e-01,3.07544500e-01}])[&prob=1.00000000e+00,prob_stddev=0.00000000e+00,prob_range={1.00000000e+00,1.00000000e+00},prob(percent)="100",prob+-sd="100+-0"]:8.276274e-02[&length_mean=8.52152885e-02,length_median=8.27627400e-02,length_95%HPD={3.52254700e-02,1.40759600e-01}],87[&prob=1.00000000e+00,prob_stddev=0.00000000e+00,prob_range={1.00000000e+00,1.00000000e+00},prob(percent)="100",prob+-sd="100+-0"]:4.968486e-02[&length_mean=5.21116511e-02,length_median=4.96848600e-02,length_95%HPD={1.36983900e-02,9.13419600e-02}])[&prob=1.00000000e+00,prob_stddev=0.00000000e+00,prob_range={1.00000000e+00,1.00000000e+00},prob(percent)="100",prob+-sd="100+-0"]:1.741366e-01[&length_mean=1.76617715e-01,length_median=1.74136600e-01,length_95%HPD={1.07939000e-01,2.45797000e-01}])[&prob=9.34324324e-01,prob_stddev=1.33776959e-02,prob_range={9.24864865e-01,9.43783784e-01},prob(percent)="93",prob+-sd="93+-1"]:3.808991e-02[&length_mean=4.03677704e-02,length_median=3.80899100e-02,length_95%HPD={4.80651200e-03,7.89435800e-02}],(83[&prob=1.00000000e+00,prob_stddev=0.00000000e+00,prob_range={1.00000000e+00,1.00000000e+00},prob(percent)="100",prob+-sd="100+-0"]:1.902536e-01[&length_mean=1.93159711e-01,length_median=1.90253600e-01,length_95%HPD={1.22364600e-01,2.69695700e-01}],(84[&prob=1.00000000e+00,prob_stddev=0.00000000e+00,prob_range={1.00000000e+00,1.00000000e+00},prob(percent)="100",prob+-sd="100+-0"]:3.225473e-03[&length_mean=4.55719616e-03,length_median=3.22547300e-03,length_95%HPD={1.05090100e-07,1.36217800e-02}],85[&prob=1.00000000e+00,prob_stddev=0.00000000e+00,prob_range={1.00000000e+00,1.00000000e+00},prob(percent)="100",prob+-sd="100+-0"]:7.606844e-03[&length_mean=9.01706307e-03,length_median=7.60684400e-03,length_95%HPD={1.71143900e-05,2.14079900e-02}])[&prob=1.00000000e+00,prob_stddev=0.00000000e+00,prob_range={1.00000000e+00,1.00000000e+00},prob(percent)="100",prob+-sd="100+-0"]:1.450415e-01[&length_mean=1.47407733e-01,length_median=1.45041500e-01,length_95%HPD={8.34390600e-02,2.10861300e-01}])[&prob=9.92702703e-01,prob_stddev=3.43997894e-03,prob_range={9.90270270e-01,9.95135135e-01},prob(percent)="99",prob+-sd="99+-0"]:4.731946e-02[&length_mean=5.03582233e-02,length_median=4.73194600e-02,length_95%HPD={1.22803100e-02,9.50706500e-02}])[&prob=9.99459459e-01,prob_stddev=0.00000000e+00,prob_range={9.99459459e-01,9.99459459e-01},prob(percent)="100",prob+-sd="100+-0"]:8.152619e-02[&length_mean=8.44564893e-02,length_median=8.15261900e-02,length_95%HPD={2.86051100e-02,1.45304200e-01}])[&prob=9.48918919e-01,prob_stddev=1.10843766e-02,prob_range={9.41081081e-01,9.56756757e-01},prob(percent)="95",prob+-sd="95+-1"]:5.831766e-02[&length_mean=6.13506962e-02,length_median=5.83176600e-02,length_95%HPD={1.26481700e-02,1.08785700e-01}],92[&prob=1.00000000e+00,prob_stddev=0.00000000e+00,prob_range={1.00000000e+00,1.00000000e+00},prob(percent)="100",prob+-sd="100+-0"]:3.389081e-01[&length_mean=3.40753214e-01,length_median=3.38908100e-01,length_95%HPD={2.36743600e-01,4.40523600e-01}])[&prob=5.79459459e-01,prob_stddev=1.52887953e-02,prob_range={5.68648649e-01,5.90270270e-01},prob(percent)="58",prob+-sd="58+-2"]:3.985941e-02[&length_mean=4.50728445e-02,length_median=3.98594100e-02,length_95%HPD={4.55002700e-05,9.97011700e-02}],(76[&prob=1.00000000e+00,prob_stddev=0.00000000e+00,prob_range={1.00000000e+00,1.00000000e+00},prob(percent)="100",prob+-sd="100+-0"]:7.533150e-02[&length_mean=7.66182455e-02,length_median=7.53315000e-02,length_95%HPD={3.68378000e-02,1.23062800e-01}],(77[&prob=1.00000000e+00,prob_stddev=0.00000000e+00,prob_range={1.00000000e+00,1.00000000e+00},prob(percent)="100",prob+-sd="100+-0"]:3.272267e-03[&length_mean=4.62412806e-03,length_median=3.27226700e-03,length_95%HPD={1.60440900e-07,1.38508800e-02}],78[&prob=1.00000000e+00,prob_stddev=0.00000000e+00,prob_range={1.00000000e+00,1.00000000e+00},prob(percent)="100",prob+-sd="100+-0"]:3.185433e-03[&length_mean=4.57741700e-03,length_median=3.18543300e-03,length_95%HPD={1.08817300e-06,1.39405600e-02}])[&prob=9.99729730e-01,prob_stddev=3.82219882e-04,prob_range={9.99459459e-01,1.00000000e+00},prob(percent)="100",prob+-sd="100+-0"]:4.857728e-02[&length_mean=5.05727880e-02,length_median=4.85772800e-02,length_95%HPD={1.81090700e-02,9.08093200e-02}])[&prob=1.00000000e+00,prob_stddev=0.00000000e+00,prob_range={1.00000000e+00,1.00000000e+00},prob(percent)="100",prob+-sd="100+-0"]:2.495360e-01[&length_mean=2.52351733e-01,length_median=2.49536000e-01,length_95%HPD={1.54879100e-01,3.39357700e-01}])[&prob=9.57027027e-01,prob_stddev=1.03199368e-02,prob_range={9.49729730e-01,9.64324324e-01},prob(percent)="96",prob+-sd="96+-1"]:8.495074e-02[&length_mean=8.80035259e-02,length_median=8.49507400e-02,length_95%HPD={1.34792500e-02,1.60827600e-01}],93[&prob=1.00000000e+00,prob_stddev=0.00000000e+00,prob_range={1.00000000e+00,1.00000000e+00},prob(percent)="100",prob+-sd="100+-0"]:4.689695e-01[&length_mean=4.75756564e-01,length_median=4.68969500e-01,length_95%HPD={3.44798300e-01,6.04051500e-01}])[&prob=9.95135135e-01,prob_stddev=2.29331929e-03,prob_range={9.93513514e-01,9.96756757e-01},prob(percent)="100",prob+-sd="100+-0"]:9.992118e-02[&length_mean=1.04481416e-01,length_median=9.99211800e-02,length_95%HPD={2.86878000e-02,1.84448900e-01}])[&prob=6.88108108e-01,prob_stddev=7.64439763e-03,prob_range={6.82702703e-01,6.93513514e-01},prob(percent)="69",prob+-sd="69+-1"]:3.883463e-02[&length_mean=4.25024449e-02,length_median=3.88346300e-02,length_95%HPD={5.48831000e-03,8.86816000e-02}],((58[&prob=1.00000000e+00,prob_stddev=0.00000000e+00,prob_range={1.00000000e+00,1.00000000e+00},prob(percent)="100",prob+-sd="100+-0"]:7.799816e-03[&length_mean=9.30366189e-03,length_median=7.79981600e-03,length_95%HPD={3.21905400e-04,2.22824300e-02}],59[&prob=1.00000000e+00,prob_stddev=0.00000000e+00,prob_range={1.00000000e+00,1.00000000e+00},prob(percent)="100",prob+-sd="100+-0"]:3.169580e-03[&length_mean=4.64159216e-03,length_median=3.16958000e-03,length_95%HPD={3.76058200e-07,1.41412600e-02}],60[&prob=1.00000000e+00,prob_stddev=0.00000000e+00,prob_range={1.00000000e+00,1.00000000e+00},prob(percent)="100",prob+-sd="100+-0"]:7.438303e-03[&length_mean=9.02363917e-03,length_median=7.43830300e-03,length_95%HPD={4.10013400e-06,2.24531400e-02}])[&prob=1.00000000e+00,prob_stddev=0.00000000e+00,prob_range={1.00000000e+00,1.00000000e+00},prob(percent)="100",prob+-sd="100+-0"]:1.593453e-01[&length_mean=1.61944964e-01,length_median=1.59345300e-01,length_95%HPD={9.13716800e-02,2.37074900e-01}],61[&prob=1.00000000e+00,prob_stddev=0.00000000e+00,prob_range={1.00000000e+00,1.00000000e+00},prob(percent)="100",prob+-sd="100+-0"]:2.907015e-01[&length_mean=2.93242510e-01,length_median=2.90701500e-01,length_95%HPD={2.01246700e-01,3.88786600e-01}])[&prob=8.97297297e-01,prob_stddev=4.58663858e-03,prob_range={8.94054054e-01,9.00540541e-01},prob(percent)="90",prob+-sd="90+-0"]:6.571921e-02[&length_mean=6.83963384e-02,length_median=6.57192100e-02,length_95%HPD={1.08853000e-02,1.26671300e-01}])[&prob=9.60270270e-01,prob_stddev=1.14665965e-03,prob_range={9.59459459e-01,9.61081081e-01},prob(percent)="96",prob+-sd="96+-0"]:7.562626e-02[&length_mean=7.82163962e-02,length_median=7.56262600e-02,length_95%HPD={1.69577600e-02,1.40370400e-01}],((55[&prob=1.00000000e+00,prob_stddev=0.00000000e+00,prob_range={1.00000000e+00,1.00000000e+00},prob(percent)="100",prob+-sd="100+-0"]:3.252259e-03[&length_mean=4.69806134e-03,length_median=3.25225900e-03,length_95%HPD={2.26027200e-06,1.39603300e-02}],56[&prob=1.00000000e+00,prob_stddev=0.00000000e+00,prob_range={1.00000000e+00,1.00000000e+00},prob(percent)="100",prob+-sd="100+-0"]:3.349096e-03[&length_mean=4.73573295e-03,length_median=3.34909600e-03,length_95%HPD={3.41976500e-06,1.38751300e-02}])[&prob=1.00000000e+00,prob_stddev=0.00000000e+00,prob_range={1.00000000e+00,1.00000000e+00},prob(percent)="100",prob+-sd="100+-0"]:8.060794e-02[&length_mean=8.27902024e-02,length_median=8.06079400e-02,length_95%HPD={3.45915900e-02,1.35506600e-01}],(57[&prob=1.00000000e+00,prob_stddev=0.00000000e+00,prob_range={1.00000000e+00,1.00000000e+00},prob(percent)="100",prob+-sd="100+-0"]:1.503784e-01[&length_mean=1.53204274e-01,length_median=1.50378400e-01,length_95%HPD={9.13026600e-02,2.23829600e-01}],(63[&prob=1.00000000e+00,prob_stddev=0.00000000e+00,prob_range={1.00000000e+00,1.00000000e+00},prob(percent)="100",prob+-sd="100+-0"]:1.061211e-02[&length_mean=1.25945465e-02,length_median=1.06121100e-02,length_95%HPD={1.12864600e-05,3.04986300e-02}],(64[&prob=1.00000000e+00,prob_stddev=0.00000000e+00,prob_range={1.00000000e+00,1.00000000e+00},prob(percent)="100",prob+-sd="100+-0"]:5.000299e-03[&length_mean=7.28841739e-03,length_median=5.00029900e-03,length_95%HPD={4.19994600e-06,2.16104400e-02}],65[&prob=1.00000000e+00,prob_stddev=0.00000000e+00,prob_range={1.00000000e+00,1.00000000e+00},prob(percent)="100",prob+-sd="100+-0"]:4.646730e-03[&length_mean=6.65438176e-03,length_median=4.64673000e-03,length_95%HPD={3.40293500e-07,1.98118700e-02}])[&prob=8.86486486e-01,prob_stddev=5.35107834e-03,prob_range={8.82702703e-01,8.90270270e-01},prob(percent)="89",prob+-sd="89+-1"]:1.129624e-02[&length_mean=1.34172633e-02,length_median=1.12962400e-02,length_95%HPD={4.73743400e-05,3.17172000e-02}])[&prob=1.00000000e+00,prob_stddev=0.00000000e+00,prob_range={1.00000000e+00,1.00000000e+00},prob(percent)="100",prob+-sd="100+-0"]:3.637563e-01[&length_mean=3.66756562e-01,length_median=3.63756300e-01,length_95%HPD={2.63099200e-01,4.76689000e-01}])[&prob=5.45405405e-01,prob_stddev=3.13420303e-02,prob_range={5.23243243e-01,5.67567568e-01},prob(percent)="55",prob+-sd="55+-3"]:2.498177e-02[&length_mean=2.88298010e-02,length_median=2.49817700e-02,length_95%HPD={7.79373500e-05,6.72819100e-02}])[&prob=9.98648649e-01,prob_stddev=1.91109941e-03,prob_range={9.97297297e-01,1.00000000e+00},prob(percent)="100",prob+-sd="100+-0"]:9.560230e-02[&length_mean=9.90330822e-02,length_median=9.56023000e-02,length_95%HPD={4.10386000e-02,1.63030000e-01}])[&prob=9.94864865e-01,prob_stddev=4.20441870e-03,prob_range={9.91891892e-01,9.97837838e-01},prob(percent)="99",prob+-sd="99+-0"]:1.436592e-01[&length_mean=1.47964768e-01,length_median=1.43659200e-01,length_95%HPD={4.43137800e-02,2.50489100e-01}],(94[&prob=1.00000000e+00,prob_stddev=0.00000000e+00,prob_range={1.00000000e+00,1.00000000e+00},prob(percent)="100",prob+-sd="100+-0"]:2.163141e-02[&length_mean=2.33458883e-02,length_median=2.16314100e-02,length_95%HPD={4.80742400e-03,4.64565500e-02}],(95[&prob=1.00000000e+00,prob_stddev=0.00000000e+00,prob_range={1.00000000e+00,1.00000000e+00},prob(percent)="100",prob+-sd="100+-0"]:3.350383e-03[&length_mean=4.82532530e-03,length_median=3.35038300e-03,length_95%HPD={1.93842200e-06,1.41032900e-02}],96[&prob=1.00000000e+00,prob_stddev=0.00000000e+00,prob_range={1.00000000e+00,1.00000000e+00},prob(percent)="100",prob+-sd="100+-0"]:3.332377e-03[&length_mean=4.81364411e-03,length_median=3.33237700e-03,length_95%HPD={9.66586800e-08,1.46043500e-02}])[&prob=7.19729730e-01,prob_stddev=1.26132561e-02,prob_range={7.10810811e-01,7.28648649e-01},prob(percent)="72",prob+-sd="72+-1"]:7.045146e-03[&length_mean=8.66312153e-03,length_median=7.04514600e-03,length_95%HPD={2.46576400e-06,2.21403400e-02}],97[&prob=1.00000000e+00,prob_stddev=0.00000000e+00,prob_range={1.00000000e+00,1.00000000e+00},prob(percent)="100",prob+-sd="100+-0"]:3.329956e-03[&length_mean=5.03651475e-03,length_median=3.32995600e-03,length_95%HPD={2.15883700e-06,1.51171500e-02}])[&prob=1.00000000e+00,prob_stddev=0.00000000e+00,prob_range={1.00000000e+00,1.00000000e+00},prob(percent)="100",prob+-sd="100+-0"]:5.288984e-01[&length_mean=5.31680139e-01,length_median=5.28898400e-01,length_95%HPD={3.52977400e-01,6.92943000e-01}],(98[&prob=1.00000000e+00,prob_stddev=0.00000000e+00,prob_range={1.00000000e+00,1.00000000e+00},prob(percent)="100",prob+-sd="100+-0"]:7.047578e-01[&length_mean=7.10051463e-01,length_median=7.04757800e-01,length_95%HPD={5.11181800e-01,9.38037400e-01}],99[&prob=1.00000000e+00,prob_stddev=0.00000000e+00,prob_range={1.00000000e+00,1.00000000e+00},prob(percent)="100",prob+-sd="100+-0"]:3.222447e-01[&length_mean=3.26453963e-01,length_median=3.22244700e-01,length_95%HPD={1.84129800e-01,4.79033800e-01}],258[&prob=1.00000000e+00,prob_stddev=0.00000000e+00,prob_range={1.00000000e+00,1.00000000e+00},prob(percent)="100",prob+-sd="100+-0"]:8.736645e-01[&length_mean=8.64126748e-01,length_median=8.73664500e-01,length_95%HPD={5.13582500e-01,1.18231600e+00}])[&prob=8.85675676e-01,prob_stddev=4.39552864e-02,prob_range={8.54594595e-01,9.16756757e-01},prob(percent)="89",prob+-sd="89+-4"]:2.287742e-01[&length_mean=2.32547764e-01,length_median=2.28774200e-01,length_95%HPD={8.23792800e-02,3.77015700e-01}],((((197[&prob=1.00000000e+00,prob_stddev=0.00000000e+00,prob_range={1.00000000e+00,1.00000000e+00},prob(percent)="100",prob+-sd="100+-0"]:5.773284e-02[&length_mean=5.95601936e-02,length_median=5.77328400e-02,length_95%HPD={2.33704800e-02,9.81178500e-02}],198[&prob=1.00000000e+00,prob_stddev=0.00000000e+00,prob_range={1.00000000e+00,1.00000000e+00},prob(percent)="100",prob+-sd="100+-0"]:3.944066e-02[&length_mean=4.11579235e-02,length_median=3.94406600e-02,length_95%HPD={1.25948800e-02,7.22294800e-02}])[&prob=9.43513514e-01,prob_stddev=3.82219882e-04,prob_range={9.43243243e-01,9.43783784e-01},prob(percent)="94",prob+-sd="94+-0"]:2.574856e-02[&length_mean=2.77464734e-02,length_median=2.57485600e-02,length_95%HPD={2.85729000e-03,5.40186400e-02}],(199[&prob=1.00000000e+00,prob_stddev=0.00000000e+00,prob_range={1.00000000e+00,1.00000000e+00},prob(percent)="100",prob+-sd="100+-0"]:5.723474e-02[&length_mean=5.94049028e-02,length_median=5.72347400e-02,length_95%HPD={2.34378200e-02,9.64277000e-02}],200[&prob=1.00000000e+00,prob_stddev=0.00000000e+00,prob_range={1.00000000e+00,1.00000000e+00},prob(percent)="100",prob+-sd="100+-0"]:2.946927e-02[&length_mean=3.10939950e-02,length_median=2.94692700e-02,length_95%HPD={6.76900800e-03,5.85461400e-02}])[&prob=8.94324324e-01,prob_stddev=4.20441870e-03,prob_range={8.91351351e-01,8.97297297e-01},prob(percent)="89",prob+-sd="89+-0"]:2.066146e-02[&length_mean=2.27132622e-02,length_median=2.06614600e-02,length_95%HPD={1.82127300e-03,4.79958000e-02}])[&prob=7.92162162e-01,prob_stddev=1.33776959e-02,prob_range={7.82702703e-01,8.01621622e-01},prob(percent)="79",prob+-sd="79+-1"]:3.494615e-02[&length_mean=3.90050564e-02,length_median=3.49461500e-02,length_95%HPD={5.91775200e-05,8.65192000e-02}],(((201[&prob=1.00000000e+00,prob_stddev=0.00000000e+00,prob_range={1.00000000e+00,1.00000000e+00},prob(percent)="100",prob+-sd="100+-0"]:7.016206e-03[&length_mean=8.78798061e-03,length_median=7.01620600e-03,length_95%HPD={1.91089600e-05,2.33128600e-02}],202[&prob=1.00000000e+00,prob_stddev=0.00000000e+00,prob_range={1.00000000e+00,1.00000000e+00},prob(percent)="100",prob+-sd="100+-0"]:5.097396e-03[&length_mean=6.91918055e-03,length_median=5.09739600e-03,length_95%HPD={3.84219300e-06,2.00316300e-02}])[&prob=9.99729730e-01,prob_stddev=3.82219882e-04,prob_range={9.99459459e-01,1.00000000e+00},prob(percent)="100",prob+-sd="100+-0"]:3.397402e-02[&length_mean=3.58027115e-02,length_median=3.39740200e-02,length_95%HPD={7.17011800e-03,6.66886300e-02}],203[&prob=1.00000000e+00,prob_stddev=0.00000000e+00,prob_range={1.00000000e+00,1.00000000e+00},prob(percent)="100",prob+-sd="100+-0"]:1.392540e-01[&length_mean=1.41351217e-01,length_median=1.39254000e-01,length_95%HPD={8.49557800e-02,2.01718700e-01}])[&prob=9.99189189e-01,prob_stddev=3.82219882e-04,prob_range={9.98918919e-01,9.99459459e-01},prob(percent)="100",prob+-sd="100+-0"]:5.094047e-02[&length_mean=5.33219952e-02,length_median=5.09404700e-02,length_95%HPD={1.40004600e-02,1.00336500e-01}],204[&prob=1.00000000e+00,prob_stddev=0.00000000e+00,prob_range={1.00000000e+00,1.00000000e+00},prob(percent)="100",prob+-sd="100+-0"]:2.792857e-01[&length_mean=2.81922079e-01,length_median=2.79285700e-01,length_95%HPD={1.92973600e-01,3.73803700e-01}])[&prob=9.98918919e-01,prob_stddev=0.00000000e+00,prob_range={9.98918919e-01,9.98918919e-01},prob(percent)="100",prob+-sd="100+-0"]:8.692412e-02[&length_mean=8.85874925e-02,length_median=8.69241200e-02,length_95%HPD={2.70346200e-02,1.54100400e-01}])[&prob=1.00000000e+00,prob_stddev=0.00000000e+00,prob_range={1.00000000e+00,1.00000000e+00},prob(percent)="100",prob+-sd="100+-0"]:7.052919e-01[&length_mean=7.08201894e-01,length_median=7.05291900e-01,length_95%HPD={4.91110900e-01,9.27289600e-01}],((((((205[&prob=1.00000000e+00,prob_stddev=0.00000000e+00,prob_range={1.00000000e+00,1.00000000e+00},prob(percent)="100",prob+-sd="100+-0"]:1.012124e-02[&length_mean=1.17881705e-02,length_median=1.01212400e-02,length_95%HPD={9.27314500e-06,2.69230400e-02}],206[&prob=1.00000000e+00,prob_stddev=0.00000000e+00,prob_range={1.00000000e+00,1.00000000e+00},prob(percent)="100",prob+-sd="100+-0"]:4.629072e-03[&length_mean=6.26504011e-03,length_median=4.62907200e-03,length_95%HPD={3.94214500e-06,1.79854900e-02}])[&prob=1.00000000e+00,prob_stddev=0.00000000e+00,prob_range={1.00000000e+00,1.00000000e+00},prob(percent)="100",prob+-sd="100+-0"]:1.393318e-01[&length_mean=1.41799932e-01,length_median=1.39331800e-01,length_95%HPD={8.62797600e-02,2.00943000e-01}],(207[&prob=1.00000000e+00,prob_stddev=0.00000000e+00,prob_range={1.00000000e+00,1.00000000e+00},prob(percent)="100",prob+-sd="100+-0"]:3.112792e-03[&length_mean=4.51494940e-03,length_median=3.11279200e-03,length_95%HPD={2.89973700e-06,1.36959200e-02}],208[&prob=1.00000000e+00,prob_stddev=0.00000000e+00,prob_range={1.00000000e+00,1.00000000e+00},prob(percent)="100",prob+-sd="100+-0"]:1.206125e-02[&length_mean=1.35113741e-02,length_median=1.20612500e-02,length_95%HPD={1.72167000e-03,2.86813200e-02}])[&prob=1.00000000e+00,prob_stddev=0.00000000e+00,prob_range={1.00000000e+00,1.00000000e+00},prob(percent)="100",prob+-sd="100+-0"]:7.411180e-02[&length_mean=7.63371386e-02,length_median=7.41118000e-02,length_95%HPD={3.60214500e-02,1.20234700e-01}])[&prob=9.99729730e-01,prob_stddev=3.82219882e-04,prob_range={9.99459459e-01,1.00000000e+00},prob(percent)="100",prob+-sd="100+-0"]:5.625192e-02[&length_mean=5.83942343e-02,length_median=5.62519200e-02,length_95%HPD={1.96350800e-02,1.00060400e-01}],((209[&prob=1.00000000e+00,prob_stddev=0.00000000e+00,prob_range={1.00000000e+00,1.00000000e+00},prob(percent)="100",prob+-sd="100+-0"]:4.915580e-02[&length_mean=5.07281031e-02,length_median=4.91558000e-02,length_95%HPD={2.21423500e-02,8.11252500e-02}],210[&prob=1.00000000e+00,prob_stddev=0.00000000e+00,prob_range={1.00000000e+00,1.00000000e+00},prob(percent)="100",prob+-sd="100+-0"]:3.280490e-02[&length_mean=3.45992726e-02,length_median=3.28049000e-02,length_95%HPD={1.21560300e-02,6.23938800e-02}])[&prob=9.98648649e-01,prob_stddev=3.82219882e-04,prob_range={9.98378378e-01,9.98918919e-01},prob(percent)="100",prob+-sd="100+-0"]:2.113725e-02[&length_mean=2.29367619e-02,length_median=2.11372500e-02,length_95%HPD={3.03709900e-03,4.60612100e-02}],(211[&prob=1.00000000e+00,prob_stddev=0.00000000e+00,prob_range={1.00000000e+00,1.00000000e+00},prob(percent)="100",prob+-sd="100+-0"]:1.446404e-01[&length_mean=1.46080735e-01,length_median=1.44640400e-01,length_95%HPD={8.97928000e-02,2.04211100e-01}],(212[&prob=1.00000000e+00,prob_stddev=0.00000000e+00,prob_range={1.00000000e+00,1.00000000e+00},prob(percent)="100",prob+-sd="100+-0"]:3.154621e-03[&length_mean=4.58564946e-03,length_median=3.15462100e-03,length_95%HPD={3.29697700e-07,1.41900400e-02}],213[&prob=1.00000000e+00,prob_stddev=0.00000000e+00,prob_range={1.00000000e+00,1.00000000e+00},prob(percent)="100",prob+-sd="100+-0"]:7.335352e-03[&length_mean=9.01171419e-03,length_median=7.33535200e-03,length_95%HPD={1.23944000e-04,2.16221000e-02}])[&prob=1.00000000e+00,prob_stddev=0.00000000e+00,prob_range={1.00000000e+00,1.00000000e+00},prob(percent)="100",prob+-sd="100+-0"]:7.058652e-02[&length_mean=7.25785306e-02,length_median=7.05865200e-02,length_95%HPD={3.56981200e-02,1.15865300e-01}])[&prob=1.00000000e+00,prob_stddev=0.00000000e+00,prob_range={1.00000000e+00,1.00000000e+00},prob(percent)="100",prob+-sd="100+-0"]:7.302345e-02[&length_mean=7.52634013e-02,length_median=7.30234500e-02,length_95%HPD={3.51303300e-02,1.18618600e-01}])[&prob=9.72432432e-01,prob_stddev=7.64439763e-04,prob_range={9.71891892e-01,9.72972973e-01},prob(percent)="97",prob+-sd="97+-0"]:2.634250e-02[&length_mean=2.89823023e-02,length_median=2.63425000e-02,length_95%HPD={2.03185000e-03,6.02645900e-02}])[&prob=1.00000000e+00,prob_stddev=0.00000000e+00,prob_range={1.00000000e+00,1.00000000e+00},prob(percent)="100",prob+-sd="100+-0"]:8.686232e-02[&length_mean=8.91238768e-02,length_median=8.68623200e-02,length_95%HPD={3.95734900e-02,1.47356700e-01}],(((214[&prob=1.00000000e+00,prob_stddev=0.00000000e+00,prob_range={1.00000000e+00,1.00000000e+00},prob(percent)="100",prob+-sd="100+-0"]:9.998777e-03[&length_mean=1.18804539e-02,length_median=9.99877700e-03,length_95%HPD={3.19528600e-04,2.80440800e-02}],(215[&prob=1.00000000e+00,prob_stddev=0.00000000e+00,prob_range={1.00000000e+00,1.00000000e+00},prob(percent)="100",prob+-sd="100+-0"]:1.117208e-02[&length_mean=1.29077431e-02,length_median=1.11720800e-02,length_95%HPD={1.05447400e-03,2.90149300e-02}],216[&prob=1.00000000e+00,prob_stddev=0.00000000e+00,prob_range={1.00000000e+00,1.00000000e+00},prob(percent)="100",prob+-sd="100+-0"]:5.461342e-03[&length_mean=6.98916697e-03,length_median=5.46134200e-03,length_95%HPD={9.51582100e-06,1.89787800e-02}])[&prob=5.40000000e-01,prob_stddev=6.87995787e-03,prob_range={5.35135135e-01,5.44864865e-01},prob(percent)="54",prob+-sd="54+-1"]:6.317232e-03[&length_mean=7.99142270e-03,length_median=6.31723200e-03,length_95%HPD={1.73916000e-06,2.13079900e-02}])[&prob=1.00000000e+00,prob_stddev=0.00000000e+00,prob_range={1.00000000e+00,1.00000000e+00},prob(percent)="100",prob+-sd="100+-0"]:1.276863e-01[&length_mean=1.29644111e-01,length_median=1.27686300e-01,length_95%HPD={7.35942100e-02,1.90285700e-01}],217[&prob=1.00000000e+00,prob_stddev=0.00000000e+00,prob_range={1.00000000e+00,1.00000000e+00},prob(percent)="100",prob+-sd="100+-0"]:1.638987e-01[&length_mean=1.67144118e-01,length_median=1.63898700e-01,length_95%HPD={1.02495500e-01,2.34801500e-01}])[&prob=9.99729730e-01,prob_stddev=3.82219882e-04,prob_range={9.99459459e-01,1.00000000e+00},prob(percent)="100",prob+-sd="100+-0"]:7.582034e-02[&length_mean=7.85135228e-02,length_median=7.58203400e-02,length_95%HPD={2.81808900e-02,1.31719400e-01}],218[&prob=1.00000000e+00,prob_stddev=0.00000000e+00,prob_range={1.00000000e+00,1.00000000e+00},prob(percent)="100",prob+-sd="100+-0"]:1.814515e-01[&length_mean=1.83539485e-01,length_median=1.81451500e-01,length_95%HPD={1.14733100e-01,2.54143800e-01}])[&prob=9.98648649e-01,prob_stddev=3.82219882e-04,prob_range={9.98378378e-01,9.98918919e-01},prob(percent)="100",prob+-sd="100+-0"]:6.589851e-02[&length_mean=6.87339296e-02,length_median=6.58985100e-02,length_95%HPD={2.01694900e-02,1.20332200e-01}])[&prob=1.00000000e+00,prob_stddev=0.00000000e+00,prob_range={1.00000000e+00,1.00000000e+00},prob(percent)="100",prob+-sd="100+-0"]:1.379095e-01[&length_mean=1.42624214e-01,length_median=1.37909500e-01,length_95%HPD={5.99398500e-02,2.27231200e-01}],((((219[&prob=1.00000000e+00,prob_stddev=0.00000000e+00,prob_range={1.00000000e+00,1.00000000e+00},prob(percent)="100",prob+-sd="100+-0"]:3.478843e-03[&length_mean=5.06379621e-03,length_median=3.47884300e-03,length_95%HPD={4.21287400e-07,1.51072000e-02}],220[&prob=1.00000000e+00,prob_stddev=0.00000000e+00,prob_range={1.00000000e+00,1.00000000e+00},prob(percent)="100",prob+-sd="100+-0"]:8.362817e-03[&length_mean=9.93362190e-03,length_median=8.36281700e-03,length_95%HPD={1.73161200e-04,2.40964900e-02}])[&prob=8.40270270e-01,prob_stddev=9.55549704e-03,prob_range={8.33513514e-01,8.47027027e-01},prob(percent)="84",prob+-sd="84+-1"]:1.825624e-02[&length_mean=2.06501362e-02,length_median=1.82562400e-02,length_95%HPD={1.34302500e-04,4.72830100e-02}],221[&prob=1.00000000e+00,prob_stddev=0.00000000e+00,prob_range={1.00000000e+00,1.00000000e+00},prob(percent)="100",prob+-sd="100+-0"]:9.534981e-02[&length_mean=9.73157782e-02,length_median=9.53498100e-02,length_95%HPD={4.37413800e-02,1.51444500e-01}])[&prob=1.00000000e+00,prob_stddev=0.00000000e+00,prob_range={1.00000000e+00,1.00000000e+00},prob(percent)="100",prob+-sd="100+-0"]:1.426205e-01[&length_mean=1.44990293e-01,length_median=1.42620500e-01,length_95%HPD={8.39560800e-02,2.15000400e-01}],222[&prob=1.00000000e+00,prob_stddev=0.00000000e+00,prob_range={1.00000000e+00,1.00000000e+00},prob(percent)="100",prob+-sd="100+-0"]:1.668612e-01[&length_mean=1.68321443e-01,length_median=1.66861200e-01,length_95%HPD={1.01174100e-01,2.41987800e-01}])[&prob=1.00000000e+00,prob_stddev=0.00000000e+00,prob_range={1.00000000e+00,1.00000000e+00},prob(percent)="100",prob+-sd="100+-0"]:1.152635e-01[&length_mean=1.18050846e-01,length_median=1.15263500e-01,length_95%HPD={5.46274700e-02,1.86435000e-01}],(223[&prob=1.00000000e+00,prob_stddev=0.00000000e+00,prob_range={1.00000000e+00,1.00000000e+00},prob(percent)="100",prob+-sd="100+-0"]:1.134013e-01[&length_mean=1.16380148e-01,length_median=1.13401300e-01,length_95%HPD={5.93881800e-02,1.76714100e-01}],224[&prob=1.00000000e+00,prob_stddev=0.00000000e+00,prob_range={1.00000000e+00,1.00000000e+00},prob(percent)="100",prob+-sd="100+-0"]:2.723968e-01[&length_mean=2.74288218e-01,length_median=2.72396800e-01,length_95%HPD={1.80719500e-01,3.66703900e-01}])[&prob=9.29189189e-01,prob_stddev=3.82219882e-03,prob_range={9.26486486e-01,9.31891892e-01},prob(percent)="93",prob+-sd="93+-0"]:5.119704e-02[&length_mean=5.45740699e-02,length_median=5.11970400e-02,length_95%HPD={1.10615800e-02,1.06933800e-01}])[&prob=9.54054054e-01,prob_stddev=6.11551811e-03,prob_range={9.49729730e-01,9.58378378e-01},prob(percent)="95",prob+-sd="95+-1"]:8.659916e-02[&length_mean=8.98256622e-02,length_median=8.65991600e-02,length_95%HPD={2.67749900e-02,1.53752200e-01}])[&prob=1.00000000e+00,prob_stddev=0.00000000e+00,prob_range={1.00000000e+00,1.00000000e+00},prob(percent)="100",prob+-sd="100+-0"]:2.356779e-01[&length_mean=2.39850523e-01,length_median=2.35677900e-01,length_95%HPD={1.32754400e-01,3.60450000e-01}],((225[&prob=1.00000000e+00,prob_stddev=0.00000000e+00,prob_range={1.00000000e+00,1.00000000e+00},prob(percent)="100",prob+-sd="100+-0"]:1.888149e-01[&length_mean=1.91193415e-01,length_median=1.88814900e-01,length_95%HPD={1.13911000e-01,2.73396500e-01}],((226[&prob=1.00000000e+00,prob_stddev=0.00000000e+00,prob_range={1.00000000e+00,1.00000000e+00},prob(percent)="100",prob+-sd="100+-0"]:1.270617e-01[&length_mean=1.28733555e-01,length_median=1.27061700e-01,length_95%HPD={7.06545500e-02,1.92160400e-01}],(227[&prob=1.00000000e+00,prob_stddev=0.00000000e+00,prob_range={1.00000000e+00,1.00000000e+00},prob(percent)="100",prob+-sd="100+-0"]:5.508754e-02[&length_mean=5.67678825e-02,length_median=5.50875400e-02,length_95%HPD={2.44757700e-02,9.45833200e-02}],228[&prob=1.00000000e+00,prob_stddev=0.00000000e+00,prob_range={1.00000000e+00,1.00000000e+00},prob(percent)="100",prob+-sd="100+-0"]:1.203906e-02[&length_mean=1.43977943e-02,length_median=1.20390600e-02,length_95%HPD={1.70811800e-05,3.54049800e-02}])[&prob=1.00000000e+00,prob_stddev=0.00000000e+00,prob_range={1.00000000e+00,1.00000000e+00},prob(percent)="100",prob+-sd="100+-0"]:2.022268e-01[&length_mean=2.04724261e-01,length_median=2.02226800e-01,length_95%HPD={1.30406900e-01,2.83594900e-01}])[&prob=9.95405405e-01,prob_stddev=1.14665965e-03,prob_range={9.94594595e-01,9.96216216e-01},prob(percent)="100",prob+-sd="100+-0"]:8.681351e-02[&length_mean=8.98270635e-02,length_median=8.68135100e-02,length_95%HPD={2.35457200e-02,1.56313200e-01}],((230[&prob=1.00000000e+00,prob_stddev=0.00000000e+00,prob_range={1.00000000e+00,1.00000000e+00},prob(percent)="100",prob+-sd="100+-0"]:7.821880e-02[&length_mean=8.01830882e-02,length_median=7.82188000e-02,length_95%HPD={3.03610300e-02,1.30513500e-01}],231[&prob=1.00000000e+00,prob_stddev=0.00000000e+00,prob_range={1.00000000e+00,1.00000000e+00},prob(percent)="100",prob+-sd="100+-0"]:1.457161e-01[&length_mean=1.47738459e-01,length_median=1.45716100e-01,length_95%HPD={8.71252000e-02,2.13729100e-01}])[&prob=1.00000000e+00,prob_stddev=0.00000000e+00,prob_range={1.00000000e+00,1.00000000e+00},prob(percent)="100",prob+-sd="100+-0"]:1.644577e-01[&length_mean=1.67603712e-01,length_median=1.64457700e-01,length_95%HPD={8.82988100e-02,2.48850000e-01}],232[&prob=1.00000000e+00,prob_stddev=0.00000000e+00,prob_range={1.00000000e+00,1.00000000e+00},prob(percent)="100",prob+-sd="100+-0"]:2.577807e-01[&length_mean=2.60897423e-01,length_median=2.57780700e-01,length_95%HPD={1.67996400e-01,3.64073100e-01}])[&prob=1.00000000e+00,prob_stddev=0.00000000e+00,prob_range={1.00000000e+00,1.00000000e+00},prob(percent)="100",prob+-sd="100+-0"]:1.627074e-01[&length_mean=1.66166064e-01,length_median=1.62707400e-01,length_95%HPD={8.98464500e-02,2.47382000e-01}])[&prob=1.00000000e+00,prob_stddev=0.00000000e+00,prob_range={1.00000000e+00,1.00000000e+00},prob(percent)="100",prob+-sd="100+-0"]:9.742340e-02[&length_mean=1.00633429e-01,length_median=9.74234000e-02,length_95%HPD={3.49272300e-02,1.71063700e-01}])[&prob=9.05945946e-01,prob_stddev=1.91109941e-02,prob_range={8.92432432e-01,9.19459459e-01},prob(percent)="91",prob+-sd="91+-2"]:6.026629e-02[&length_mean=6.41272148e-02,length_median=6.02662900e-02,length_95%HPD={7.15267700e-03,1.28832800e-01}],229[&prob=1.00000000e+00,prob_stddev=0.00000000e+00,prob_range={1.00000000e+00,1.00000000e+00},prob(percent)="100",prob+-sd="100+-0"]:3.490864e-01[&length_mean=3.53294693e-01,length_median=3.49086400e-01,length_95%HPD={2.43114900e-01,4.70485200e-01}])[&prob=9.63783784e-01,prob_stddev=4.58663858e-03,prob_range={9.60540541e-01,9.67027027e-01},prob(percent)="96",prob+-sd="96+-0"]:1.071960e-01[&length_mean=1.10251994e-01,length_median=1.07196000e-01,length_95%HPD={2.45726000e-02,2.01275400e-01}])[&prob=1.00000000e+00,prob_stddev=0.00000000e+00,prob_range={1.00000000e+00,1.00000000e+00},prob(percent)="100",prob+-sd="100+-0"]:3.809794e-01[&length_mean=3.86394638e-01,length_median=3.80979400e-01,length_95%HPD={2.15214500e-01,5.64339500e-01}],((((235[&prob=1.00000000e+00,prob_stddev=0.00000000e+00,prob_range={1.00000000e+00,1.00000000e+00},prob(percent)="100",prob+-sd="100+-0"]:1.212879e-02[&length_mean=1.37879335e-02,length_median=1.21287900e-02,length_95%HPD={1.68431300e-03,3.01084700e-02}],236[&prob=1.00000000e+00,prob_stddev=0.00000000e+00,prob_range={1.00000000e+00,1.00000000e+00},prob(percent)="100",prob+-sd="100+-0"]:3.488496e-03[&length_mean=5.03877673e-03,length_median=3.48849600e-03,length_95%HPD={2.99618000e-06,1.53515000e-02}])[&prob=9.99189189e-01,prob_stddev=1.14665965e-03,prob_range={9.98378378e-01,1.00000000e+00},prob(percent)="100",prob+-sd="100+-0"]:5.571902e-02[&length_mean=5.76135114e-02,length_median=5.57190200e-02,length_95%HPD={1.73189800e-02,1.00603500e-01}],(237[&prob=1.00000000e+00,prob_stddev=0.00000000e+00,prob_range={1.00000000e+00,1.00000000e+00},prob(percent)="100",prob+-sd="100+-0"]:1.253765e-02[&length_mean=1.47170485e-02,length_median=1.25376500e-02,length_95%HPD={4.60329900e-04,3.44803700e-02}],(238[&prob=1.00000000e+00,prob_stddev=0.00000000e+00,prob_range={1.00000000e+00,1.00000000e+00},prob(percent)="100",prob+-sd="100+-0"]:3.363486e-03[&length_mean=4.97514780e-03,length_median=3.36348600e-03,length_95%HPD={1.15898200e-06,1.51951800e-02}],(239[&prob=1.00000000e+00,prob_stddev=0.00000000e+00,prob_range={1.00000000e+00,1.00000000e+00},prob(percent)="100",prob+-sd="100+-0"]:3.590316e-03[&length_mean=5.06250309e-03,length_median=3.59031600e-03,length_95%HPD={3.40237500e-06,1.50358900e-02}],((240[&prob=1.00000000e+00,prob_stddev=0.00000000e+00,prob_range={1.00000000e+00,1.00000000e+00},prob(percent)="100",prob+-sd="100+-0"]:3.630297e-03[&length_mean=5.07716132e-03,length_median=3.63029700e-03,length_95%HPD={2.48009300e-06,1.53118700e-02}],244[&prob=1.00000000e+00,prob_stddev=0.00000000e+00,prob_range={1.00000000e+00,1.00000000e+00},prob(percent)="100",prob+-sd="100+-0"]:2.319336e-02[&length_mean=2.50880166e-02,length_median=2.31933600e-02,length_95%HPD={6.66058000e-03,4.68875900e-02}])[&prob=9.94324324e-01,prob_stddev=1.14665965e-03,prob_range={9.93513514e-01,9.95135135e-01},prob(percent)="99",prob+-sd="99+-0"]:1.200642e-02[&length_mean=1.36239052e-02,length_median=1.20064200e-02,length_95%HPD={9.21300800e-04,2.97851800e-02}],241[&prob=1.00000000e+00,prob_stddev=0.00000000e+00,prob_range={1.00000000e+00,1.00000000e+00},prob(percent)="100",prob+-sd="100+-0"]:3.442777e-03[&length_mean=5.02297770e-03,length_median=3.44277700e-03,length_95%HPD={3.41614100e-08,1.53472300e-02}],242[&prob=1.00000000e+00,prob_stddev=0.00000000e+00,prob_range={1.00000000e+00,1.00000000e+00},prob(percent)="100",prob+-sd="100+-0"]:3.853379e-03[&length_mean=5.68921102e-03,length_median=3.85337900e-03,length_95%HPD={1.61648000e-06,1.70837700e-02}],243[&prob=1.00000000e+00,prob_stddev=0.00000000e+00,prob_range={1.00000000e+00,1.00000000e+00},prob(percent)="100",prob+-sd="100+-0"]:3.941061e-03[&length_mean=5.66490847e-03,length_median=3.94106100e-03,length_95%HPD={1.83908000e-06,1.69842700e-02}])[&prob=9.85405405e-01,prob_stddev=6.11551811e-03,prob_range={9.81081081e-01,9.89729730e-01},prob(percent)="99",prob+-sd="99+-1"]:8.298118e-03[&length_mean=1.00418611e-02,length_median=8.29811800e-03,length_95%HPD={2.56560400e-04,2.40730700e-02}])[&prob=9.99459459e-01,prob_stddev=7.64439763e-04,prob_range={9.98918919e-01,1.00000000e+00},prob(percent)="100",prob+-sd="100+-0"]:1.838198e-02[&length_mean=1.98677171e-02,length_median=1.83819800e-02,length_95%HPD={3.78992900e-03,3.93506500e-02}])[&prob=7.82162162e-01,prob_stddev=8.40883740e-03,prob_range={7.76216216e-01,7.88108108e-01},prob(percent)="78",prob+-sd="78+-1"]:1.385840e-02[&length_mean=1.65514685e-02,length_median=1.38584000e-02,length_95%HPD={2.32129600e-05,4.09656400e-02}])[&prob=1.00000000e+00,prob_stddev=0.00000000e+00,prob_range={1.00000000e+00,1.00000000e+00},prob(percent)="100",prob+-sd="100+-0"]:1.273783e-01[&length_mean=1.29503008e-01,length_median=1.27378300e-01,length_95%HPD={7.03134700e-02,1.96326200e-01}])[&prob=8.31621622e-01,prob_stddev=1.49065754e-02,prob_range={8.21081081e-01,8.42162162e-01},prob(percent)="83",prob+-sd="83+-1"]:7.820070e-02[&length_mean=8.30699214e-02,length_median=7.82007000e-02,length_95%HPD={4.74426800e-03,1.64472000e-01}],(((245[&prob=1.00000000e+00,prob_stddev=0.00000000e+00,prob_range={1.00000000e+00,1.00000000e+00},prob(percent)="100",prob+-sd="100+-0"]:3.543759e-02[&length_mean=3.72047756e-02,length_median=3.54375900e-02,length_95%HPD={1.04813800e-02,6.50114900e-02}],246[&prob=1.00000000e+00,prob_stddev=0.00000000e+00,prob_range={1.00000000e+00,1.00000000e+00},prob(percent)="100",prob+-sd="100+-0"]:4.108878e-03[&length_mean=5.89875921e-03,length_median=4.10887800e-03,length_95%HPD={6.72434400e-06,1.75069600e-02}])[&prob=1.00000000e+00,prob_stddev=0.00000000e+00,prob_range={1.00000000e+00,1.00000000e+00},prob(percent)="100",prob+-sd="100+-0"]:4.052374e-02[&length_mean=4.28853563e-02,length_median=4.05237400e-02,length_95%HPD={1.26843400e-02,8.06104300e-02}],(((247[&prob=1.00000000e+00,prob_stddev=0.00000000e+00,prob_range={1.00000000e+00,1.00000000e+00},prob(percent)="100",prob+-sd="100+-0"]:1.322941e-02[&length_mean=1.48883959e-02,length_median=1.32294100e-02,length_95%HPD={1.52067800e-03,3.15352300e-02}],251[&prob=1.00000000e+00,prob_stddev=0.00000000e+00,prob_range={1.00000000e+00,1.00000000e+00},prob(percent)="100",prob+-sd="100+-0"]:8.040979e-03[&length_mean=9.63070412e-03,length_median=8.04097900e-03,length_95%HPD={6.83213400e-05,2.32305800e-02}])[&prob=9.77297297e-01,prob_stddev=7.64439763e-04,prob_range={9.76756757e-01,9.77837838e-01},prob(percent)="98",prob+-sd="98+-0"]:1.746131e-02[&length_mean=1.91038514e-02,length_median=1.74613100e-02,length_95%HPD={1.49361900e-03,3.87825100e-02}],248[&prob=1.00000000e+00,prob_stddev=0.00000000e+00,prob_range={1.00000000e+00,1.00000000e+00},prob(percent)="100",prob+-sd="100+-0"]:3.481849e-03[&length_mean=4.94104501e-03,length_median=3.48184900e-03,length_95%HPD={1.10320800e-06,1.46854200e-02}],249[&prob=1.00000000e+00,prob_stddev=0.00000000e+00,prob_range={1.00000000e+00,1.00000000e+00},prob(percent)="100",prob+-sd="100+-0"]:8.136336e-03[&length_mean=9.78765016e-03,length_median=8.13633600e-03,length_95%HPD={4.87973400e-04,2.38179800e-02}])[&prob=5.54054054e-01,prob_stddev=7.64439763e-04,prob_range={5.53513514e-01,5.54594595e-01},prob(percent)="55",prob+-sd="55+-0"]:7.297649e-03[&length_mean=8.95913663e-03,length_median=7.29764900e-03,length_95%HPD={1.68313500e-06,2.26367400e-02}],250[&prob=1.00000000e+00,prob_stddev=0.00000000e+00,prob_range={1.00000000e+00,1.00000000e+00},prob(percent)="100",prob+-sd="100+-0"]:5.756686e-03[&length_mean=7.51078877e-03,length_median=5.75668600e-03,length_95%HPD={1.37978700e-06,2.05854900e-02}])[&prob=1.00000000e+00,prob_stddev=0.00000000e+00,prob_range={1.00000000e+00,1.00000000e+00},prob(percent)="100",prob+-sd="100+-0"]:1.187930e-01[&length_mean=1.21005791e-01,length_median=1.18793000e-01,length_95%HPD={6.66740800e-02,1.78744500e-01}])[&prob=8.00270270e-01,prob_stddev=3.82219882e-04,prob_range={8.00000000e-01,8.00540541e-01},prob(percent)="80",prob+-sd="80+-0"]:5.254605e-02[&length_mean=5.49582678e-02,length_median=5.25460500e-02,length_95%HPD={1.06929900e-02,1.03753400e-01}],((252[&prob=1.00000000e+00,prob_stddev=0.00000000e+00,prob_range={1.00000000e+00,1.00000000e+00},prob(percent)="100",prob+-sd="100+-0"]:3.865971e-03[&length_mean=5.61103960e-03,length_median=3.86597100e-03,length_95%HPD={5.66094200e-07,1.64427400e-02}],253[&prob=1.00000000e+00,prob_stddev=0.00000000e+00,prob_range={1.00000000e+00,1.00000000e+00},prob(percent)="100",prob+-sd="100+-0"]:5.281151e-03[&length_mean=7.39283058e-03,length_median=5.28115100e-03,length_95%HPD={9.98027200e-07,2.11020800e-02}])[&prob=8.98918919e-01,prob_stddev=3.05775905e-03,prob_range={8.96756757e-01,9.01081081e-01},prob(percent)="90",prob+-sd="90+-0"]:2.781319e-02[&length_mean=3.24545166e-02,length_median=2.78131900e-02,length_95%HPD={2.27834300e-05,7.71178000e-02}],254[&prob=1.00000000e+00,prob_stddev=0.00000000e+00,prob_range={1.00000000e+00,1.00000000e+00},prob(percent)="100",prob+-sd="100+-0"]:7.879172e-02[&length_mean=8.37663963e-02,length_median=7.87917200e-02,length_95%HPD={2.47206100e-02,1.53673800e-01}])[&prob=1.00000000e+00,prob_stddev=0.00000000e+00,prob_range={1.00000000e+00,1.00000000e+00},prob(percent)="100",prob+-sd="100+-0"]:2.625757e-01[&length_mean=2.65035832e-01,length_median=2.62575700e-01,length_95%HPD={1.61293000e-01,3.63270300e-01}])[&prob=9.98918919e-01,prob_stddev=7.64439763e-04,prob_range={9.98378378e-01,9.99459459e-01},prob(percent)="100",prob+-sd="100+-0"]:1.787604e-01[&length_mean=1.82872100e-01,length_median=1.78760400e-01,length_95%HPD={7.53230700e-02,3.04254700e-01}])[&prob=1.00000000e+00,prob_stddev=0.00000000e+00,prob_range={1.00000000e+00,1.00000000e+00},prob(percent)="100",prob+-sd="100+-0"]:4.219044e-01[&length_mean=4.29226324e-01,length_median=4.21904400e-01,length_95%HPD={2.22109600e-01,6.20566500e-01}],255[&prob=1.00000000e+00,prob_stddev=0.00000000e+00,prob_range={1.00000000e+00,1.00000000e+00},prob(percent)="100",prob+-sd="100+-0"]:6.595685e-01[&length_mean=6.62835975e-01,length_median=6.59568500e-01,length_95%HPD={4.44081700e-01,8.76507800e-01}])[&prob=9.95675676e-01,prob_stddev=0.00000000e+00,prob_range={9.95675676e-01,9.95675676e-01},prob(percent)="100",prob+-sd="100+-0"]:3.702560e-01[&length_mean=3.74211847e-01,length_median=3.70256000e-01,length_95%HPD={1.60201900e-01,6.14586800e-01}],(259[&prob=1.00000000e+00,prob_stddev=0.00000000e+00,prob_range={1.00000000e+00,1.00000000e+00},prob(percent)="100",prob+-sd="100+-0"]:1.279284e-01[&length_mean=1.27985074e-01,length_median=1.27928400e-01,length_95%HPD={4.66249100e-02,2.11735300e-01}],(260[&prob=1.00000000e+00,prob_stddev=0.00000000e+00,prob_range={1.00000000e+00,1.00000000e+00},prob(percent)="100",prob+-sd="100+-0"]:3.479368e-03[&length_mean=4.92681828e-03,length_median=3.47936800e-03,length_95%HPD={1.48599200e-06,1.46731900e-02}],261[&prob=1.00000000e+00,prob_stddev=0.00000000e+00,prob_range={1.00000000e+00,1.00000000e+00},prob(percent)="100",prob+-sd="100+-0"]:3.311305e-03[&length_mean=4.88231820e-03,length_median=3.31130500e-03,length_95%HPD={5.08060500e-07,1.50841000e-02}],262[&prob=1.00000000e+00,prob_stddev=0.00000000e+00,prob_range={1.00000000e+00,1.00000000e+00},prob(percent)="100",prob+-sd="100+-0"]:3.624352e-03[&length_mean=5.19649404e-03,length_median=3.62435200e-03,length_95%HPD={2.24687100e-07,1.49184200e-02}])[&prob=8.35675676e-01,prob_stddev=2.44620724e-02,prob_range={8.18378378e-01,8.52972973e-01},prob(percent)="84",prob+-sd="84+-2"]:4.482916e-02[&length_mean=4.98256915e-02,length_median=4.48291600e-02,length_95%HPD={5.40707200e-05,1.11268300e-01}])[&prob=1.00000000e+00,prob_stddev=0.00000000e+00,prob_range={1.00000000e+00,1.00000000e+00},prob(percent)="100",prob+-sd="100+-0"]:1.263150e+00[&length_mean=1.27565555e+00,length_median=1.26315000e+00,length_95%HPD={9.13665300e-01,1.61916600e+00}])[&prob=5.30270270e-01,prob_stddev=4.12797472e-02,prob_range={5.01081081e-01,5.59459459e-01},prob(percent)="53",prob+-sd="53+-4"]:1.388512e-01[&length_mean=1.42204279e-01,length_median=1.38851200e-01,length_95%HPD={3.23128800e-02,2.62723300e-01}],233[&prob=1.00000000e+00,prob_stddev=0.00000000e+00,prob_range={1.00000000e+00,1.00000000e+00},prob(percent)="100",prob+-sd="100+-0"]:8.401887e-01[&length_mean=8.42525234e-01,length_median=8.40188700e-01,length_95%HPD={6.14377600e-01,1.07476700e+00}],(256[&prob=1.00000000e+00,prob_stddev=0.00000000e+00,prob_range={1.00000000e+00,1.00000000e+00},prob(percent)="100",prob+-sd="100+-0"]:5.164065e-01[&length_mean=5.21972486e-01,length_median=5.16406500e-01,length_95%HPD={3.00202800e-01,7.37823800e-01}],257[&prob=1.00000000e+00,prob_stddev=0.00000000e+00,prob_range={1.00000000e+00,1.00000000e+00},prob(percent)="100",prob+-sd="100+-0"]:6.308698e-01[&length_mean=6.41345613e-01,length_median=6.30869800e-01,length_95%HPD={4.07195600e-01,9.04006000e-01}])[&prob=1.00000000e+00,prob_stddev=0.00000000e+00,prob_range={1.00000000e+00,1.00000000e+00},prob(percent)="100",prob+-sd="100+-0"]:5.503500e-01[&length_mean=5.59591996e-01,length_median=5.50350000e-01,length_95%HPD={3.08180600e-01,8.25803600e-01}])[&prob=9.65675676e-01,prob_stddev=3.32531297e-02,prob_range={9.42162162e-01,9.89189189e-01},prob(percent)="97",prob+-sd="97+-3"]:2.721655e-01[&length_mean=2.69675441e-01,length_median=2.72165500e-01,length_95%HPD={7.61482500e-02,4.34653400e-01}],((((100[&prob=1.00000000e+00,prob_stddev=0.00000000e+00,prob_range={1.00000000e+00,1.00000000e+00},prob(percent)="100",prob+-sd="100+-0"]:1.722383e-01[&length_mean=1.99039448e-01,length_median=1.72238300e-01,length_95%HPD={7.87259000e-03,4.54866400e-01}],196[&prob=1.00000000e+00,prob_stddev=0.00000000e+00,prob_range={1.00000000e+00,1.00000000e+00},prob(percent)="100",prob+-sd="100+-0"]:3.118070e-01[&length_mean=3.32210802e-01,length_median=3.11807000e-01,length_95%HPD={2.98912900e-02,6.50559200e-01}])[&prob=9.87027027e-01,prob_stddev=1.83465543e-02,prob_range={9.74054054e-01,1.00000000e+00},prob(percent)="99",prob+-sd="99+-2"]:5.837826e-01[&length_mean=5.88674762e-01,length_median=5.83782600e-01,length_95%HPD={2.93254500e-01,9.06343800e-01}],(101[&prob=1.00000000e+00,prob_stddev=0.00000000e+00,prob_range={1.00000000e+00,1.00000000e+00},prob(percent)="100",prob+-sd="100+-0"]:1.661943e-01[&length_mean=1.69495623e-01,length_median=1.66194300e-01,length_95%HPD={9.37528700e-02,2.62567800e-01}],102[&prob=1.00000000e+00,prob_stddev=0.00000000e+00,prob_range={1.00000000e+00,1.00000000e+00},prob(percent)="100",prob+-sd="100+-0"]:1.058813e-01[&length_mean=1.09420549e-01,length_median=1.05881300e-01,length_95%HPD={3.60142600e-02,1.78989000e-01}])[&prob=9.96756757e-01,prob_stddev=0.00000000e+00,prob_range={9.96756757e-01,9.96756757e-01},prob(percent)="100",prob+-sd="100+-0"]:1.681674e-01[&length_mean=1.74258470e-01,length_median=1.68167400e-01,length_95%HPD={5.00768600e-02,3.08093400e-01}])[&prob=9.12162162e-01,prob_stddev=4.93063647e-02,prob_range={8.77297297e-01,9.47027027e-01},prob(percent)="91",prob+-sd="91+-5"]:1.388709e-01[&length_mean=1.41840287e-01,length_median=1.38870900e-01,length_95%HPD={3.23556600e-02,2.56995800e-01}],((103[&prob=1.00000000e+00,prob_stddev=0.00000000e+00,prob_range={1.00000000e+00,1.00000000e+00},prob(percent)="100",prob+-sd="100+-0"]:3.350716e-03[&length_mean=4.81950587e-03,length_median=3.35071600e-03,length_95%HPD={2.01543800e-07,1.43871100e-02}],104[&prob=1.00000000e+00,prob_stddev=0.00000000e+00,prob_range={1.00000000e+00,1.00000000e+00},prob(percent)="100",prob+-sd="100+-0"]:3.468082e-03[&length_mean=4.81728896e-03,length_median=3.46808200e-03,length_95%HPD={1.42958300e-06,1.40610000e-02}],105[&prob=1.00000000e+00,prob_stddev=0.00000000e+00,prob_range={1.00000000e+00,1.00000000e+00},prob(percent)="100",prob+-sd="100+-0"]:7.891489e-03[&length_mean=9.29695943e-03,length_median=7.89148900e-03,length_95%HPD={6.64036400e-05,2.23870300e-02}],106[&prob=1.00000000e+00,prob_stddev=0.00000000e+00,prob_range={1.00000000e+00,1.00000000e+00},prob(percent)="100",prob+-sd="100+-0"]:2.448599e-01[&length_mean=2.47534930e-01,length_median=2.44859900e-01,length_95%HPD={1.71607200e-01,3.30572500e-01}])[&prob=1.00000000e+00,prob_stddev=0.00000000e+00,prob_range={1.00000000e+00,1.00000000e+00},prob(percent)="100",prob+-sd="100+-0"]:2.093263e-01[&length_mean=2.12289905e-01,length_median=2.09326300e-01,length_95%HPD={1.37067100e-01,2.99423300e-01}],(107[&prob=1.00000000e+00,prob_stddev=0.00000000e+00,prob_range={1.00000000e+00,1.00000000e+00},prob(percent)="100",prob+-sd="100+-0"]:5.930160e-03[&length_mean=7.54953320e-03,length_median=5.93016000e-03,length_95%HPD={1.11443900e-05,2.03995000e-02}],108[&prob=1.00000000e+00,prob_stddev=0.00000000e+00,prob_range={1.00000000e+00,1.00000000e+00},prob(percent)="100",prob+-sd="100+-0"]:5.502038e-03[&length_mean=7.48459460e-03,length_median=5.50203800e-03,length_95%HPD={4.51706900e-06,2.13144400e-02}])[&prob=1.00000000e+00,prob_stddev=0.00000000e+00,prob_range={1.00000000e+00,1.00000000e+00},prob(percent)="100",prob+-sd="100+-0"]:1.600769e-01[&length_mean=1.62198501e-01,length_median=1.60076900e-01,length_95%HPD={9.01470200e-02,2.30193000e-01}])[&prob=9.97837838e-01,prob_stddev=7.64439763e-04,prob_range={9.97297297e-01,9.98378378e-01},prob(percent)="100",prob+-sd="100+-0"]:8.254432e-02[&length_mean=8.43654106e-02,length_median=8.25443200e-02,length_95%HPD={2.25261700e-02,1.44214600e-01}],((((109[&prob=1.00000000e+00,prob_stddev=0.00000000e+00,prob_range={1.00000000e+00,1.00000000e+00},prob(percent)="100",prob+-sd="100+-0"]:1.135888e-02[&length_mean=1.31999080e-02,length_median=1.13588800e-02,length_95%HPD={2.43833000e-05,3.04838200e-02}],110[&prob=1.00000000e+00,prob_stddev=0.00000000e+00,prob_range={1.00000000e+00,1.00000000e+00},prob(percent)="100",prob+-sd="100+-0"]:1.630256e-02[&length_mean=1.77829537e-02,length_median=1.63025600e-02,length_95%HPD={1.86916300e-03,3.77128900e-02}])[&prob=1.00000000e+00,prob_stddev=0.00000000e+00,prob_range={1.00000000e+00,1.00000000e+00},prob(percent)="100",prob+-sd="100+-0"]:1.901329e-01[&length_mean=1.93185253e-01,length_median=1.90132900e-01,length_95%HPD={9.28660100e-02,2.90654500e-01}],(((145[&prob=1.00000000e+00,prob_stddev=0.00000000e+00,prob_range={1.00000000e+00,1.00000000e+00},prob(percent)="100",prob+-sd="100+-0"]:1.161725e-02[&length_mean=1.36830352e-02,length_median=1.16172500e-02,length_95%HPD={3.07590700e-04,3.22323300e-02}],146[&prob=1.00000000e+00,prob_stddev=0.00000000e+00,prob_range={1.00000000e+00,1.00000000e+00},prob(percent)="100",prob+-sd="100+-0"]:3.907890e-03[&length_mean=5.69630322e-03,length_median=3.90789000e-03,length_95%HPD={1.15449600e-06,1.69297000e-02}])[&prob=1.00000000e+00,prob_stddev=0.00000000e+00,prob_range={1.00000000e+00,1.00000000e+00},prob(percent)="100",prob+-sd="100+-0"]:5.686677e-02[&length_mean=5.94980899e-02,length_median=5.68667700e-02,length_95%HPD={1.83760400e-02,1.04492600e-01}],(147[&prob=1.00000000e+00,prob_stddev=0.00000000e+00,prob_range={1.00000000e+00,1.00000000e+00},prob(percent)="100",prob+-sd="100+-0"]:4.777447e-03[&length_mean=6.99493810e-03,length_median=4.77744700e-03,length_95%HPD={3.90639800e-06,2.11031100e-02}],148[&prob=1.00000000e+00,prob_stddev=0.00000000e+00,prob_range={1.00000000e+00,1.00000000e+00},prob(percent)="100",prob+-sd="100+-0"]:4.110445e-03[&length_mean=5.79065606e-03,length_median=4.11044500e-03,length_95%HPD={4.31533200e-06,1.69393200e-02}])[&prob=1.00000000e+00,prob_stddev=0.00000000e+00,prob_range={1.00000000e+00,1.00000000e+00},prob(percent)="100",prob+-sd="100+-0"]:8.216490e-02[&length_mean=8.47164291e-02,length_median=8.21649000e-02,length_95%HPD={3.50883800e-02,1.37288000e-01}])[&prob=1.00000000e+00,prob_stddev=0.00000000e+00,prob_range={1.00000000e+00,1.00000000e+00},prob(percent)="100",prob+-sd="100+-0"]:1.775285e-01[&length_mean=1.80917186e-01,length_median=1.77528500e-01,length_95%HPD={8.45431900e-02,2.90944000e-01}],(149[&prob=1.00000000e+00,prob_stddev=0.00000000e+00,prob_range={1.00000000e+00,1.00000000e+00},prob(percent)="100",prob+-sd="100+-0"]:1.557630e-01[&length_mean=1.58478775e-01,length_median=1.55763000e-01,length_95%HPD={7.63384400e-02,2.48308000e-01}],(150[&prob=1.00000000e+00,prob_stddev=0.00000000e+00,prob_range={1.00000000e+00,1.00000000e+00},prob(percent)="100",prob+-sd="100+-0"]:3.462273e-03[&length_mean=4.88869683e-03,length_median=3.46227300e-03,length_95%HPD={8.97880700e-07,1.44582900e-02}],151[&prob=1.00000000e+00,prob_stddev=0.00000000e+00,prob_range={1.00000000e+00,1.00000000e+00},prob(percent)="100",prob+-sd="100+-0"]:3.410858e-03[&length_mean=4.93691952e-03,length_median=3.41085800e-03,length_95%HPD={3.80598300e-07,1.50567600e-02}])[&prob=1.00000000e+00,prob_stddev=0.00000000e+00,prob_range={1.00000000e+00,1.00000000e+00},prob(percent)="100",prob+-sd="100+-0"]:3.191401e-01[&length_mean=3.23088598e-01,length_median=3.19140100e-01,length_95%HPD={2.02447400e-01,4.41441700e-01}])[&prob=9.38918919e-01,prob_stddev=9.93771692e-03,prob_range={9.31891892e-01,9.45945946e-01},prob(percent)="94",prob+-sd="94+-1"]:1.241332e-01[&length_mean=1.28479758e-01,length_median=1.24133200e-01,length_95%HPD={4.35589400e-02,2.31662700e-01}])[&prob=1.00000000e+00,prob_stddev=0.00000000e+00,prob_range={1.00000000e+00,1.00000000e+00},prob(percent)="100",prob+-sd="100+-0"]:4.817092e-01[&length_mean=4.86301800e-01,length_median=4.81709200e-01,length_95%HPD={3.34363200e-01,6.42653300e-01}])[&prob=9.91621622e-01,prob_stddev=4.96885846e-03,prob_range={9.88108108e-01,9.95135135e-01},prob(percent)="99",prob+-sd="99+-0"]:1.341581e-01[&length_mean=1.37628529e-01,length_median=1.34158100e-01,length_95%HPD={4.54098600e-02,2.45185000e-01}],(169[&prob=1.00000000e+00,prob_stddev=0.00000000e+00,prob_range={1.00000000e+00,1.00000000e+00},prob(percent)="100",prob+-sd="100+-0"]:3.771003e-03[&length_mean=5.19040646e-03,length_median=3.77100300e-03,length_95%HPD={3.19745800e-06,1.49361800e-02}],170[&prob=1.00000000e+00,prob_stddev=0.00000000e+00,prob_range={1.00000000e+00,1.00000000e+00},prob(percent)="100",prob+-sd="100+-0"]:5.266776e-02[&length_mean=5.37822625e-02,length_median=5.26677600e-02,length_95%HPD={2.43662100e-02,8.33898300e-02}])[&prob=1.00000000e+00,prob_stddev=0.00000000e+00,prob_range={1.00000000e+00,1.00000000e+00},prob(percent)="100",prob+-sd="100+-0"]:4.538673e-01[&length_mean=4.57152696e-01,length_median=4.53867300e-01,length_95%HPD={3.26835100e-01,5.89560100e-01}])[&prob=6.30000000e-01,prob_stddev=1.03199368e-02,prob_range={6.22702703e-01,6.37297297e-01},prob(percent)="63",prob+-sd="63+-1"]:4.095058e-02[&length_mean=4.57918052e-02,length_median=4.09505800e-02,length_95%HPD={3.52857400e-05,9.87738700e-02}],((((((111[&prob=1.00000000e+00,prob_stddev=0.00000000e+00,prob_range={1.00000000e+00,1.00000000e+00},prob(percent)="100",prob+-sd="100+-0"]:7.551861e-03[&length_mean=9.12709573e-03,length_median=7.55186100e-03,length_95%HPD={1.18814100e-04,2.22684400e-02}],112[&prob=1.00000000e+00,prob_stddev=0.00000000e+00,prob_range={1.00000000e+00,1.00000000e+00},prob(percent)="100",prob+-sd="100+-0"]:3.226420e-03[&length_mean=4.67107076e-03,length_median=3.22642000e-03,length_95%HPD={1.12694800e-07,1.36288100e-02}],113[&prob=1.00000000e+00,prob_stddev=0.00000000e+00,prob_range={1.00000000e+00,1.00000000e+00},prob(percent)="100",prob+-sd="100+-0"]:3.008310e-03[&length_mean=4.50172438e-03,length_median=3.00831000e-03,length_95%HPD={1.21672500e-06,1.34677600e-02}],114[&prob=1.00000000e+00,prob_stddev=0.00000000e+00,prob_range={1.00000000e+00,1.00000000e+00},prob(percent)="100",prob+-sd="100+-0"]:7.736215e-03[&length_mean=9.33424087e-03,length_median=7.73621500e-03,length_95%HPD={2.82805600e-04,2.23827300e-02}],115[&prob=1.00000000e+00,prob_stddev=0.00000000e+00,prob_range={1.00000000e+00,1.00000000e+00},prob(percent)="100",prob+-sd="100+-0"]:7.858464e-03[&length_mean=9.45885562e-03,length_median=7.85846400e-03,length_95%HPD={7.41147400e-05,2.21288100e-02}],116[&prob=1.00000000e+00,prob_stddev=0.00000000e+00,prob_range={1.00000000e+00,1.00000000e+00},prob(percent)="100",prob+-sd="100+-0"]:2.651836e-02[&length_mean=2.91448378e-02,length_median=2.65183600e-02,length_95%HPD={5.84959700e-03,5.85768500e-02}],117[&prob=1.00000000e+00,prob_stddev=0.00000000e+00,prob_range={1.00000000e+00,1.00000000e+00},prob(percent)="100",prob+-sd="100+-0"]:1.693134e-02[&length_mean=1.83685213e-02,length_median=1.69313400e-02,length_95%HPD={2.86810500e-03,3.58218900e-02}],120[&prob=1.00000000e+00,prob_stddev=0.00000000e+00,prob_range={1.00000000e+00,1.00000000e+00},prob(percent)="100",prob+-sd="100+-0"]:1.741750e-02[&length_mean=1.89720978e-02,length_median=1.74175000e-02,length_95%HPD={3.25945200e-03,3.75475900e-02}])[&prob=9.61621622e-01,prob_stddev=0.00000000e+00,prob_range={9.61621622e-01,9.61621622e-01},prob(percent)="96",prob+-sd="96+-0"]:1.655831e-02[&length_mean=1.83098089e-02,length_median=1.65583100e-02,length_95%HPD={2.92881000e-03,3.86694400e-02}],(118[&prob=1.00000000e+00,prob_stddev=0.00000000e+00,prob_range={1.00000000e+00,1.00000000e+00},prob(percent)="100",prob+-sd="100+-0"]:7.382241e-03[&length_mean=8.97032833e-03,length_median=7.38224100e-03,length_95%HPD={1.16944700e-06,2.16399200e-02}],119[&prob=1.00000000e+00,prob_stddev=0.00000000e+00,prob_range={1.00000000e+00,1.00000000e+00},prob(percent)="100",prob+-sd="100+-0"]:8.587593e-03[&length_mean=1.01608983e-02,length_median=8.58759300e-03,length_95%HPD={3.14452000e-04,2.39613200e-02}])[&prob=9.99729730e-01,prob_stddev=3.82219882e-04,prob_range={9.99459459e-01,1.00000000e+00},prob(percent)="100",prob+-sd="100+-0"]:2.223180e-02[&length_mean=2.38845177e-02,length_median=2.22318000e-02,length_95%HPD={4.38295500e-03,4.66165900e-02}])[&prob=1.00000000e+00,prob_stddev=0.00000000e+00,prob_range={1.00000000e+00,1.00000000e+00},prob(percent)="100",prob+-sd="100+-0"]:1.720412e-01[&length_mean=1.73814597e-01,length_median=1.72041200e-01,length_95%HPD={1.00288500e-01,2.50474800e-01}],((121[&prob=1.00000000e+00,prob_stddev=0.00000000e+00,prob_range={1.00000000e+00,1.00000000e+00},prob(percent)="100",prob+-sd="100+-0"]:3.437745e-03[&length_mean=5.00106323e-03,length_median=3.43774500e-03,length_95%HPD={1.24053100e-06,1.50760400e-02}],123[&prob=1.00000000e+00,prob_stddev=0.00000000e+00,prob_range={1.00000000e+00,1.00000000e+00},prob(percent)="100",prob+-sd="100+-0"]:8.497983e-02[&length_mean=8.71636573e-02,length_median=8.49798300e-02,length_95%HPD={4.99671100e-02,1.31907400e-01}])[&prob=9.36216216e-01,prob_stddev=0.00000000e+00,prob_range={9.36216216e-01,9.36216216e-01},prob(percent)="94",prob+-sd="94+-0"]:8.010641e-03[&length_mean=9.61056371e-03,length_median=8.01064100e-03,length_95%HPD={1.98635000e-04,2.35996800e-02}],122[&prob=1.00000000e+00,prob_stddev=0.00000000e+00,prob_range={1.00000000e+00,1.00000000e+00},prob(percent)="100",prob+-sd="100+-0"]:3.532187e-03[&length_mean=5.16755105e-03,length_median=3.53218700e-03,length_95%HPD={4.69427100e-07,1.59135700e-02}])[&prob=1.00000000e+00,prob_stddev=0.00000000e+00,prob_range={1.00000000e+00,1.00000000e+00},prob(percent)="100",prob+-sd="100+-0"]:1.733109e-01[&length_mean=1.75652069e-01,length_median=1.73310900e-01,length_95%HPD={1.09514500e-01,2.52853400e-01}])[&prob=8.84054054e-01,prob_stddev=4.20441870e-03,prob_range={8.81081081e-01,8.87027027e-01},prob(percent)="88",prob+-sd="88+-0"]:9.323108e-02[&length_mean=9.55584597e-02,length_median=9.32310800e-02,length_95%HPD={3.57594600e-02,1.64216000e-01}],((126[&prob=1.00000000e+00,prob_stddev=0.00000000e+00,prob_range={1.00000000e+00,1.00000000e+00},prob(percent)="100",prob+-sd="100+-0"]:3.721585e-03[&length_mean=5.39912032e-03,length_median=3.72158500e-03,length_95%HPD={5.65279200e-06,1.63043700e-02}],127[&prob=1.00000000e+00,prob_stddev=0.00000000e+00,prob_range={1.00000000e+00,1.00000000e+00},prob(percent)="100",prob+-sd="100+-0"]:2.071201e-02[&length_mean=2.27078667e-02,length_median=2.07120100e-02,length_95%HPD={4.58932000e-03,4.59626600e-02}])[&prob=1.00000000e+00,prob_stddev=0.00000000e+00,prob_range={1.00000000e+00,1.00000000e+00},prob(percent)="100",prob+-sd="100+-0"]:2.785921e-01[&length_mean=2.80933982e-01,length_median=2.78592100e-01,length_95%HPD={1.89714900e-01,3.76016200e-01}],128[&prob=1.00000000e+00,prob_stddev=0.00000000e+00,prob_range={1.00000000e+00,1.00000000e+00},prob(percent)="100",prob+-sd="100+-0"]:1.620867e-01[&length_mean=1.64272720e-01,length_median=1.62086700e-01,length_95%HPD={9.65981500e-02,2.38284600e-01}])[&prob=9.98378378e-01,prob_stddev=0.00000000e+00,prob_range={9.98378378e-01,9.98378378e-01},prob(percent)="100",prob+-sd="100+-0"]:9.115080e-02[&length_mean=9.41048073e-02,length_median=9.11508000e-02,length_95%HPD={3.53653800e-02,1.56752800e-01}])[&prob=8.11081081e-01,prob_stddev=1.49065754e-02,prob_range={8.00540541e-01,8.21621622e-01},prob(percent)="81",prob+-sd="81+-1"]:6.733469e-02[&length_mean=7.01277696e-02,length_median=6.73346900e-02,length_95%HPD={1.49578100e-02,1.35165100e-01}],((124[&prob=1.00000000e+00,prob_stddev=0.00000000e+00,prob_range={1.00000000e+00,1.00000000e+00},prob(percent)="100",prob+-sd="100+-0"]:7.667297e-03[&length_mean=9.27705091e-03,length_median=7.66729700e-03,length_95%HPD={1.99846400e-05,2.26585600e-02}],125[&prob=1.00000000e+00,prob_stddev=0.00000000e+00,prob_range={1.00000000e+00,1.00000000e+00},prob(percent)="100",prob+-sd="100+-0"]:4.055332e-03[&length_mean=5.65901628e-03,length_median=4.05533200e-03,length_95%HPD={5.37488000e-06,1.64377300e-02}])[&prob=1.00000000e+00,prob_stddev=0.00000000e+00,prob_range={1.00000000e+00,1.00000000e+00},prob(percent)="100",prob+-sd="100+-0"]:2.793109e-01[&length_mean=2.83502802e-01,length_median=2.79310900e-01,length_95%HPD={1.60744700e-01,4.05605000e-01}],183[&prob=1.00000000e+00,prob_stddev=0.00000000e+00,prob_range={1.00000000e+00,1.00000000e+00},prob(percent)="100",prob+-sd="100+-0"]:6.126928e-01[&length_mean=6.17750675e-01,length_median=6.12692800e-01,length_95%HPD={4.39868100e-01,8.04976700e-01}])[&prob=9.89459459e-01,prob_stddev=1.18488163e-02,prob_range={9.81081081e-01,9.97837838e-01},prob(percent)="99",prob+-sd="99+-1"]:1.721374e-01[&length_mean=1.76034964e-01,length_median=1.72137400e-01,length_95%HPD={7.72333700e-02,2.89563000e-01}])[&prob=5.60810811e-01,prob_stddev=1.10843766e-02,prob_range={5.52972973e-01,5.68648649e-01},prob(percent)="56",prob+-sd="56+-1"]:5.190986e-02[&length_mean=5.95555834e-02,length_median=5.19098600e-02,length_95%HPD={1.30548800e-04,1.35098000e-01}],((((187[&prob=1.00000000e+00,prob_stddev=0.00000000e+00,prob_range={1.00000000e+00,1.00000000e+00},prob(percent)="100",prob+-sd="100+-0"]:8.786676e-02[&length_mean=8.96563668e-02,length_median=8.78667600e-02,length_95%HPD={4.57008900e-02,1.35057300e-01}],188[&prob=1.00000000e+00,prob_stddev=0.00000000e+00,prob_range={1.00000000e+00,1.00000000e+00},prob(percent)="100",prob+-sd="100+-0"]:1.191307e-02[&length_mean=1.41327093e-02,length_median=1.19130700e-02,length_95%HPD={1.67479400e-05,3.46355200e-02}])[&prob=1.00000000e+00,prob_stddev=0.00000000e+00,prob_range={1.00000000e+00,1.00000000e+00},prob(percent)="100",prob+-sd="100+-0"]:1.710056e-01[&length_mean=1.74764352e-01,length_median=1.71005600e-01,length_95%HPD={8.16460200e-02,2.69873300e-01}],(189[&prob=1.00000000e+00,prob_stddev=0.00000000e+00,prob_range={1.00000000e+00,1.00000000e+00},prob(percent)="100",prob+-sd="100+-0"]:2.551888e-01[&length_mean=2.57392968e-01,length_median=2.55188800e-01,length_95%HPD={1.68350200e-01,3.56060400e-01}],190[&prob=1.00000000e+00,prob_stddev=0.00000000e+00,prob_range={1.00000000e+00,1.00000000e+00},prob(percent)="100",prob+-sd="100+-0"]:2.045677e-01[&length_mean=2.07411551e-01,length_median=2.04567700e-01,length_95%HPD={1.23829600e-01,3.03285900e-01}])[&prob=9.57297297e-01,prob_stddev=9.17327716e-03,prob_range={9.50810811e-01,9.63783784e-01},prob(percent)="96",prob+-sd="96+-1"]:1.024930e-01[&length_mean=1.06130028e-01,length_median=1.02493000e-01,length_95%HPD={2.30802900e-02,1.84967800e-01}])[&prob=9.98108108e-01,prob_stddev=3.82219882e-04,prob_range={9.97837838e-01,9.98378378e-01},prob(percent)="100",prob+-sd="100+-0"]:1.877029e-01[&length_mean=1.91513376e-01,length_median=1.87702900e-01,length_95%HPD={8.32409500e-02,3.07831600e-01}],191[&prob=1.00000000e+00,prob_stddev=0.00000000e+00,prob_range={1.00000000e+00,1.00000000e+00},prob(percent)="100",prob+-sd="100+-0"]:4.641505e-01[&length_mean=4.70259414e-01,length_median=4.64150500e-01,length_95%HPD={3.16896800e-01,6.28963300e-01}])[&prob=1.00000000e+00,prob_stddev=0.00000000e+00,prob_range={1.00000000e+00,1.00000000e+00},prob(percent)="100",prob+-sd="100+-0"]:2.581610e-01[&length_mean=2.62095609e-01,length_median=2.58161000e-01,length_95%HPD={1.20753000e-01,3.90373200e-01}],(((192[&prob=1.00000000e+00,prob_stddev=0.00000000e+00,prob_range={1.00000000e+00,1.00000000e+00},prob(percent)="100",prob+-sd="100+-0"]:7.836507e-03[&length_mean=9.50645631e-03,length_median=7.83650700e-03,length_95%HPD={7.73618600e-05,2.32423500e-02}],193[&prob=1.00000000e+00,prob_stddev=0.00000000e+00,prob_range={1.00000000e+00,1.00000000e+00},prob(percent)="100",prob+-sd="100+-0"]:3.298639e-03[&length_mean=4.83193995e-03,length_median=3.29863900e-03,length_95%HPD={1.76566200e-06,1.49451500e-02}])[&prob=1.00000000e+00,prob_stddev=0.00000000e+00,prob_range={1.00000000e+00,1.00000000e+00},prob(percent)="100",prob+-sd="100+-0"]:1.373407e-01[&length_mean=1.40127608e-01,length_median=1.37340700e-01,length_95%HPD={7.32626800e-02,2.10862100e-01}],195[&prob=1.00000000e+00,prob_stddev=0.00000000e+00,prob_range={1.00000000e+00,1.00000000e+00},prob(percent)="100",prob+-sd="100+-0"]:3.081582e-01[&length_mean=3.13843975e-01,length_median=3.08158200e-01,length_95%HPD={2.06939300e-01,4.44616400e-01}])[&prob=9.73243243e-01,prob_stddev=4.20441870e-03,prob_range={9.70270270e-01,9.76216216e-01},prob(percent)="97",prob+-sd="97+-0"]:6.787351e-02[&length_mean=7.12849108e-02,length_median=6.78735100e-02,length_95%HPD={1.34393700e-02,1.34232200e-01}],194[&prob=1.00000000e+00,prob_stddev=0.00000000e+00,prob_range={1.00000000e+00,1.00000000e+00},prob(percent)="100",prob+-sd="100+-0"]:1.060631e-01[&length_mean=1.08635115e-01,length_median=1.06063100e-01,length_95%HPD={4.60595500e-02,1.78160400e-01}])[&prob=1.00000000e+00,prob_stddev=0.00000000e+00,prob_range={1.00000000e+00,1.00000000e+00},prob(percent)="100",prob+-sd="100+-0"]:2.584064e-01[&length_mean=2.62211129e-01,length_median=2.58406400e-01,length_95%HPD={1.49180900e-01,3.77474800e-01}])[&prob=1.00000000e+00,prob_stddev=0.00000000e+00,prob_range={1.00000000e+00,1.00000000e+00},prob(percent)="100",prob+-sd="100+-0"]:2.519811e-01[&length_mean=2.54372191e-01,length_median=2.51981100e-01,length_95%HPD={1.42477000e-01,3.90579900e-01}])[&prob=9.22972973e-01,prob_stddev=1.18488163e-02,prob_range={9.14594595e-01,9.31351351e-01},prob(percent)="92",prob+-sd="92+-1"]:7.582220e-02[&length_mean=7.92191587e-02,length_median=7.58222000e-02,length_95%HPD={2.05338100e-02,1.45866000e-01}])[&prob=9.64324324e-01,prob_stddev=6.87995787e-03,prob_range={9.59459459e-01,9.69189189e-01},prob(percent)="96",prob+-sd="96+-1"]:7.778960e-02[&length_mean=8.06840683e-02,length_median=7.77896000e-02,length_95%HPD={1.47482900e-02,1.48879600e-01}],(((129[&prob=1.00000000e+00,prob_stddev=0.00000000e+00,prob_range={1.00000000e+00,1.00000000e+00},prob(percent)="100",prob+-sd="100+-0"]:9.602985e-03[&length_mean=1.14239119e-02,length_median=9.60298500e-03,length_95%HPD={2.19487300e-06,2.83676800e-02}],130[&prob=1.00000000e+00,prob_stddev=0.00000000e+00,prob_range={1.00000000e+00,1.00000000e+00},prob(percent)="100",prob+-sd="100+-0"]:1.107438e-02[&length_mean=1.26936131e-02,length_median=1.10743800e-02,length_95%HPD={1.47616800e-04,2.92346900e-02}])[&prob=1.00000000e+00,prob_stddev=0.00000000e+00,prob_range={1.00000000e+00,1.00000000e+00},prob(percent)="100",prob+-sd="100+-0"]:3.306304e-01[&length_mean=3.33272148e-01,length_median=3.30630400e-01,length_95%HPD={2.29587500e-01,4.50499300e-01}],(131[&prob=1.00000000e+00,prob_stddev=0.00000000e+00,prob_range={1.00000000e+00,1.00000000e+00},prob(percent)="100",prob+-sd="100+-0"]:3.444724e-03[&length_mean=4.98570325e-03,length_median=3.44472400e-03,length_95%HPD={2.36422000e-06,1.46176400e-02}],132[&prob=1.00000000e+00,prob_stddev=0.00000000e+00,prob_range={1.00000000e+00,1.00000000e+00},prob(percent)="100",prob+-sd="100+-0"]:3.410338e-03[&length_mean=4.88114632e-03,length_median=3.41033800e-03,length_95%HPD={1.69405700e-07,1.43738200e-02}])[&prob=1.00000000e+00,prob_stddev=0.00000000e+00,prob_range={1.00000000e+00,1.00000000e+00},prob(percent)="100",prob+-sd="100+-0"]:1.613654e-01[&length_mean=1.64009458e-01,length_median=1.61365400e-01,length_95%HPD={9.11597000e-02,2.46783200e-01}])[&prob=1.00000000e+00,prob_stddev=0.00000000e+00,prob_range={1.00000000e+00,1.00000000e+00},prob(percent)="100",prob+-sd="100+-0"]:1.599125e-01[&length_mean=1.63574689e-01,length_median=1.59912500e-01,length_95%HPD={7.68533900e-02,2.53539600e-01}],(((133[&prob=1.00000000e+00,prob_stddev=0.00000000e+00,prob_range={1.00000000e+00,1.00000000e+00},prob(percent)="100",prob+-sd="100+-0"]:3.293976e-03[&length_mean=4.78203918e-03,length_median=3.29397600e-03,length_95%HPD={5.31796900e-07,1.44153600e-02}],134[&prob=1.00000000e+00,prob_stddev=0.00000000e+00,prob_range={1.00000000e+00,1.00000000e+00},prob(percent)="100",prob+-sd="100+-0"]:3.374989e-03[&length_mean=4.70634908e-03,length_median=3.37498900e-03,length_95%HPD={8.14769500e-07,1.41676400e-02}])[&prob=1.00000000e+00,prob_stddev=0.00000000e+00,prob_range={1.00000000e+00,1.00000000e+00},prob(percent)="100",prob+-sd="100+-0"]:1.196987e-01[&length_mean=1.21923595e-01,length_median=1.19698700e-01,length_95%HPD={5.47531900e-02,1.93508500e-01}],(135[&prob=1.00000000e+00,prob_stddev=0.00000000e+00,prob_range={1.00000000e+00,1.00000000e+00},prob(percent)="100",prob+-sd="100+-0"]:3.380674e-03[&length_mean=4.84982256e-03,length_median=3.38067400e-03,length_95%HPD={1.03151300e-06,1.49649000e-02}],136[&prob=1.00000000e+00,prob_stddev=0.00000000e+00,prob_range={1.00000000e+00,1.00000000e+00},prob(percent)="100",prob+-sd="100+-0"]:7.938124e-03[&length_mean=9.41844747e-03,length_median=7.93812400e-03,length_95%HPD={4.07097700e-04,2.29244700e-02}])[&prob=1.00000000e+00,prob_stddev=0.00000000e+00,prob_range={1.00000000e+00,1.00000000e+00},prob(percent)="100",prob+-sd="100+-0"]:1.645216e-01[&length_mean=1.66910765e-01,length_median=1.64521600e-01,length_95%HPD={9.13646000e-02,2.43892700e-01}])[&prob=1.00000000e+00,prob_stddev=0.00000000e+00,prob_range={1.00000000e+00,1.00000000e+00},prob(percent)="100",prob+-sd="100+-0"]:2.326691e-01[&length_mean=2.35992959e-01,length_median=2.32669100e-01,length_95%HPD={1.30689900e-01,3.53598100e-01}],((((172[&prob=1.00000000e+00,prob_stddev=0.00000000e+00,prob_range={1.00000000e+00,1.00000000e+00},prob(percent)="100",prob+-sd="100+-0"]:3.179777e-03[&length_mean=4.49328671e-03,length_median=3.17977700e-03,length_95%HPD={8.52469200e-08,1.36603200e-02}],173[&prob=1.00000000e+00,prob_stddev=0.00000000e+00,prob_range={1.00000000e+00,1.00000000e+00},prob(percent)="100",prob+-sd="100+-0"]:3.158488e-03[&length_mean=4.53904492e-03,length_median=3.15848800e-03,length_95%HPD={1.62611600e-06,1.37451300e-02}])[&prob=1.00000000e+00,prob_stddev=0.00000000e+00,prob_range={1.00000000e+00,1.00000000e+00},prob(percent)="100",prob+-sd="100+-0"]:2.246043e-01[&length_mean=2.27678307e-01,length_median=2.24604300e-01,length_95%HPD={1.54030600e-01,3.04325100e-01}],174[&prob=1.00000000e+00,prob_stddev=0.00000000e+00,prob_range={1.00000000e+00,1.00000000e+00},prob(percent)="100",prob+-sd="100+-0"]:5.913304e-02[&length_mean=6.11903300e-02,length_median=5.91330400e-02,length_95%HPD={2.25161300e-02,1.06032500e-01}])[&prob=1.00000000e+00,prob_stddev=0.00000000e+00,prob_range={1.00000000e+00,1.00000000e+00},prob(percent)="100",prob+-sd="100+-0"]:7.927254e-02[&length_mean=8.20029883e-02,length_median=7.92725400e-02,length_95%HPD={3.64296700e-02,1.33719900e-01}],(175[&prob=1.00000000e+00,prob_stddev=0.00000000e+00,prob_range={1.00000000e+00,1.00000000e+00},prob(percent)="100",prob+-sd="100+-0"]:3.182909e-03[&length_mean=4.66646830e-03,length_median=3.18290900e-03,length_95%HPD={9.80201900e-07,1.42582000e-02}],176[&prob=1.00000000e+00,prob_stddev=0.00000000e+00,prob_range={1.00000000e+00,1.00000000e+00},prob(percent)="100",prob+-sd="100+-0"]:3.202608e-03[&length_mean=4.68150276e-03,length_median=3.20260800e-03,length_95%HPD={4.80514600e-07,1.39815600e-02}])[&prob=1.00000000e+00,prob_stddev=0.00000000e+00,prob_range={1.00000000e+00,1.00000000e+00},prob(percent)="100",prob+-sd="100+-0"]:1.283990e-01[&length_mean=1.30981219e-01,length_median=1.28399000e-01,length_95%HPD={7.35030800e-02,1.88271500e-01}])[&prob=5.42162162e-01,prob_stddev=1.22310362e-02,prob_range={5.33513514e-01,5.50810811e-01},prob(percent)="54",prob+-sd="54+-1"]:3.747039e-02[&length_mean=3.98468800e-02,length_median=3.74703900e-02,length_95%HPD={9.21325500e-03,7.67401500e-02}],177[&prob=1.00000000e+00,prob_stddev=0.00000000e+00,prob_range={1.00000000e+00,1.00000000e+00},prob(percent)="100",prob+-sd="100+-0"]:9.502301e-02[&length_mean=9.69004936e-02,length_median=9.50230100e-02,length_95%HPD={4.62436500e-02,1.53089800e-01}],(178[&prob=1.00000000e+00,prob_stddev=0.00000000e+00,prob_range={1.00000000e+00,1.00000000e+00},prob(percent)="100",prob+-sd="100+-0"]:3.391159e-03[&length_mean=4.84467960e-03,length_median=3.39115900e-03,length_95%HPD={2.52184200e-07,1.46251700e-02}],179[&prob=1.00000000e+00,prob_stddev=0.00000000e+00,prob_range={1.00000000e+00,1.00000000e+00},prob(percent)="100",prob+-sd="100+-0"]:7.707416e-03[&length_mean=9.16771818e-03,length_median=7.70741600e-03,length_95%HPD={1.11190200e-05,2.17963700e-02}])[&prob=1.00000000e+00,prob_stddev=0.00000000e+00,prob_range={1.00000000e+00,1.00000000e+00},prob(percent)="100",prob+-sd="100+-0"]:1.414892e-01[&length_mean=1.43732696e-01,length_median=1.41489200e-01,length_95%HPD={8.02857100e-02,2.13980000e-01}])[&prob=1.00000000e+00,prob_stddev=0.00000000e+00,prob_range={1.00000000e+00,1.00000000e+00},prob(percent)="100",prob+-sd="100+-0"]:3.913650e-01[&length_mean=3.96210539e-01,length_median=3.91365000e-01,length_95%HPD={2.66335100e-01,5.33013500e-01}])[&prob=1.00000000e+00,prob_stddev=0.00000000e+00,prob_range={1.00000000e+00,1.00000000e+00},prob(percent)="100",prob+-sd="100+-0"]:1.778705e-01[&length_mean=1.82070669e-01,length_median=1.77870500e-01,length_95%HPD={8.26512900e-02,3.02452900e-01}])[&prob=1.00000000e+00,prob_stddev=0.00000000e+00,prob_range={1.00000000e+00,1.00000000e+00},prob(percent)="100",prob+-sd="100+-0"]:1.584229e-01[&length_mean=1.60504725e-01,length_median=1.58422900e-01,length_95%HPD={7.48866100e-02,2.49175800e-01}],(((((137[&prob=1.00000000e+00,prob_stddev=0.00000000e+00,prob_range={1.00000000e+00,1.00000000e+00},prob(percent)="100",prob+-sd="100+-0"]:3.504900e-01[&length_mean=3.52745860e-01,length_median=3.50490000e-01,length_95%HPD={2.41147000e-01,4.58826900e-01}],138[&prob=1.00000000e+00,prob_stddev=0.00000000e+00,prob_range={1.00000000e+00,1.00000000e+00},prob(percent)="100",prob+-sd="100+-0"]:1.596667e-02[&length_mean=2.09036667e-02,length_median=1.59666700e-02,length_95%HPD={1.49617900e-06,5.83642400e-02}])[&prob=1.00000000e+00,prob_stddev=0.00000000e+00,prob_range={1.00000000e+00,1.00000000e+00},prob(percent)="100",prob+-sd="100+-0"]:3.771943e-01[&length_mean=3.80265809e-01,length_median=3.77194300e-01,length_95%HPD={2.53276400e-01,5.09674200e-01}],((((139[&prob=1.00000000e+00,prob_stddev=0.00000000e+00,prob_range={1.00000000e+00,1.00000000e+00},prob(percent)="100",prob+-sd="100+-0"]:3.274734e-03[&length_mean=4.62567732e-03,length_median=3.27473400e-03,length_95%HPD={1.05819300e-06,1.35761900e-02}],140[&prob=1.00000000e+00,prob_stddev=0.00000000e+00,prob_range={1.00000000e+00,1.00000000e+00},prob(percent)="100",prob+-sd="100+-0"]:3.146027e-03[&length_mean=4.65369566e-03,length_median=3.14602700e-03,length_95%HPD={2.55182300e-06,1.40717500e-02}])[&prob=1.00000000e+00,prob_stddev=0.00000000e+00,prob_range={1.00000000e+00,1.00000000e+00},prob(percent)="100",prob+-sd="100+-0"]:2.011772e-01[&length_mean=2.02974909e-01,length_median=2.01177200e-01,length_95%HPD={1.31862100e-01,2.80738200e-01}],143[&prob=1.00000000e+00,prob_stddev=0.00000000e+00,prob_range={1.00000000e+00,1.00000000e+00},prob(percent)="100",prob+-sd="100+-0"]:1.721294e-01[&length_mean=1.73795076e-01,length_median=1.72129400e-01,length_95%HPD={1.04561800e-01,2.44964500e-01}])[&prob=9.23513514e-01,prob_stddev=1.03199368e-02,prob_range={9.16216216e-01,9.30810811e-01},prob(percent)="92",prob+-sd="92+-1"]:7.025505e-02[&length_mean=7.29167746e-02,length_median=7.02550500e-02,length_95%HPD={2.10331600e-02,1.34146700e-01}],(141[&prob=1.00000000e+00,prob_stddev=0.00000000e+00,prob_range={1.00000000e+00,1.00000000e+00},prob(percent)="100",prob+-sd="100+-0"]:7.926242e-03[&length_mean=9.48263833e-03,length_median=7.92624200e-03,length_95%HPD={2.51866800e-04,2.25666900e-02}],142[&prob=1.00000000e+00,prob_stddev=0.00000000e+00,prob_range={1.00000000e+00,1.00000000e+00},prob(percent)="100",prob+-sd="100+-0"]:3.398995e-03[&length_mean=4.90668611e-03,length_median=3.39899500e-03,length_95%HPD={2.16726200e-07,1.44739000e-02}])[&prob=1.00000000e+00,prob_stddev=0.00000000e+00,prob_range={1.00000000e+00,1.00000000e+00},prob(percent)="100",prob+-sd="100+-0"]:1.284580e-01[&length_mean=1.30568925e-01,length_median=1.28458000e-01,length_95%HPD={6.87319400e-02,2.01388200e-01}])[&prob=9.97567568e-01,prob_stddev=3.82219882e-04,prob_range={9.97297297e-01,9.97837838e-01},prob(percent)="100",prob+-sd="100+-0"]:8.978863e-02[&length_mean=9.27333019e-02,length_median=8.97886300e-02,length_95%HPD={2.29292700e-02,1.70394200e-01}],(154[&prob=1.00000000e+00,prob_stddev=0.00000000e+00,prob_range={1.00000000e+00,1.00000000e+00},prob(percent)="100",prob+-sd="100+-0"]:3.693654e-03[&length_mean=5.24490224e-03,length_median=3.69365400e-03,length_95%HPD={1.71549500e-07,1.58132300e-02}],155[&prob=1.00000000e+00,prob_stddev=0.00000000e+00,prob_range={1.00000000e+00,1.00000000e+00},prob(percent)="100",prob+-sd="100+-0"]:7.232720e-03[&length_mean=8.71173988e-03,length_median=7.23272000e-03,length_95%HPD={4.52582600e-05,2.13006800e-02}])[&prob=1.00000000e+00,prob_stddev=0.00000000e+00,prob_range={1.00000000e+00,1.00000000e+00},prob(percent)="100",prob+-sd="100+-0"]:5.296331e-01[&length_mean=5.35584711e-01,length_median=5.29633100e-01,length_95%HPD={3.97297400e-01,7.01302800e-01}])[&prob=8.77297297e-01,prob_stddev=3.82219882e-03,prob_range={8.74594595e-01,8.80000000e-01},prob(percent)="88",prob+-sd="88+-0"]:7.034231e-02[&length_mean=7.45259693e-02,length_median=7.03423100e-02,length_95%HPD={1.00839200e-02,1.44940800e-01}])[&prob=8.76216216e-01,prob_stddev=3.82219882e-03,prob_range={8.73513514e-01,8.78918919e-01},prob(percent)="88",prob+-sd="88+-0"]:8.305422e-02[&length_mean=8.91903565e-02,length_median=8.30542200e-02,length_95%HPD={4.05617700e-03,1.77186200e-01}],156[&prob=1.00000000e+00,prob_stddev=0.00000000e+00,prob_range={1.00000000e+00,1.00000000e+00},prob(percent)="100",prob+-sd="100+-0"]:5.214431e-01[&length_mean=5.25349225e-01,length_median=5.21443100e-01,length_95%HPD={3.42528400e-01,7.08044000e-01}])[&prob=8.33243243e-01,prob_stddev=1.79643344e-02,prob_range={8.20540541e-01,8.45945946e-01},prob(percent)="83",prob+-sd="83+-2"]:8.701659e-02[&length_mean=9.26360317e-02,length_median=8.70165900e-02,length_95%HPD={1.35248600e-02,1.81668200e-01}],144[&prob=1.00000000e+00,prob_stddev=0.00000000e+00,prob_range={1.00000000e+00,1.00000000e+00},prob(percent)="100",prob+-sd="100+-0"]:1.891329e-01[&length_mean=1.92522107e-01,length_median=1.89132900e-01,length_95%HPD={1.11873800e-01,2.89228800e-01}])[&prob=9.83513514e-01,prob_stddev=1.91109941e-03,prob_range={9.82162162e-01,9.84864865e-01},prob(percent)="98",prob+-sd="98+-0"]:1.892772e-01[&length_mean=1.90502134e-01,length_median=1.89277200e-01,length_95%HPD={8.22327200e-02,2.99940000e-01}],((((157[&prob=1.00000000e+00,prob_stddev=0.00000000e+00,prob_range={1.00000000e+00,1.00000000e+00},prob(percent)="100",prob+-sd="100+-0"]:3.319110e-03[&length_mean=4.83771680e-03,length_median=3.31911000e-03,length_95%HPD={5.75347100e-07,1.45768700e-02}],158[&prob=1.00000000e+00,prob_stddev=0.00000000e+00,prob_range={1.00000000e+00,1.00000000e+00},prob(percent)="100",prob+-sd="100+-0"]:7.952400e-03[&length_mean=9.63273113e-03,length_median=7.95240000e-03,length_95%HPD={9.79528900e-05,2.35278200e-02}])[&prob=1.00000000e+00,prob_stddev=0.00000000e+00,prob_range={1.00000000e+00,1.00000000e+00},prob(percent)="100",prob+-sd="100+-0"]:7.229683e-02[&length_mean=7.41145245e-02,length_median=7.22968300e-02,length_95%HPD={2.89751900e-02,1.27407800e-01}],159[&prob=1.00000000e+00,prob_stddev=0.00000000e+00,prob_range={1.00000000e+00,1.00000000e+00},prob(percent)="100",prob+-sd="100+-0"]:8.823614e-02[&length_mean=9.05541048e-02,length_median=8.82361400e-02,length_95%HPD={4.22384200e-02,1.47945800e-01}])[&prob=1.00000000e+00,prob_stddev=0.00000000e+00,prob_range={1.00000000e+00,1.00000000e+00},prob(percent)="100",prob+-sd="100+-0"]:2.407891e-01[&length_mean=2.44025502e-01,length_median=2.40789100e-01,length_95%HPD={1.47044300e-01,3.38285500e-01}],((160[&prob=1.00000000e+00,prob_stddev=0.00000000e+00,prob_range={1.00000000e+00,1.00000000e+00},prob(percent)="100",prob+-sd="100+-0"]:3.465538e-03[&length_mean=5.11340292e-03,length_median=3.46553800e-03,length_95%HPD={1.33441700e-06,1.53086800e-02}],161[&prob=1.00000000e+00,prob_stddev=0.00000000e+00,prob_range={1.00000000e+00,1.00000000e+00},prob(percent)="100",prob+-sd="100+-0"]:3.588437e-03[&length_mean=5.15704191e-03,length_median=3.58843700e-03,length_95%HPD={1.41148800e-06,1.54228400e-02}])[&prob=5.36486486e-01,prob_stddev=1.91109941e-03,prob_range={5.35135135e-01,5.37837838e-01},prob(percent)="54",prob+-sd="54+-0"]:6.497920e-03[&length_mean=8.15979790e-03,length_median=6.49792000e-03,length_95%HPD={5.35286600e-06,2.18233300e-02}],162[&prob=1.00000000e+00,prob_stddev=0.00000000e+00,prob_range={1.00000000e+00,1.00000000e+00},prob(percent)="100",prob+-sd="100+-0"]:1.214181e-02[&length_mean=1.36221772e-02,length_median=1.21418100e-02,length_95%HPD={3.36022300e-04,3.01069000e-02}])[&prob=1.00000000e+00,prob_stddev=0.00000000e+00,prob_range={1.00000000e+00,1.00000000e+00},prob(percent)="100",prob+-sd="100+-0"]:1.861545e-01[&length_mean=1.88293494e-01,length_median=1.86154500e-01,length_95%HPD={1.08059100e-01,2.72616800e-01}])[&prob=1.00000000e+00,prob_stddev=0.00000000e+00,prob_range={1.00000000e+00,1.00000000e+00},prob(percent)="100",prob+-sd="100+-0"]:1.280570e-01[&length_mean=1.31593138e-01,length_median=1.28057000e-01,length_95%HPD={4.56438400e-02,2.14902800e-01}],((163[&prob=1.00000000e+00,prob_stddev=0.00000000e+00,prob_range={1.00000000e+00,1.00000000e+00},prob(percent)="100",prob+-sd="100+-0"]:7.358452e-02[&length_mean=7.55224042e-02,length_median=7.35845200e-02,length_95%HPD={3.84051300e-02,1.14647700e-01}],164[&prob=1.00000000e+00,prob_stddev=0.00000000e+00,prob_range={1.00000000e+00,1.00000000e+00},prob(percent)="100",prob+-sd="100+-0"]:3.364242e-03[&length_mean=4.89738065e-03,length_median=3.36424200e-03,length_95%HPD={2.45743900e-06,1.45987000e-02}])[&prob=1.00000000e+00,prob_stddev=0.00000000e+00,prob_range={1.00000000e+00,1.00000000e+00},prob(percent)="100",prob+-sd="100+-0"]:5.499360e-02[&length_mean=5.65324189e-02,length_median=5.49936000e-02,length_95%HPD={2.16934400e-02,9.11338400e-02}],165[&prob=1.00000000e+00,prob_stddev=0.00000000e+00,prob_range={1.00000000e+00,1.00000000e+00},prob(percent)="100",prob+-sd="100+-0"]:8.069030e-03[&length_mean=9.54506897e-03,length_median=8.06903000e-03,length_95%HPD={4.21186100e-05,2.23607100e-02}],166[&prob=1.00000000e+00,prob_stddev=0.00000000e+00,prob_range={1.00000000e+00,1.00000000e+00},prob(percent)="100",prob+-sd="100+-0"]:3.263575e-03[&length_mean=4.65594695e-03,length_median=3.26357500e-03,length_95%HPD={7.10412300e-07,1.38407900e-02}],167[&prob=1.00000000e+00,prob_stddev=0.00000000e+00,prob_range={1.00000000e+00,1.00000000e+00},prob(percent)="100",prob+-sd="100+-0"]:3.076470e-03[&length_mean=4.51451036e-03,length_median=3.07647000e-03,length_95%HPD={4.01039200e-07,1.36953400e-02}],168[&prob=1.00000000e+00,prob_stddev=0.00000000e+00,prob_range={1.00000000e+00,1.00000000e+00},prob(percent)="100",prob+-sd="100+-0"]:3.091753e-03[&length_mean=4.46823255e-03,length_median=3.09175300e-03,length_95%HPD={6.63657100e-08,1.33845300e-02}])[&prob=1.00000000e+00,prob_stddev=0.00000000e+00,prob_range={1.00000000e+00,1.00000000e+00},prob(percent)="100",prob+-sd="100+-0"]:1.927312e-01[&length_mean=1.95877498e-01,length_median=1.92731200e-01,length_95%HPD={1.11663800e-01,2.83174600e-01}])[&prob=1.00000000e+00,prob_stddev=0.00000000e+00,prob_range={1.00000000e+00,1.00000000e+00},prob(percent)="100",prob+-sd="100+-0"]:2.502411e-01[&length_mean=2.53912024e-01,length_median=2.50241100e-01,length_95%HPD={1.45677400e-01,3.65646300e-01}])[&prob=9.74324324e-01,prob_stddev=1.91109941e-03,prob_range={9.72972973e-01,9.75675676e-01},prob(percent)="97",prob+-sd="97+-0"]:1.015749e-01[&length_mean=1.05324560e-01,length_median=1.01574900e-01,length_95%HPD={3.83369200e-02,1.88836900e-01}],(152[&prob=1.00000000e+00,prob_stddev=0.00000000e+00,prob_range={1.00000000e+00,1.00000000e+00},prob(percent)="100",prob+-sd="100+-0"]:6.755548e-03[&length_mean=9.28211313e-03,length_median=6.75554800e-03,length_95%HPD={2.53930100e-06,2.63931900e-02}],153[&prob=1.00000000e+00,prob_stddev=0.00000000e+00,prob_range={1.00000000e+00,1.00000000e+00},prob(percent)="100",prob+-sd="100+-0"]:2.495112e-02[&length_mean=2.64844378e-02,length_median=2.49511200e-02,length_95%HPD={3.83154800e-03,5.05787400e-02}])[&prob=1.00000000e+00,prob_stddev=0.00000000e+00,prob_range={1.00000000e+00,1.00000000e+00},prob(percent)="100",prob+-sd="100+-0"]:5.059296e-01[&length_mean=5.11720049e-01,length_median=5.05929600e-01,length_95%HPD={3.64235000e-01,6.64044800e-01}],171[&prob=1.00000000e+00,prob_stddev=0.00000000e+00,prob_range={1.00000000e+00,1.00000000e+00},prob(percent)="100",prob+-sd="100+-0"]:3.930362e-01[&length_mean=3.95222391e-01,length_median=3.93036200e-01,length_95%HPD={2.65254600e-01,5.31856300e-01}],180[&prob=1.00000000e+00,prob_stddev=0.00000000e+00,prob_range={1.00000000e+00,1.00000000e+00},prob(percent)="100",prob+-sd="100+-0"]:4.873762e-01[&length_mean=4.89411059e-01,length_median=4.87376200e-01,length_95%HPD={3.48685500e-01,6.45397400e-01}],(181[&prob=1.00000000e+00,prob_stddev=0.00000000e+00,prob_range={1.00000000e+00,1.00000000e+00},prob(percent)="100",prob+-sd="100+-0"]:3.260336e-03[&length_mean=4.67840471e-03,length_median=3.26033600e-03,length_95%HPD={3.15538900e-06,1.38309300e-02}],182[&prob=1.00000000e+00,prob_stddev=0.00000000e+00,prob_range={1.00000000e+00,1.00000000e+00},prob(percent)="100",prob+-sd="100+-0"]:3.306381e-03[&length_mean=4.62191976e-03,length_median=3.30638100e-03,length_95%HPD={1.09149000e-06,1.36016700e-02}])[&prob=1.00000000e+00,prob_stddev=0.00000000e+00,prob_range={1.00000000e+00,1.00000000e+00},prob(percent)="100",prob+-sd="100+-0"]:5.137690e-01[&length_mean=5.16030709e-01,length_median=5.13769000e-01,length_95%HPD={3.67328000e-01,6.79094800e-01}])[&prob=8.26486486e-01,prob_stddev=3.13420303e-02,prob_range={8.04324324e-01,8.48648649e-01},prob(percent)="83",prob+-sd="83+-3"]:9.134861e-02[&length_mean=9.63167056e-02,length_median=9.13486100e-02,length_95%HPD={1.30217200e-02,1.87113600e-01}],((184[&prob=1.00000000e+00,prob_stddev=0.00000000e+00,prob_range={1.00000000e+00,1.00000000e+00},prob(percent)="100",prob+-sd="100+-0"]:3.027813e-03[&length_mean=4.59389013e-03,length_median=3.02781300e-03,length_95%HPD={1.07744600e-07,1.39770900e-02}],185[&prob=1.00000000e+00,prob_stddev=0.00000000e+00,prob_range={1.00000000e+00,1.00000000e+00},prob(percent)="100",prob+-sd="100+-0"]:3.247939e-03[&length_mean=4.61247395e-03,length_median=3.24793900e-03,length_95%HPD={1.28566800e-06,1.37988800e-02}])[&prob=9.97837838e-01,prob_stddev=1.52887953e-03,prob_range={9.96756757e-01,9.98918919e-01},prob(percent)="100",prob+-sd="100+-0"]:5.809739e-02[&length_mean=6.06613003e-02,length_median=5.80973900e-02,length_95%HPD={1.48725400e-02,1.17115500e-01}],186[&prob=1.00000000e+00,prob_stddev=0.00000000e+00,prob_range={1.00000000e+00,1.00000000e+00},prob(percent)="100",prob+-sd="100+-0"]:9.956447e-02[&length_mean=1.01742908e-01,length_median=9.95644700e-02,length_95%HPD={4.49934200e-02,1.63424200e-01}])[&prob=1.00000000e+00,prob_stddev=0.00000000e+00,prob_range={1.00000000e+00,1.00000000e+00},prob(percent)="100",prob+-sd="100+-0"]:4.687615e-01[&length_mean=4.72352404e-01,length_median=4.68761500e-01,length_95%HPD={3.25029600e-01,6.30939100e-01}])[&prob=9.74324324e-01,prob_stddev=3.24886899e-02,prob_range={9.51351351e-01,9.97297297e-01},prob(percent)="97",prob+-sd="97+-3"]:2.452006e-01[&length_mean=2.51469104e-01,length_median=2.45200600e-01,length_95%HPD={1.01324500e-01,4.10032500e-01}])[&prob=1.00000000e+00,prob_stddev=0.00000000e+00,prob_range={1.00000000e+00,1.00000000e+00},prob(percent)="100",prob+-sd="100+-0"]:3.996648e-01[&length_mean=4.04690197e-01,length_median=3.99664800e-01,length_95%HPD={2.30152500e-01,5.80649100e-01}])[&prob=5.02702703e-01,prob_stddev=9.17327716e-03,prob_range={4.96216216e-01,5.09189189e-01},prob(percent)="50",prob+-sd="50+-1"]:7.840975e-02[&length_mean=8.42540534e-02,length_median=7.84097500e-02,length_95%HPD={1.05542400e-04,1.78140600e-01}])[&prob=8.41081081e-01,prob_stddev=6.87995787e-03,prob_range={8.36216216e-01,8.45945946e-01},prob(percent)="84",prob+-sd="84+-1"]:7.633939e-02[&length_mean=7.99647902e-02,length_median=7.63393900e-02,length_95%HPD={1.10564400e-02,1.53164700e-01}])[&prob=9.56756757e-01,prob_stddev=1.52887953e-03,prob_range={9.55675676e-01,9.57837838e-01},prob(percent)="96",prob+-sd="96+-0"]:7.349468e-02[&length_mean=7.79753280e-02,length_median=7.34946800e-02,length_95%HPD={1.40188000e-02,1.50788700e-01}],(((((((26[&prob=1.00000000e+00,prob_stddev=0.00000000e+00,prob_range={1.00000000e+00,1.00000000e+00},prob(percent)="100",prob+-sd="100+-0"]:1.826109e-02[&length_mean=2.06259050e-02,length_median=1.82610900e-02,length_95%HPD={6.58829700e-04,4.51697300e-02}],27[&prob=1.00000000e+00,prob_stddev=0.00000000e+00,prob_range={1.00000000e+00,1.00000000e+00},prob(percent)="100",prob+-sd="100+-0"]:2.185800e-02[&length_mean=2.46440826e-02,length_median=2.18580000e-02,length_95%HPD={4.68567800e-04,5.37860600e-02}])[&prob=1.00000000e+00,prob_stddev=0.00000000e+00,prob_range={1.00000000e+00,1.00000000e+00},prob(percent)="100",prob+-sd="100+-0"]:1.829205e-01[&length_mean=1.85610712e-01,length_median=1.82920500e-01,length_95%HPD={1.20341400e-01,2.56155800e-01}],(31[&prob=1.00000000e+00,prob_stddev=0.00000000e+00,prob_range={1.00000000e+00,1.00000000e+00},prob(percent)="100",prob+-sd="100+-0"]:7.756227e-02[&length_mean=7.95612276e-02,length_median=7.75622700e-02,length_95%HPD={3.96064900e-02,1.22768800e-01}],32[&prob=1.00000000e+00,prob_stddev=0.00000000e+00,prob_range={1.00000000e+00,1.00000000e+00},prob(percent)="100",prob+-sd="100+-0"]:1.443112e-01[&length_mean=1.46261468e-01,length_median=1.44311200e-01,length_95%HPD={9.07540700e-02,2.02812900e-01}])[&prob=9.87027027e-01,prob_stddev=1.52887953e-03,prob_range={9.85945946e-01,9.88108108e-01},prob(percent)="99",prob+-sd="99+-0"]:3.094814e-02[&length_mean=3.33288416e-02,length_median=3.09481400e-02,length_95%HPD={6.50223900e-03,6.46569200e-02}])[&prob=1.00000000e+00,prob_stddev=0.00000000e+00,prob_range={1.00000000e+00,1.00000000e+00},prob(percent)="100",prob+-sd="100+-0"]:6.878291e-02[&length_mean=7.12594441e-02,length_median=6.87829100e-02,length_95%HPD={2.20052200e-02,1.20237200e-01}],(28[&prob=1.00000000e+00,prob_stddev=0.00000000e+00,prob_range={1.00000000e+00,1.00000000e+00},prob(percent)="100",prob+-sd="100+-0"]:3.082652e-02[&length_mean=3.28666420e-02,length_median=3.08265200e-02,length_95%HPD={6.04903600e-03,6.50011100e-02}],(29[&prob=1.00000000e+00,prob_stddev=0.00000000e+00,prob_range={1.00000000e+00,1.00000000e+00},prob(percent)="100",prob+-sd="100+-0"]:2.174609e-02[&length_mean=2.39727809e-02,length_median=2.17460900e-02,length_95%HPD={1.62537900e-03,5.11247600e-02}],30[&prob=1.00000000e+00,prob_stddev=0.00000000e+00,prob_range={1.00000000e+00,1.00000000e+00},prob(percent)="100",prob+-sd="100+-0"]:9.550349e-02[&length_mean=9.70876622e-02,length_median=9.55034900e-02,length_95%HPD={5.20915700e-02,1.48205700e-01}])[&prob=7.83783784e-01,prob_stddev=2.29331929e-03,prob_range={7.82162162e-01,7.85405405e-01},prob(percent)="78",prob+-sd="78+-0"]:1.379807e-02[&length_mean=1.59287305e-02,length_median=1.37980700e-02,length_95%HPD={8.45369300e-06,3.60482200e-02}])[&prob=8.43243243e-01,prob_stddev=2.36976327e-02,prob_range={8.26486486e-01,8.60000000e-01},prob(percent)="84",prob+-sd="84+-2"]:2.985665e-02[&length_mean=3.22578795e-02,length_median=2.98566500e-02,length_95%HPD={4.01005700e-04,6.64620400e-02}])[&prob=1.00000000e+00,prob_stddev=0.00000000e+00,prob_range={1.00000000e+00,1.00000000e+00},prob(percent)="100",prob+-sd="100+-0"]:3.874380e-01[&length_mean=3.91430726e-01,length_median=3.87438000e-01,length_95%HPD={2.69147700e-01,5.39892800e-01}],35[&prob=1.00000000e+00,prob_stddev=0.00000000e+00,prob_range={1.00000000e+00,1.00000000e+00},prob(percent)="100",prob+-sd="100+-0"]:4.032611e-01[&length_mean=4.07825084e-01,length_median=4.03261100e-01,length_95%HPD={2.76705000e-01,5.48312900e-01}])[&prob=9.79459459e-01,prob_stddev=7.64439763e-03,prob_range={9.74054054e-01,9.84864865e-01},prob(percent)="98",prob+-sd="98+-1"]:1.288313e-01[&length_mean=1.33165436e-01,length_median=1.28831300e-01,length_95%HPD={4.46913700e-02,2.34059500e-01}],34[&prob=1.00000000e+00,prob_stddev=0.00000000e+00,prob_range={1.00000000e+00,1.00000000e+00},prob(percent)="100",prob+-sd="100+-0"]:1.746111e-01[&length_mean=1.77592601e-01,length_median=1.74611100e-01,length_95%HPD={8.77073100e-02,2.75268900e-01}])[&prob=9.61891892e-01,prob_stddev=3.82219882e-04,prob_range={9.61621622e-01,9.62162162e-01},prob(percent)="96",prob+-sd="96+-0"]:1.178298e-01[&length_mean=1.21736019e-01,length_median=1.17829800e-01,length_95%HPD={3.92501000e-02,2.09004300e-01}],33[&prob=1.00000000e+00,prob_stddev=0.00000000e+00,prob_range={1.00000000e+00,1.00000000e+00},prob(percent)="100",prob+-sd="100+-0"]:3.300740e-01[&length_mean=3.33535884e-01,length_median=3.30074000e-01,length_95%HPD={2.20306800e-01,4.48992100e-01}])[&prob=8.53243243e-01,prob_stddev=1.91109941e-03,prob_range={8.51891892e-01,8.54594595e-01},prob(percent)="85",prob+-sd="85+-0"]:8.286104e-02[&length_mean=8.86664381e-02,length_median=8.28610400e-02,length_95%HPD={4.30700600e-03,1.83096400e-01}],38[&prob=1.00000000e+00,prob_stddev=0.00000000e+00,prob_range={1.00000000e+00,1.00000000e+00},prob(percent)="100",prob+-sd="100+-0"]:4.456744e-01[&length_mean=4.51475061e-01,length_median=4.45674400e-01,length_95%HPD={2.99021000e-01,6.30651200e-01}])[&prob=9.99459459e-01,prob_stddev=0.00000000e+00,prob_range={9.99459459e-01,9.99459459e-01},prob(percent)="100",prob+-sd="100+-0"]:1.784204e-01[&length_mean=1.81463796e-01,length_median=1.78420400e-01,length_95%HPD={6.09953000e-02,3.06899600e-01}])[&prob=8.23513514e-01,prob_stddev=6.49773799e-03,prob_range={8.18918919e-01,8.28108108e-01},prob(percent)="82",prob+-sd="82+-1"]:6.063560e-02[&length_mean=6.40008381e-02,length_median=6.06356000e-02,length_95%HPD={7.96434700e-03,1.26237300e-01}],(19[&prob=1.00000000e+00,prob_stddev=0.00000000e+00,prob_range={1.00000000e+00,1.00000000e+00},prob(percent)="100",prob+-sd="100+-0"]:2.794709e-02[&length_mean=2.97285331e-02,length_median=2.79470900e-02,length_95%HPD={6.89813100e-03,5.65593600e-02}],20[&prob=1.00000000e+00,prob_stddev=0.00000000e+00,prob_range={1.00000000e+00,1.00000000e+00},prob(percent)="100",prob+-sd="100+-0"]:1.429815e-02[&length_mean=1.63330681e-02,length_median=1.42981500e-02,length_95%HPD={1.44929600e-06,3.69952100e-02}])[&prob=1.00000000e+00,prob_stddev=0.00000000e+00,prob_range={1.00000000e+00,1.00000000e+00},prob(percent)="100",prob+-sd="100+-0"]:3.395614e-01[&length_mean=3.41792361e-01,length_median=3.39561400e-01,length_95%HPD={2.28669800e-01,4.59132800e-01}])[&prob=1.00000000e+00,prob_stddev=0.00000000e+00,prob_range={1.00000000e+00,1.00000000e+00},prob(percent)="100",prob+-sd="100+-0"]:1.619836e-01[&length_mean=1.65454193e-01,length_median=1.61983600e-01,length_95%HPD={7.10713300e-02,2.64519400e-01}],((43[&prob=1.00000000e+00,prob_stddev=0.00000000e+00,prob_range={1.00000000e+00,1.00000000e+00},prob(percent)="100",prob+-sd="100+-0"]:1.115581e-02[&length_mean=1.25289348e-02,length_median=1.11558100e-02,length_95%HPD={1.67361600e-03,2.75433500e-02}],44[&prob=1.00000000e+00,prob_stddev=0.00000000e+00,prob_range={1.00000000e+00,1.00000000e+00},prob(percent)="100",prob+-sd="100+-0"]:3.071712e-03[&length_mean=4.35053829e-03,length_median=3.07171200e-03,length_95%HPD={1.35720100e-06,1.32921500e-02}])[&prob=1.00000000e+00,prob_stddev=0.00000000e+00,prob_range={1.00000000e+00,1.00000000e+00},prob(percent)="100",prob+-sd="100+-0"]:1.301559e-01[&length_mean=1.32309679e-01,length_median=1.30155900e-01,length_95%HPD={6.05123200e-02,2.08842300e-01}],(45[&prob=1.00000000e+00,prob_stddev=0.00000000e+00,prob_range={1.00000000e+00,1.00000000e+00},prob(percent)="100",prob+-sd="100+-0"]:2.904607e-03[&length_mean=4.19448046e-03,length_median=2.90460700e-03,length_95%HPD={1.33194300e-06,1.24862800e-02}],46[&prob=1.00000000e+00,prob_stddev=0.00000000e+00,prob_range={1.00000000e+00,1.00000000e+00},prob(percent)="100",prob+-sd="100+-0"]:2.986487e-03[&length_mean=4.27837065e-03,length_median=2.98648700e-03,length_95%HPD={1.48106100e-06,1.26963800e-02}])[&prob=1.00000000e+00,prob_stddev=0.00000000e+00,prob_range={1.00000000e+00,1.00000000e+00},prob(percent)="100",prob+-sd="100+-0"]:1.724423e-01[&length_mean=1.74970850e-01,length_median=1.72442300e-01,length_95%HPD={9.93386700e-02,2.57137800e-01}])[&prob=1.00000000e+00,prob_stddev=0.00000000e+00,prob_range={1.00000000e+00,1.00000000e+00},prob(percent)="100",prob+-sd="100+-0"]:5.957209e-01[&length_mean=6.00400560e-01,length_median=5.95720900e-01,length_95%HPD={4.36180600e-01,7.90461700e-01}])[&prob=9.33783784e-01,prob_stddev=1.03199368e-02,prob_range={9.26486486e-01,9.41081081e-01},prob(percent)="93",prob+-sd="93+-1"]:6.818975e-02[&length_mean=7.21085388e-02,length_median=6.81897500e-02,length_95%HPD={7.50974400e-03,1.41121600e-01}])[&prob=9.98648649e-01,prob_stddev=1.91109941e-03,prob_range={9.97297297e-01,1.00000000e+00},prob(percent)="100",prob+-sd="100+-0"]:8.959927e-02[&length_mean=9.15208446e-02,length_median=8.95992700e-02,length_95%HPD={3.55309200e-02,1.54422500e-01}])[&prob=1.00000000e+00,prob_stddev=0.00000000e+00,prob_range={1.00000000e+00,1.00000000e+00},prob(percent)="100",prob+-sd="100+-0"]:7.680450e-02[&length_mean=7.96754121e-02,length_median=7.68045000e-02,length_95%HPD={2.95199400e-02,1.34933300e-01}],11[&prob=1.00000000e+00,prob_stddev=0.00000000e+00,prob_range={1.00000000e+00,1.00000000e+00},prob(percent)="100",prob+-sd="100+-0"]:2.363743e-01[&length_mean=2.39711762e-01,length_median=2.36374300e-01,length_95%HPD={1.60753600e-01,3.20402000e-01}])[&prob=7.56216216e-01,prob_stddev=1.52887953e-03,prob_range={7.55135135e-01,7.57297297e-01},prob(percent)="76",prob+-sd="76+-0"]:3.121648e-02[&length_mean=3.35138146e-02,length_median=3.12164800e-02,length_95%HPD={5.66342600e-03,6.66754500e-02}],(9[&prob=1.00000000e+00,prob_stddev=0.00000000e+00,prob_range={1.00000000e+00,1.00000000e+00},prob(percent)="100",prob+-sd="100+-0"]:1.254981e-02[&length_mean=1.81909288e-02,length_median=1.25498100e-02,length_95%HPD={1.18226800e-06,5.42635400e-02}],15[&prob=1.00000000e+00,prob_stddev=0.00000000e+00,prob_range={1.00000000e+00,1.00000000e+00},prob(percent)="100",prob+-sd="100+-0"]:3.382202e-02[&length_mean=4.43932968e-02,length_median=3.38220200e-02,length_95%HPD={3.58766600e-05,1.21964700e-01}])[&prob=1.00000000e+00,prob_stddev=0.00000000e+00,prob_range={1.00000000e+00,1.00000000e+00},prob(percent)="100",prob+-sd="100+-0"]:1.699486e-01[&length_mean=1.73494830e-01,length_median=1.69948600e-01,length_95%HPD={8.58950600e-02,2.67584600e-01}])[&prob=6.22972973e-01,prob_stddev=4.20441870e-03,prob_range={6.20000000e-01,6.25945946e-01},prob(percent)="62",prob+-sd="62+-0"]:2.065400e-02[&length_mean=2.38533816e-02,length_median=2.06540000e-02,length_95%HPD={6.49754100e-05,5.67583400e-02}])[&prob=8.35945946e-01,prob_stddev=3.43997894e-03,prob_range={8.33513514e-01,8.38378378e-01},prob(percent)="84",prob+-sd="84+-0"]:2.958045e-02[&length_mean=3.30306342e-02,length_median=2.95804500e-02,length_95%HPD={6.51461000e-04,6.84543200e-02}],10[&prob=1.00000000e+00,prob_stddev=0.00000000e+00,prob_range={1.00000000e+00,1.00000000e+00},prob(percent)="100",prob+-sd="100+-0"]:2.080857e-01[&length_mean=2.11312680e-01,length_median=2.08085700e-01,length_95%HPD={1.30521700e-01,3.04295400e-01}])[&prob=1.00000000e+00,prob_stddev=0.00000000e+00,prob_range={1.00000000e+00,1.00000000e+00},prob(percent)="100",prob+-sd="100+-0"]:1.029165e-01[&length_mean=1.05238487e-01,length_median=1.02916500e-01,length_95%HPD={4.98492000e-02,1.66854400e-01}],(2[&prob=1.00000000e+00,prob_stddev=0.00000000e+00,prob_range={1.00000000e+00,1.00000000e+00},prob(percent)="100",prob+-sd="100+-0"]:3.155339e-02[&length_mean=3.35692326e-02,length_median=3.15533900e-02,length_95%HPD={7.64624100e-03,6.20982200e-02}],3[&prob=1.00000000e+00,prob_stddev=0.00000000e+00,prob_range={1.00000000e+00,1.00000000e+00},prob(percent)="100",prob+-sd="100+-0"]:8.824597e-02[&length_mean=8.99490008e-02,length_median=8.82459700e-02,length_95%HPD={4.75240000e-02,1.38376900e-01}])[&prob=6.47297297e-01,prob_stddev=1.26132561e-02,prob_range={6.38378378e-01,6.56216216e-01},prob(percent)="65",prob+-sd="65+-1"]:3.003010e-02[&length_mean=3.19575455e-02,length_median=3.00301000e-02,length_95%HPD={3.55011000e-03,6.18335100e-02}]);

end;
